# A well-defined NHC–Ir(iii) catalyst for the silylation of aromatic C–H bonds: substrate survey and mechanistic insights†
†Electronic supplementary information (ESI) available: Spectroscopic and analytical data. DFT optimized structures and computational details. X-ray crystallographic data for **1**, **2** and **3** (CCDC 1507888, 1507890 and 1507889, respectively). For ESI and crystallographic data in CIF or other electronic format see DOI: 10.1039/c6sc04899d


**DOI:** 10.1039/c6sc04899d

**Published:** 2017-04-05

**Authors:** Laura Rubio-Pérez, Manuel Iglesias, Julen Munárriz, Victor Polo, Vincenzo Passarelli, Jesús J. Pérez-Torrente, Luis A. Oro

**Affiliations:** a Departamento Química Inorgánica – ISQCH , Universidad de Zaragoza – CSIC , Pedro Cerbuna 12 , 50009 Zaragoza , Spain . Email: miglesia@unizar.es ; Email: oro@unizar.es; b Departamento Química Física – Instituto de Biocomputación y Física de Sistemas Complejos (BIFI) , Universidad de Zaragoza , Pedro Cerbuna 12 , 50009 Zaragoza , Spain; c Centro Universitario de la Defensa , Ctra. Huesca s/n , ES-50090 Zaragoza , Spain; d King Fahd University of Petroleum & Minerals (KFUPM) , Dhahran 31261 , Saudi Arabia

## Abstract

A well-defined NHC–Ir(iii) catalyst, [Ir(H)_2_(IPr)(py)_3_][BF_4_] (IPr = 1,3-bis-(2,6-diisopropylphenyl)imidazol-2-ylidene), that provides access to a wide range of aryl- and heteroaryl-silanes by intermolecular dehydrogenative C–H bond silylation has been prepared and fully characterized. The directed and non-directed functionalisation of C–H bonds has been accomplished successfully using an arene as the limiting reagent and a variety of hydrosilanes in excess, including Et_3_SiH, Ph_2_MeSiH, PhMe_2_SiH, Ph_3_SiH and (EtO)_3_SiH. Examples that show unexpected selectivity patterns that stem from the presence of aromatic substituents in hydrosilanes are also presented. The selective bisarylation of bis(hydrosilane)s by directed or non-directed silylation of C–H bonds is also reported herein. Theoretical calculations at the DFT level shed light on the intermediate species in the catalytic cycle and the role played by the ligand system on the Ir(iii)/Ir(i) mechanism.

## Abstract 2

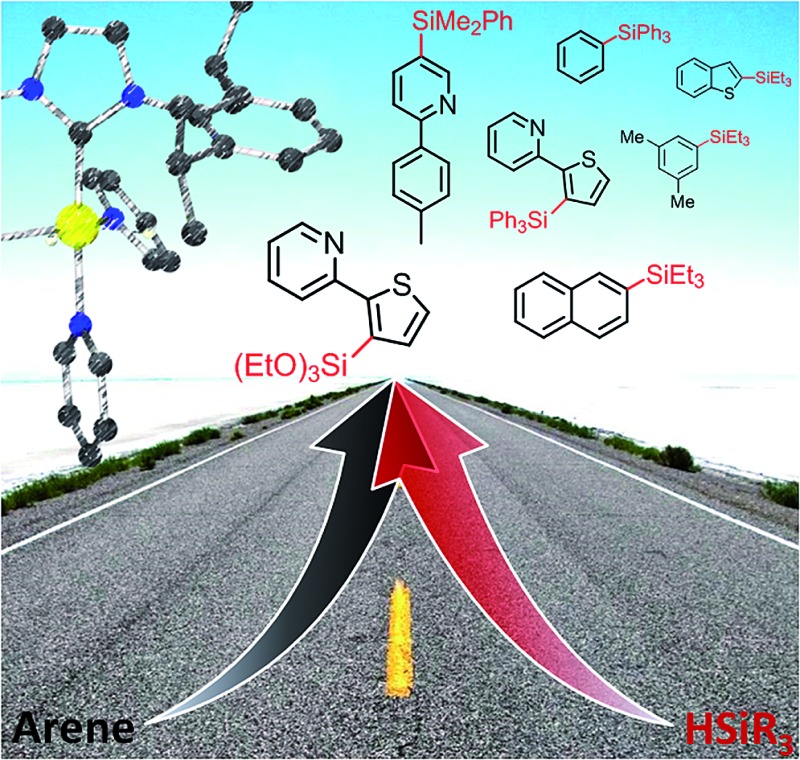
A well-defined NHC-Ir(iii) catalyst provides access to a wide range of aryl- and heteroarylsilanes by intermolecular dehydrogenative C–H bond silylation.

## Introduction

Organosilicon compounds are key building blocks in modern organic synthesis, often used as intermediates for complex molecules or monomers for silicone polymers. The synthetic versatility of organosilanes can be attributed to their straightforward functionalisation by various organic transformations, together with the low cost and non-toxic nature of silicon reagents.^1^ Moreover, conjugated organosilicon materials are attractive targets *per se* owing to their unique properties, which permit widespread applicability in the fields of organic electronics and photonics.^2,3^

The preparation of organosilanes by catalytic silylation of C–H bonds represents a more atom- and step-efficient alternative to stoichiometric processes^4^ and cross-coupling reactions.^5^ The silylation of arenes and heteroarenes, in particular, is an important reaction due to the ubiquitous presence of these moieties in natural products and materials. These reactions are typically divided into two main groups: intermolecular and intramolecular. The former requires prefunctionalisation of the (hetero)arene with a hydrosilane moiety, which may be achieved by hydrosilylation or dehydrogenative silylation using di(hydro)silanes.^6^ Intermolecular silylations may be classified into directed and undirected reactions. Directed silylations require the presence of a coordinating group in the substrate that reversibly binds to the catalyst. This interaction leaves a C–H bond in the proximity of the active site, which facilitates its activation and defines the selectivity of the process. These reactions mostly use disilanes^7^ or hydrosilanes as silicon sources. The latter usually requires the presence of a hydrogen acceptor,^8^ although acceptor-less reactions have also been described.^9^ Undirected silylation reactions, on the other hand, make use of substrates that lack a coordinating group that is able to direct the reaction. These are more challenging substrates due to their ensuing selectivity issues and low reactivity; however, the scope of this reaction has experienced significant progress^10^ since the early reports by Curtis and Berry.^11^

In spite of the prodigious advances that the C–H silylation methodology has experienced in recent years,^12^ there is still much room for further development. On the lookout for expanding the synthetic reach of this catalytic process, various improvements may be envisaged: (1) the use of more synthetically useful hydrosilane partners is an unresolved problem.^12*a*^ For instance, the preparation of organotrialkoxysilanes by catalytic C–H bond silylation remains widely unexplored.^13–15^ (2) A comprehensive survey of hydro(aryl)silanes would be of interest owing to the potential applicability of these reactions in the synthesis of new materials. Only a limited number of examples has been hitherto reported on this topic.^16,17^ (3) The use of an arene as the limiting reagent is of remarkable importance for the synthetic applicability of this reaction since the arene is frequently the most valuable component in these transformations. Examples of non-directed silylation of arenes under this stoichiometry are scarce and a wider substrate scope, especially regarding unactivated substrates, is highly desirable.^18^

Most of the literature on the catalytic dehydrogenative silylation of C–H bonds has focused on the use of “*in situ*” generated catalysts from commercial metal precursors and ligands. However, somewhat less attention has been paid to the development of well-defined organometallic complexes.^16c,19^ In this regard, the design of catalysts featuring N-heterocyclic carbenes (NHCs) as ancillary ligands has been surprisingly overlooked,^19*b*^ especially when taking into account their success story in homogeneous catalysis.^20^

We report herein on the synthesis and characterization of a well-defined Ir(iii)–NHC complex that behaves as an efficient and versatile catalyst for the dehydrogenative silylation of aromatic C–H bonds for a wide range of hydrosilanes using an arene as the limiting reagent. By means of this catalytic process we have prepared a broad variety of arylsilanes, including examples of the elusive triarylsilanes and trialkoxysilanes. In addition, an experimental and theoretical study on the mechanism that controls this process is discussed here.

In the search for new catalysts for the silylation of C–H bonds, we envisaged an NHC–Ir species featuring labile ligands that would allow for the coordination of substrates and additives^19b,21^ while facilitating the C–H and Si–H activation processes thanks to the unique properties of the NHC ligand.^22^ On these grounds, complex [Ir(H)_2_(IPr)(py)_3_][BF_4_] (IPr = 1,3-bis-(2,6-diisopropylphenyl)imidazol-2-ylidene) (**1**) would be an excellent candidate for this study since the pyridine ligands can be straightforwardly substituted^23^ and the two hydrides may be removed with a hydrogen acceptor or expelled as molecular hydrogen.^24^

## Results and discussion

### Synthesis and characterization of the pre-catalyst

**Scheme 1:**
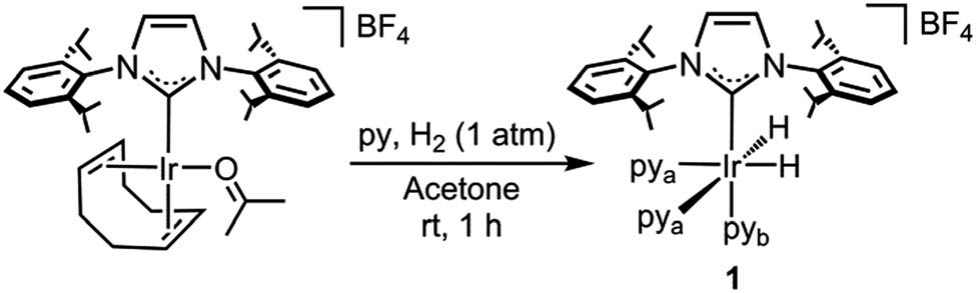
Synthesis of complex **1**.

Complex **1** was prepared in good yield in acetone from [Ir(acetone)(COD)(IPr)][BF_4_] (COD = 1,5-cyclooctadiene) in the presence of excess pyridine (py) under a hydrogen atmosphere (Scheme 1).^25^

**Fig. 1:**
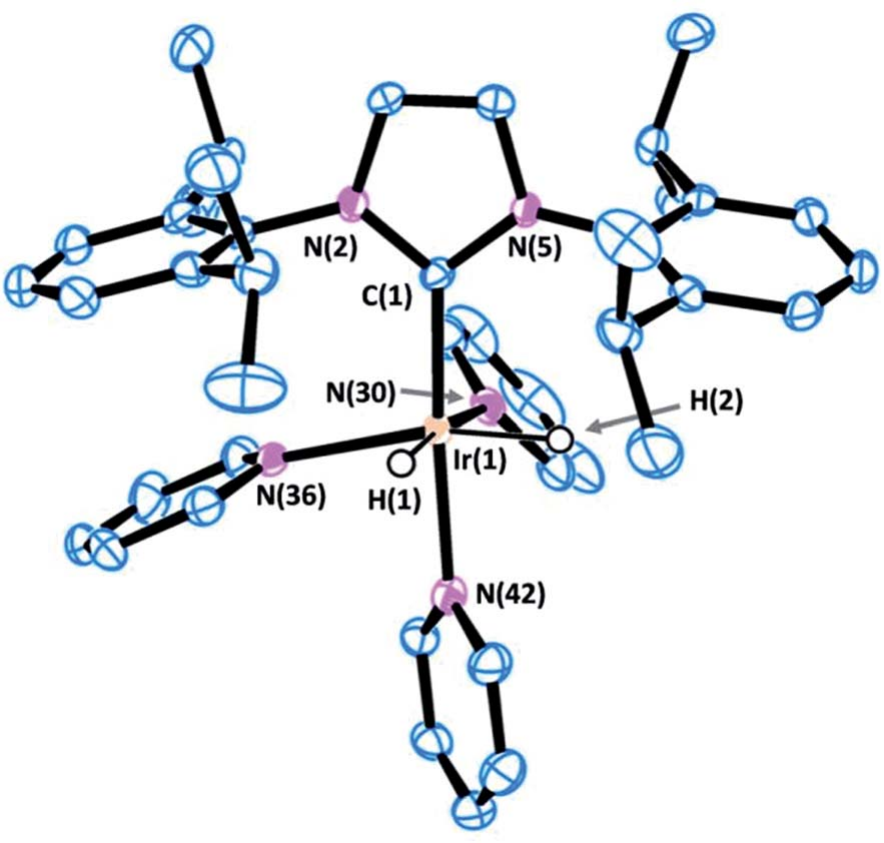
ORTEP view of the cation [Ir(H)_2_(IPr)(py)_3_]^+^ in **1** with a numbering scheme adopted. Most of the hydrogens are omitted for clarity and thermal ellipsoids are at 50% probability. Selected bond lengths (Å) and angles (°): C(1)–N(2) 1.378(4), C(1)–N(5) 1.379(4), C(1)–Ir(1) 1.996(3), N(30)–Ir(1) 2.180(3), N(36)–Ir(1) 2.231(3), N(42)–Ir(1) 2.126(3), N(2)–C(1)–N(5) 102.2(3), C(1)–Ir(1)–N(42) 172.88(13), C(1)–Ir(1)–N(30) 95.39(13), N(42)–Ir(1)–N(30) 89.24(12), C(1)–Ir(1)–N(36) 103.04(13), N(42)–Ir(1)–N(36) 81.93(11), N(30)–Ir(1)–N(36) 94.44(11).

Crystals of complex **1** were obtained by the slow diffusion of diethyl ether into a saturated dichloromethane solution. Its global connectivity pattern was confirmed by single crystal X-ray diffraction (Fig. 1). The molecular structure of **1** shows that the iridium centre adopts a slightly distorted octahedral geometry and the two pyridines *cis* to the IPr ligand are visibly displaced from the equatorial plane, probably due to the steric interference of the bulky wingtip groups of the NHC. Remarkably, the two pyridines in the equatorial plane, *trans* to the hydrides, feature longer Ir–N bond lengths compared to that situated in the *trans* position to the IPr ligand.

The ^1^H NMR spectrum of **1** shows a singlet peak in the high field region at *δ* = –22.48 ppm for both hydride ligands. The IPr ligand presents one singlet peak for the NC*H* protons at *δ* = 7.07 ppm and a septuplet peak for the C*H*Me_2_ protons of the isopropyl groups at *δ* = 2.87 ppm, which suggests a fast rotation of the NHC ligand about the Ir–C bond at room temperature.

Variable temperature NMR analysis shows no line broadening at 193 K, which is consistent with a free energy rotation barrier lower than *ca.* 30 kJ mol^–1^. Two different types of pyridine ligands are observed at *δ* = 8.14 and 7.84 ppm in a 2 : 1 ratio respectively and are positioned in a facial arrangement. The distinct environments observed for the pyridine ligands (labeled “a” and “b” in Scheme 1) are consistent with pyridine dissociation being slow on the NMR time scale.

The most representative resonance in the ^13^C NMR spectrum is that corresponding to the carbene carbon at *δ* = 154.7 ppm. The ^19^F NMR spectrum confirms the cationic nature of **1** with a peak at *δ* = –155.2 ppm that is assigned to the BF_4_^–^ counterion.

### Catalysis

Initial catalytic tests using **1** as a pre-catalyst and 2-(2-thienyl)pyridine as a substrate focused on the optimisation of the reaction conditions and the assessment of whether a hydrogen acceptor would be required. When norbornene was employed as a hydrogen acceptor, a nearly quantitative yield was obtained after 24 h at 110 °C; however, under acceptor-less conditions only a 45% yield was achieved. Other hydrogen acceptors such as cyclohexene or 3,3-dimethyl-1-butene were tested, although somewhat lower yields were obtained.

**Scheme 2:**
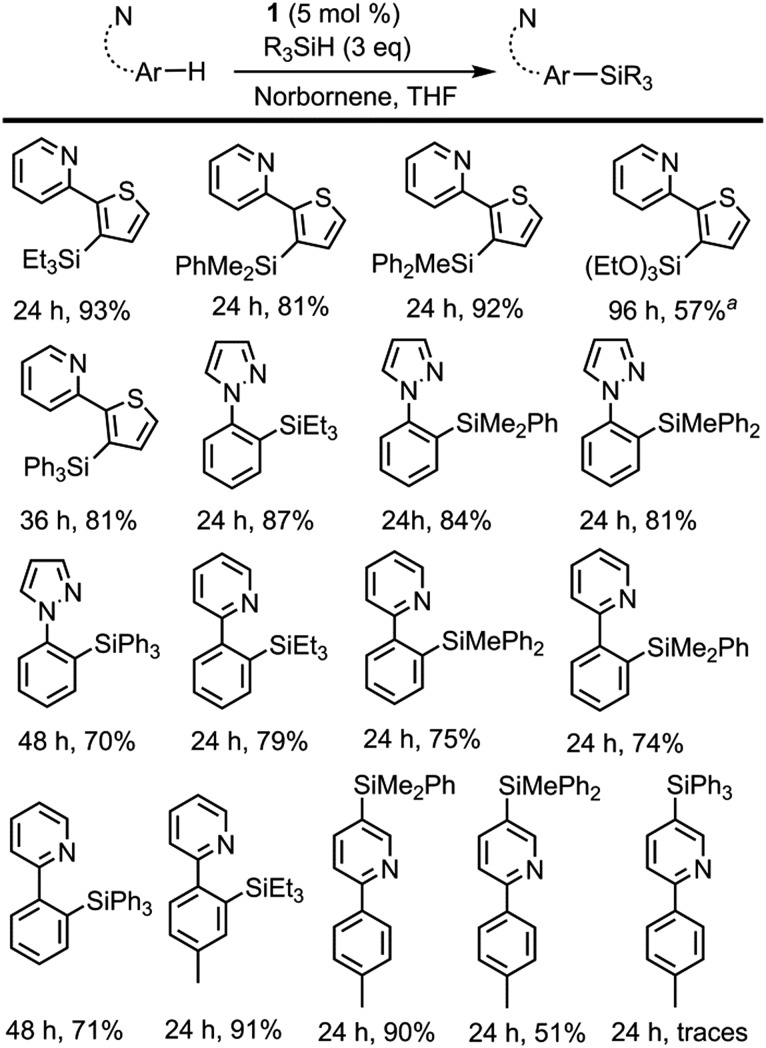
Directed dehydrogenative silylation of aromatic and heteroaromatic rings. Reaction conditions: cat **1** (5 mol%), norbornene (0.40 mmol), arene (0.13 mmol), R_3_SiH (0.40 mmol) in THF (2 mL) at 110 °C. Isolated yields are shown. ^*a*^ Yield determined by ^1^H NMR using THF-d_8_.

**Scheme 3:**
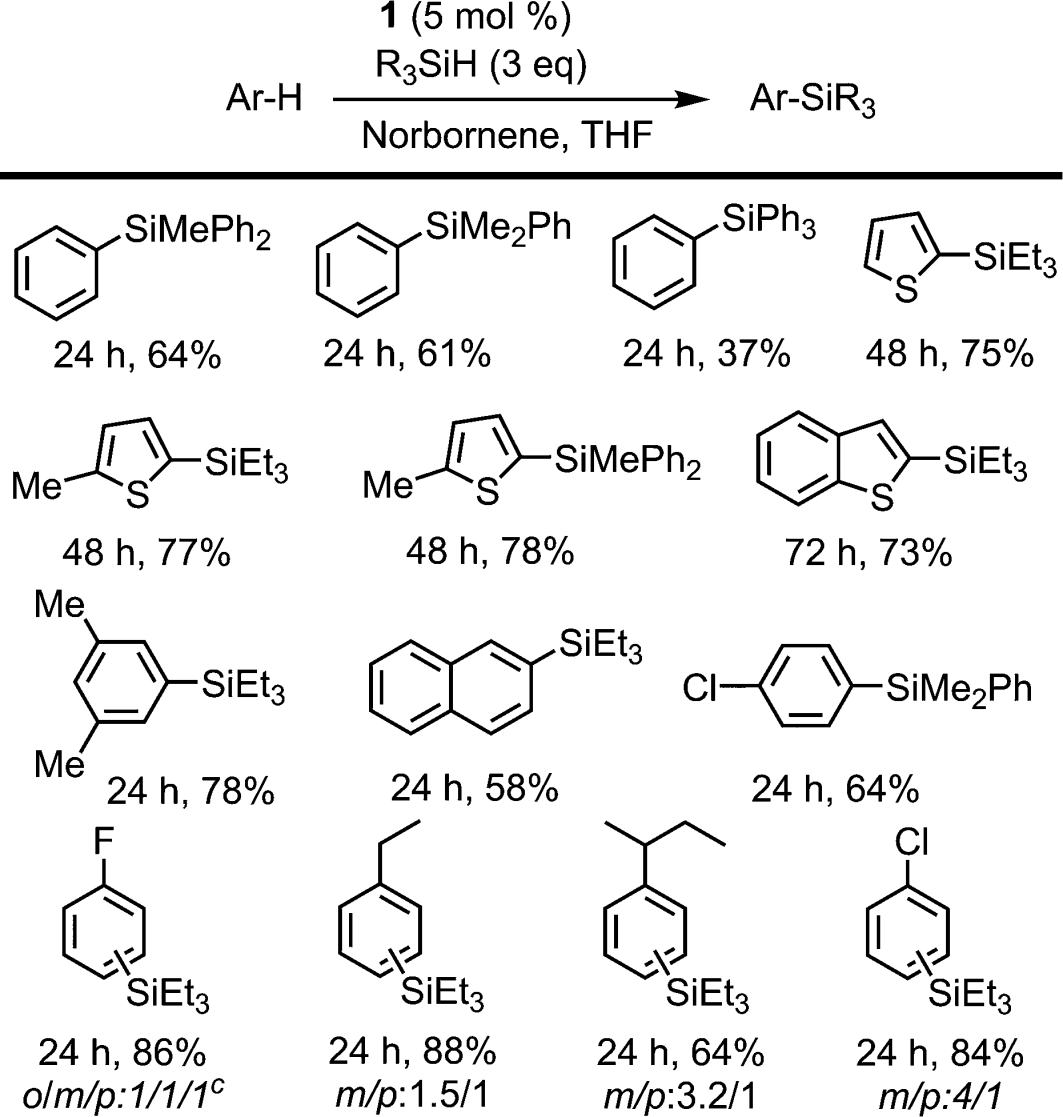
Non-directed dehydrogenative silylation of aromatic and heteroaromatic rings. Reaction conditions: cat **1** (5 mol%), norbornene (0.40 mmol), arene (0.13 mmol), R_3_SiH (0.40 mmol) in THF (2 mL) at 110 °C. Isolated yields are shown. ^*a*^ Disilylated product was identified in 7% yield.

In order to assess the scope of **1** as a pre-catalyst for the silylation of C–H bonds with different hydrosilanes, a variety of aromatic substrates with and without a directing group (Schemes 2 and 3, respectively) were examined. The catalytic reactions were performed in THF at 110 °C in a sealed flask using a 5 mol% catalyst loading and a hydrosilane/arene ratio of 3 : 1.

The use of **1** as a pre-catalyst permits the silylation of 2-(2-thienyl)pyridine with a wide range of hydrosilanes, namely, Et_3_SiH, Ph_2_MeSiH, PhMe_2_SiH, Ph_3_SiH and (EtO)_3_SiH. Remarkably, to the best of our knowledge, these are the only examples of the intermolecular catalytic silylation of aryl C–H bonds that successfully employ triaryl-^17^ or trialkoxy-silanes (excluding the boron catalysed silylation of *N*,*N*-dimethylaniline reported by Hou *et al.* ^10*a*^ and the silatranes reported by Miyaura *et al.* ^13^). However, in the case of the latter, no product was recovered when purification of the crude mixture was attempted by column chromatography. Other substrates featuring nitrogen-containing directing groups, namely, 1-phenylpyrazole, 2-phenylpyridine, and 2-(*p*-tolyl)pyridine, were also successfully converted to the silylated products, except for triethoxysilane (Scheme 2). To our surprise, the silylation of 2-(*p*-tolyl)pyridine showed an unexpected selectivity shift when aromatic silanes were used instead of triethylsilane. In contrast to the previous examples, the directing group, *i.e.* the pyridine moiety, undergoes exclusive silylation of its C5–H bond. This rare selectivity has also been reported recently by Oestreich and co-workers.^26^

The intermolecular non-directed silylation of aromatic and heteroaromatic molecules was also achieved by employing an arene as the limiting reactant (3 equivalents of silane). Among these reactions, the regioselective silylation of naphthalene at the C2-position was also achieved. This is, to the extent of our knowledge, the first example of naphthalene functionalisation by catalytic C–H bond silylation. The silylation of *m*-xylene, thiophene, benzothiophene and 2-methylthiophene was also regioselective, which contrasts to the mixture of regioisomers obtained for fluoro-, chloro-, ethylbenzene and *sec*-butylbenzene using triethylsilane (Scheme 3). To our delight, the selective silylation of chlorobenzene to afford the *para* isomer exclusively was accomplished with PhMe_2_SiH.

The relative reactivity of the different silanes may be estimated from the results presented in Schemes 2 and 3. The least reactive silane is (EtO)_3_SiH since it only works for the most reactive substrate, 2-thienylpyridine, and requires a reaction time of 96 h. The following hydrosilanes in an ascending order of reactivity would be Ph_3_SiH, as longer reaction times are required, then Et_3_SiH, Ph_2_MeSiH and PhMe_2_SiH, which usually show similar reactivity.

A competitive experiment was performed using 1 equivalent of 2-phenylpyridine and 1 equivalent of ethylbenzene with Et_3_SiH under the reaction conditions described in Scheme 2 in order to assess the relative reactivity of directed and non-directed reactions. Exclusive silylation of 2-phenylpyridine was observed, which supports the expected reactivity boost that stems from the presence of a directing group.

**Scheme 4:**
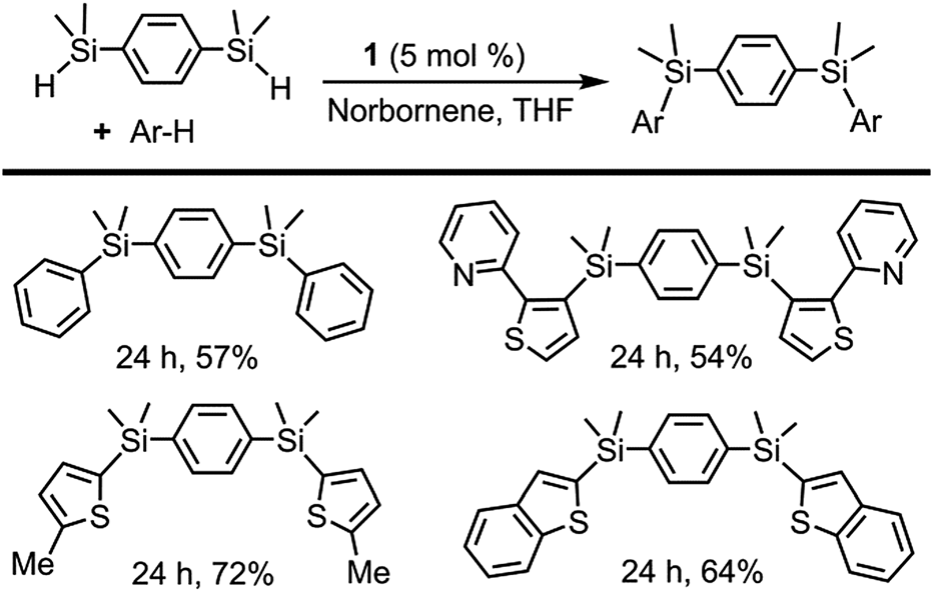
Directed and non-directed dehydrogenative silylation of aromatic and heteroaromatic rings with bis(hydrosilane)s. Reaction conditions: cat **1** (5 mol%), norbornene (0.40 mmol), arene (0.13 mmol), bis(hydrosilane) (0.40 mmol) in THF (2 mL) at 110 °C. Isolated yields are shown.

The selective synthesis of bisarylated bis(silanes) was achieved by the reaction of arenes with the bis(hydrosilane)s, employing **1** as a pre-catalyst (Scheme 4). It is worth mentioning that, in contrast to other examples in the literature, no formation of the monoarylated products^10*b*^ was observed in spite of using excess bis(hydrosilane)s. Due to its unique selectivity, this reaction may find application as a method for the chemoselective synthesis of new conjugated organosilicon materials, which have been hitherto prepared by means of stoichiometric reactions^3b,e,f,27^ or catalytic silylation from aryl halides.^28^

### Mechanistic insights

The mechanistic knowledge of this type of reaction is mainly restricted to the experimental study by Hartwig *et al.* ^21^ on the Rh(i)-catalyzed silylation of arenes, and the theoretical calculations reported by Murata and co-workers on a Ru-catalysed process.^29^ A plausible mechanism for an Ir(iii)-catalysed silylation reaction was proposed by Mashima *et al.*,^19*b*^ however, no kinetic or theoretical support for this postulation has been presented so far.

**Fig. 2:**
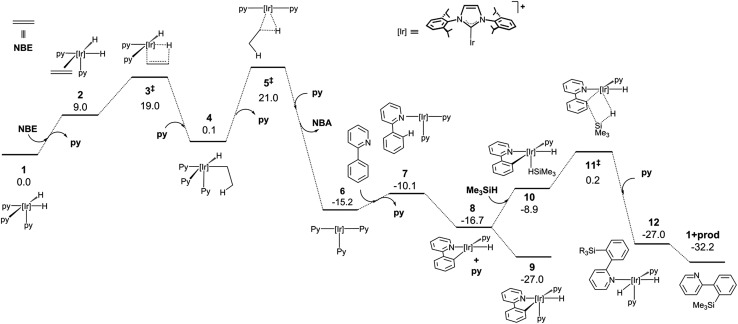
DFT calculated Gibbs free energy profile at 110 °C and a concentration of 1 M (in kcal mol^–1^ and relative to **1** and the isolated molecules) for the Ir-catalysed silylation of 2-phenylpyridine with a hydrogen acceptor.

**Fig. 3:**
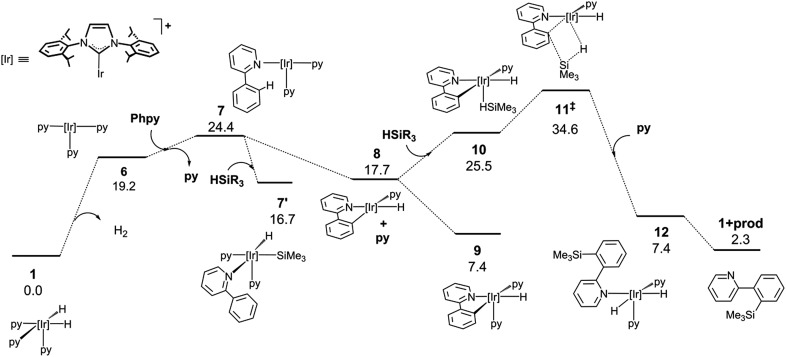
DFT calculated Gibbs free energy profile at 110 °C and a concentration of 1 M (in kcal mol^–1^ and relative to **1** and the isolated molecules) for the Ir-catalysed silylation of 2-phenylpyridine without a hydrogen acceptor.

In order to attain a better understanding of the catalytic cycle that operates in these reactions, a computational study at the DFT level was performed using the B3LYP-D3(PCM)/def2TZVP//B3LYP-D3/def2SVP theoretical level which considered the pre-catalyst **1**, 2-phenylpyridine, HSiMe_3_ as a model for the hydrosilane and NBE (norbornene) as the hydrogen acceptor. The energetic profiles for the directed silylation of 2-phenylpyridine, with and without NBE as the hydrogen acceptor, are shown in Fig. 2 and 3.

The first part of the mechanism involves the dehydrogenation of **1** by the hydrogen acceptor to give a square planar Ir(i) species capable of undergoing cyclometallation with 2-phenylpyridine. The dehydrogenation of **1** with NBE requires the exchange of the pyridine ligand by the olefin followed by the migratory insertion of the double bond into one Ir–H bond *via* **3^‡^** (‡ denotes a transition state) and surmounting an energy barrier of 19.0 kcal mol^–1^. The alkyl intermediate is thus formed and the remaining hydride ligand undergoes reductive elimination through **5^‡^** to give norbornane (NBA) and the Ir(i) square-planar intermediate **6**. The overall dehydrogenation process is exergonic (–15.2 kcal mol^–1^) and features an activation energy of 21.0 kcal mol^–1^. Coordination of 2-phenylpyridine (Phpy) and dissociation of pyridine affords **7**, which subsequently releases a second py ligand and undergoes oxidative addition of the C–H bond adjacent to the pyridine moiety through a barrierless process (ESI†) to yield **8** (–27.0 kcal mol^–1^). Alternatively, the non-directed *o*-, *m*- and *p*- activations of the Ph ring present remarkably higher activation barriers, and a certain amount of *para* or *meta* product would be expected due to the similar energies of their transition states (see ESI†). Hence, N-coordination of Phpy is required to explain the selectivity of the reaction, which is similar to Morokuma’s study.^30^

At this point, coordination of py affords the resting state **9**, which can be isolated by reacting **1** with Phpy (*vide infra*). Coordination of the silane to **8** yields **10**, which undergoes σ-complex assisted metathesis (σ-CAM) between the Ir–C bond of the phenyl moiety and the Si–H bond of the silane *via* transition state **11^‡^**, thus yielding the dihydride intermediate **12**.

An alternative Ir(v) pathway has been discarded since no stationary point on the potential energy surface could be found for the hypothetically conceivable Ir(v) intermediate resulting from the oxidative addition of the silane to the cyclometalated species, which agrees with the mechanism proposed by Mashima and co-workers.^19*b*^ Finally, the substitution of the silylated substrate by a pyridine molecule releases the reaction product and regenerates **1**; this process is neatly exergonic by –23.1 kcal mol^–1^. The effective activation energy for the catalytic cycle is 27.2 kcal mol^–1^ based on the energy span concept,^31^ which is defined in this case by the off-cycle species **9** and transition state **11^‡^**.

Alternatively, the thermic dehydrogenation of **1** to give **6** is also affordable under the reaction conditions but the overall process is thermodynamically much less favourable (Fig. 3). It is worth noting that no transition structures could be found in the reductive elimination of H_2_ from **1** to form **6** plus hydrogen (see ESI†).^32^ The thermodynamics for the acceptor and acceptor-less reaction profiles differ by 34.5 kcal mol^–1^, which is approximately equal to the Δ*H*° for the hydrogenation of norbornene (33.2 kcal mol^–1^).^33^ In addition, the higher energy span found for this process explains the lower reactivity observed for the acceptor-less reaction (27.2 kcal mol^–1^ and 34.6 kcal mol^–1^ for the acceptor and acceptor-less processes, respectively). The possibility of oxidative addition of the silane over the NHC–Ir(i) intermediate **7** was also studied; however, the resulting species (**7′**) is 9.3 kcal mol^–1^ less stable than that resulting from the oxidative addition of the C–H bond (**9**) and only 7.7 kcal mol^–1^ more stable than **7**. Therefore, **7′** may be in equilibrium with **7** under the reaction conditions, thus allowing for the transformation of **7′** into **9**.

### Reactivity studies

#### Reactivity of **1**

**Scheme 5:**
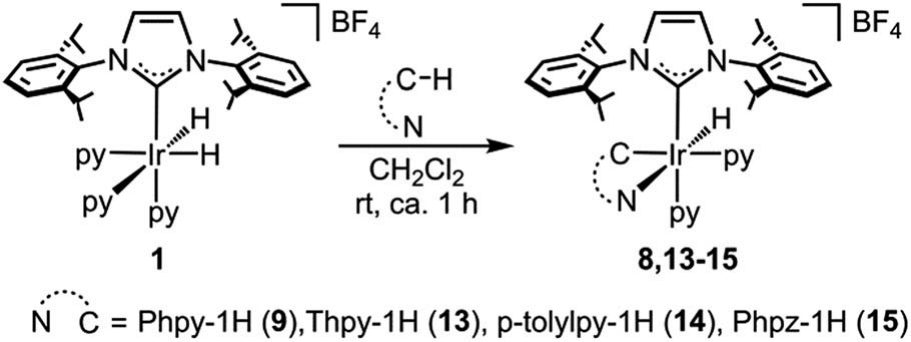
Synthesis of complexes **9** and **13–15**.

In the search for experimental evidence that would support the mechanism proposed above, several stoichiometric experiments were performed. The reaction of complex **1** at room temperature with 1 equivalent of 2-phenylpyridine (Phpy), 2-thienylpyridine (Thpy), 2-(*p*-tolyl)pyridine (*p*-tolylpy) or 1-phenylimidazole (Phpz), with and without norbornene, afforded the corresponding cyclometalated derivatives: complexes **9** and **13–15** (Scheme 5). In this regard, the sluggish formation of complexes **9** and **13–15** in the presence of norbornene at room temperature, and the concomitant generation of norbornane, agrees with the calculated energy barrier (21.0 kcal mol^–1^) for the formation of intermediate **9**.

**Fig. 4:**
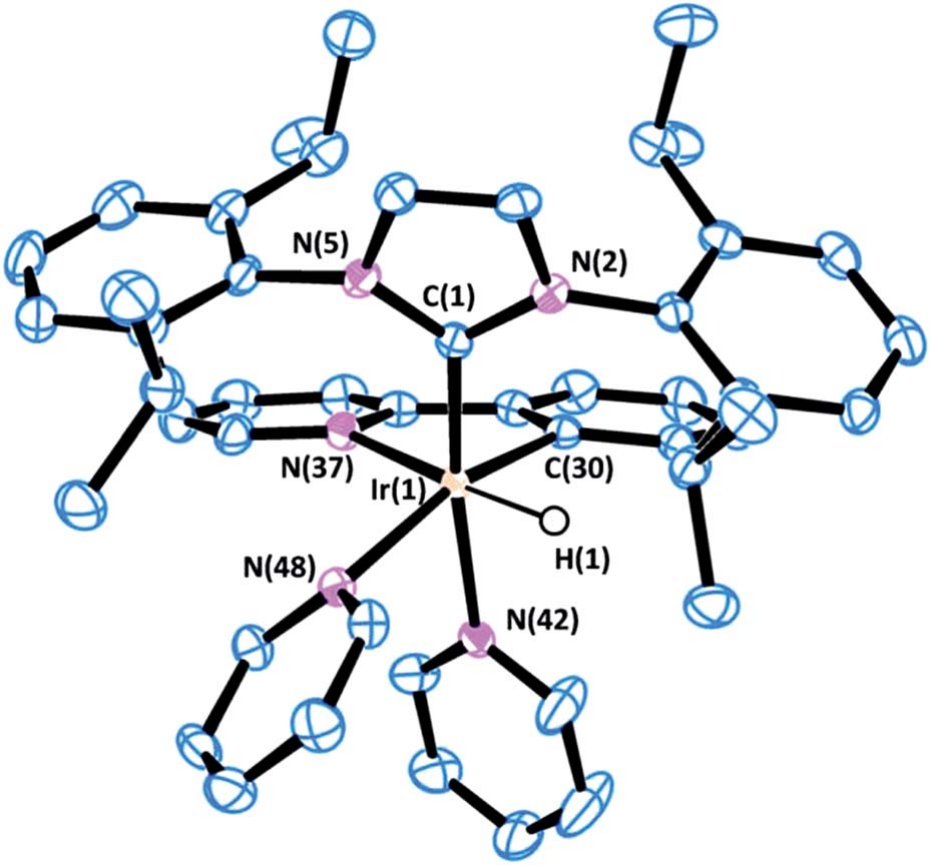
ORTEP view of the cation [Ir(H)(IPr)(Phpy-1H)(py)_2_]^+^ in **9**·CH_2_Cl_2_ with a numbering scheme adopted. Most of the hydrogens are omitted for clarity and thermal ellipsoids are at 50% probability. Selected bond lengths (Å) and angles (°): C(1)–N(2) 1.375(4), C(1)–N(5) 1.376(4), C(1)–Ir(1) 2.019(4), C(30)–Ir(1) 2.017(3), N(37)–Ir(1) 2.186(3), N(42)–Ir(1) 2.138(3), N(48)–Ir(1) 2.194(3), N(2)–C(1)–N(5) 102.5(3), C(30)–Ir(1)–C(1) 99.60(14), C(30)–Ir(1)–N(42) 82.93(13), C(1)–Ir(1)–N(42) 168.91(12), C(30)–Ir(1)–N(37) 79.42(13), C(1)–Ir(1)–N(37) 101.11(13), N(42)–Ir(1)–N(37) 89.96(12), C(30)–Ir(1)–N(48) 164.74(12), C(1)–Ir(1)–N(48) 95.37(12), N(42)–Ir(1)–N(48) 81.81(11), N(37)–Ir(1)–N(48) 100.63(12).

**Fig. 5:**
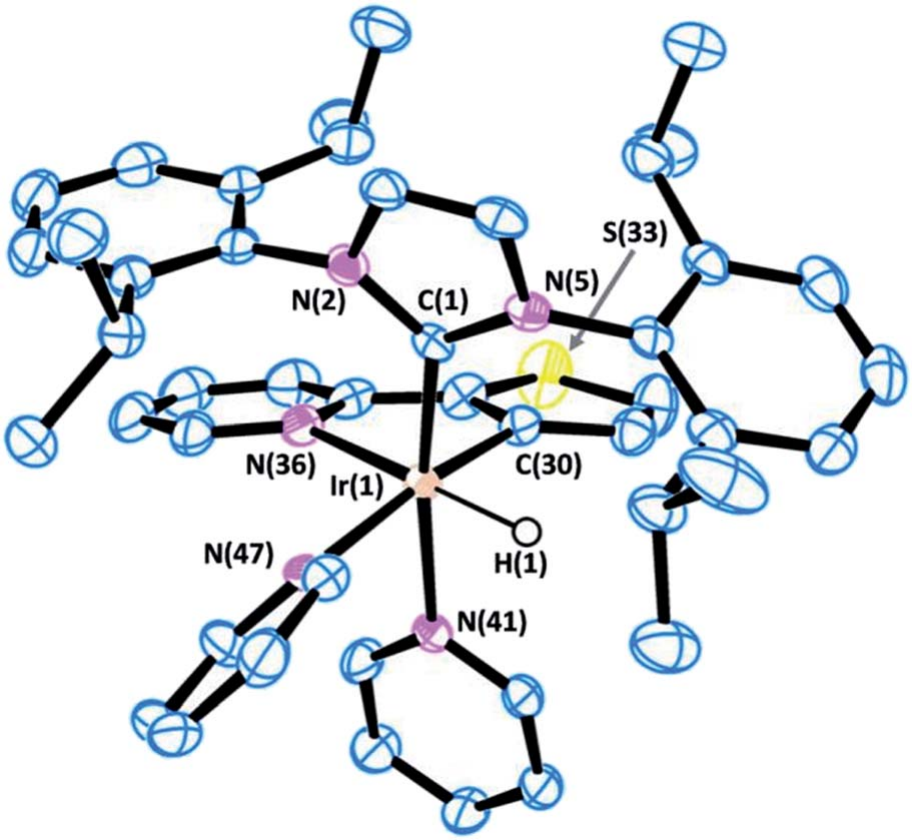
ORTEP view of the cation [Ir(H)(IPr)(Thpy-1H)(py)_2_]^+^ in **13**·1.5·CH_2_Cl_2_ with a numbering scheme adopted. Most of the hydrogens are omitted for clarity and thermal ellipsoids are at 50% probability. Selected bond lengths (Å) and angles (°): C(1)–N(2) 1.375(5), C(1)–N(5) 1.382(5), C(1)–Ir(1) 2.007(4), C(30)–Ir(1) 2.013(4), N(36)–Ir(1) 2.195(4), N(41)–Ir(1) 2.146(4), N(47)–Ir(1) 2.176(4), N(2)–C(1)–N(5) 102.8(3), C(1)–Ir(1)–C(30) 97.21(17), C(1)–Ir(1)–N(41) 170.95(15), C(30)–Ir(1)–N(41) 83.69(16), C(1)–Ir(1)–N(47) 92.20(15), C(30)–Ir(1)–N(47) 170.58(16), N(41)–Ir(1)–N(47) 86.97(14), C(1)–Ir(1)–N(36) 100.97(15), C(30)–Ir(1)–N(36) 79.16(17), N(41)–Ir(1)–N(36) 88.05(13), N(47)–Ir(1)–N(36) 99.36(15).

All of the complexes were isolated as air stable solids and fully characterized by multinuclear NMR spectroscopy. In addition, the molecular structures of complexes **9** and **13** were determined by X-ray diffraction analysis on suitable crystals that were obtained by slow diffusion of diethyl ether into a solution of the corresponding complex in CH_2_Cl_2_ (Fig. 4 and 5).

The most representative resonances in the ^1^H NMR are those in the highfield region, corresponding to the hydrido ligands, which shift upon cyclometallation of the substrate from *δ* = –22.48 ppm in **1** to *δ* = –18.14, –19.30, –18.10 and –19.70 ppm in **9**, **13**, **14** and **15**, respectively.

Besides, APT, HSQC and HMBC NMR experiments support the metallation of the corresponding substrates, thereby confirming the directed C–H activation process.

The X-ray diffraction analysis provides valuable information that may shed light into the selectivity patterns observed in directed silylation. In both compounds the Ir(iii) centre shows a distorted octahedral geometry with the cyclometalated ligand accommodated in the equatorial plane, *cis* to the IPr ligand. The pyridine moiety in the Phpy-1H and Thpy-1H ligands is situated *trans* to the hydride, thus allowing the two py ligands to sit *trans* to the IPr ligand and the metallated carbon atom.

The distorted geometry of **9** and **13** is attributable to the steric repulsion between the cumbersome side arms of IPr and the cyclometalated ligand. This causes the NHC ligand to move away from Phpy-1H (**9**) or Thpy-1H (**13**) (C(1)–Ir(1)–N(42) 168.91(12) and C(1)–Ir(1)–N(37) 101.11(13) for **9** or C(1)–Ir(1)–N(41) 170.95(15) and C(1)–Ir(1)–N(36) 100.97(15) for **13**) and closer to the apical py ligand (C(30)–Ir(1)–N(42) 82.93(13) for **9** or C(30)–Ir(1)–N(41) 83.69(16) for **13**). Moreover, the geometry of the NHC is also affected: (i) the yaw angle (in plane tilting of the NHC) is *ca.* 10° for **9** and **13**; (ii) the methyl (^*i*^Pr) group situated above the py moiety of the cyclometallated ligand shows a dihedral angle C_Ar(C–H)_···C_ipso(C-iPr)_···C_CH(iPr)_···C_Me(iPr)_ of *ca.* 26°, while the other ^*i*^Pr groups feature dihedral angles between 40 and 57°. Both structural parameters are indicative of the steric constraints originating from cyclometallation. On these grounds, an increase in steric hindrance in the system, as is the case for *p*-tolylpy, which would be exacerbated by the use of aromatic silanes, may lead to the de-coordination of the py moiety, reductive elimination and, eventually, oxidative addition of the C–H that affords the least encumbered species. The metallated intermediate that originates from the oxidative addition of the C5–H bond is the one that is situated in the methyl group furthest from the IPr ligand (see the py-silylation products described in Scheme 2).

**Scheme 6:**
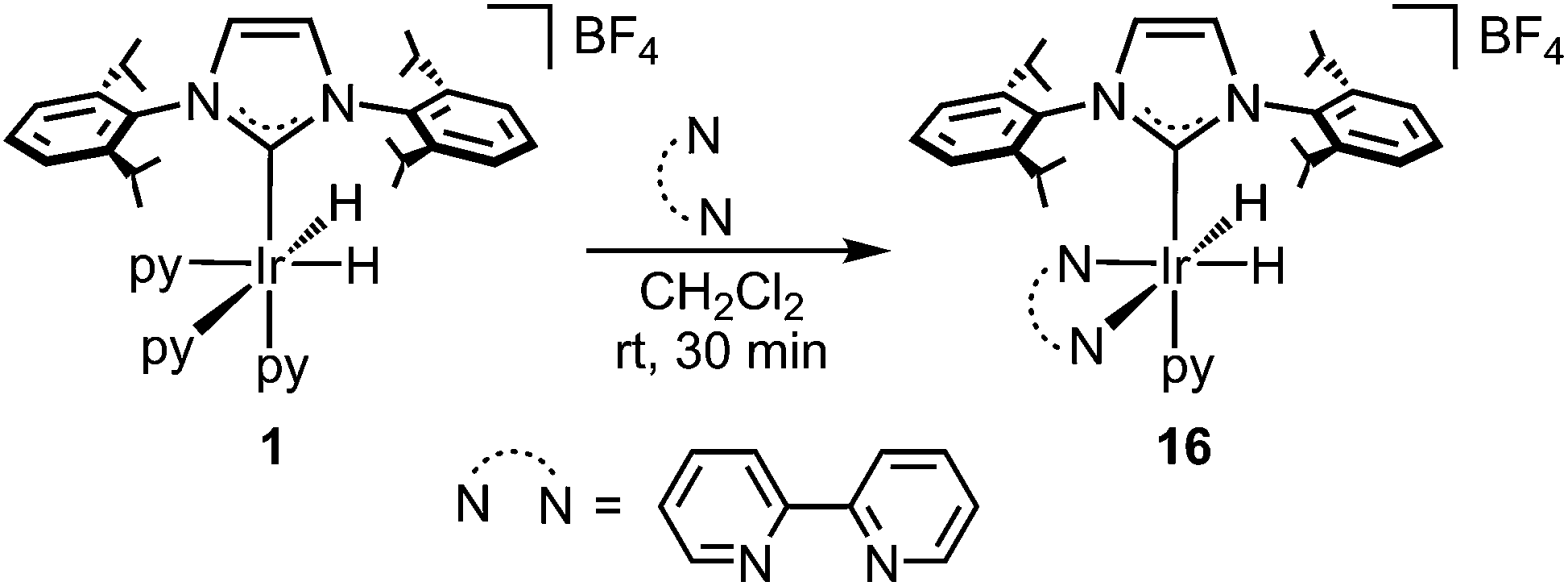
Synthesis of complex **16**.

The reaction of **1** with 1 equivalent of 2,2′-bipyridine (bipy) at room temperature in CH_2_Cl_2_ affords complex [Ir(bipy)(H)_2_(IPr)(py)][BF_4_] (**16**) (Scheme 6), which shows no catalytic activity. This suggests that the presence of the chelating ligand, bipy, thwarts the activation of the arene, which consequently inhibits the catalytic activity of the complex. Moreover, the addition of pyridine (10 equivalents) to the reaction of Phpy with Et_3_SiH, under the conditions described in Scheme 2, resulted in a significant decrease in catalytic activity. In this case, the ^1^H MNR spectrum of the crude mixture shows only a 57% conversion, which contrasts to the example reported in Scheme 2 (without added py) where total conversion was obtained from the crude mixture.

#### Reactivity of the cyclometalated complexes

The addition of 3 equivalents of triethylsilane to a solution of **9** in CH_2_Cl_2_ at room temperature renders the starting complex unaltered, which is consistent with the higher temperatures required for the formation of the organosilane and the calculated energy barrier for this process (27.2 kcal mol^–1^ from **9** to **11^‡^**). Attempts to identify reaction intermediates *in situ* by NMR spectroscopy in 1,1,2,2-tetrachloroethane-d_2_ showed that no reaction takes place up to 100 °C.

With the intention of finding support for the calculated mechanism, cyclometalated complexes **9** and **14** were employed as pre-catalysts under the reaction conditions described in Scheme 2. The reaction of Phpy with Et_3_SiH catalysed by **9** and the reaction of *p*-tolylpy with Ph_2_MeSiH catalysed by **14** gave the silylated products in 81% and 54% yield, respectively (almost identical yields compared to **1**). These experiments, together with the DFT calculations, seem to suggest that **9** may be a resting state that enters the catalytic cycle upon loss of a pyridine ligand.

**Scheme 7:**
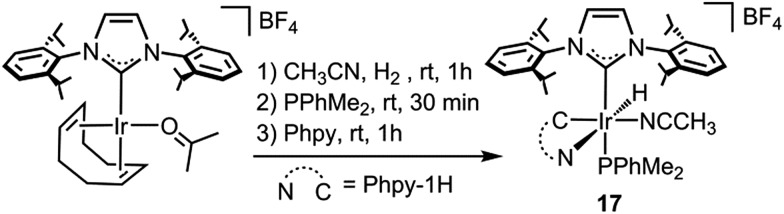
Synthesis of complex **17**.

Moreover, a complex related to **1**, namely [Ir(CH_3_CN)(H)(IPr)(Phpy-1H)(PPhMe_2_)][BF_4_] (**17**), which presents a PPhMe_2_ ligand *trans* to the NHC ligand and an acetonitrile ligand *cis* to the hydride ligand, instead of the apical and equatorial pyridine ligands in **1**, was prepared (Scheme 7). When complex **17** was used as a catalyst for the reaction of Phpy with Et_3_SiH, under the reaction conditions described in Scheme 2, no silylated product was obtained. The fact that **17** is inactive towards the silylation of Phpy agrees with the proposed mechanism, since a labile position *trans* to the IPr ligand is required for the end-on coordination of the silane. Complex **17** features a strongly coordinating ligand *trans* to the NHC ligand which blocks this coordination site, while the availability of an easily accessible position *cis* to the hydride ligand does not seem to play any role in the reaction, which further supports the calculated mechanism. In this regard, the use of the IPr ligand probably facilitates the dissociation of the *trans* positioned py ligand (NHCs feature stronger trans effects than the ligands usually employed for these transformations),^34^ thus generating an available coordination site that may account for the unexpected activity of this system towards less reactive silanes, *e.g.* (EtO)_3_SiH.

In summary, the reactivity shown by complex **1** and the cyclometalated complexes **9** and **13–15** is in accordance with the calculated reaction profile for a variety of reasons: (i) the addition of an arene to **1** gives the corresponding resting states of the catalytic cycle (complexes **9** and **13–15**), which exhibit virtually identical catalytic activity compared to **1**. Furthermore, these species become inactive if the position *trans* to the NHC ligand, where silane coordination should take place, is blocked with a phosphane ligand; (ii) the reaction rates are significantly reduced in the presence of excess py, moreover, when the two coordination sites *trans* to the hydrides in **1** are blocked with bipy, the resulting complex, **16**, is not a competent catalyst for the silylation of Phpy with Et_3_SiH; (iii) complex **9** only reacts with the silane at high temperatures to directly afford the silylated product, which is in agreement with the σ-CAM reaction being the rate limiting step followed by a downslope process toward the organosilane **1**.

Additionally, an experiment employing PhMe_2_SiD and Phpy showed no deuterium incorporation into the silylated product, which also agrees with the proposed mechanism.

## Conclusions

We have prepared a well-defined Ir(iii) complex that acts as an efficient pre-catalyst for the intermolecular silylation of a wide variety of arenes and heteroarenes with and without a directing group. Moreover, in view of expanding the synthetic applicability of this reaction the (hetero)arene was successfully employed in all cases as the limiting reagent. This process is compatible with the use of several hydrosilanes, including examples with Et_3_SiH, Ph_2_MeSiH, PhMe_2_SiH, Ph_3_SiH and (EtO)_3_SiH. It is worth noting that, in certain cases, the presence of aromatic substituents in the hydrosilanes triggers unprecedented selectivity patterns worthy of a more in-depth study in the future. The use of **1** as a pre-catalyst also permits the efficient bisarylation of bis(hydrosilane)s by directed or non-directed silylation of C–H bonds, which may be utilised as a new tool for the synthesis of conjugated organosilicon materials.

The mechanistic studies performed in this work point towards an Ir(iii)/Ir(i) mechanism where the dehydrogenation of the Ir(iii) species **1** generates a very electron-rich NHC–Ir(i) intermediate **6** that allows for the facile activation of the arene C–H bond.

## Experimental

### General considerations

All experiments were carried out under an inert atmosphere using standard Schlenk techniques. The solvents were dried by known procedures and distilled under argon prior to use or obtained oxygen- and water-free from a Solvent Purification System (Innovative Technologies). The starting complex was prepared according to a literature procedure [Ir(COD)(IPr)(acetone)][BF_4_].^25*f*^ All other commercially available starting materials were purchased from Sigma-Aldrich, Merck and J. T. Baker and were used without further purification. H_2_ gas (>99.5%) was obtained from Infra.

^1^H, ^13^C{1H}, ^19^F, ^1^H–^29^Si HMBC, ^1^H–^13^C HMBC, ^1^H–^13^C HSQC and ^1^H–^1^H COSY NMR spectra were recorded either on a Bruker ARX 300 MHz or a Bruker Avance 400 MHz instrument. Chemical shifts (expressed in parts per million) are referenced to residual solvent peaks for ^1^H and ^13^C{1H}, and to an external reference of CFCl_3_ for ^19^F. Coupling constants, *J*, are given in Hz. Spectral assignments were achieved by combination of ^1^H–^1^H COSY, ^13^C APT and 1H–13C HSQC/HMBC experiments. C, H, and N analyses were carried out in a Perkin-Elmer 2400 CHNS/O analyser. GC-MS spectra were recorded on a Hewlett-Packard GC-MS system. Column chromatography was performed using silica gel (70–230 mesh).

### Synthesis and characterization of complexes **9** and **13–17** ‡

#### [Ir(H)_2_(IPr)(py)_3_][BF_4_] (**1**)


‡In the NMR characterisation, the terms py-a and py-b refer to the pyridine ligands *cis* and *trans* to the IPr ligand, respectively.A solution of [Ir(COD)(IPr)(acetone)][BF_4_] (300 mg, 0.36 mmol) in acetone (10 mL) was reacted with pyridine (0.5 mL) and stirred under a hydrogen atmosphere (1 bar) for 1 h. The resulting pale yellow solution was concentrated to *ca.* 0.5 mL, and treated with diethyl ether to afford a white solid. The solid was separated by decantation, washed with diethyl ether and dried *in vacuo*. A CH_2_Cl_2_ solution (0.4 mL) of this solid (12 mg) was layered with diethyl ether (5 mL) and stored in a glove box at room temperature to afford crystals suitable for X-ray diffraction. Yield: 72% (234 mg, 0.25 mmol). ^1^H NMR (300 MHz, CD_2_Cl_2_, 263 K): *δ* 8.14 ppm (d, *J*_H–H_ = 5.0, 4H, H_*o*-py-a_); 7.84 (d, *J*_H–H_ = 5.7, 2H, H_*o*-py-b_); 7.71 (t, *J*_H–H_ = 7.6, 2H, H_*p*-py-a_); 7.66 (t, *J*_H–H_ = 7.5, 1H, H_*p*-py-b_); 7.30 (t, *J*_H–H_ = 7.7, 2H, H_*p*-IPr_); 7.11 (d, *J*_H–H_ = 7.7, 4H, H_*m*-IPr_); 7.09 (dd, *J*_H–H_ = 7.6, 5.0, 4H, H_*m*-py-a_); 7.07 (s, 2H, =CHN); 6.96 (dd, *J*_H–H_ = 7.5, 5.7, 4H, H_*m*-py-b_); 2.87 (sept, *J*_H–H_ = 6.9, 4H, C*H*Me_IPr_); 1.16 and 1.11 (both d, *J*_H–H_ = 6.9, 24H, CH*Me*_IPr_); –22.48 (s, 2H, Ir–H). ^13^C {^1^H}-APT, HSQC and HMBC NMR (75 MHz, CD_2_Cl_2_, 298 K): *δ* 155.3 ppm (s, C_*o*-py-b_); 154.7 (s, Ir–C_IPr_); 153.5 (s, C_*o*-py-a_); 145.6 (s, C_q-IPr_); 138.1 (s, C_q_N); 136.8 (s, C_*p*-py-b_); 136.6 (s, C_*p*-py-a_); 129.9 (s, C_*p*-IPr_); 125.9 (s, C_*m*-py-a_); 125.7 (s, C_*m*-py-b_); 123.8 (s, C_*m*-IPr_); 28.9 (s, *C*HMe_IPr_); 25.9 and 21.6 (both s, CH*Me*_IPr_). ^19^F NMR (400 NMR, CD_2_Cl_2_, 298 K): *δ* –155.2 ppm (s, BF_4_). Anal. calcd. for C_42_H_54_BF_4_IrN_5_ (908.40 + CH_2_Cl_2_): C, 52.02; H, 5.69; N, 7.05%. Found: C, 51.95; H, 5.85; N, 6.85%.

#### [Ir(H)(IPr)(Phpy-1H)(py)_2_][BF_4_] (**9**)

2-Phenylpyridine (19 μL, 0.13 mmol) was added to a solution of **1** (120 mg, 0.13 mmol) in 5 mL of dichloromethane and the resulting solution was stirred for 40 min at room temperature. After this time, the resulting light yellow solution was concentrated to *ca.* 0.5 mL and diethyl ether was added to give a white solid. The solid thus formed was separated by decantation, washed with diethyl ether and dried *in vacuo*. Yield: 63% (82 mg, 0.08 mmol). A CH_2_Cl_2_ solution (0.3 mL) of the solid (10 mg) was layered with diethyl ether (5 mL) and stored in a glove box at room temperature to afford crystals suitable for X-ray diffraction. ^1^H NMR (300 MHz, CD_2_Cl_2_, 298 K): *δ* 8.24 ppm (d, *J*_H–H_ = 5.1, 2H, H_*o*-py-a_); 7.92 (t, *J*_H–H_ = 6.6, 1H, H_*p*-py-a_); 7.91 (d, *J*_H–H_ = 7.9, 1H, H_3-py_); 7.72 (dd, *J*_H–H_ = 7.9, 6.7, 1H, H_4-py_); 7.55 (d, *J*_H–H_ = 7.9, 1H, H_*o*-Ph_); 7.49 (t, *J*_H–H_ = 6.7, 1H, H_*p*-py-b_); 7.47 (t, *J*_H–H_ = 6.6, 2H, H_*p*-IPr_); 7.40 (d, *J*_H–H_ = 5.6, 1H, H_6-py_); 7.37 (d, *J*_H–H_ = 6.0, 2H, H_*o*-py-b_); 7.31 (dd, *J*_H–H_ = 6.6, 5.1, 2H, H_m-py-a_); 7.11 (d, *J*_H–H_ = 6.6, 4H, H_*m*-IPr_); 7.09 (s, 2H, =CHN); 6.78 (dd, *J*_H–H_ = 6.7, 5.6, 1H, H_5-py_); 6.77 (dd, *J*_H–H_ = 6.7, 6.0, 2H, H_*m*-py-b_); 6.75 (dd, *J*_H–H_ = 7.9, 7.7, 1H, H_*m*1-Ph_); 6.46 (dd, *J*_H–H_ = 8.2, 7.7, 1H, H_*p*-Ph_); 5.97 (d, *J*_H–H_ = 8.2, 1H, H_*m*2-Ph_); 2.87 and 2.25 (both sept, *J*_H–H_ = 6.6, 4H, C*H*Me_IPr_); 1.11, 1.05, 1.02, and 0.43 (all d, *J*_H–H_ = 6.6, CH*Me*_IPr_); –18.14 (s, 1H, Ir–H). ^13^C {^1^H}-APT, HSQC and HMBC NMR (75 MHz, CD_2_Cl_2_, 298 K): *δ* 165.1 ppm (s, C_2-py_); 153.5 (s, C_*o*-py-a_); 152.7 (s, C_*o*-py-b_); 150.8 (s, Ir–C_IPr_); 148.2 (s, C_6-py_); 146.6 and 146.4 (both s, C_q-IPr_); 145.3 (s, Ir–C_Ph_); 143.6 (s, C_q-Ph_); 143.4 (s, C_*m*2-Ph_); 138.0 (s, C_*p*-py-a_); 137.1 (s, C_q_N); 137.0 (s, C_*p*-py-b_); 136.8 (s, C_4-py_); 130.2 (s, C_*p*-IPr_); 129.5 (s, C_*p*-Ph_); 126.2 (s, C_*m*-py-a_); 125.7 (s, C_*m*-py-b_); 125.6 (s, =CHN); 124.4 and 123.7 (both s, C_*m*-IPr_); 123.6 (s, C_*o*-Ph_); 123.0 (s, C_5-py_); 121.4 (s, C_*m*1-Ph_); 119.9 (s, C_3-py_); 29.0 and 28.9 (s, *C*HMe_IPr_); 26.9, 26.2, 21.3, and 20.8 (all, s, CH*Me*_IPr_). ^19^F NMR (400 NMR, CD_2_Cl_2_, 298 K): *δ* –152.5 ppm (s, BF_4_). Anal. calcd. for C_48_H_55_IrN_5_BF_4_ (981.41): C, 58.77; H, 5.65; N, 7.14%. Found: C, 58.70; H, 5.66; N, 7.16%.

#### [Ir(H)(IPr)(Thpy-1H)(py)_2_][BF_4_] (**13**)

2-Thienylpyridine (21 mg, 0.13 mmol) was added to a solution of **1** (120 mg, 0.13 mmol) in 5 mL of dichloromethane and the resulting solution was stirred for 40 min at room temperature. After this time, the resulting light yellow solution was concentrated to *ca.* 0.5 mL and diethyl ether was added to give a white solid. The solid thus formed was separated by decantation, washed with diethyl ether and dried *in vacuo*. Yield: 67% (87 mg, 0.09 mmol). ^1^H NMR (400 MHz, CD_2_Cl_2_, 283 K): *δ* 8.27 ppm (d, *J*_H–H_ = 5.3, 2H, H_*o*-py-a_); 7.92 (t, *J*_H–H_ = 7.8, 1H, H_*p*-py-a_); 7.63 (dd, *J*_H–H_ = 7.9, 6.9, 1H, H_4-py_); 7.49 (t, *J*_H–H_ = 7.6, 2H, H_*p*-IPr_); 7.48 (d, *J*_H–H_ = 7.9, 1H, H_3-py_); 7.47 (t, *J*_H–H_ = 7.1, 1H, H_*p*-py-b_); 7.30 (dd, *J*_H–H_ = 7.8, 5.3, 2H, H_*m*-py-a_); 7.29 (d, *J*_H–H_ = 6.2, 1H, H_*o*-py-b_); 7.25 and 7.13 (both d, *J*_H–H_ = 7.6, 4H, H_*m*-IPr_); 7.20 (d, *J*_H–H_ = 5.5, 1H, H_6-py_); 7.11 (s, 2H, =CHN); 6.94 and 5.48 (both d, *J*_H–H_ = 4.7, 2H, H_Th_); 6.78 (dd, *J*_H–H_ = 7.1, 6.2, 2H, H_*m*-py-b_); 6.66 (dd, *J*_H–H_ = 6.9, 5.5, 1H, H_5-py_); 2.86 and 2.28 (both sept, *J*_H–H_ = 6.9, 4H, C*H*Me_IPr_); 1.13, 1.06, 1.05, and 0.55 (all d, *J*_H–H_ = 6.9, 24H, CH*Me*_IPr_); –19.30 (s, 1H, Ir–H). ^13^C {^1^H}-APT, HSQC and HMBC NMR (100 MHz, CD_2_Cl_2_, 298 K): *δ* 160.9 ppm (s, C_2-py_); 154.0 (s, C_*o*-py-a_); 152.4 (s, C_*o*-py-b_); 150.1 (s, Ir–C_IPr_); 148.5 (s, Ir–C_Th_); 148.4 (s, C_6-py_); 146.6 and 146.2 (both s, C_q-IPr_); 140.0 and 128.0 (both s, C_Th_); 138.1 (s, C_*p*-py-a_); 137.3 (s, C_4-py_); 137.1 (s, C_q_N); 137.0 (s, C_*p*-py-b_); 136.8 (s, C_q-Th_); 130.3 (s, C_*p*-IPr_); 126.3 (s, C_*m*-py-a_); 125.5 (s, C_*m*-py-b_); 125.4 and 124.3 (both s, C_*m*-IPr_); 123.8 (s, =CHN); 120.4 (s, C_5-py_); 119.2 (s, C_3-py_); 29.1 and 28.9 (s, *C*HMe_IPr_); 27.0, 26.3, 21.4, and 20.7 (all, s, CH*Me*_IPr_). ^19^F NMR (400 NMR, CD_2_Cl_2_, 298 K): *δ* –153.0 ppm (s, BF_4_). Anal. calcd. for C_46_H_53_IrN_5_BF_4_ (995.40): C, 55.98; H, 5.41; N, 7.10%. Found: C, 55.93; H, 5.46; N 7.10%.

#### [Ir(H)(IPr)(py)_2_(*p*-tolylpy-1H)][BF_4_] (**14**)

2-(*p*-Tolyl)pyridine (22 mg, 0.13 mmol) was added to a solution of **1** (120 mg, 0.13 mmol) in 5 mL of dichloromethane and the resulting solution was stirred for 40 min at room temperature. After this time, the resulting light yellow solution was concentrated to *ca.* 0.5 mL and diethyl ether was added to give a light yellow solid. The solid thus formed was separated by decantation, washed with diethyl ether and dried *in vacuo*. Yield: 67% (88 mg, 0.09 mmol). ^1^H NMR (400 MHz, CD_2_Cl_2_, 298 K): *δ* 8.20 ppm (d, *J*_H–H_ = 5.1, 2H, H_*o*-py-a_); 7.93 (t, *J*_H–H_ = 6.9, 1H, H_*p*-py-a_); 7.90 (d, *J*_H–H_ = 7.6, 1H, H_3-py_); 7.69 (dd, *J*_H–H_ = 7.6, 6.9, 1H, H_4-py_); 7.49 (t, *J*_H–H_ = 6.9, 1H, H_*p*-py-b_); 7.48 (both t, *J*_H–H_ = 7.9, 2H, H_*p*-IPr_); 7.46 (d, *J*_H–H_ = 8.2, 1H, H_*o*-Ph_); 7.36 (br, 2H, H_*o*-py-b_); 7.33 (d, *J*_H–H_ = 5.8, 1H, H_6-py_); 7.30 (dd, *J*_H–H_ = 6.9, 5.1, 2H, H_*m*-py-a_); 7.23 and 7.09 (both d, *J*_H–H_ = 7.9, 4H, H_*m*-IPr_); 7.22 (s, 2H, =CHN); 6.79 (dd, *J*_H–H_ = 6.9, 5.3, 2H, H_*m*-py-b_); 6.71 (dd, *J*_H–H_ = 6.9, 5.8, 1H, H_5-py_); 6.58 (d, *J*_H–H_ = 8.2, 1H, H_*m*1-Ph_); 5.75 (s, 1H, H_*m*2-Ph_); 2.91 and 2.20 (both br, 4H, C*H*Me_IPr_); 1.95 (s, 3H, Me), 1.15, 1.04, 1.03, and 0.37 (all d, *J*_H–H_ = 6.2, 24H, CH*Me*_IPr_); –18.10 (s, Ir–H). ^13^C {^1^H}-APT, HSQC and HMBC NMR (100 MHz, CD_2_Cl_2_, 298 K): *δ* 165.0 ppm (s, C_2-py_); 153.5 (s, C_*o*-py-a_); 152.4 (s, C_*o*-py-b_); 151.2 (s, C_IPr-Ir_); 147.9 (s, C_6-py_); 146.5 and 146.4 (both s, C_q-IPr_); 145.2 (s, Ir–C_Ph_); 143.9 (s, C_*m*2-Ph_); 141.1 (s, C_q-Ph_); 139.3 (s, C_q-Me_); 138.1 (s, C_*p*-py-a_); 137.0 (s, C_*p*-py-b_); 136.9 (s, C_q_N); 136.6 (s, C_4-py_); 130.3 (s, C_*p*-IPr_); 126.1 (s, C_*m*-py-a_); 125.7 (s, C_*m*-py-b_); 125.2 and 124.2 (both s, C_*m*-IPr_); 123.7 (s, =CHN); 123.6 (s, C_*o*-Ph_); 122.8 (s, C_*m*1-Ph_); 122.5 (s, C_5-py_); 119.7 (s, C_3-py_); 29.0 and 28.9 (both s, *C*HMe_IPr_); 26.9, 26.2, 21.3, and 20.3 (all s, CH*Me*_IPr_); 21.4 (s, Me). ^19^F NMR (400 NMR, CD_2_Cl_2_, 298 K): *δ* –152.9 ppm (s, BF_4_). Anal. calcd. for C_49_H_57_IrN_5_BF_4_ (995.43): C, 59.15; H, 5.77; N 7.04%. Found: C, 59.15; H, 5.76; N, 7.10%.

#### [Ir(H)(IPr)(Phpz-1H)(py)_2_][BF_4_] (**15**)

1-Phenylpyrazole (17 μL, 0.13 mmol) was added to a solution of **1** (120 mg, 0.13 mmol) in 5 mL of dichloromethane and the resulting solution was stirred for 1 h at room temperature. After this time, the resulting light yellow solution was concentrated to *ca.* 0.5 mL and diethyl ether was added to give a white solid. The solid thus formed was separated by decantation, washed with diethyl ether and dried *in vacuo*. Yield: 65% (77 mg, 0.09 mmol). ^1^H NMR (400 MHz, CD_2_Cl_2_, 283 K): *δ* 8.30 ppm (d, *J*_H–H_ = 5.1, 2H, H_*o*-py-a_); 7.81 (d, *J*_H–H_ = 2.9, 1H, H_5-pz_); 7.80 (t, *J*_H–H_ = 6.8, 1H, H_*p*-py-a_); 7.44 (t, *J*_H–H_ = 8.0, 2H, H_*p*-IPr_); 7.36 (t, *J*_H–H_ = 6.9, 1H, H_*p*-py-b_); 7.35 (d, *J*_H–H_ = 5.4, 2H, H_*o*-py-b_); 7.21 (dd, *J*_H–H_ = 6.8, 5.1, 2H, H_*m*-py-a_); 7.14 (d, *J*_H–H_ = 1.9, 1H, H_3-pz_); 7.08 (s, 2H, =CHN); 7.07 (d, *J*_H–H_ = 8.0, 4H, H_*m*-IPr_); 6.92 (d, *J*_H–H_ = 7.6, 1H, H_*o*-Ph_); 6.79 (dd, *J*_H–H_ = 7.6, 7.1, 1H, H_*m*1-Ph_); 6.76 (d, *J*_H–H_ = 7.4, 1H, H_*m*2-Ph_); 6.71 (dd, *J*_H–H_ = 6.9, 2H, H_*m*-py-b_); 6.69 (dd, *J*_H–H_ = 7.4, 7.1, 1H, H_*p*-Ph_); 6.45 (dd, *J*_H–H_ = 2.9, 1.9, 1H, H_4-pz_); 2.85 and 2.49 (both sept, *J*_H–H_ = 6.9, 4H, C*H*Me_IPr_); 1.12, 1.09, 1.02, and 0.76 (all d, *J*_H–H_ = 6.9, CH*Me*_IPr_); –19.70 (s, 1H, Ir–H). ^13^C {^1^H}-APT, HSQC and HMBC NMR (100 MHz, CD_2_Cl_2_, 283 K): *δ* 154.6 ppm (s, C_*o*-py-a_); 152.0 (s, C_*o*-py-b_); 149.1 (s, Ir–C_IPr_); 146.4 and 146.0 (both s, C_q-IPr_); 143.9 (s, Ir–C_Ph_); 143.0 (s, C_*m*2-Ph_); 138.7 (s, C_3-pz_); 137.6 (s, C_*p*-py-a_); 136.9 (s, C_*p*-py-b_); 130.0 (s, C_*p*-IPr_); 128.5 (s, C_q-Ph_); 126.4 (s, C_5-pz_); 126.3 (s, C_*m*-py-a_); 126.2 (s, C_*p*-Ph_); 125.3 (s, C_*m*-py-b_); 125.2 (s, =CHN); 123.8 and 123.6 (both s, C_*m*-IPr_); 122.6 (s, C_*m*1-Ph_); 111.1 (s, C_*o*-Ph_); 107.6 (s, C_4-pz_); 29.1 and 28.9 (both s, *C*HMe_IPr_); 26.8, 26.3, 21.2, and 21.2 (all s, CH*Me*_IPr_). Anal. calcd. for C_46_H_55_BF_4_IrN_6_ (971.41 + 0.5·CH_2_Cl_2_): C, 55.11; H, 5.57; N, 8.29%. Found: C, 55.08; H, 5.85; N, 8.61%.

#### [Ir(bipy)(H)_2_(IPr)(py)]BF_4_ (**16**)

2,2′-Bipyridine (16 mg, 0.10 mmol) was added to a solution of **1** (80 mg, 0.10 mmol) in 5 mL of dichloromethane. The resulting solution was stirred for 30 min at room temperature. After this time, the resulting yellow solution was concentrated to *ca.* 0.5 mL and diethyl ether was added to give a yellow solid. The solid was separated by decantation, washed with diethyl ether and dried *in vacuo*. Yield: 79% (69 mg, 0.0764 mmol). ^1^H NMR (400 MHz, CD_2_Cl_2_, 298 K): *δ* 8.67 ppm (d, *J*_H–H_ = 8.1, 2H, H_*m*2-dipy_); 8.56 (d, *J*_H–H_ = 6.1, 2H, H_*o*-py_); 8.42 (dd, *J*_H–H_ = 8.1, 7.8, 2H, H_*p*-dipy_); 8.29 (t, *J*_H–H_ = 7.6, 2H, H_*p*-IPr_); 8.05 (t, *J*_H–H_ = 7.6, 1H, H_*p*-py_); 7.97 (d, *J*_H–H_ = 7.6, 4H, H_*m*-IPr_); 7.80 (d, *J*_H–H_ = 5.1, 2H, H_*o*-dipy_); 7.67 (dd, *J*_H–H_ = 7.8, 5.1, 2H, H_*m*1-dipy_); 7.54 (s, 2H, =CHN); 7.43 (dd, *J*_H–H_ = 7.6, 6.1, 2H, H_*m*-py_); 3.28 (sept, *J*_H–H_ = 6.9, 4H, CHMe_IPr_); 1.69 and 1.50 (both d, *J*_H–H_ = 6.9, 24H, CH*Me*_IPr_); –19.80 (s, 2H, Ir–H). ^13^C {^1^H}-APT, HSQC and HMBC NMR (75 MHz, CD_2_Cl_2_, 298 K): *δ* 156.3 ppm (s, C_q-dipy_); 155.4 (s, C_*o*-py_); 153.0 (s, C_*o*-dipy_); 150.5 (s, Ir–C_IPr_); 147.4 (s, C_q-IPr_); 137.0 (s, C_*p*-dipy_); 136.9 (s, C_q_N); 136.7 (s, C_*p*-py_); 130.1 (s, C_*p*-IPr_); 127.0 (s, C_*m*1-dipy_); 125.1 (s, C_*m*-py_); 124.5 (s, C_*m*-IPr_); 123.5 (s, =CHN); 123.1 (s, C_*m*2-dipy_); 28.8 (s, *C*HMe_IPr_); 25.5 and 21.3 (s, CH*Me*_IPr_). ^19^F NMR (400 NMR, CD_2_Cl_2_, 298 K): *δ* –153.1 ppm (s, BF_4_). Anal. calcd. for C_42_H_51_BF_4_IrN_5_ (905.38): C, 55.75; H, 5.68; N, 7.74%. Found: C, 56.24; H, 5.73; N 7.59%.

#### [Ir(CH_3_CN)(H)(IPr)(Phpy-1H)(PPhMe_2_)][BF_4_] (**17**)

A solution of [Ir(COD)(IPr)(acetone)][BF_4_] (150 mg, 0.18 mmol) in acetonitrile (5 mL) was stirred under a hydrogen atmosphere (1 bar) for 1 h. The solvent was removed under reduced pressure and the remaining pale yellow residue was redissolved in dichloromethane (5 mL). Subsequently, the resulting solution was treated with dimethylphenylphosphine (0.19 mmol, 27 μL) and allowed to react at room temperature for 30 min. Then, 1-phenylpyrazole (25 μL, 0.19 mmol) was added to the solution and stirred at room temperature for 1 h. The resulting pale yellow solution was filtered through Celite, concentrated to *ca.* 0.5 mL and treated with diethyl ether to afford a white solid. The solid was separated by decantation, washed with diethyl ether, and dried *in vacuo*. Yield: 64% (115 mg, 0.11 mmol). ^1^H NMR (400 MHz, CD_2_Cl_2_, 298 K): *δ* 7.86 ppm (d, *J*_H–H_ = 5.4, 1H, H_6-py_); 7.53 (d, *J*_H–H_ = 7.9, 1H, H_3-py_); 7.47 (dd, *J*_H–H_ = 7.9, 6.8, 1H, H_4-py_); 7.46 (d, *J*_H–H_ = 7.6, 1H, H_*o*-Phpy_); 7.45 (t, *J*_H–H_ = 7.7, 2H, H_*p*-IPr_); 7.38 and 7.04 (both d, *J*_H–H_ = 7.7, 4H, H_*m*-IPr_); 7.25 (t, *J*_H–H_ = 7.2, 1H, H_*p*-Ph_); 7.16 (ddd, *J*_H–H_ = 7.8, 7.2, *J*_H–P_ = 2.1, 2H, H_*m*-Ph_); 7.07 (s, 2H, =CHN); 6.92 (dd, *J*_H–H_ = 7.6, 7.1, H_*m*1-phpy_); 6.81 (dd, *J*_H–H_ = 7.4, 7.1, 1H, H_*p*-Phpy_); 6.72 (dd, *J*_H–P_ = 9.7, *J*_H–H_ = 7.8, 2H, H_*o*-Ph_); 6.69 (d, *J*_H–H_ = 7.4, 1H, H_*o*-Phpy_); 6.62 (dd, *J*_H–H_ = 6.8, 5.4, 1H, H_5-py_); 2.55 and 2.41 (both sept, *J*_H–H_ = 6.8, 4H, C*H*Me_IPr_); 2.09 (s, 3H, MeCN); 1.35, 1.14, 1.02, and 0.86 (all d, *J*_H–H_ = 6.8, 24H, CH*Me*_IPr_); 1.23 and 0.57 (both d, *J*_H–P_ = 9.7, 6H, PMe); –18.1 (d, *J*_H–P_ = 17.9, IrH). ^13^C {^1^H}-APT, HSQC and HMBC NMR (75 MHz, CD_2_Cl_2_, 298 K): *δ* 163.5 ppm (s, C_2-py_); 163.3 (d, *J*_H–P_ = 118.7, Ir–C_IPr_); 149.4 (s, C_6-py_); 145.9 and 145.2 (both s, C_q-IPr_); 143.9 (d, *J*_H–P_ = 2.8, C_q-Phpy_); 143.0 (s, C_*m*2-Phpy_); 142.8 (d, *J*_H–P_ = 11.7, Ir–C_Ph_); 137.4 (s, C_q_N); 135.5 (s, C_4-py_); 132.8 (d, *J*_H–P_ = 48.8, C_q-Ph_); 130.3 (s, C_*p*-IPr_); 129.9 (s, C_*p*-Phpy_); 129.1 (d, *J*_H–P_ = 2.5, C_*p*-Ph_); 129.0 (d, *J*_H–P_ = 8.5, C_*m*-Ph_); 128.1 (d, *J*_H-P_ = 9.2, C_*o*-Ph_); 125.2 and 125.1 (both s, =CHN); 123.9 (s, C_*o*-Phpy_); 123.9 and 123.4 (both s, C_*m*-IPr_); 122.4 (s, C_5-py_); 120.7 (s, C_*m*1-Phpy_); 118.7 (s, Me*C*N); 118.6 (s, C_3-py_); 28.5 and 28.4 (both s, *C*HMe_IPr_); 26.8, 25.3, 22.8, and 21.5 (all s, CH*Me*_IPr_); 13.8 and 9.3 (both d, *J*_H–P_ = 41.5, PMe); 3.4 (s, *Me*CN). ^31^P NMR (100 NMR, CD_2_Cl_2_, 298 K): *δ* –28.0 ppm. ^19^F NMR (400 NMR, CD_2_Cl_2_, 298 K): *δ* –152.5 ppm (s, BF_4_). Anal. calcd. for C_48_H_60_BF_4_IrN_4_P (1003.42 + CH_2_Cl_2_): C, 54.10; H, 5.74; N, 5.15%. Found: C, 54.89; H, 6.08; N, 5.62%.

### General procedure for the catalytic silylation of C–H bonds

A sealed flask was charged with complex **1** (5 mol%), THF (2.0 mL), an arene (1 eq., 0.13 mmol), norbornene (3 eq., 0.40 mmol) and a hydrosilane (3 eq., 0.40 mmol). The solution was kept at 110 °C in a thermostatic bath for the reaction time described in the article. The progress of the reactions was monitored by ^1^H NMR spectroscopy and the conversion was determined by integration of the peaks of the starting material with the peaks of the products. At the end of the reaction, the solution was concentrated under reduced pressure to afford the crude residue, which was purified by column chromatography on silica gel using mixtures of hexane/ethyl acetate to isolate the corresponding product.
