# Supplementary material for: A well-defined NHC–Ir(iii) catalyst for the silylation of aromatic C–H bonds: substrate survey and mechanistic insights
Source: Chem Sci. 2017 Apr 5;8(7):4811–22. doi: 10.1039/c6sc04899d (PMC5602144; doi:10.1039/c6sc04899d)
Supplement: Supplementary file 1 [file SC-008-C6SC04899D-s001.pdf]

Electronic Supplementary Material (ESI) for Chemical Science.
This journal is © The Royal Society of Chemistry 2017

Supporting Information for

A Well-Defined NHC-Ir(III) Catalyst for the Silylation of Aromatic C–H Bonds: Substrate Survey and Mechanistic Insights

Laura Rubio-Pérez,[a] *Manuel Iglesias,**[a] *Julen Munárriz,*[b] *Victor Polo,*[b] *Vincenzo Passarelli,*[a,c] *Jesús J. Pérez-Torrente,*[a] *and Luis A. Oro**[a,d]

[a] Departamento Química Inorgánica – ISQCH, Universidad de Zaragoza – CSIC, Pedro Cerbuna 12, 50009 Zaragoza (Spain)

[b] Departamento Química Física – Instituto de Biocomputación y Física de Sistemas Complejos (BIFI), Universidad de Zaragoza, Pedro Cerbuna 12, 50009 Zaragoza (Spain)

[c] Centro Universitario de la Defensa, Ctra. Huesca s/n, ES-50090 Zaragoza (Spain)

[d] King Fahd University of Petroleum & Minerals (KFUPM), Dhahran 31261 (Saudi Arabia).

Table of Contents:

- **Characterisation of organosilanes** **2**

- **NMR spectra of organometallic compounds** **17**

- **NMR spectra of organic compounds** **39**

- **Solid state structure determination** **119**

- **Computational Methods** **120**

- **References** **151**

Characterisation of organosilanes

2-(3-(triethylsilyl)thiophen-2-yl)pyridine

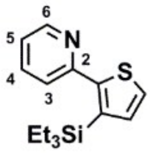

Prepared according to the general procedure from 2-(2-thienyl)pyridine (21 mg, 0.13 mmol) and triethylsilane (63 µL, 0.40 mmol) at 110 ºC for 24 h, to provide the title compound as light yellow oil, which was purified by column chromatography using 100% hexane as eluent. Isolated yield: 93%. ^{1}H NMR (400 MHz, $CDCl_3$, 298 K): δ 8.62 (dq, $J_{H\text{-}H}$ = 4.9, 1.0, 1H, $H_{6\text{-}py}$), 7.69 (ddd, $J_{H\text{-}H}$ = 7.9, 7.5, 1.2, 1H, $H_{4\text{-}py}$), 7.60 (dt, $J_{H\text{-}H}$ = 7.9, 1.0, 1H, $H_{3\text{-}py}$), 7.40 and 7.22 (both d, $J_{H\text{-}H}$ = 5.0, 2H, H_{th}), 7.19 (ddd, $J_{H\text{-}H}$ = 7.5, 4.9, 1.2, 1H, $H_{5\text{-}py}$), 0.91 (t, $J_{H\text{-}H}$ = 7.3, 9H, $SiCH_2CH_3$), 0.84 (q, $J_{H\text{-}H}$ = 7.3, 6H, $SiCH_2CH_3$). ^{13}C {^{1}H}-APT NMR plus HSQC and HMBC (100 MHz, $CDCl_3$, 298 K): δ 154.2 (s, $C_{2\text{-}py}$), 150.2 (s, $C_{q\text{-}th}$), 148.8 (s, $C_{6\text{-}py}$), 137.1 (s, $C_{q\text{-}Si}$), 136.2 (s, $C_{4\text{-}py}$), 135.5 and 125.4 (both s, C_{th}), 122.2 (s, $C_{3\text{-}py}$), 121.9 (s, $C_{5\text{-}py}$), 7.7 (s, $SiCH_2CH_3$), 4.7 (s, $SiCH_2CH_3$). ^{29}Si {^{1}H} NMR (79 MHz, $CDCl_3$, 298 K): δ –0.69. GC-MS: 274 [M^+ - 1], 260 [M^+ - Me], 246 [M^+ - Et], 230, 218, 203, 188, 176, 162, 153, 144, 128, 118, 109, 95, 78.

2-(3-(dimethyl(phenyl)silyl)thiophen-2-yl)pyridine

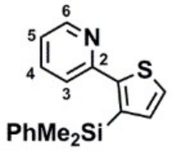

Prepared according to the general procedure from 2-(2-thienyl)pyridine (21 mg, 0.13 mmol) and dimethylphenylsilane (61 µL, 0.40 mmol) at 110 ºC for 24 h, to provide the title compound as light yellow oil, which was purified by column chromatography using a mixture of hexane/ethyl acetate (90/10). Isolated yield: 81%. ^{1}H NMR (300 MHz, $CDCl_3$, 298 K): δ 8.48 (dq, $J_{H\text{-}H}$ = 4.9, 1.0, 1H, $H_{6\text{-}py}$), 7.6-7.3 (m, 5H, $H_{Ph\text{-}Si}$), 7.56 (ddd, $J_{H\text{-}H}$ = 8.2, 7.4, 1.0, 1H, $H_{4\text{-}py}$), 7.50 (dt, $J_{H\text{-}H}$ = 8.2, 1.0, 1H, $H_{3\text{-}py}$), 7.38 and 7.14 (both d, $J_{H\text{-}H}$ = 5.0, 2H, H_{th}), 7.10 (ddd, $J_{H\text{-}H}$ = 7.4, 4.9, 1.2, 1H, $H_{5\text{-}py}$), 0.56 (s, 6H, $SiMe_2Ph$). ^{13}C {^{1}H}-APT NMR plus HSQC and HMBC (100 MHz, $CDCl_3$, 298 K): δ 153.6 (s, $C_{2\text{-}py}$), 150.6 (s, $C_{q\text{-}th}$), 148.4 (s, $C_{6\text{-}py}$), 137.7 (s, $C_{q\text{-}Si}$), 136.1 and 125.4 (both s, C_{th}), 136.0 (s, $C_{4\text{-}py}$), 133.8, 127.9, and 127.6 (all s, $C_{Ph\text{-}Si}$), 121.8 (s, $C_{3\text{-}py}$), 121.6 (s, $C_{5\text{-}py}$), –0.6 (s, $SiMe_2Ph$). ^{29}Si {^{1}H} NMR (79 MHz, $CDCl_3$, 298 K): δ –13.20. GC-MS: 294 [M^+ - 1], 280 [M^+ - Me], 264, 246, 236, 218, 202, 188, 176, 162, 144, 132, 119, 105, 91.

2-(3-(methyldiphenylsilyl)thiophen-2-yl)pyridine

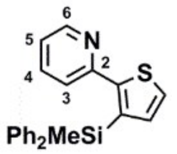

Prepared according to the general procedure from 2-(2-thienyl)pyridine (21 mg, 0.13 mmol) and diphenylmethylsilane (79 µL, 0.40 mmol) at 110 ºC for 24 h, to provide the title compound as colorless oil, which was purified by column chromatography using a mixture of hexane/ethyl acetate (85/15). Isolated yield: 92%. ^{1}H NMR (400 MHz, $CDCl_3$, 298 K): δ 8.23 (dq, $J_{H\text{-}H}$ = 4.8, 1.1, 1H, $H_{6\text{-}py}$), 7.52 (d, $J_{H\text{-}H}$ = 7.7, 4H, $H_{o\text{-}PhSi}$), 7.46 (ddd, $J_{H\text{-}H}$ = 7.8, 5.8, 1.8, 1H, $H_{4\text{-}py}$), 7.45 (dd, $J_{H\text{-}H}$ = 7.8, 1.1, 1H, $H_{3\text{-}py}$), 7.35 (d, $J_{H\text{-}H}$ = 5.0, 1H, $H_{th\text{-}S}$), 7.33 (t, $J_{H\text{-}H}$ = 7.6, 3H, $H_{p\text{-}PhSi}$), 7.31 (dd, $J_{H\text{-}H}$ = 7.7, 7.6, 4H, $H_{m\text{-}PhSi}$), 6.98 (ddd, $J_{H\text{-}H}$ = 5.8, 4.8, 1.8, 1H, $H_{5\text{-}py}$), 6.92 (d, $J_{H\text{-}H}$ = 5.0, 1H, H_{th}), 0.85 (s, 3H, SiMe). ^{13}C {^{1}H}-APT NMR plus HSQC and HMBC (100 MHz, $CDCl_3$, 298 K): δ 152.7 (s, $C_{2\text{-}py}$), 151.5 (s, $C_{q\text{-}th}$), 148.3 (s, $C_{6\text{-}py}$), 138.4 (s, $C_{q\text{-}PhSi}$), 137.5 (s, $C_{th\text{-}S}$), 136.1 (s, $C_{4\text{-}py}$), 135.7 (s, $C_{q\text{-}Si}$), 134.7 (s, $C_{o\text{-}PhSi}$), 128.7 (s, $C_{p\text{-}PhSi}$), 127.6 (s, $C_{m\text{-}PhSi}$), 121.7 (s, $C_{5\text{-}py}$), 121.3 (s, $C_{3\text{-}py}$), –1.9 (s, SiMe). ^{29}Si {^{1}H} NMR (79 MHz, $CDCl_3$, 298 K): δ –16.80. GC-MS: 356 [M^{+} - 1], 343, 332, 317, 280, 264, 253, 218, 207, 188, 177, 158, 147, 132, 120,105, 91, 78.

2-(3-(triphenylsilyl)thiophen-2-yl)pyridine

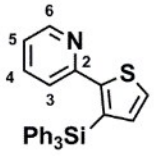

Prepared according to the general procedure from 2-(2-thienyl)pyridine (21 mg, 0.13 mmol) and triphenylsilane (103 mg, 0.40 mmol) at 110 ºC for 24 h, to provide the title compound as white crystals, which was purified by column chromatography using a mixture of hexane/ethyl acetate (80/20). Isolated yield: 81%. ^{1}H NMR (400 MHz, $CDCl_3$, 298 K): δ 7.95 (dq, $J_{H\text{-}H}$ = 4.9, 1.0, 1H, $H_{6\text{-}py}$), 7.57 (dd, $J_{H\text{-}H}$ = 8.0, 1.4, 6H, $H_{o\text{-}PhSi}$), 7.40 (dt, $J_{H\text{-}H}$ = 7.9, 1.0, 1H, $H_{3\text{-}py}$), 7.37 and 6.92 (both d, $J_{H\text{-}H}$ = 5.0, 2H, H_{th}), 7.35 (tt, $J_{H\text{-}H}$ = 7.2, 1.4, 3H, $H_{p\text{-}PhSi}$), 7.28 (ddd, $J_{H\text{-}H}$ = 8.0, 7.2, 1.4, 6H, $H_{m\text{-}PhSi}$), 7.27 (ddd, $J_{H\text{-}H}$ = 7.9, 7.5, 1.0, 1H, $H_{4\text{-}py}$), 6.82 (ddd, $J_{H\text{-}H}$ = 7.5, 4.9, 1.0, 1H, $H_{5\text{-}py}$). ^{13}C {^{1}H}-APT NMR plus HSQC and HMBC (100 MHz, $CDCl_3$, 298 K): δ 152.2 and 133.7 (both s, $C_{q\text{-}th}$), 152.0 (s, $C_{2\text{-}py}$), 147.9 (s, $C_{6\text{-}py}$), 138.1 and 125.6 (both s, C_{th}), 136.4 (s, $C_{q\text{-}PhSi}$), 135.9 (s, $C_{o\text{-}PhSi}$), 135.7 (s, $C_{4\text{-}py}$), 128.8 (s, $C_{p\text{-}PhSi}$), 127.5 (s, $C_{m\text{-}PhSi}$), 121.6 (s, $C_{5\text{-}py}$), 120.9 (s, $C_{3\text{-}py}$). ^{29}Si {^{1}H} NMR (79 MHz, $CDCl_3$, 298 K): δ –20.9. GC-MS: 418 [M^{+} - 1], 399, 386, 372, 356, 342 [M^{+} - Ph], 326, 306, 294, 278, 265 [M^{+} - 2Ph], 252, 38, 219 204, 188 [M^{+} - 3Ph], 160, 154, 140.

2-(3-(triethoxysilyl)thiophen-2-yl)pyridine

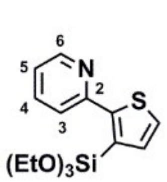

Prepared according to the general procedure from 2-(2-thienyl)pyridine (21 mg, 0.13 mmol) and triethylsilane (63 μL, 0.40 mmol) at 110 ºC for 96 h. Conversion: 57%. This organic compound could not be purified by column chromatography. ^{1}H NMR (400 MHz, $C_2D_2Cl_4$, 298 K): δ 7.84 (d, J_{H-H} = 4.6, 1H, H_{6-py}), 7.30 (dd, J_{H-H} = 7.5, 6.3, 1H, H_{4-py}), 7.07 (d, J_{H-H} = 7.5, 1H, H_{3-py}), 6.83 (d, J_{H-H} = 5.8, 1H, H_{th-S}), 6.70 (dd, J_{H-H} = 6.3, 4.6, 1H, H_{5-py}), 6.43 (d, J_{H-H} = 5.8, 1H, H_{th}), 3.14 (q, J_{H-H} = 7.1, 6H, SiOCH_2CH$_3$), 0.50 (t, J_{H-H} = 7.1, 9H, SiOCH$_2$CH_3). ^{13}C {^{1}H}-APT NMR plus HSQC and HMBC (100 MHz, $CDCl_3$, 298 K): δ 154.7 (s, C_{2-py}), 145.6 (s, C_{q-th}), 144.2 (s, C_{6-py}), 134.4 (s, C_{4-py}), 131.3 (s, C_{th-S}), 129.1 (s, C_{q-Si}), 128.4 (s, C_{th}), 122.1 (s, C_{5-py}), 120.6 (s, C_{3-py}), 59.3 (s, SiOCH$_2$CH$_3$), 16.9 (s, SiOCH$_2$$CH_3$). ^{29}Si {^{1}H} NMR (79 MHz, $CDCl_3$, 298 K): δ –83.65. GC-MS: 323 [M^+], 308 [M^+ - Me], 278 [M^+ - OEt], 262, 250, 233, 222, 213, 204, 189, 177, 161, 148, 139, 128, 111, 100, 89.

2-(2-(triethylsilyl)phenyl)pyridine

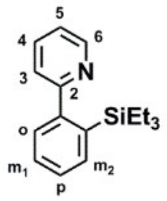

Prepared according to the general procedure from 2-phenylpyridine (19 μL, 0.13 mmol) and triethylsilane (63 μL, 0.40 mmol) at 110 ºC for 24 h, to provide the title compound as colorless oil, which was purified by column chromatography using a mixture of hexane/ethyl acetate (90/10). Isolated yield: 79%. ^{1}H NMR (400 MHz, $CDCl_3$, 298 K): δ 8.66 (dq, J_{H-H} = 4.9, 1.0, 1H, H_{6-py}), 7.75 (ddd, J_{H-H} = 7.9, 7.1, 1.2, 1H, H_{4-py}), 7.67 (d, J_{H-H} = 7.3, 1H, $H_{m2-Phpy}$), 7.47 (dt, J_{H-H} = 7.9, 1.0, 1H, H_{3-py}), 7.44 (dd, J_{H-H} = 7.1, 7.1, 1H, $H_{m1-Phpy}$), 7.43 (d, J_{H-H} = 7.1, 1H, H_{o-Phpy}), 7.41 (dd, J_{H-H} = 7.3, 7.1, 1H, H_{p-Phpy}), 7.28 (ddd, J_{H-H} = 7.1, 4.9, 1.2, 1H, H_{5-py}), 0.86 (t, J_{H-H} = 8.1, 9H, SiCH$_2$CH_3), 0.58 (q, J_{H-H} = 8.1, 6H, SiCH_2CH$_3$). ^{13}C {^{1}H}-APT NMR plus HSQC and HMBC (100 MHz, $CDCl_3$, 298 K): δ 161.8 (s, C_{2-py}), 147.9 (s, C_{q-Phpy}), 135.9 (s, C_{q-Si}), 136.4 (s, $C_{m2-Phpy}$), 136.1 (s, C_{4-py}), 129.1 (s, C_{o-Phpy}), 128.5 (s, $C_{m1-Phpy}$), 127.1 (s, C_{p-Phpy}), 123.2 (s, C_{3-py}), 121.9 (s, C_{5-py}), 7.7 (s, SiCH$_2$$CH_3$), 4.5 (s, Si$CH_2CH_3$). ^{29}Si {^{1}H} NMR (79 MHz, $CDCl_3$, 298 K): δ 3.31. GC-MS: 268 [M^+ - 1], 254 [M^+ - Me], 240 [M^+ - Et], 224, 212, 197, 182, 154, 139, 129, 115, 106.

2-(2-(dimethylphenylsilyl)phenyl)pyridine

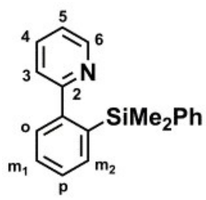

Prepared according to the general procedure from 2-phenylpyridine (19 μL, 0.13 mmol) and dimethylphenylsilane (61 μL, 0.40 mmol) at 110 ºC for 24 h, to provide the title compound as light yellow oil, which was purified by column chromatography using a mixture of hexane/ethyl acetate

(90/10). Isolated yield: 74%. ^{1}H NMR (300 MHz, CDCl$_3$, 298 K): δ 8.50 (d, J_{H-H} = 4.5, 1H, H_{6-py}), 7.67 (d, J_{H-H} = 7.2, 1H, H_{o-Phpy}), 7.58 (td, J = 7.7, 1.7 Hz, 1H, H_{p-Phpy}), 7.62-7.40 (m, 5H, H_{m-Phpy} + H_{4-py} + H_{3-py}), 7.41-733 (m, 2H, H_{m-PhSi}), 7.33-7.23 (m, 3H, H_{o-PhSi} + H_{p-PhSi}), 7.15 (ddd, J_{H-H} = 7.4, 4.9, 0.9, 1H, H_{5-py}), 0.39 (s, 6H, SiMe). ^{13}C {^{1}H}-APT NMR plus HSQC and HMBC (100 MHz, CDCl$_3$, 298 K): δ 160.5 (s, C_{2-py}), 148.3 (s, C_{6-py}), 147.1 (s, C_{q-Phpy}), 140.9 (s, C_{q-Ph}), 137.3 (s, C_{q-PhSi}), 136.8 (s, C_{o-Phpy}), 136.1 (s, C_{4-py}), 133.6 (s, C_{m-PhSi}), 129.1 (s, $C_{m1-Phpy}$), 128.5 (s, C_{p-PhSi}), 128.2 (s, C_{m-Phpy}), 127.7 (s, C_{p-Phpy}), 127.5 (s, C_{3-py}), 127.4 (s, C_{o-PhSi}), 122.7 (s, C_{m-PhSi}), 121.8 (s, C_{5-py}), –0.32 (s, SiMe). ^{29}Si {^{1}H} NMR (79 MHz, CDCl$_3$, 298 K): δ –8.90. GC-MS: 288 [M$^+$ - 1], 274 [M$^+$ - Me], 261, 233, 213, 197, 180, 169, 154, 136, 104.

2-(2-(methyldiphenylsilyl)phenyl)pyridine

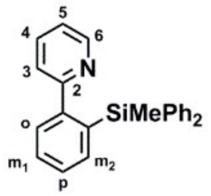

Prepared according to the general procedure from 2-phenylpyridine (19 μL, 0.13 mmol) and diphenylmethylsilane (79 μL, 0.40 mmol) at 110 ºC for 24 h, to provide the title compound as colorless oil, which was purified by column chromatography using a mixture of hexane/ethyl acetate (80/20). Isolated yield: 75%. ^{1}H NMR (300 MHz, CDCl$_3$, 298 K): δ 8.24 (dq, J_{H-H} = 4.9, 1.1, 1H, H_{6-py}), 7.71 (d, J_{H-H} = 7.9, 1H, H_{o-Phpy}), 7.52 (dd, J_{H-H} = 7.9, 7.4, 1H, $H_{m1-Phpy}$), 7.50 (d, J_{H-H} = 7.7, 1H, $H_{m2-Phpy}$), 7.49 (ddd, J_{H-H} = 7.7, 7.3, 1.3, 1H, H_{4-py}), 7.44 (d, J_{H-H} = 8.0, 4H, H_{o-PhSi}), 7.43 (dt, J_{H-H} = 7.7, 1.1, 1H, H_{3-py}), 7.35 (dd, J_{H-H} = 7.7, 7.4, 1H, H_{p-Phpy}), 7.33 (t, J_{H-H} = 7.3, 2H, H_{p-PhSi}), 7.29 (dd, J_{H-H} = 8.0, 7.3, 4H, H_{m-PhSi}), 7.02 (ddd, J_{H-H} = 7.3, 4.9, 1.3, 1H, H_{5-py}), 0.64 (s, 3H, SiMe). ^{13}C {^{1}H}-APT NMR plus HSQC and HMBC (100 MHz, CDCl$_3$, 298 K): δ 159.1 (s, C_{2-py}), 147.9 (s, C_{6-py}), 146.9 (s, C_{q-Phpy}), 139.6 (s, C_{q-PhSi}), 138.9 (s, $C_{m2-Phpy}$), 136.2 (s, C_{4-py}), 135.4 (s, C_{q-Si}), 134.7 (s, C_{o-PhSi}), 129.5 (s, $C_{m1-Phpy}$), 128.3 (s, C_{p-PhSi}), 128.0 (s, C_{o-Phpy}), 127.7 (s, C_{p-Phpy}), 127.5 (s, C_{m-PhSi}), 121.9 (s, C_{3-py}), 121.8 (s, C_{5-py}), –1.4 (s, SiMe). ^{29}Si {^{1}H} NMR (79 MHz, CDCl$_3$, 298 K): δ –13.60. GC-MS: 350 [M$^+$ - 1], 336 [M$^+$ - Me], 322, 294, 274, 258, 241, 230, 215, 197, 182, 167, 154, 139, 129, 115, 105, 91.

2-(2-(triphenylsilyl)phenyl)pyridine

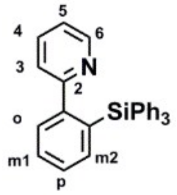

Prepared according to the general procedure from 2-phenylpyridine (19 μL, 0.13 mmol) and triphenylsilane (103 mg, 0.40 mmol) at 110 ºC for 24 h, to provide the title compound as colorless crystals suitable for X-ray, which was purified by column chromatography using a mixture of

hexane/ethyl acetate (75/25). Isolated yield: 71%. ^{1}H NMR (400 MHz, $CDCl_3$, 298 K): δ 7.85 (d, J_{H-H} = 7.9, 1H, H_{o-Phpy}), 7.56 (d, J_{H-H} = 5.2, 1H, H_{6-py}), 7.55 (d, J_{H-H} = 7.9, 1H, H_{3-py}), 7.47 (dd, J_{H-H} = 7.9, 7.5, 1H, $H_{m1-Phpy}$), 7.38 (d, J_{H-H} = 7.9, 6H, H_{o-PhSi}), 7.36 (d, J_{H-H} = 7.6, 1H, $H_{m2-Phpy}$), 7.35 (dd, J_{H-H} = 7.9, 7.4, 1H, H_{4-py}), 7.25 (dd, J_{H-H} = 7.6, 7.5, 1H, H_{p-Phpy}), 7.17 (t, J_{H-H} = 7.5, 1H, H_{p-PhSi}), 7.12 (dd, J_{H-H} = 7.9, 7.5, 6H, H_{m-PhSi}), 6.66 (dd, J_{H-H} = 5.2, 7.4, 1H, H_{5-py}). ^{13}C {^{1}H}-APT NMR plus HSQC and HMBC (100 MHz, $CDCl_3$, 298 K): δ 155.9 (s, C_{2-py}), 146.9 (s, C_{6-py}), 145.5 (s, C_{q-Phpy}), 140.5 (s, $C_{m2-Phpy}$), 139.3 (s, C_{q-PhSi}), 136.2 (s, C_{4-py}), 135.6 (s, C_{o-PhSi}), 133.3 (s, C_{q-Si}), 130.1 (s, $C_{m1-Phpy}$), 128.4 (s, C_{p-Phpy}), 128.0 (s, C_{p-PhSi}), 127.1 (s, C_{m-PhSi}), 126.6 (s, C_{o-Phpy}), 121.9 (s, C_{5-py}), 118.9 (s, C_{3-py}). ^{29}Si {^{1}H} NMR (79 MHz, $CDCl_3$, 298 K): δ -21.45. GC-MS: 412 [M^+ - 1], 389, 352, 336 [M^+ - Ph], 312, 295, 284, 259 [M^+ - 2Ph], 243, 230, 219, 207, 182 [M^+ - 3Ph], 167, 154, 141, 129, 117, 105.

1-(2-(triethylsilyl)phenyl)-1H-pyrazole

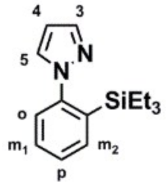

Prepared according to the general procedure from 1-phenylpyrazole (18 μL, 0.13 mmol) and triethylsilane (63 μL, 0.40 mmol) at 110 ºC for 24 h, to provide the title compound as colorless oil, which was purified by column chromatography using 100% hexane. Isolated yield: 87%. ^{1}H NMR (400 MHz, $CDCl_3$, 298 K): δ 7.57 (d, J_{H-H} = 1.8, 1H, H_{3-pz}), 7.51 (d, J_{H-H} = 7.0, 1H, $H_{m2-Phpz}$), 7.49 (d, J_{H-H} = 2.3, 1H, H_{5-pz}), 7.33 (dd, J_{H-H} = 7.3, 6.9, 1H, $H_{m1-Phpz}$), 7.30 (dd, J_{H-H} = 7.3, 7.0, 1H, H_{p-Phpz}), 7.17 (d, J_{H-H} = 6.9, 1H, H_{o-Phpz}), 6.33 (dd, J_{H-H} = 2.3, 1.8, 1H, H_{4-pz}), 0.76 (t, J_{H-H} = 7.9, 9H, $SiCH_2CH_3$, 0.46 (q, J_{H-H} = 7.9, 6H, $SiCH_2CH_3$). ^{13}C {^{1}H}-APT NMR plus HSQC and HMBC (100 MHz, $CDCl_3$, 298 K): δ 146.4 (s, C_{q-Phpz}), 139.8 (s, C_{3-pz}), 136.7 (s, $C_{m2-Phpz}$), 133.8 (s, C_{q-Si}), 130.4 (s, C_{5-pz}), 129.6 (s, $C_{m1-Phpz}$), 127.6 (s, C_{p-Phpz}), 126.1 (s, C_{o-Phpz}), 106.5 (s, C_{4-pz}), 7.58 (s, $SiCH_2CH_3$), 3.47 (s, $SiCH_2CH_3$). ^{29}Si {^{1}H} NMR (79 MHz, $CDCl_3$, 298 K): δ 3.88. GC-MS: 257 [M^+ -1], 243 [M^+ - Me], 229 [M^+ - Et], 213, 201, 186, 171, 159, 144, 132, 125, 117, 105, 95, 86.

1-(2-(dimethylphenylsilyl)phenyl)-1H-pyrazole

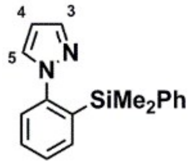

Prepared according to the general procedure from 1-phenylpyrazole (18 μL, 0.13 mmol) and dimethylphenylsilane (61 μL, 0.40 mmol) at 110 ºC for 24 h, to provide the title compound as light yellow oil, which was purified by column chromatography using a mixture of hexane/ethyl acetate (85/15). Isolated yield: 84%. ^{1}H NMR (400 MHz, $CDCl_3$, 298 K): δ 7.89 (d, J_{H-H} = 2.7, 1H, H_{5-pz}), 7.66 (d, J_{H-H} = 1.7, 1H, H_{3-pz}), 7.63 (d, J_{H-H} = 8.6, 2H, H_{o-PhSi}), 7.4-7.2 (m, 4H, H_{Phpz}), 7.38 (dd, J_{H-H} = 8.6, 7.4,

2H, $H_{m\text{-}PhSi}$), 7.23 (t, $J_{H\text{-}H}$ = 7.4, 1H, $H_{p\text{-}PhSi}$), 6.40 (dd, $J_{H\text{-}H}$ = 2.7, 1.7, 1H, $H_{4\text{-}pz}$), 0.22 (s, 6H, SiMe). ^{13}C $\{^1H\}$-APT NMR plus HSQC and HMBC (100 MHz, $CDCl_3$, 298 K): δ 148.3 (s, $C_{q\text{-}Phpz}$), 143.1 (s, $C_{3\text{-}pz}$), 140.9 (s, $C_{q\text{-}PhSi}$), 136.4 (s, $C_{q\text{-}Si}$), 135.8, 130.8, 129.5, and 127.6 (all s, C_{Phpz}), 131.4 (s, $C_{m\text{-}PhSi}$), 129.6 (s, $C_{p\text{-}PhSi}$), 128.7 (s, $C_{5\text{-}pz}$), 121.2 (s, $C_{o\text{-}PhSi}$), 109.6 (s, $C_{4\text{-}pz}$), 0.0 (s, SiMe). ^{29}Si $\{^1H\}$ NMR (79 MHz, $CDCl_3$, 298 K): δ –7.80 GC-MS: 277[M^+ - 1], 263[M^+ - Me], 248[M^+ - 2Me], 235, 220, 201, 185, 171, 156, 144, 134.

1-(2-(methyldiphenylsilyl)phenyl)-1H-pyrazole

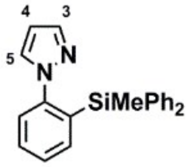

Prepared according to the general procedure from 1-phenylpyrazole (18 μL, 0.13 mmol) and diphenylmethylsilane (79 μL, 0.40 mmol) at 110 ºC for 24 h, to provide the title compound as light yellow oil, which was purified by column chromatography using a mixture of hexane/ethyl acetate (90/10). Isolated yield: 81%. ^1H NMR (400 MHz, $CDCl_3$, 298 K): δ 7.50 (d, $J_{H\text{-}H}$ = 1.8, 1H, $H_{3\text{-}pz}$), 7.5-7.3 (m, 4H, H_{Phpz}), 7.47 (d, $J_{H\text{-}H}$ = 7.9, 4H, $H_{o\text{-}PhSi}$), 7.39 (t, $J_{H\text{-}H}$ = 6.9, 2H, $H_{p\text{-}PhSi}$), 7.34 (dd, $J_{H\text{-}H}$ = 7.9, 6.8, 4H, $H_{m\text{-}PhSi}$), 7.28 (d, $J_{H\text{-}H}$ = 2.5, 1H, $H_{5\text{-}pz}$), 6.18 (dd, $J_{H\text{-}H}$ = 2.5, 1.8, 1H, $H_{4\text{-}pz}$), 0.44 (s, 3H, SiMe). ^{13}C $\{^1H\}$-APT NMR plus HSQC and HMBC (100 MHz, $CDCl_3$, 298 K): δ 146.8 (s, $C_{q\text{-}Phpz}$), 139.8 (s, $C_{3\text{-}pz}$), 138.5, 130.5, 127.5, and 125.7 (all s, C_{Phpz}), 137.0 (s, $C_{q\text{-}PhSi}$), 135.0 (s, $C_{o\text{-}PhSi}$), 132.1 (s, $C_{q\text{-}Si}$), 130.3 (s, $C_{5\text{-}pz}$), 129.2 (s, $C_{p\text{-}PhSi}$), 127.8 (s, $C_{m\text{-}PhSi}$), 106.8 (s, $C_{4\text{-}pz}$), –3.8 (s, SiMe). ^{29}Si $\{^1H\}$ NMR (79 MHz, $CDCl_3$, 298 K): δ –10.7. GC-MS: 339 [M^+ - 1], 325 [M^+ - Me], 312, 296, 284, 263, 247, 234, 220, 206, 195, 186, 171, 165, 154, 144, 131.

1-(2-(triphenylsilyl)phenyl)-1H-pyrazole

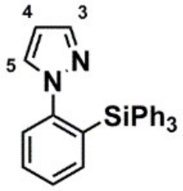

Prepared according to the general procedure from 1-phenylpyrazole (18 μL, 0.13 mmol) and triphenylsilane (103 mg, 0.40 mmol) at 110 ºC for 24 h, to provide the title compound as colorless crystals, which was purified by column chromatography using a mixture of hexane/ethyl acetate (80/20). Isolated yield: 70%. ^1H NMR (400 MHz, $CDCl_3$, 298 K): δ 7.5-7.2 (m, 4H, H_{Phpz}), 7.42 (d, $J_{H\text{-}H}$ = 8.0, 6H, $H_{o\text{-}PhSi}$), 7.32 (d, $J_{H\text{-}H}$ = 2.5, 1H, $H_{5\text{-}pz}$), 7.23 (t, $J_{H\text{-}H}$ = 7.3, 3H, $H_{p\text{-}PhSi}$), 7.21 (dd, $J_{H\text{-}H}$ = 8.0, 7.3, 6H, $H_{m\text{-}PhSi}$), 6.93 (d, $J_{H\text{-}H}$ = 1.7, 1H, $H_{3\text{-}pz}$), 5.76 (dd, $J_{H\text{-}H}$ = 2.5, 1.7, 1H, $H_{4\text{-}pz}$). ^{13}C $\{^1H\}$-APT NMR plus HSQC and HMBC (100 MHz, $CDCl_3$, 298 K): 146.7 ($C_{q\text{-}PhPz}$), 139.9 (s, $C_{3\text{-}pz}$), 139.8, 130.9, 127.0, and 124.0 (C_{Phpz}), 135.9 (s, $C_{o\text{-}PhSi}$), 135.6 (s, $C_{q\text{-}PhSi}$), 129.0 (s, $C_{p\text{-}PhSi}$), 128.9 (s, $C_{5\text{-}pz}$), 128.6 (s, $C_{q\text{-}Si}$), 127.5 (s, $C_{m\text{-}}$

$_{PhSi}$), 106.9 (s, C_{4-pz}). ^{29}Si {^{1}H} NMR (79 MHz, $CDCl_3$, 298 K): δ –15.78. GC-MS: 402[M^+ -1], 325[M^+ - Ph], 296, 284, 270, 259, 248, 221, 206, 193, 181, 171, 165, 154, 143, 129, 117, 105.

2-(4-methyl-2-(triethylsilyl)phenyl)pyridine

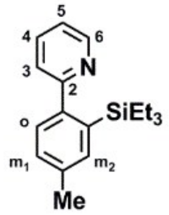

Prepared according to the general procedure from 2-(*p*-tolyl)pyridine (23 µL, 0.13 mmol) and triethylsilane (63 µL, 0.40 mmol) at 110 ºC for 24 h, to provide the title compound as light yellow oil, which was purified by column chromatography using a mixture of hexane/ethyl acetate (95/5). Isolated yield: 91%. ^{1}H NMR (300 MHz, $CDCl_3$, 298 K): δ 8.63 (dq, J_{H-H} = 4.9, 1.0, 1H, H_{6-py}), 7.72 (ddd, J_{H-H} = 7.8, 6.0, 1.8, 1H, H_{4-py}), 7.46 (s, 1H, $H_{m2-Phpy}$), 7.44 (dt, J_{H-H} = 7.8, 1.0, 1H, H_{3-py}), 7.35 (d, J_{H-H} = 7.6, 1H, H_{o-Phpy}), 7.25 (ddd, J_{H-H} = 6.0, 4.9, 1.8, 1H, H_{5-py}), 7.24 (d, J_{H-H} = 7.6, 1H, $H_{m1-Phpy}$), 2.43 (s, 3H, Me), 0.86 (t, J_{H-H} = 8.2, 3H, $SiCH_2CH_3$), 0.58 (q, J_{H-H} = 8.2, 4H, $SiCH_2CH_3$). ^{13}C {^{1}H}-APT NMR plus HSQC and HMBC (100 MHz, $CDCl_3$, 298 K): δ 161.9 (s, C_{2-py}), 148.4 (s, C_{6-py}), 145.3 (s, C_{q-Phpy}), 137.2 (s, $C_{m2-Phpy}$), 136.5 (s, C_{q-Me}), 135.9 (s, C_{4-py}), 135.8 (s, C_{q-Si}), 129.2 (s, $C_{m1-Phpy}$), 128.9 (s, C_{o-Phpy}), 123.2 (s, C_{3-py}), 121.7 (s, C_{5-py}), 21.4 (s, Me), 7.6 (s, $SiCH_2CH_3$), 4.6 (s, $SiCH_2CH_3$). ^{29}Si {^{1}H} NMR (59 MHz, $CDCl_3$, 298 K): δ 2.92. GC-MS: 283 [M^+ - 1], 268 [M^+ - Me], 254 [M^+ - Et], 238, 226, 211, 196, 182, 167, 164, 143, 135, 127, 113, 98.

5-(dimethyl(phenyl)silyl)-2-(p-tolyl)pyridine

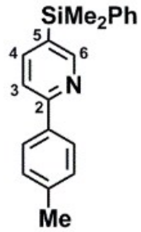

Prepared according to the general procedure from 2-(*p*-tolyl)pyridine (23 µL, 0.13 mmol) and dimethylphenylsilane (61 µL, 0.40 mmol) at 110 ºC for 24 h, to provide the title compound as yellow oil, which purified by column chromatography using a mixture of 90/10 hexane/ethyl acetate. Isolated yield: 90%. ^{1}H NMR (400 MHz, $CDCl_3$, 298 K): δ 8.79 (dd, J_{H-H} = 1.9, 1.0, 1H, H_{6-py}), 7.84 (dd, J_{H-H} = 7.9, 1.9, 1H, H_{4-py}), 7.69 (dd, J_{H-H} = 7.9, 1.0, 1H, H_{3-py}), 7.93 (d, J_{H-H} = 8.3, 2H, H_{o-Phpy}), 7.30 (d, J_{H-H} = 8.3, 2H, H_{m-Phpy}), 7.57 (d, J_{H-H} = 8.7, 2H, H_{o-PhSi}), 7.41 (dd, J_{H-H} = 8.7, 6.9, 2H, H_{m-PhSi}), 7.39 (t, J_{H-H} = 6.9, 1H, H_{p-PhSi}), 2.43 (s, 3H, Me), 0.63 (s, 6H, SiMe). ^{13}C {^{1}H}-APT NMR plus HSQC and HMBC (100 MHz, $CDCl_3$, 298 K): δ 157.6 (s, C_{2-py}), 154.4 (s, C_{6-py}), 142.7 (s, C_{4-py}), 139.1 (s, C_{q-Me}), 137.1 (s, C_{q-PhSi}), 136.5 (s, C_{q-Phpy}), 134.1 (s, C_{o-PhSi}), 130.9 (s, C_{5-py}), 129.5 (s, C_{m-Phpy}), 129.4 (s, C_{p-PhSi}), 128.0 (s, C_{m-PhSi}), 126.7

(s, $C_{o\text{-}Phpy}$), 119.5 (s, $C_{3\text{-}py}$), 21.2 (s, Me), –2.5 (s, SiMe). ^{29}Si {^{1}H} NMR (79 MHz, $CDCl_3$, 298 K): δ –8.74. GC-MS: 303 [M^+], 288 [M^+ - Me], 272, 257, 244, 226, 210, 196, 182, 169, 155, 144, 129, 115, 105.

5-(methyldiphenylsilyl)-2-(p-tolyl)pyridine

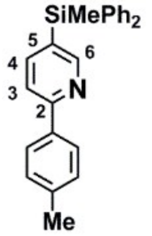

Prepared according to the general procedure from 2-(*p*-tolyl)pyridine (23 μL, 0.13 mmol) and methyldiphenylsilane (79 μL, 0.40 mmol) at 110 ºC for 48 h, to provide the title compound as colorless oil, which was purified by column chromatography using a mixture of 90/10 hexane/ethyl acetate. Isolated yield: 51%. ^{1}H RMN (400 MHz, $CDCl_3$, 298K): δ 8.68 (dd, $J_{H\text{-}H}$ = 1.0, 1.8, 1H, $H_{6\text{-}py}$), 7.85 (d, $J_{H\text{-}H}$ = 8.1, 2H, $H_{o\text{-}Phpy}$), 7.76 (dd, $J_{H\text{-}H}$ = 8.0, 1.8, $H_{4\text{-}py}$), 7.63 (dd, $J_{H\text{-}H}$ = 8.0, 1.0, 1H, $H_{3\text{-}py}$), 7.47 (d, $J_{H\text{-}H}$ = 7.7, 2H, $H_{o\text{-}Ph}$), 7.34 (t, $J_{H\text{-}H}$ = 7.0, 1H, $H_{p\text{-}Ph}$), 7.32 (dd, $J_{H\text{-}H}$ = 7.7, 7.0, 2H, $H_{m\text{-}Ph}$), 7.21 (d, $J_{H\text{-}H}$ = 8.1, 2H, $H_{m\text{-}Phpy}$), 2.33 (s, 3H, Me), 0.82 (s, 3H, SiMe). ^{13}C {^{1}H} (100 MHz, $CDCl_3$, 298K): δ 157.6 (s, $C_{2\text{-}py}$), 155.1 (s, $C_{6\text{-}py}$), 143.9 (s, $C_{4\text{-}py}$), 139.5 (s, $C_{q\text{-}Me}$), 135.2 (s, $C_{o\text{-}Ph}$), 134.9 (s, $C_{q\text{-}Ph}$), 129.8 (s, $C_{p\text{-}Ph}$), 129.6 (s, $C_{m\text{-}Phpy}$), 129.2 (s, $C_{5\text{-}py}$), 128.1 (s, $C_{m\text{-}Ph}$), 126.8 (s, $C_{o\text{-}Phpy}$), 119.7 (s, $C_{3\text{-}py}$), 21.3 (s, Me), –3.6 (s, SiMe). ^{29}Si {^{1}H} NMR (79 MHz, $CDCl_3$, 298 K): δ -12.72. GC-MS: 365 [M^+], 350 [M^+ - Me], 330, 317, 300, 288, 271, 253, 239, 224, 207, 194, 178, 167, 153, 128, 116, 105, 91.

Methyltriphenylsilane

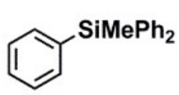

Prepared according to the general procedure from benzene (12 μL, 0.13 mmol) and diphenylmethylsilane (79 μL, 0.40 mmol) at 110 ºC for 24 h, to provide the title compound as colorless oil, which was purified by column chromatography using a mixture 90/10, hexane/ethyl acetate. Isolated yield: 64%. ^{1}H NMR (400 MHz, $CDCl_3$, 298 K): δ 7.64 (d, $J_{H\text{-}H}$ = 7.7, 6H, $H_{o\text{-}Ph}$), 7.43 (t, $J_{H\text{-}H}$ = 6.9, 3H, $H_{p\text{-}Ph}$), 7.42 (dd, $J_{H\text{-}H}$ = 7.7, 6.9, 6H, $H_{m\text{-}Ph}$), 0.70 (s, 3H, SiMe). ^{13}C {^{1}H}-APT NMR plus HSQC and HMBC (100 MHz, $CDCl_3$, 298 K): δ 137.0 (s, $C_{q\text{-}Ph}$), 134.0 (s, $C_{o\text{-}Ph}$), 129.9 (s, $C_{p\text{-}Ph}$), 127.9 (s, $C_{m\text{-}Ph}$), -1.2 (s, SiMe). ^{29}Si {^{1}H} NMR (79 MHz, $CDCl_3$, 298 K): δ -3.43. GC-MS: 273 [M^+ -1], 253, 223, 207, 195, 181, 165, 154, 145, 133, 120, 102, 91, 77.

Dimethyldiphenylsilane

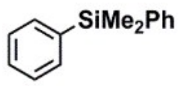

Prepared according to the general procedure from benzene (12 µL, 0.13 mmol) and dimethylphenylsilane (61 µL, 0.40 mmol) at 110 ºC for 24 h, to provide the title compound as light yellow oil, which was purified by column chromatography using a mixture 90/10, hexane/ethyl acetate. Isolated yield: 61%. ^{1}H NMR (400 MHz, $CDCl_3$, 298 K): δ 7.56 (d, $J_{H\text{-}H}$ = 7.7, 4H, $H_{o\text{-}Ph}$), 7.40 (t, $J_{H\text{-}H}$ = 7.0, 2H, $H_{p\text{-}Ph}$), 7.39 (dd, $J_{H\text{-}H}$ = 7.7, 7.0, 4H, $H_{m\text{-}Ph}$), 0.59 (s, 6H, SiMe). ^{13}C {^{1}H}-APT NMR plus HSQC and HMBC (100 MHz, $CDCl_3$, 298 K): δ 138.2 (s, $C_{q\text{-}Ph}$), 134.1 (s, $C_{o\text{-}Ph}$), 129.1 (s, $C_{p\text{-}Ph}$), 127.8 (s, $C_{m\text{-}Ph}$), –2.4 (s, SiMe). ^{29}Si {^{1}H} NMR (79 MHz, $CDCl_3$, 298 K): δ –7.81. GC-MS: 212 [M^+], 197 [M^+ - Me], 181, 172, 165, 154, 145, 135, 128, 119, 113, 105, 93, 85, 79.

Tetraphenylsilane

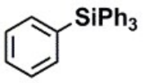

Prepared according to the general procedure from benzene (12 µL, 0.13 mmol) and triphenylsilane (103 mg, 0.40 mmol) at 110 ºC for 24 h, to provide the title compound as white solid, which was purified by column chromatography using a mixture 75/25, hexane/ethyl acetate. Isolated yield: 37%. ^{1}H NMR (400 MHz, $CDCl_3$, 298 K): δ 7.56 (d, $J_{H\text{-}H}$ = 7.9, 8H, $H_{o\text{-}Ph}$), 7.37 (t, $J_{H\text{-}H}$ = 7.3, 4H, $H_{p\text{-}Ph}$), 7.31 (dd, $J_{H\text{-}H}$ = 7.9, 7.3, 8H, $H_{m\text{-}Ph}$). ^{13}C {^{1}H}-APT NMR plus HSQC and HMBC (100 MHz, $CDCl_3$, 298 K): δ 135.1 (s, $C_{q\text{-}Ph}$), 135.0 (s, $C_{o\text{-}Ph}$), 130.1 (s, $C_{p\text{-}Ph}$), 128.0 (s, $C_{m\text{-}Ph}$). ^{29}Si {^{1}H} NMR (79 MHz, $CDCl_3$, 298 K): δ -–13.54. GC-MS: 336 [M^+], 313, 300, 285, 275, 259, 241, 229, 215, 205, 191, 182, 165, 155, 139, 129, 115, 105, 91, 77.

triethyl(thiophen-2-yl)silane

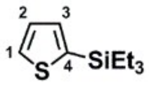

Prepared according to the general procedure from thiophene (11 µL, 0.13 mmol) and triethylsilane (63 µL, 0.40 mmol) at 110 ºC for 24 h, to provide the title compound as colorless oil, which was purified by column chromatography using 100% hexane as eluent. Isolated yield: 75%. ^{1}H NMR (300 MHz, $CDCl_3$, 298 K): δ 7.53 (d, $J_{H\text{-}H}$ = 4.6, 1H, $H_{1\text{-}th}$), 7.19 (d, $J_{H\text{-}H}$ = 3.3, 1H, $H_{3\text{-}th}$), 7.13 (dd, $J_{H\text{-}H}$ = 4.6, 3.3, 1H, $H_{2\text{-}th}$), 0.92 (t, $J_{H\text{-}H}$ = 7.9, 3H, $SiCH_2CH_3$), 0.74 (q, $J_{H\text{-}H}$ = 7.9, 2H, $SiCH_2CH_3$). ^{13}C {^{1}H}-APT NMR plus HSQC and HMBC (75 MHz, $CDCl_3$, 298 K): δ 136.4 (s, $C_{4\text{-}th}$), 134.5 (s, $C_{3\text{-}th}$), 130.3 (s, $C_{1\text{-}th}$), 128.0 (s, $C_{2\text{-}th}$), 7.3 (s, $SiCH_2CH_3$), 4.5 (s, $SiCH_2CH_3$). ^{29}Si {^{1}H} NMR (59 MHz, $CDCl_3$, 298 K): δ -0.33. GC-MS: 198 [M^+], 169 [M^+ - Et], 141, 127, 113, 107, 87.

triethyl(5-methylthiophen-2-yl)silane

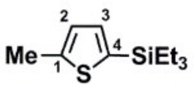

Prepared according to the general procedure from 2-methylthiophene (13 µL, 0.13 mmol) and triethylsilane (63 µL, 0.40 mmol) at 110 ºC for 24 h, to provide the title compound as colorless oil, which was purified by column chromatography using a mixture 95/5, hexane/ethyl acetate. Isolated yield: 77%. ^{1}H NMR (400 MHz, $CDCl_3$, 298 K): δ 7.08 (d, J_{H-H} = 3.2, 1H, H_{3-th}), 6.88 (dq, J_{H-H} = 3.2, 1.0, $1H_{2-th}$), 2.57 (d, J_{H-H} = 1.0, 3H, Me), 1.03 (t, J_{H-H} = 8.3, 3H, $SiCH_2CH_3$), 0.81 (q, J_{H-H} = 8.3, 2H, $SiCH_2CH_3$). ^{13}C {^{1}H}-APT NMR plus HSQC and HMBC (100 MHz, $CDCl_3$, 298 K): δ 145.2 (s, C_{1-th}), 134.9 (s, C_{3-th}), 134.5 (s, C_{4-th}), 126.7 (s, C_{2-th}), 15.1 (s, Me), 7.4 (s, $SiCH_2CH_3$), 4.5 (s, $SiCH_2CH_3$). ^{29}Si {^{1}H} NMR (79 MHz, $CDCl_3$, 298 K): δ –1.12. GC-MS: 212 [M^+], 197 [M^+ - Me], 181, 155, 127, 121, 111, 97, 87, 77.

methyl(5-methylthiophen-2-yl)diphenylsilane

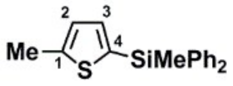

Prepared according to the general procedure from 2-methylthiophene (13 µL, 0.13 mmol) and diphenylmethylsilane (79 µL, 0.40 mmol) at 110 ºC for 24 h, to provide the title compound as colorless oil, which was purified by column chromatography using a mixture 95/5, hexane/ethyl acetate. Isolated yield: 78%. ^{1}H NMR (400 MHz, $CDCl_3$, 298 K): δ 7.62 (d, J_{H-H} = 7.7, 4H, H_{o-PhSi}), 7.44 (t, J_{H-H} = 6.9, 2H, H_{p-PhSi}), 7.42 (dd, J_{H-H} = 7.7, 6.9, 4H, H_{m-PhSi}), 7.13 (d, J_{H-H} = 3.2, 1H, H_{3-th}), 6.91 (dq, J_{H-H} = 3.2, 1.1, 1H, H_{2-th}), 2.57 (d, J_{H-H} = 1.0, 3H, Me), 0.89 (s, SiMe). ^{13}C {^{1}H}-APT NMR plus HSQC and HMBC (100 MHz, $CDCl_3$, 298 K): δ 146.9 (s, C_{1-th}), 137.3 (s, C_{3-th}), 136.0 (s, C_{q-PhSi}), 135.0 (s, C_{o-PhSi}), 133.3 (s, C_{4-th}), 129.6 (s, C_{p-PhSi}), 127.9 (s, C_{m-PhSi}), 127.0 (s, C_{2-th}), 15.2 (s, Me), –2.3 (s, SiMe). ^{29}Si {^{1}H} NMR (79 MHz, $CDCl_3$, 298 K): δ –17.16. GC-MS: 294 [M^+], 279 [M^+ - Me], 239, 217, 202, 197, 181, 165, 155, 137, 125, 115, 105.

benzo[b]thiophen-2-yltriethylsilane

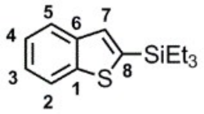

Prepared according to the general procedure from benzothiophene (18 mg, 0.13 mmol) and triethylsilane (63 µL, 0.40 mmol) at 110 ºC for 24 h, to provide the title compound as colorless oil, which was purified by column chromatography using a mixture 90/10, hexane/ethyl acetate. Isolated yield: 73%. ^{1}H NMR (300 MHz, $CDCl_3$, 298 K): δ 7.88 (d, J_{H-H} = 7.9, 1H, H_2), 7.82 (d, J_{H-H} = 6.9, 1H, H_5), 7.46 (s, 1H, H_7), 7.34 (m, 1H, H_4), 7.29 (m, 1H, H_3), 1.03 (t, J_{H-H} = 7.1, 3H, $SiCH_2CH_3$), 0.88 (q, J_{H-H} = 7.1, 2H, $SiCH_2CH_3$). ^{13}C {^{1}H}-APT NMR plus HSQC and HMBC (75 MHz, $CDCl_3$, 298 K): δ 143.7 (s, C_6), 141.0 (s, C_1), 139.0 (s, C_8), 131.6 (s, C_7), 124.0 and 123.9 (both s, $C_{4,5}$), 123.3 (s, C_5), 122.2 (s, C_2), 7.3 (s, $SiCH_2CH_3$), 4.3 (s, $SiCH_2CH_3$). ^{29}Si {^{1}H} NMR (59 MHz, $CDCl_3$, 298 K): δ 1.17. GC-MS: 248 [M^+], 219 [M^+ - Et], 191, 177, 163, 147, 134, 127, 115, 103, 96.

3,5-dimethylphenyl)triethylsilane

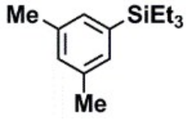

Prepared according to the general procedure from *m*-xylene (16 µL, 0.13 mmol) and triethylsilane (63 µL, 0.40 mmol) at 110 ºC for 24 h, to provide the title compound as colorless oil, which was purified by column chromatography using 100% hexane as eluent. Isolated yield: 78%. ^{1}H NMR (300 MHz, $CDCl_3$, 298 K): δ 7.09 (m, 2H, $H_{o\text{-}Ph}$), 6.99 (m, 1H, $H_{p\text{-}Ph}$), 2.31 (m, 6H, Me), 0.96 (t, $J_{H\text{-}H}$ = 7.1, 3H, $SiCH_2CH_3$), 0.79 (q, $J_{H\text{-}H}$ = 7.1, 2H, $SiCH_2CH_3$). ^{13}C {^{1}H}-APT NMR plus HSQC and HMBC (75 MHz, $CDCl_3$, 298 K): δ 137.2 (s, $C_{q\text{-}Si}$), 136.8 (s, $C_{q\text{-}Me}$), 131.9 (s, $C_{o\text{-}Ph}$), 130.5 (s, $C_{p\text{-}Ph}$), 21.4 (s, Me), 7.5 (s, $SiCH_2CH_3$), 3.4 (s, $SiCH_2CH_3$). ^{29}Si {^{1}H} NMR (59 MHz, $CDCl_3$, 298 K): δ 0.89. GC-MS: 220 [M^+], 191 [M^+ - Et], 175, 163, 147, 135, 129, 119, 105, 91.

triethyl(naphthalen-2-yl)silane

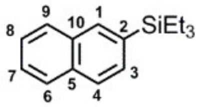

Prepared according to the general procedure from naphthalene (17 mg, 0.13 mmol) and triethylsilane (63 µL, 0.40 mmol) at 110 ºC for 24 h, to provide the title compound as colorless oil, which was purified by column chromatography using 100% hexane as eluent. Isolated yield: 58%. ^{1}H NMR (400 MHz, $CDCl_3$, 298 K): δ 8.00 (s, 1H, $H_{1\text{-}Ph}$), 7.90 and 7.88 (both d, $J_{H\text{-}H}$ = 6.3, 2H, $H_{6,9\text{-}Ph}$), 7.84 (d, $J_{H\text{-}H}$ = 8.2, 1H, $H_{4\text{-}Ph}$), 7.59 (d, $J_{H\text{-}H}$ = 8.2, 1H, $H_{3\text{-}Ph}$), 7.51 and 7.49 (both dd, $J_{H\text{-}H}$ = 6.3, 6.3, 1H, $H_{7,8\text{-}Ph}$), 1.02 (t, $J_{H\text{-}H}$ = 8.0, 3H, $SiCH_2CH_3$), 0.91 (q, $J_{H\text{-}H}$ = 8.0, 2H, $SiCH_2CH_3$). ^{13}C {^{1}H}-APT NMR plus HSQC and HMBC (100 MHz, $CDCl_3$, 298 K): δ 135.0 (s, $C_{2\text{-}Ph}$), 134.8 (s, $C_{1\text{-}Ph}$), 133.6 (s, $C_{10\text{-}Ph}$), 132.9 (s, $C_{5\text{-}Ph}$), 130.6 (s, $C_{3\text{-}Ph}$), 128.0 and 127.6 (both s, $C_{6,9\text{-}Ph}$), 126.7 (s, $C_{4\text{-}Ph}$), 126.1 and 125.7 (both s, $C_{7,8\text{-}Ph}$), 7.5 (s, $SiCH_2CH_3$), 3.4 (s, $SiCH_2CH_3$). ^{29}Si {^{1}H} NMR (79 MHz, $CDCl_3$, 298 K): δ 1.96. GC-MS: 242 [M^+], 227 [M^+ - Me], 214, 197, 186, 169, 155, 143, 135, 121, 107.

triethyl(fluorophenyl)silane

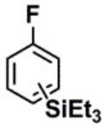

Prepared according to the general procedure from fluorobenzene (12 µL, 0.13 mmol) and triethylsilane (63 µL, 0.40 mmol) at 110 ºC for 24 h, to provide the title compound as a mixture of *o*/*m*/*p* (33/33/33) colorless oil, which was separated by column chromatography using 100% hexane as eluent. Isolated yield: 86%. ^{1}H NMR (400 MHz, $CDCl_3$, 298 K): δ 7.5-6.9 (all m, 4H, H_{Ph}), 1.00 (t, $J_{H\text{-}H}$ = 7.1, 9H, $SiCH_2CH_3$), 0.86 (q, $J_{H\text{-}H}$ = 7.1, 6H, $SiCH_2CH_3$). ^{13}C {^{1}H}-APT NMR plus HSQC and HMBC (100 MHz,

$CDCl_3$, 298 K): δ 169.1, 165.0, and 164.3 (all d, J_{C-F} = 290, C_{q-F}), 142.3, 140.7, and 140.6 (all s, C_{q-Si}), 136.0 (d, J_{C-F} = 12.1, C_{Ph}), 135.3 (s, C_{Ph}), 131.1 (d, J_{C-F} = 8.9, C_{Ph}), 129.7 (d, J_{C-F} = 3.8, C_{Ph}), 129.4 (d, J_{C-F} = 7.1, C_{Ph}), 123.7 (d, J_{C-F} = 5.1, C_{Ph}), 120.4 (d, J_{C-F} = 18.4, C_{Ph}), 115.6 (d, J_{C-F} = 21.5, C_{Ph}), 114.8 (d, J_{C-F} = 20.4, C_{Ph}), 114.7 (d, J_{C-F} = 25.7, C_{Ph}), 7.4, 7.3, and 7.2 (all s, $SiCH_2CH_3$), 4.6, 3.4, and 3.3 (all s, $SiCH_2CH_3$). ^{29}Si {^1H} NMR (79 MHz, $CDCl_3$, 298 K): δ 2.43. GC-MS: 210 [M^+], 182 [M^+ - Et], 153, 139, 125, 105, 91, 77. **NOTE: A mixture of the ortho, meta and para isomers in a 1:1:1 ratio is observed.**

Chlorophenyltriethylsylane

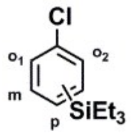

Prepared according to the general procedure from chlorobenzene (14 μL, 0.13 mmol) and triethylsilane (63 μL, 0.40 mmol) at 110 ºC for 24 h, to provide the title compound as colorless oil, which was purified by column chromatography using 100% hexane as eluent. Isolated yield: 84%. *m*-Isomer ^1H NMR (300 MHz, $CDCl_3$, 298 K): δ 7.35 (s, 1H, H_{o2-Ph}), 7.27 (d, J_{H-H} = 6.8, 1H, H_{o1-Ph}), 7.26 (d, J_{H-H} = 7.2, 1H, H_{p-Ph}), 7.21 (dd, J_{H-H} = 7.2, 6.8, 1H, H_{m-Ph}), 0.88 (t, J_{H-H} = 7.2, 9H, $SiCH_2CH_3$), 0.73 (q, J_{H-H} = 7.2, 6H, $SiCH_2CH_3$). ^{13}C {^1H}-APT NMR plus HSQC and HMBC (75 MHz, $CDCl_3$, 298 K): δ 140.3 (s, C_{q-Si}), 134.1 (s, C_{q-Cl}), 133.8 (s, C_{o2-Ph}), 132.0 (s, C_{o1-Ph}), 129.1 (s, C_{m-Ph}), 128.8 (s, C_{p-Ph}), 7.3 (s, $SiCH_2CH_3$), 3.2 (s, $SiCH_2CH_3$). ^{29}Si {^1H} NMR (59 MHz, $CDCl_3$, 298 K): δ 2.18. GC-MS: 226 [M^+], 209 [M^+ - Me], 197 [M^+ - Et], 183, 169, 155, 141, 133, 141, 125, 115, 105, 91. *p*-Isomer ^1H NMR (300 MHz, $CDCl_3$, 298 K): δ 7.33 (d, J_{H-H} = 8.2, 2H, H_{o-Ph}), 7.25 (d, J_{H-H} = 8.2, 2H, H_{m-Ph}), 0.88 (t, J_{H-H} = 7.2, 9H, $SiCH_2CH_3$), 0.73 (q, J_{H-H} = 7.2, 6H, $SiCH_2CH_3$). ^{13}C {^1H}-APT NMR plus HSQC and HMBC (75 MHz, $CDCl_3$, 298 K): δ 135.7 (s, C_{q-Si}), 135.5 (s, C_{o-Ph}), 134.9 (s, C_{q-Cl}), 127.9 (s, C_{m-Ph}), 7.3 (s, $SiCH_2CH_3$), 3.3 (s, $SiCH_2CH_3$). ^{29}Si {^1H} NMR (59 MHz, $CDCl_3$, 298 K): δ 2.18. GC-MS: 226 [M^+], 209 [M^+ - Me], 197 [M^+ - Et], 183, 169, 155, 141, 133, 141, 125, 115, 105, 91.

Triethyl(ethylphenyl)silane

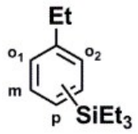

Prepared according to the general procedure from ethylbenzene (16 μL, 0.13 mmol) and triethylsilane (63 μL, 0.40 mmol) at 110 ºC for 24 h, to provide the title compound as colorless oil, which was purified by column chromatography using 100% hexane as eluent. Isolated yield: 88%. ^1H NMR (300 MHz, $CDCl_3$, 298 K): δ 7.43 (d, J_{H-H} = 8.1, 2H, H_{m-Ph}), 7.20 (d, J_{H-H} = 8.1, 2H, H_{o-Ph}), 2.66 (q, J_{H-H} = 7.4, 2H, CH_2CH_3), 1.25 (t, J_{H-H} = 7.4, 3H, CH_2CH_3), 0.99 (t, J_{H-H} = 7.02, 3H, $SiCH_2CH_3$), 0.81 (t, J_{H-H} = 7.02, 3H, $SiCH_2CH_3$). ^{13}C {^1H}-APT NMR plus HSQC and HMBC (75 MHz, $CDCl_3$, 298 K): δ 144.7 (s, C_{q-Et}), 134.2 (s, C_{m-Ph}), 134.0 (s, C_{q-Si}), 127.3 (s, C_{o-Ph}), 28.8 (s, CH_2CH_3), 15.3 (s, CH_2CH_3), 7.4 (s, $SiCH_2CH_3$),

3.4 (s, $SiCH_2CH_3$). ^{29}Si {^1H} NMR (59 MHz, $CDCl_3$, 298 K): δ 1.03. GC-MS: 220 [M^+], 191 [M^+ - Et], 177, 163, 145, 135, 119, 105, 91, 81, 74.

^1H NMR (300 MHz, $CDCl_3$, 298 K): δ 7.34 (s, 1H, $H_{o2\text{-}Ph}$), 7.32 (dd, $J_{H\text{-}H}$ = 7.6, 6.5, 1H, $H_{m\text{-}Ph}$), 7.29 (d, $J_{H\text{-}H}$ = 7.6, 1H, $H_{p\text{-}Ph}$), 7.20 (d, $J_{H\text{-}H}$ = 6.5, 1H, $H_{o1\text{-}Ph}$), 2.66 (q, $J_{H\text{-}H}$ = 7.6, 2H, 2H, CH_2CH_3), 1.26 (t, $J_{H\text{-}H}$ = 7.4, 3H, CH_2CH_3), 0.99 (t, $J_{H\text{-}H}$ = 7.02, 3H, $SiCH_2CH_3$), 0.82 (t, $J_{H\text{-}H}$ = 7.02, 3H, $SiCH_2CH_3$). ^{13}C {^1H}-APT NMR plus HSQC and HMBC (75 MHz, $CDCl_3$, 298 K): δ 143.2 (s, $C_{q\text{-}Et}$), 137.4 (s, $C_{q\text{-}Si}$), 133.7, 131.5, 128.3, and 127.6 (both s, C_{Ph}), 29.0 (s, CH_2CH_3), 15.3 (s, CH_2CH_3), 7.4 (s, $SiCH_2CH_3$), 3.5 (s, $SiCH_2CH_3$). ^{29}Si {^1H} NMR (59 MHz, $CDCl_3$, 298 K): δ 1.03. GC-MS: 220 [M^+], 191 [M^+ - Et], 177, 163, 145, 135, 119, 105, 91, 81, 74.

(sec-butylphenyl)triethylsilane

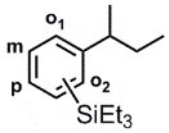

Prepared according to the general procedure from *sec*-butylbenzene (21 µL, 0.13 mmol) and triethylsilane (63 µL, 0.40 mmol) at 110 ºC for 24 h, to provide the title compound as colorless oil, which was obtained purified by column chromatography using 100% hexaneas eluent. Isolated yield: 64%. ^1H NMR (300 MHz, $CDCl_3$, 298 K): δ 7.30 (s, 1H, $H_{o2\text{-}Ph}$), 7.28 (dd, $J_{H\text{-}H}$ = 7.2, 6.7, 1H, $H_{m\text{-}Ph}$), 7.26 (d, $J_{H\text{-}H}$ = 6.7, 1H, $H_{o1\text{-}Ph}$), 7.16 (d, $J_{H\text{-}H}$ = 7.2, 1H, $H_{p\text{-}Ph}$), 2.58 (qd, $J_{H\text{-}H}$ = 7.1, 7.1, 1H, C<u>H</u>Me), 1.59 (dt, $J_{H\text{-}H}$ = 7.4, 7.1, 2H, CH_2CH_3), 1.24 (d, $J_{H\text{-}H}$ = 7.1, 1H, CH*Me*), 0.97 (t, $J_{H\text{-}H}$ = 7.0, 9H, $SiCH_2CH_3$), 0.82 (t, $J_{H\text{-}H}$ = 7.4, 3H, CH_2CH_3), 0.81 (q, $J_{H\text{-}H}$ = 7.0, $SiCH_2CH_3$). ^{13}C {^1H}-APT NMR plus HSQC and HMBC (75 MHz, $CDCl_3$, 298 K): δ 146.4 (s, $C_{q\text{-}Ph}$), 137.2 (s, $C_{q\text{-}Si}$), 133.1 (s, $C_{m\text{-}Ph}$), 131.6 (s, $C_{o2\text{-}Ph}$), 127.5 (s, $C_{o1\text{-}Ph}$), 127.3 (s, $C_{p\text{-}Ph}$), 41.7 (s, <u>C</u>HMe), 31.3 (s, CH_2CH_3), 21.7 (s, CH*Me*), 12.2 (s, CH_2CH_3), 7.4 (s, $SiCH_2CH_3$), 3.5 (s, $SiCH_2CH_3$).). ^{29}Si {^1H} NMR (59 MHz, $CDCl_3$, 298 K): δ 1.01. GC-MS: 248 [M^+], 233 [M^+ - Me], 219 [M^+ - Et], 205, 191, 177, 163, 145, 133, 121, 105, 91.

^1H NMR (300 MHz, $CDCl_3$, 298 K): δ 7.40 (d, $J_{H\text{-}H}$ = 8.1, 2H, $H_{m\text{-}Ph}$), 7.16 (d, $J_{H\text{-}H}$ = 8.1, 2H, $H_{o\text{-}Ph}$), 2.55 (qd, $J_{H\text{-}H}$ = 6.9, 6.9, 1H, C*H*Me), 1.60 (dt, $J_{H\text{-}H}$ = 7.4, 6.9, 6H, CH_2CH_3), 1.24 (d, $J_{H\text{-}H}$ = 6.9, 3H, CH*Me*), 0.97 (t, $J_{H\text{-}H}$ = 7.0, 9H, $SiCH_2CH_3$), 0.81 (t, $J_{H\text{-}H}$ = 7.4, 3H, CH_2CH_3), 0.80 (q, $J_{H\text{-}H}$ = 7.0, 6H, $SiCH_2CH_3$). ^{13}C {^1H}-APT NMR plus HSQC and HMBC (75 MHz, $CDCl_3$, 298 K): δ 148.2 (s, $C_{q\text{-}Ph}$), 137.1 (s, $C_{q\text{-}Si}$), 134.2 (s, $C_{m\text{-}Ph}$), 126.4 (s, $C_{o\text{-}Ph}$), 41.6 (s, *C*HMe), 31.1 (s, CH_2CH_3), 21.5 (s, CH*Me*), 12.3 (s, CH_2CH_3), 7.4 (s, $SiCH_2CH_3$), 3.4 (s, $SiCH_2CH_3$).). ^{29}Si {^1H} NMR (59 MHz, $CDCl_3$, 298 K): δ 1.01. GC-MS: 248 [M^+], 233 [M^+ - Me], 219 [M^+ - Et], 205, 191, 177, 163, 145, 133, 121, 105, 91.

4-Chlorophenyl(dimethylphenyl)sylane

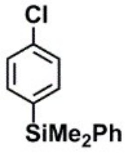

Prepared according to the general procedure from chlorobenzene (14 μL, 0.13 mmol) and dimethylphenylsilane (61 μL, 0.40 mmol) at 110 ºC for 24 h, to provide the title compound as a colorless oil, which was purified by column chromatography using a mixture of hexane/ethyl acetate (80/20). Isolated yield: 64%. ^{1}H NMR (300 MHz, $CDCl_3$, 298 K): δ 7.53 (d, J_{H-H} = 8.6, 2H, H_{o-Ph}), 7.39 (d, J_{H-H} = 8.6, 2H, H_{m-Ph}), 7.38 (d, J_{H-H} = 7.4, 2H, H_{o-PhSi}), 7.35 (dd, J_{H-H} = 7.4, 7.1, 2H, H_{m-PhSi}), 7.34 (t, J_{H-H} = 7.1, 1H, H_{p-PhSi}), 0.58 (s, 6H, SiMe). ^{13}C {^{1}H}-APT NMR plus HSQC and HMBC (75 MHz, $CDCl_3$, 298 K): δ 141.1 and 137.3 (both s, C_{q-Ph}), 134.3 (s, C_{q-Cl}), 134.1 (s, C_{o-Ph}), 132.2 (s, C_{o-PhSi}), 129.4 (s, C_{p-PhSi}), 129.2 (s, C_{m-PhSi}), 127.9 (s, C_{m-Ph}), –2.5 (s, SiMe). ^{29}Si {^{1}H} NMR (59 MHz, $CDCl_3$, 298 K): δ -8.05. GC-MS: 246 [M^+], 231 [M^+ - Me], 215, 195, 181, 173, 165, 152, 127, 119, 105, 91, 77.

1,4-bis(dimethyl(phenyl)silyl)benzene

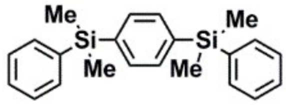

Prepared according to the general procedure from benzene (12 μL, 0.13 mmol) and 1,4-bis(dimethylsilyl)benzene (89 μL, 0.40 mmol) at 110 ºC for 24 h, to provide the title compound as colorless oil, which was purified by column chromatography using a mixture of hexane/ethyl acetate (85/15). Isolated yield: 57%. ^{1}H NMR (300 MHz, $CDCl_3$, 298 K): δ 7.47 (d, J_{H-H} = 8.6, 4H, H_{o-Ph}), 7.45 (dd, J_{H-H} = 8.6, 7.1, 4H, H_{m-Ph}), 7.44 (s, 4H, H_{Si-Ph}), 7.30 (t, J_{H-H} = 7.1, 2H, H_{p-Ph}), 0.48 (SiMe). ^{13}C {^{1}H}-APT NMR plus HSQC and HMBC (75 MHz, $CDCl_3$, 298 K): δ 139.2 and 138.1 (both s, C_{q-Ph}), 134.1 (s, C_{o-Ph}), 133.5 (s, C_{m-Ph}), 129.1 (s, C_{p-Ph}), 127.7 (s, C_{Si-Ph}), 2.4 (s, SiMe). ^{29}Si {^{1}H} NMR (59 MHz, $CDCl_3$, 298 K): δ –7.80. GC-MS: 346 [M^+], 331 [M^+ - Me], 315, 299, 273, 251, 237, 211, 195, 181, 158, 135, 119, 105.

1,4-bis(dimethyl(2-(pyridin-2-yl)thiophen-3-yl)silyl)benzene

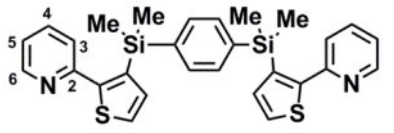

Prepared according to the general procedure from 2-(2-thienyl)pyridine (21 μL, 0.13 mmol) and 1,4-bis(dimethylsilyl)benzene (89 μL, 0.40 mmol) at 110 ºC for 24 h, to provide the title compound as colorless oil, which was purified by column chromatography using a mixture of hexane/ethyl acetate (85/15). Isolated yield: 54%. ^{1}H NMR (300 MHz, $CDCl_3$, 298 K): δ 8.50 (d, J_{H-H} = 4.8, 2H, H_{6-py}), 7.59 (dd, J_{H-H} = 6.6, 7.2, 2H, H_{4-py}), 7.58 (d, J_{H-H} = 6.6, 2H, H_{3-py}), 7.55 (d, J_{H-H} = 3.5, 2H, H_{th-S}), 7.52 (s, 4H, H_{Si-Ph}), 7.19 (d, J_{H-H} = 3.5, 2H, H_{th}), 7.06 (dd, J_{H-H} = 6.2, 4.8, 2H, H_{5-py}), 0.55 (s, SiMe). ^{13}C {^{1}H}-APT NMR plus HSQC and HMBC (75 MHz, $CDCl_3$, 298 K): δ 152.4 (s, C_{2-py}), 150.3 (s, C_{q-th}), 149.6 (s, C_{6-py}), 140.5 (s, C_{q-Si}), 138.6 (s, C_{q-PhSi}), 136.5 (s, C_{4-py}), 136.2 (s, C_{th}), 133.2 (s, C_{Si-Ph}), 125.6 (s, C_{th-S}), 121.8 (s, C_{5-py}), 118.8 (s, C_{3-py}), –1.6 (s, SiMe). ^{29}Si {^{1}H} NMR (59 MHz, $CDCl_3$, 298 K): δ –11.56. HRMS (ESI) m/z calcd. for $C_{28}H_{28}N_2NaS_2Si_2$ (M + Na) 535.1125, found: 535.1112.

1,4-bis(dimethyl(5-methylthiophen-2-yl)silyl)benzene

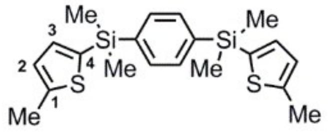

Prepared according to the general procedure from 2-methylthiophene (13 µL, 0.13 mmol) and 1,4-bis(dimethylsilyl)benzene (89 µL, 0.40 mmol) at 110 ºC for 24 h, to provide the title compound as colorless oil, which was purified by after column chromatography using a mixture of hexane/ethyl acetate (85/15). Isolated yield: 72%. ^{1}H RMN (400 MHz, $CDCl_3$, 298K): δ 7.46 (s, 4H, H_{Ph}), 6.98 (d, $J_{H\text{-}H}$ = 3.2, 2H, $H_{2\text{-}th}$), 6.74 (dq, $J_{H\text{-}H}$ = 3.2, 0.9, 2H, $H_{3\text{-}th}$), 2.43 (d, $J_{H\text{-}H}$ = 0.9, 6H, Me), 0.47 (s, 12H, SiMe). ^{13}C {^{1}H} (100.4 MHz, $CDCl_3$, 298K): δ 147.6 (s, $C_{1\text{-}th}$), 140.6 (s, $C_{q\text{-}Ph}$), 137.0 (s, $C_{2\text{-}th}$), 136.9 (s, $C_{4\text{-}th}$), 134.6 (s, C_{Ph}), 128.3 (s, $C_{3\text{-}th}$), 16.5 (s, Me), 0.0 (s, SiMe). ^{29}Si {^{1}H} NMR (79 MHz, $CDCl_3$, 298 K): δ –12.26. GC-MS: 386 [M^+], 371 [M^+ - Me], 355, 341, 313, 296, 281, 259, 243, 231, 215, 201, 178, 155, 141, 127, 115, 103, 91.

1,4-bis(benzo[b]thiophen-2-yldimethylsilyl)benzene

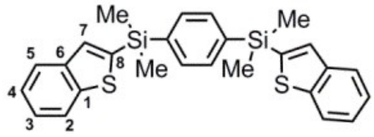

Prepared according to the general procedure from benzothiophene (18 mg, 0.13 mmol) and 1,4-bis(dimethylsilyl)benzene (89 µL, 0.40 mmol) at 110 ºC for 24 h, to provide the title compound as colorless oil, which was purified by column chromatography using a mixture of hexane/ethyl acetate (85/15). Isolated yield: 64%. ^{1}H NMR (400 MHz, $CDCl_3$, 298 K): δ 7.75 (d, $J_{H\text{-}H}$ = 7.5, 1H, H_2), 7.68 (d, $J_{H\text{-}H}$ = 6.8, 1H, H_5), 7.51 (s, 4H, H_{Ph}), 7.38 (s, 2H, H_7), 7.20 (dd, $J_{H\text{-}H}$ = 7.2, 6.8, 1H, H_4), 7.19 (dd, $J_{H\text{-}H}$ = 7.5, 7.2, 1H, H_3), 0.55 (s, 12H, SiMe). ^{13}C {^{1}H}-APT NMR plus HSQC and HMBC (100 MHz, $CDCl_3$, 298 K): δ 145.5 (s, C_1), 142.6 (s, C_6), 141.3 (s, $C_{q\text{-}Ph}$), 140.3 (s, C_8), 135.1 (s, C_{Ph}), 133.9 (s, C_7), 126.0 (s, C_3), 125.7 (s, C_4), 125.2 (s, C_5), 123.8 (s, C_2), 0.0 (s, SiMe). ^{29}Si {^{1}H} NMR (79 MHz, $CDCl_3$, 298 K): δ –10.97. HRMS (ESI) m/z calcd. for $C_{26}H_{26}N_2NaS_2Si_2$ (M + Na) 481.0907, found: 481.0875.

NMR spectra of organometallic compounds

$[Ir(H)_2(IPr)(py)_3][BF_4]$ (**1**)

^{1}H-NMR

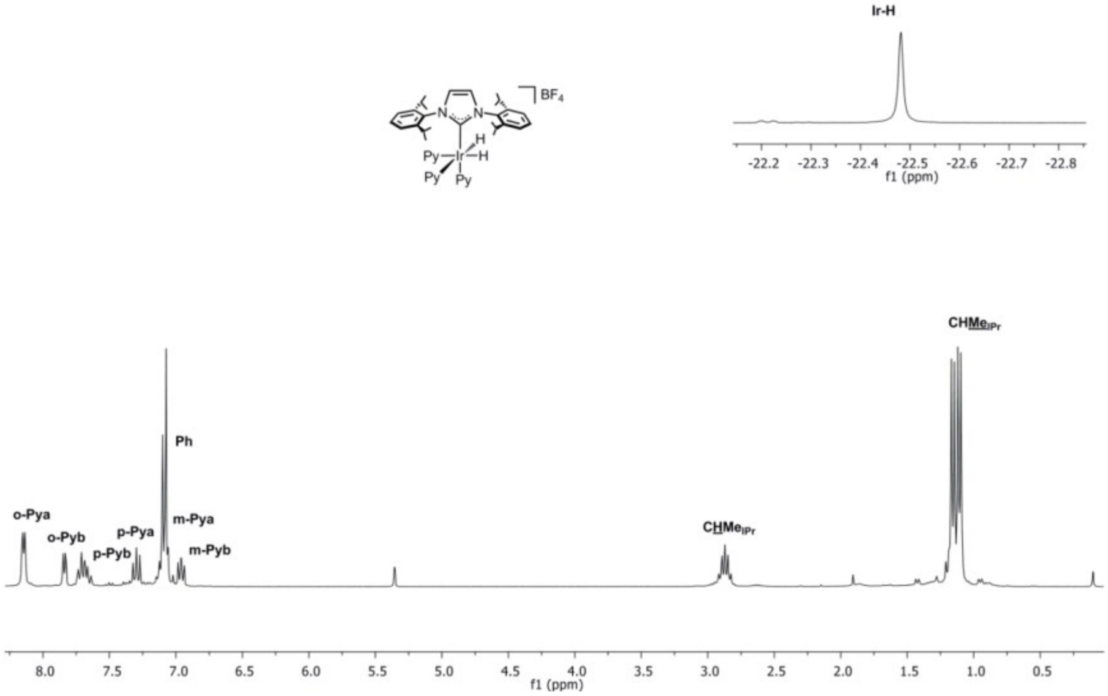

^{13}C-NMR

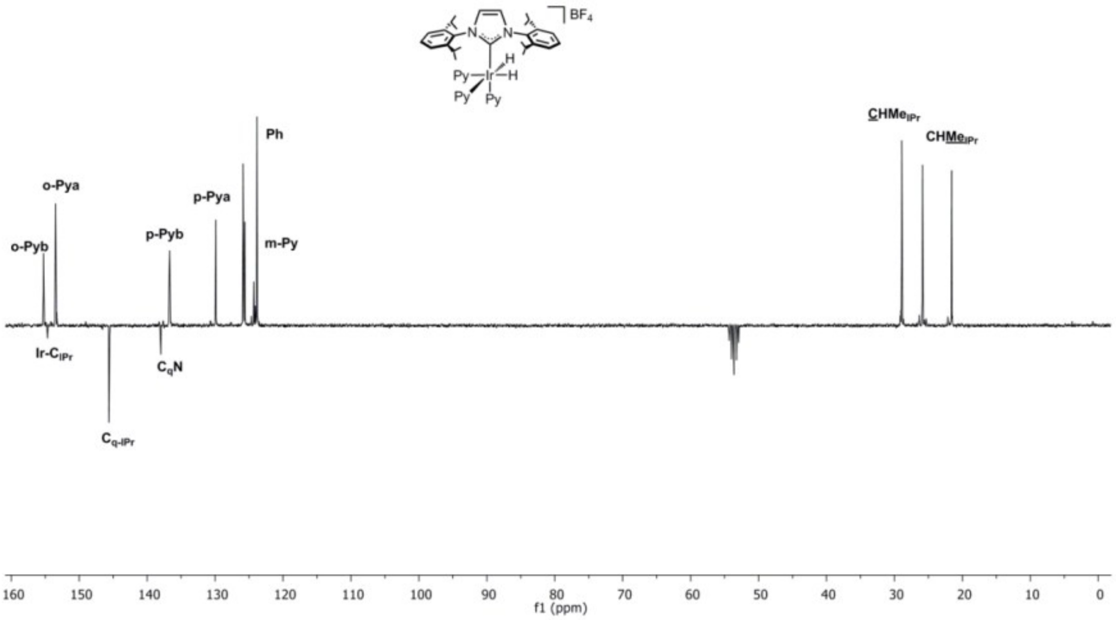

^{1}H-^{1}H COSY

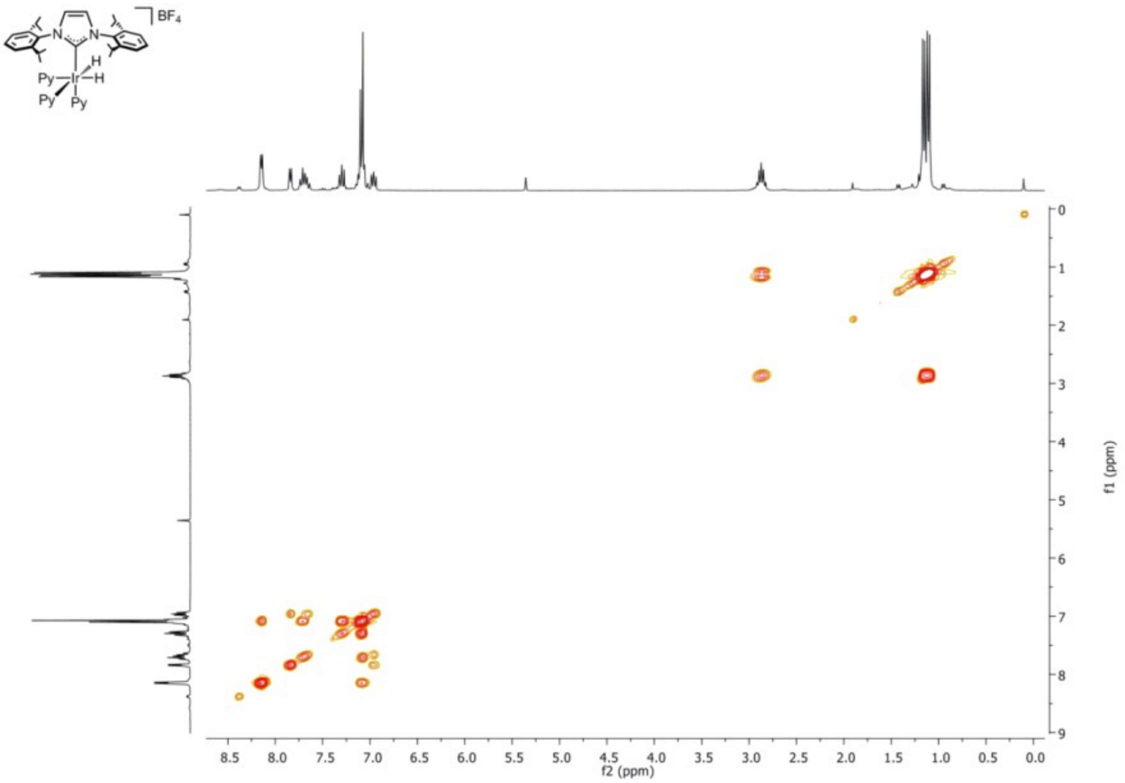

^{1}H-^{13}C HSQC

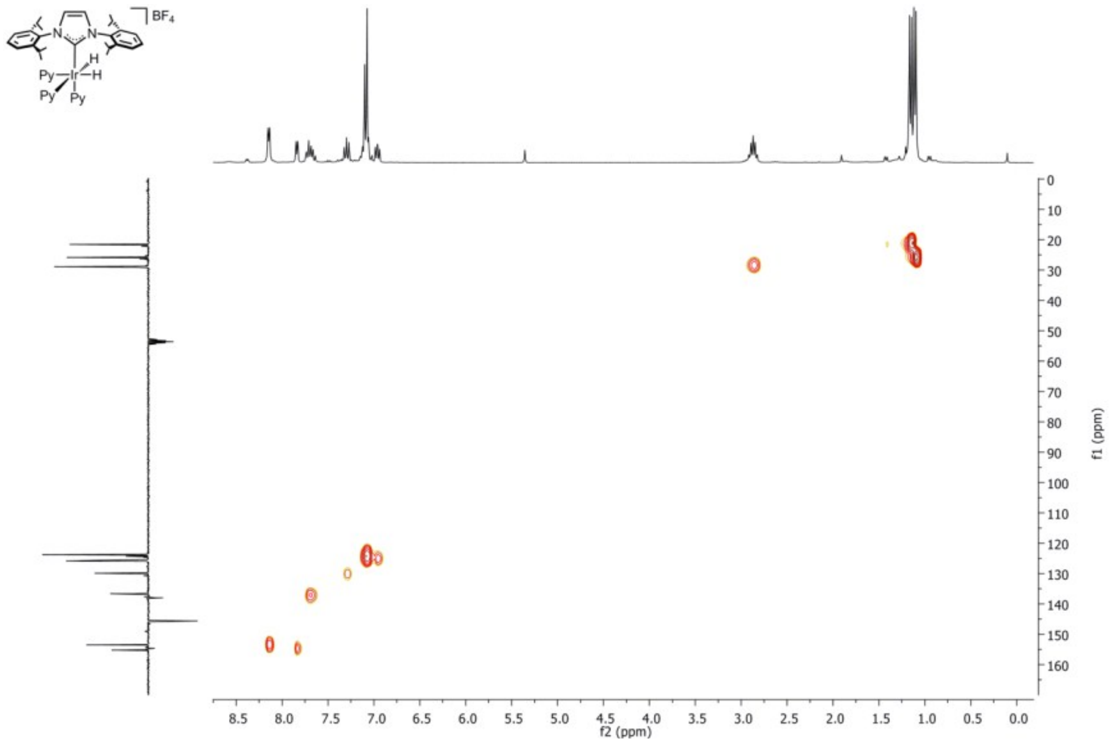

^{1}H-^{13}C HMBC

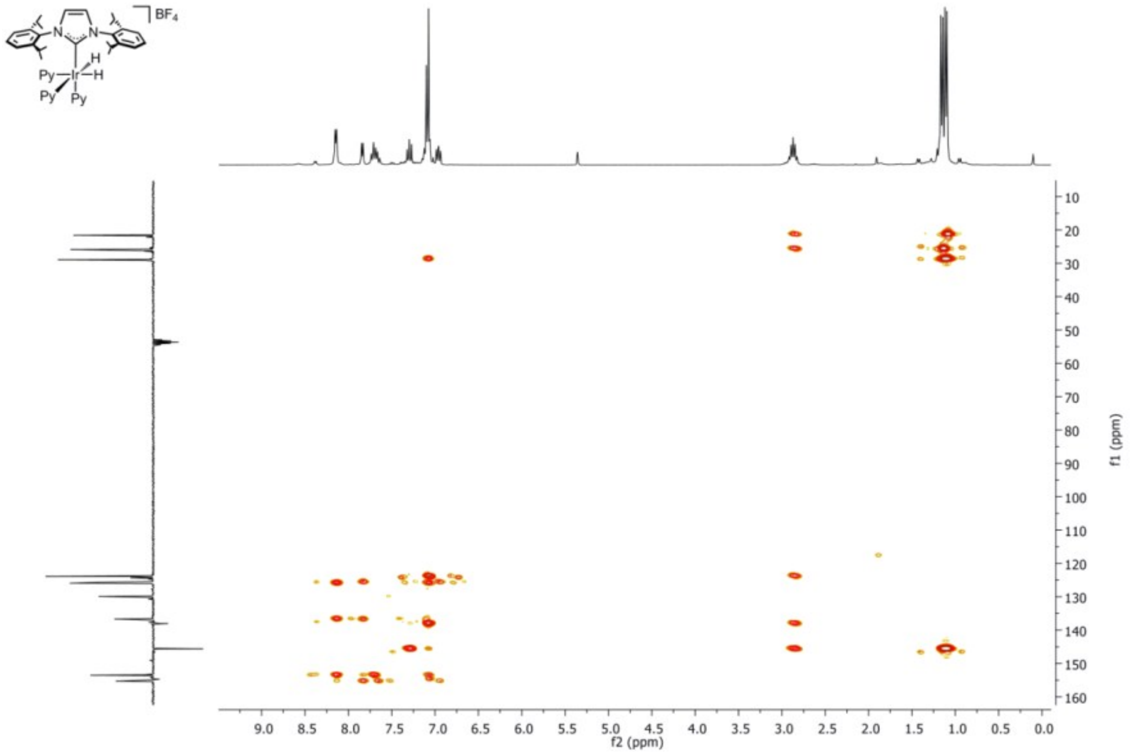

^{19}F NMR

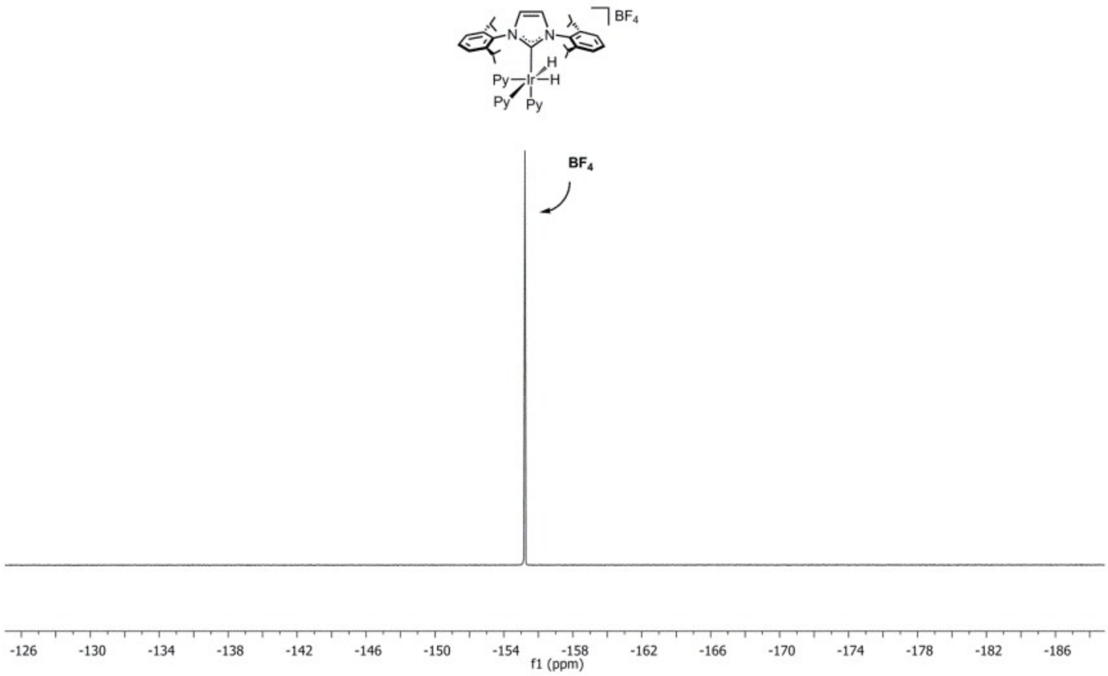

[Ir(H)(IPr)(Phpy-1H)(py)$_2$][BF$_4$] (**2**)

^{1}H NMR

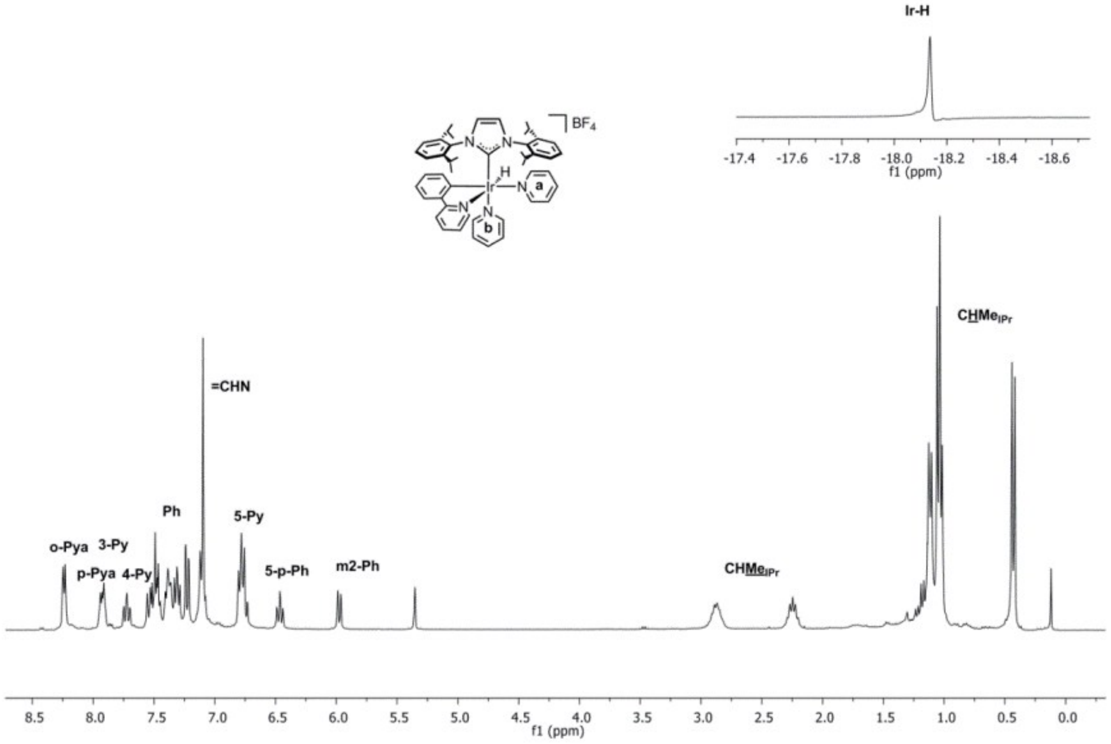

^{13}C NMR

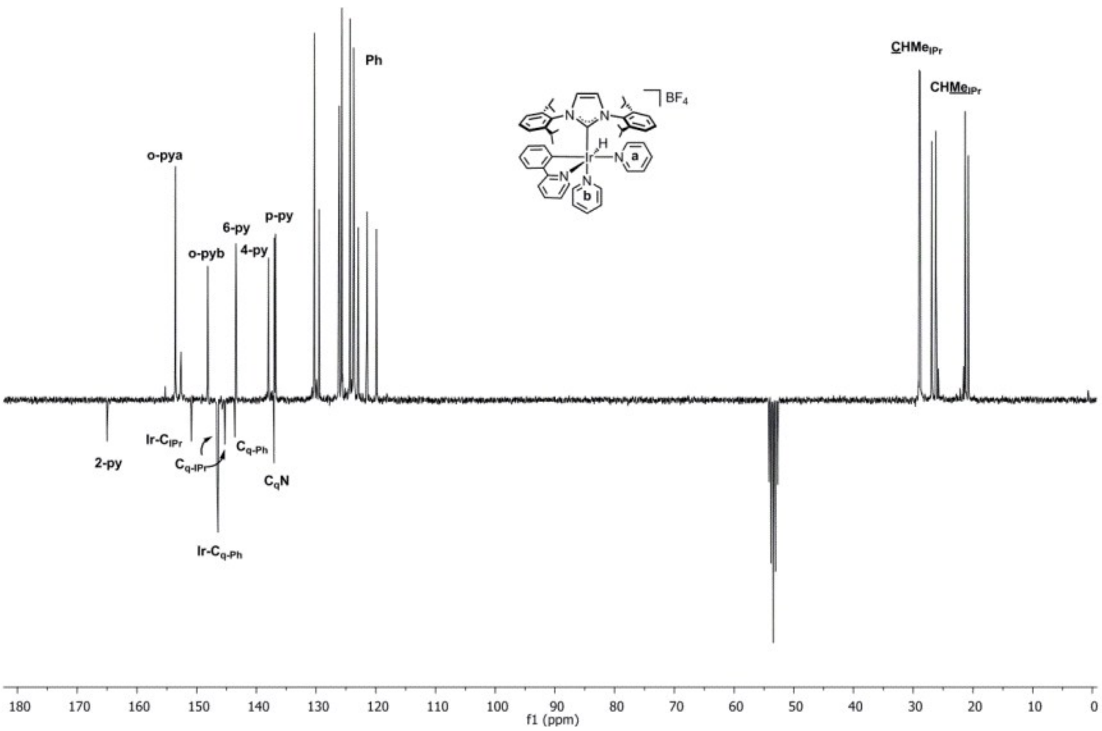

^1H-^1H COSY

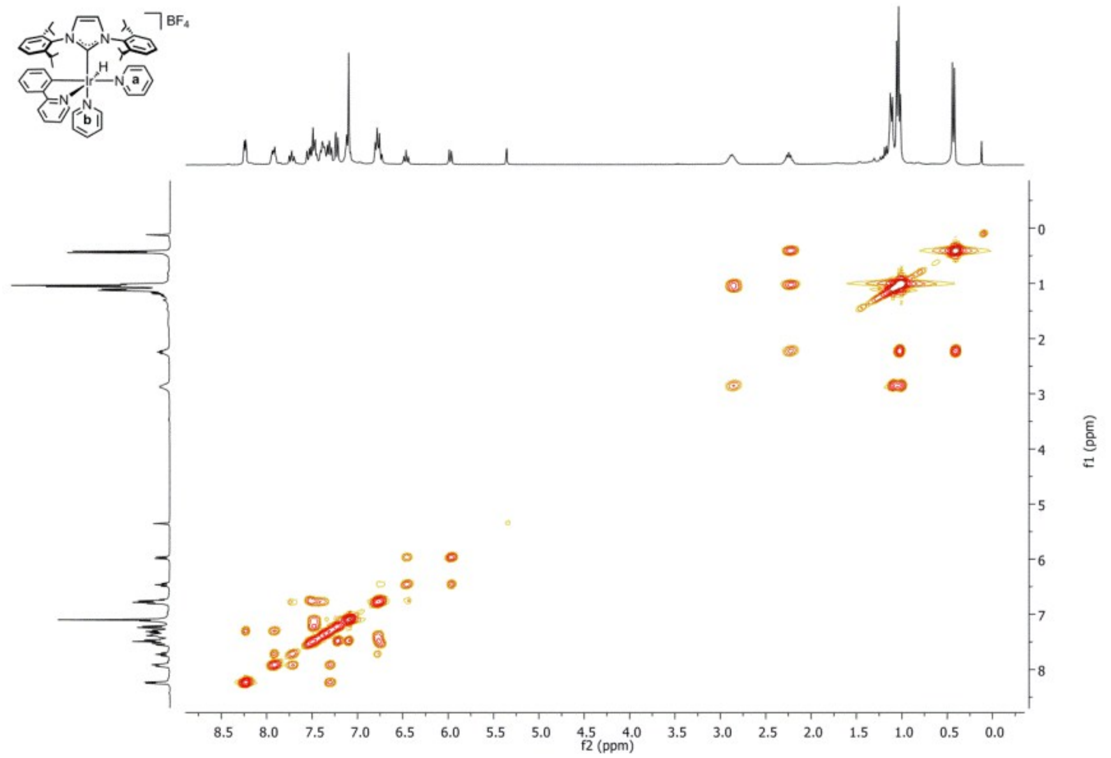

^1H-^{13}C HSQC

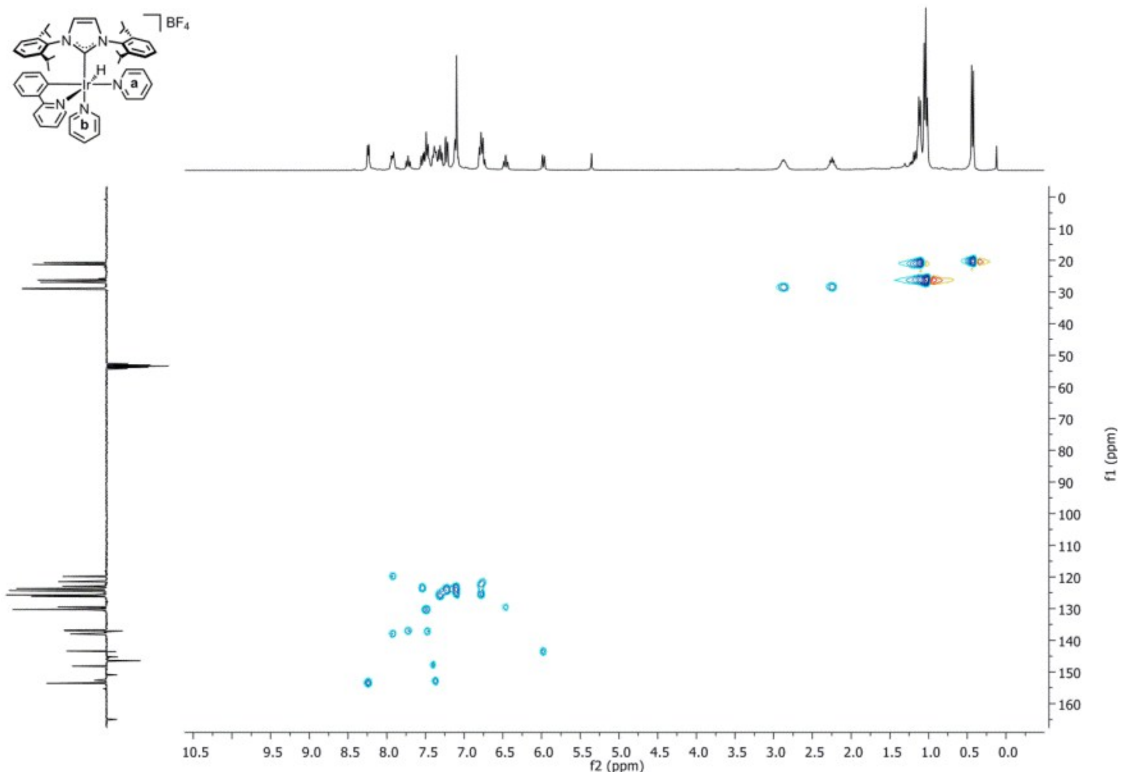

^{1}H-^{13}C HMBC

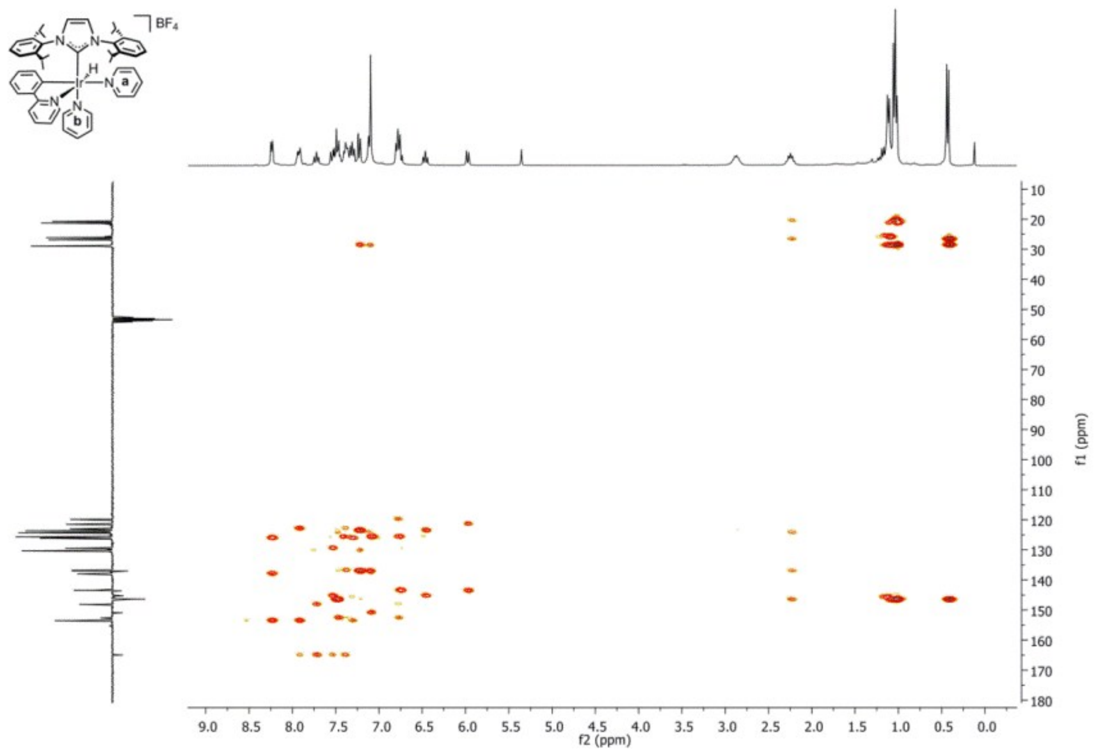

[Ir(H)(IPr)(Thpy-1H)(py)$_2$][BF_4] (**3**)

^{1}H NMR

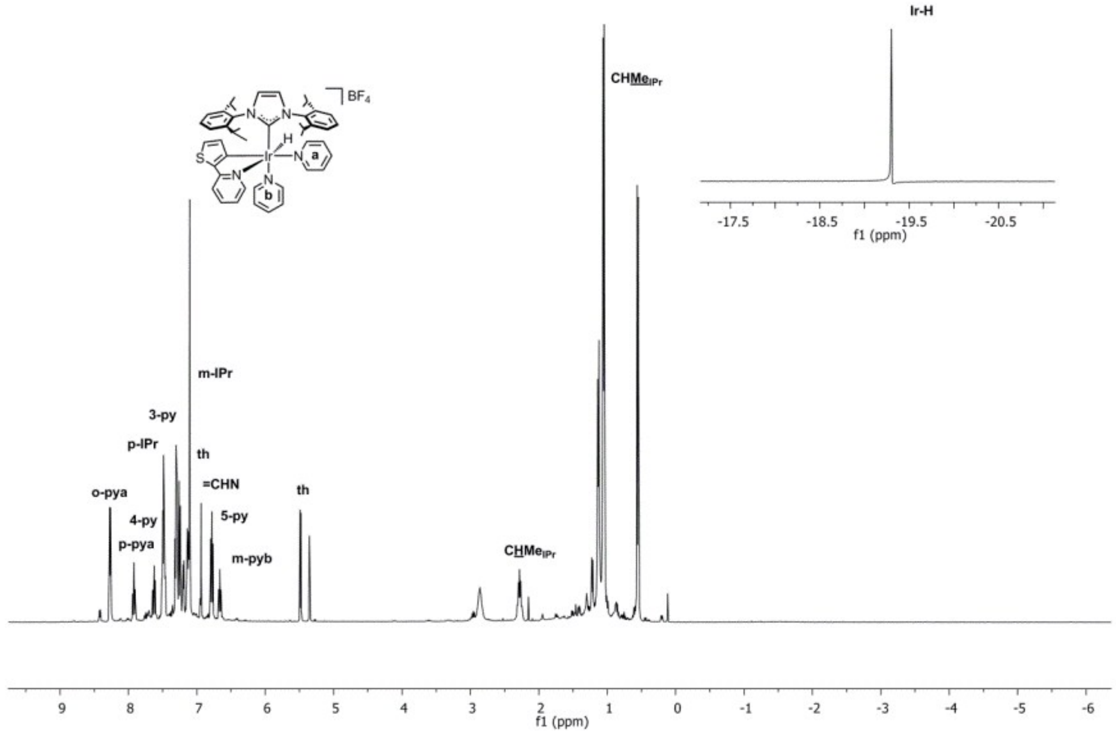

^{13}C NMR

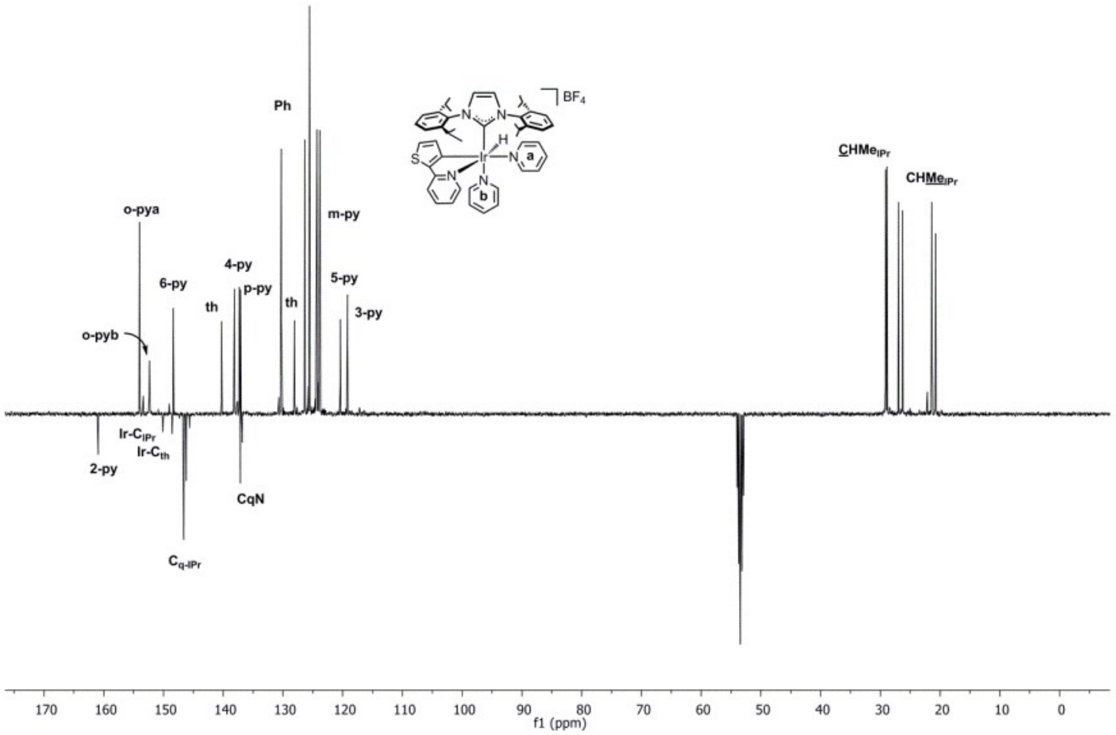

^{1}H-^{1}H COSY

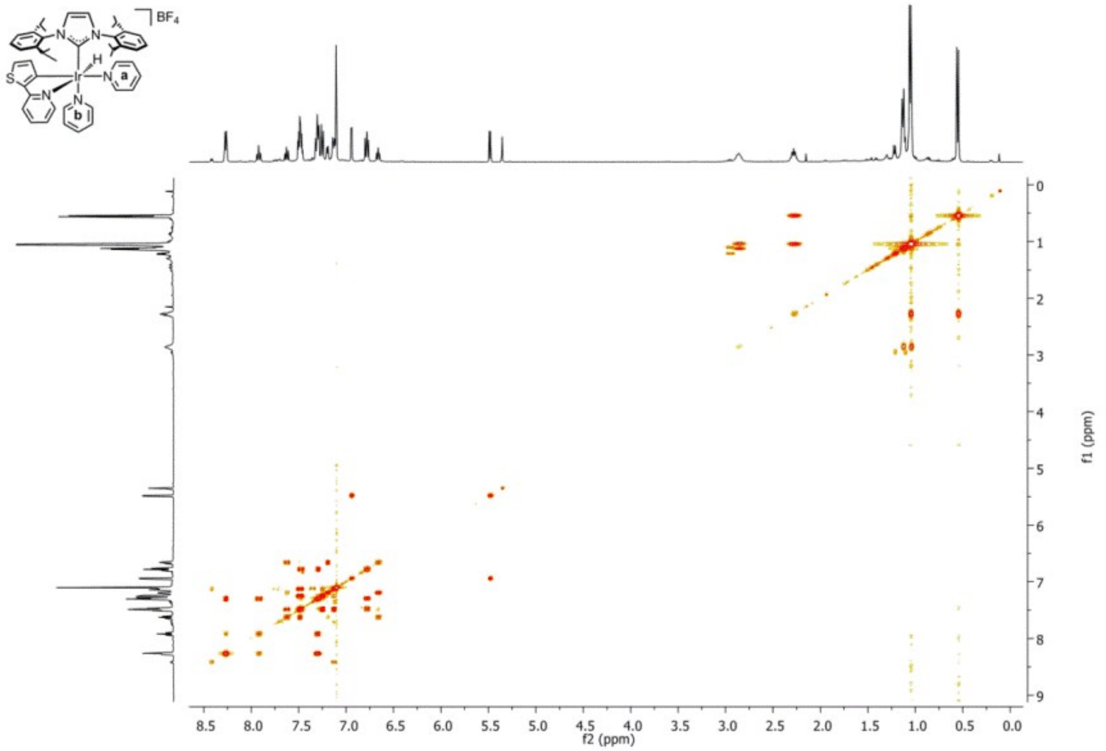

^{1}H-^{13}C HSQC

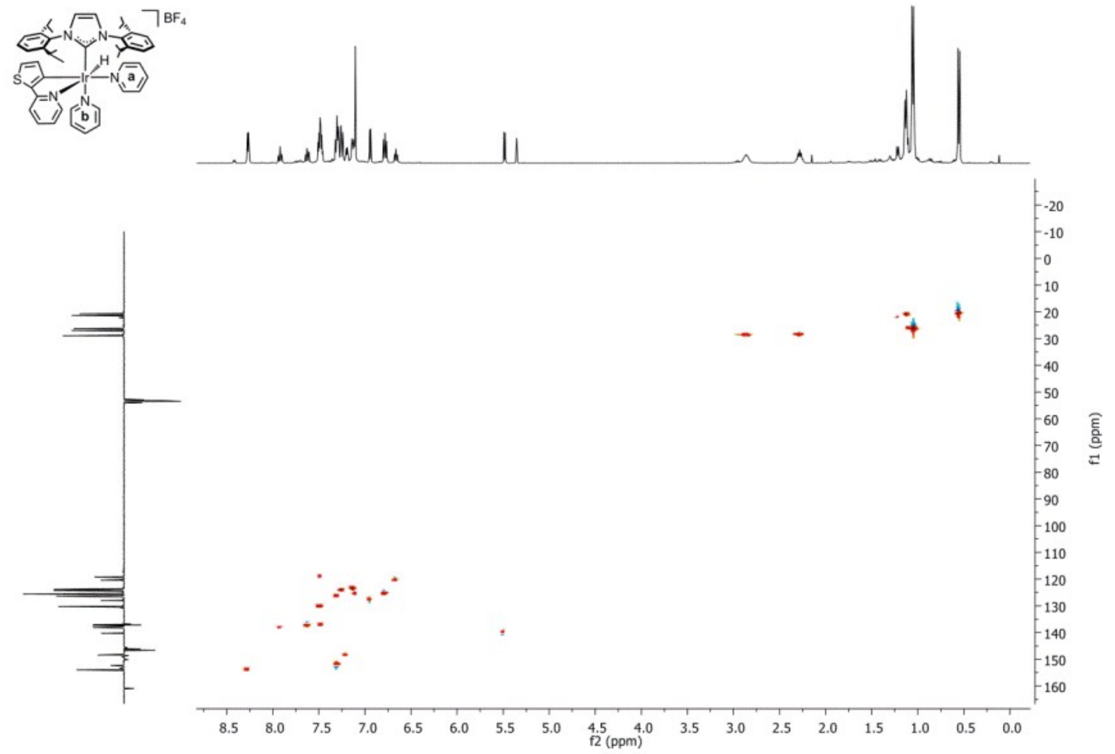

^{1}H-^{13}C HMBC

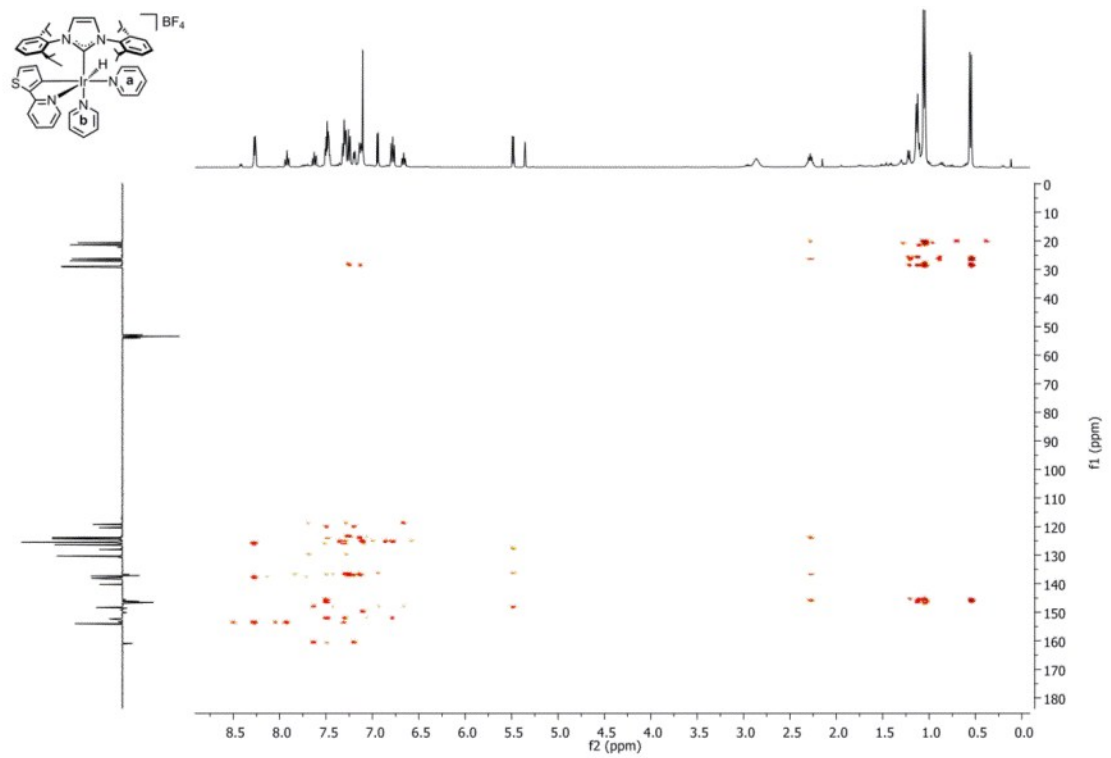

^{19}F NMR

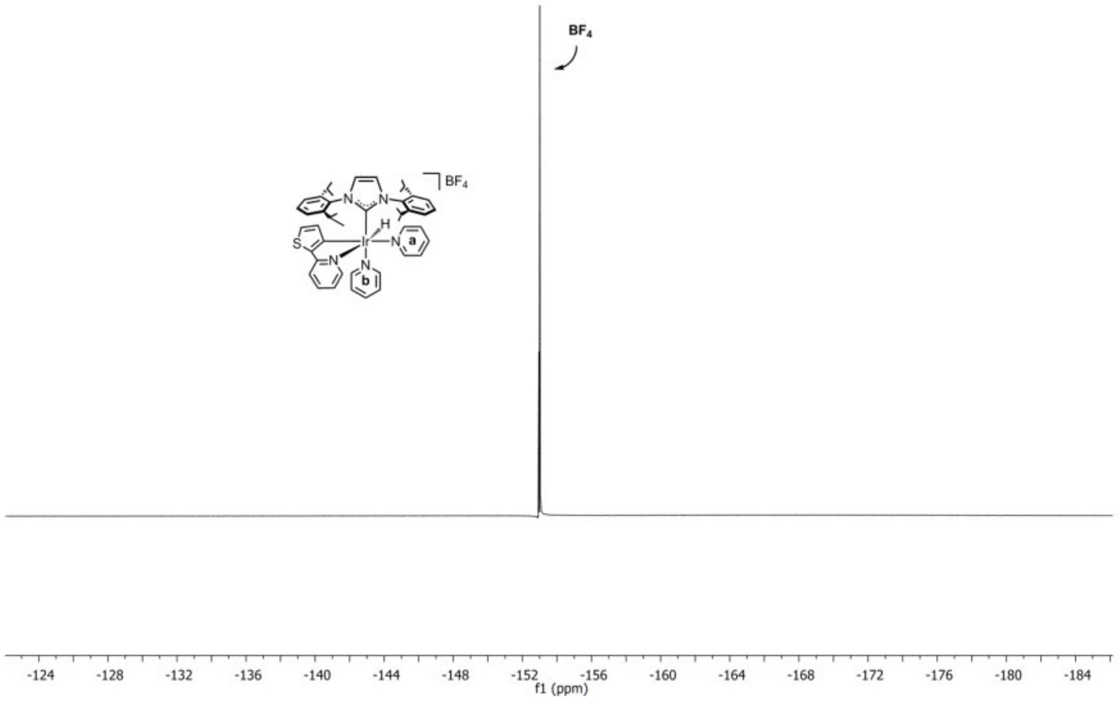

[Ir(H)(IPr)(*p*-tolylpy-1H)(py)$_2$][BF$_4$] (**4**)

^{1}H NMR

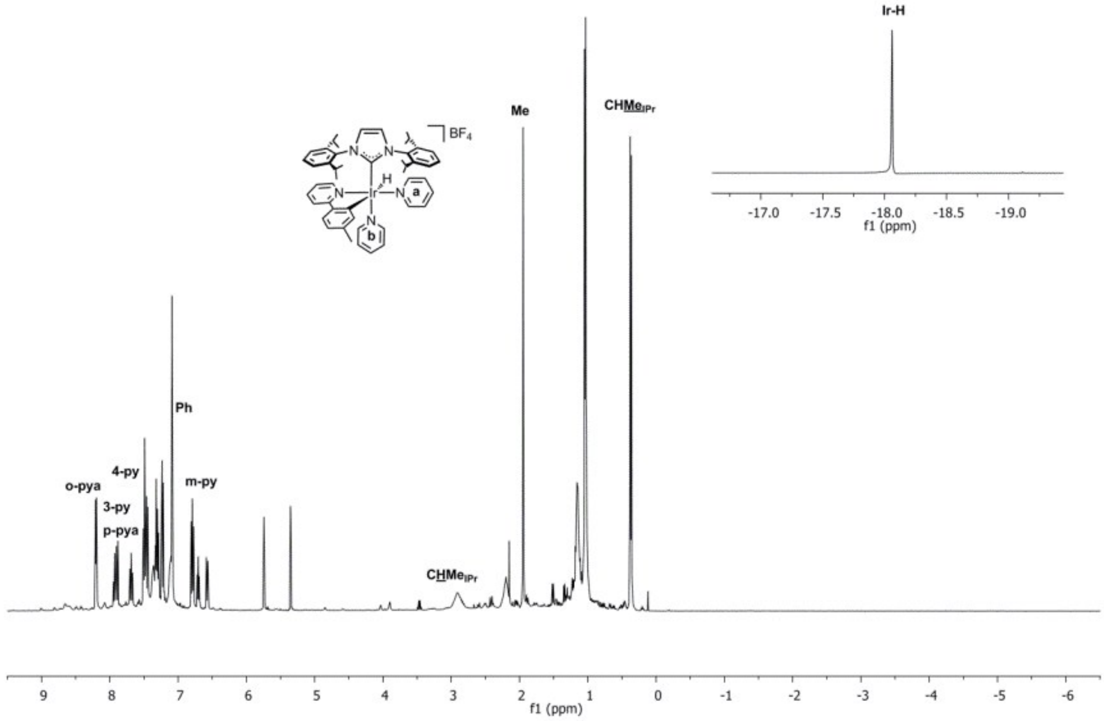

^{13}C NMR

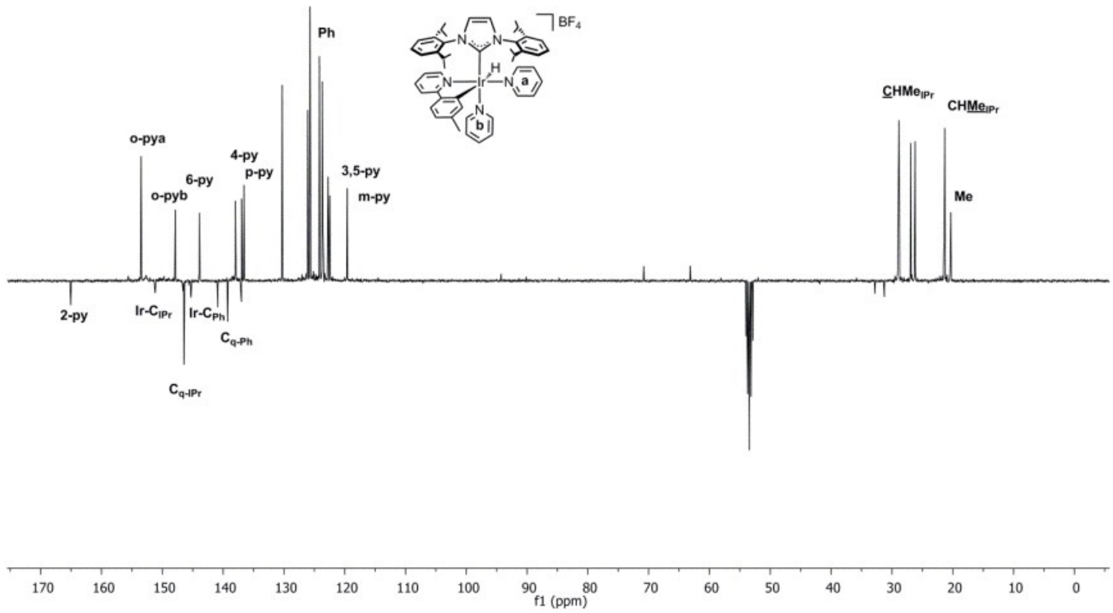

^{1}H-^{1}H COSY

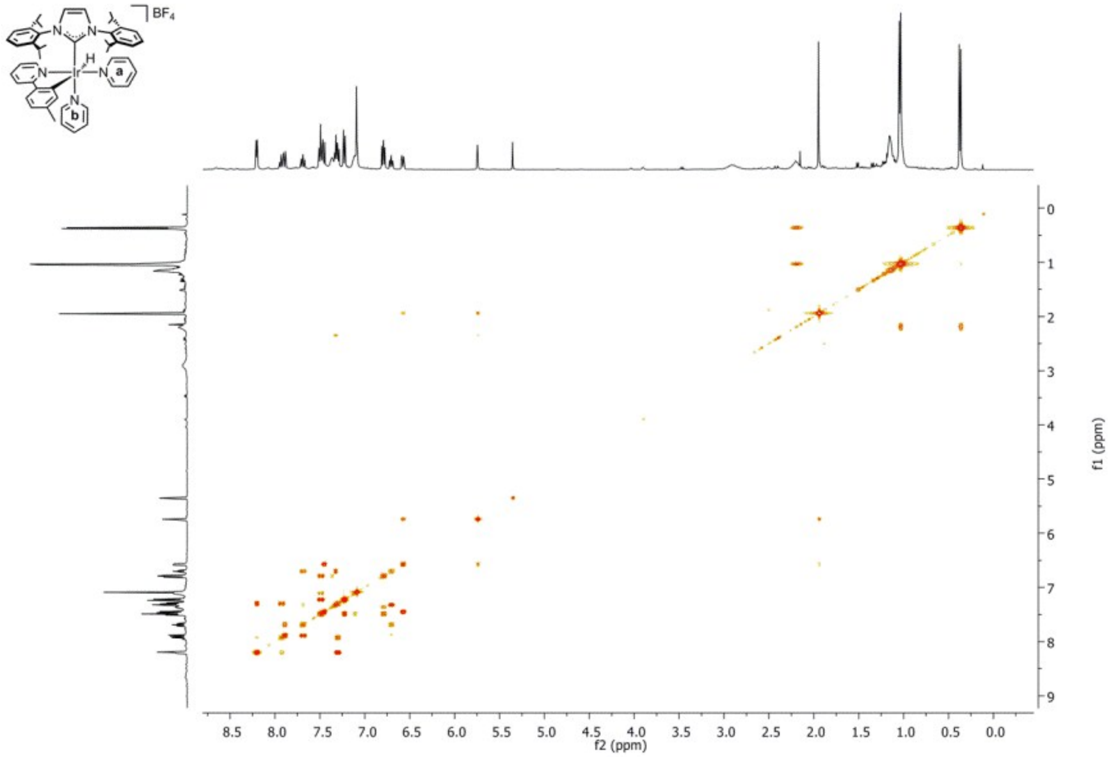

^{1}H-^{13}C HSQC

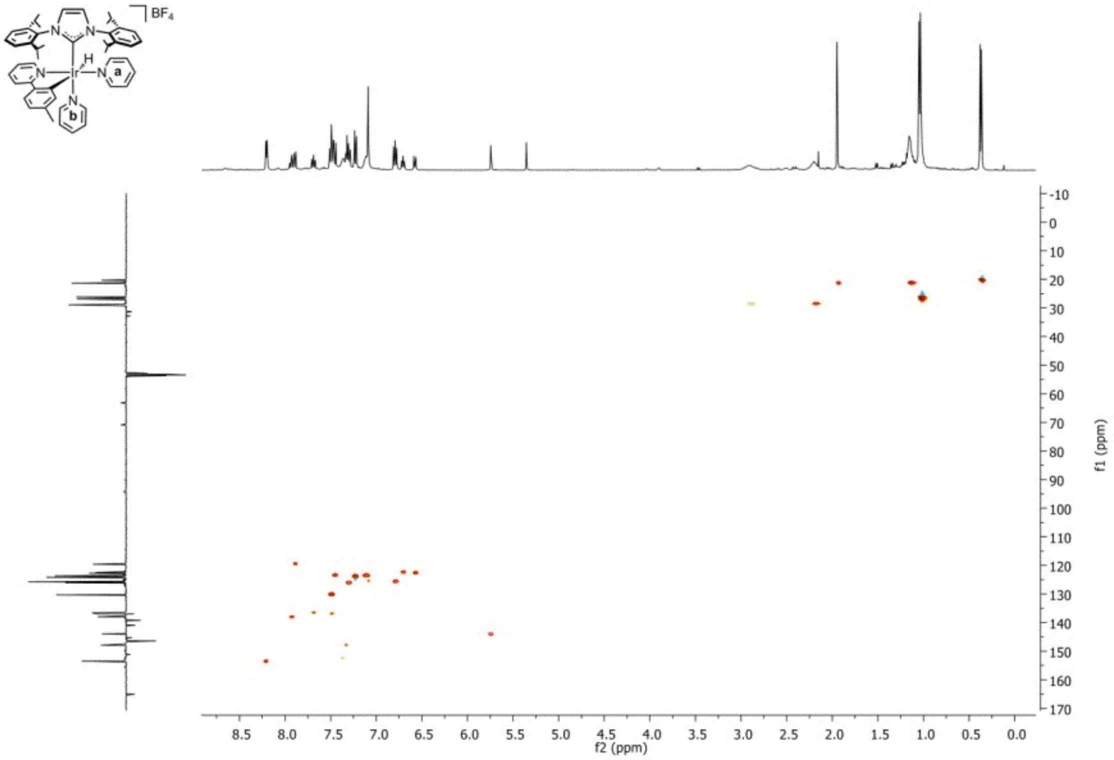

^{1}H-^{13}C HMBC

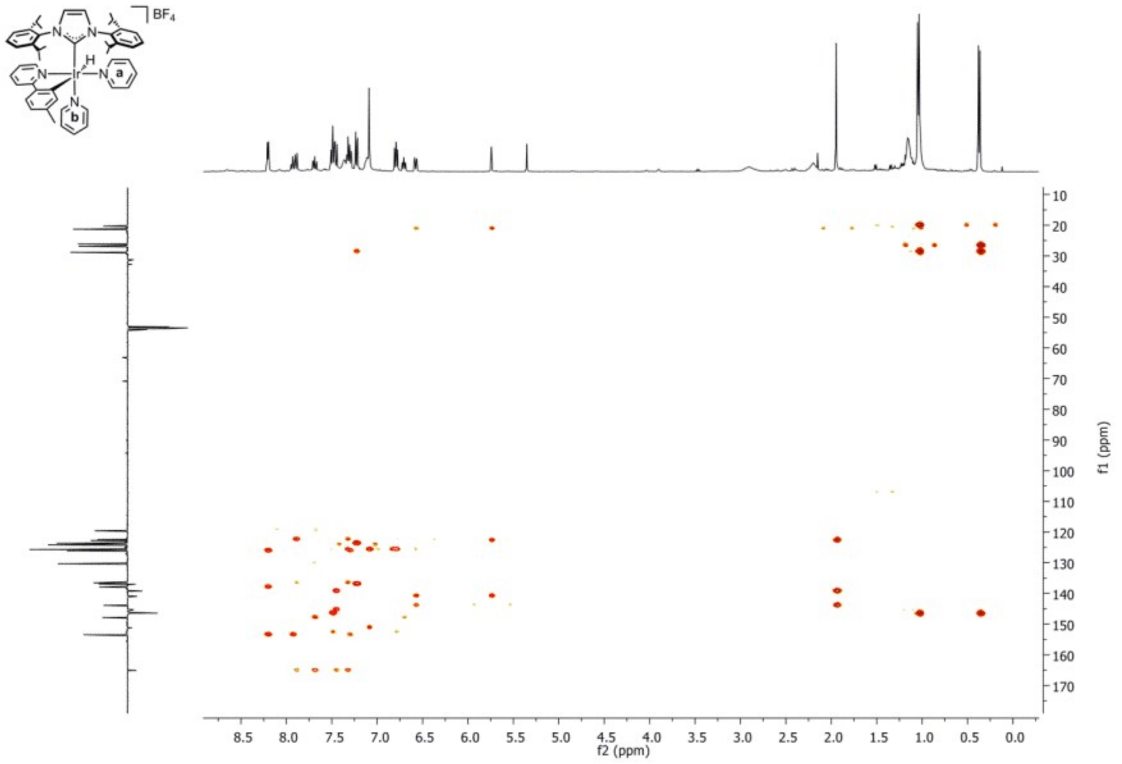

^{19}F NMR

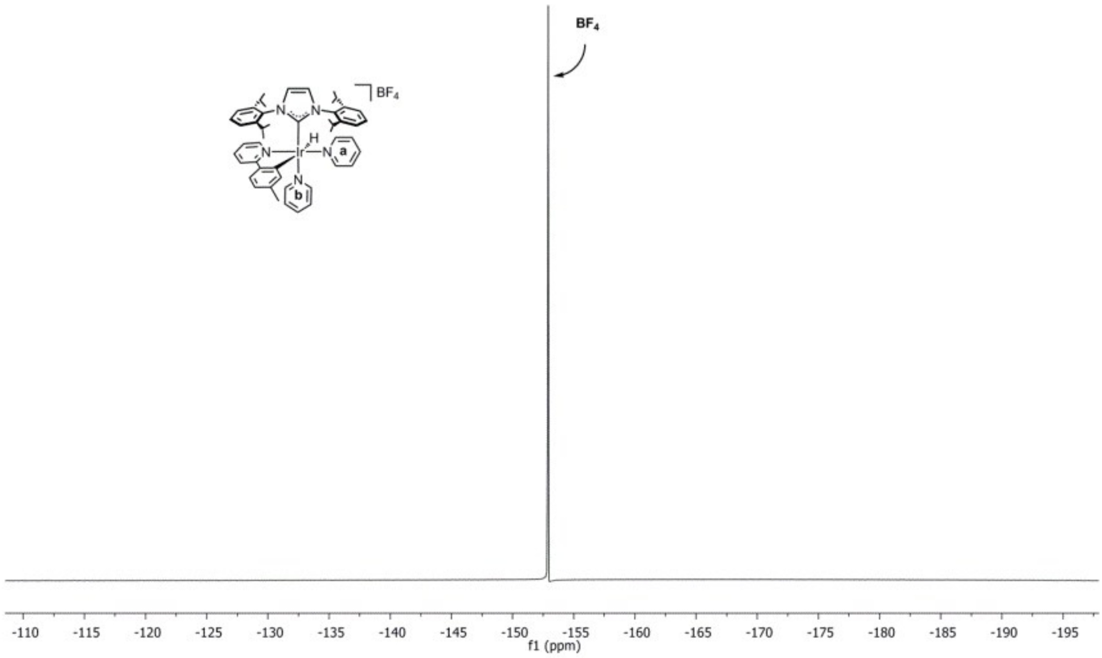

[Ir(H)(IPr)(Phpz-1H)(py)$_2$][BF$_4$] (**5**)

^{1}H NMR

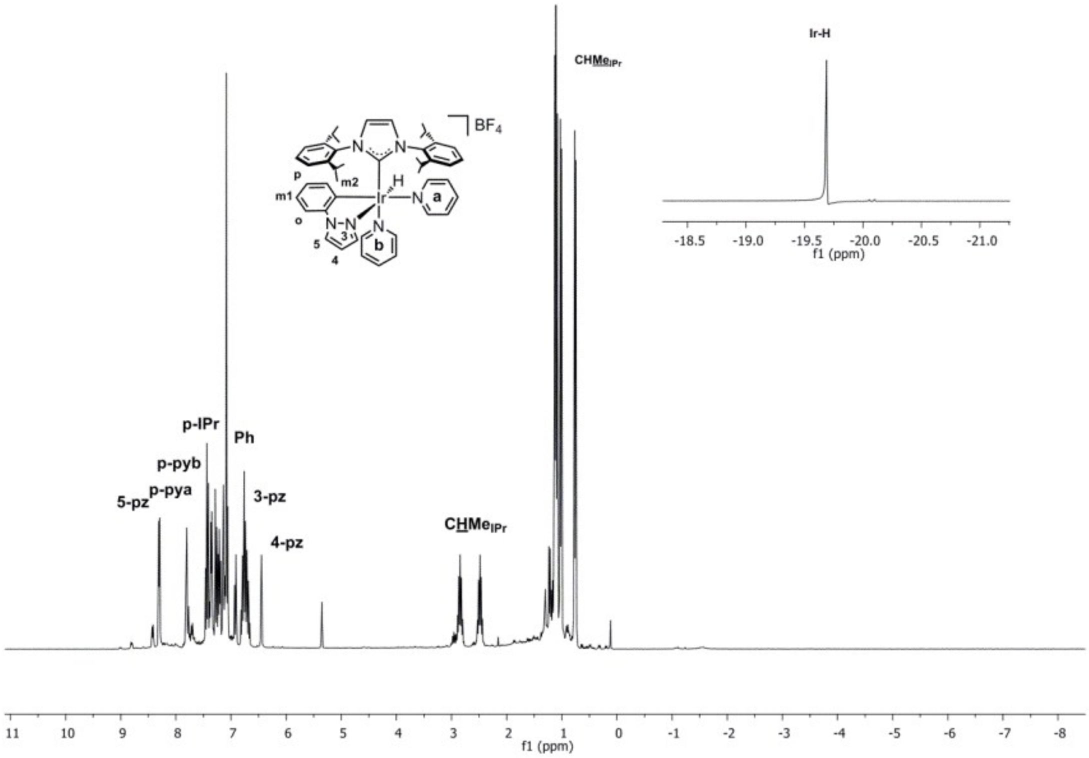

^{13}C NMR

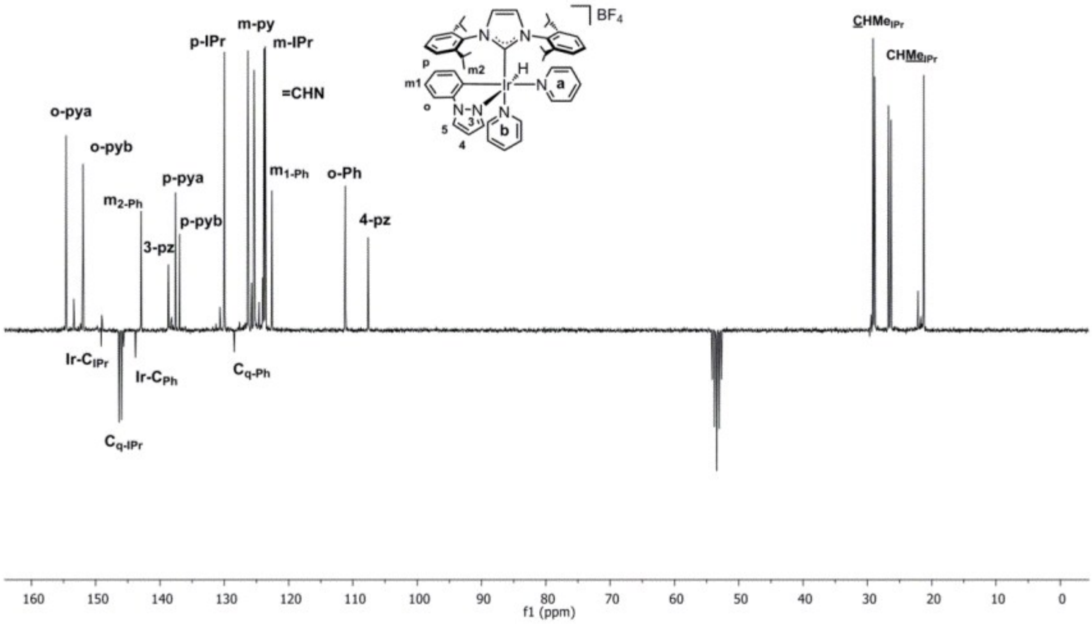

^{1}H-^{1}H COSY

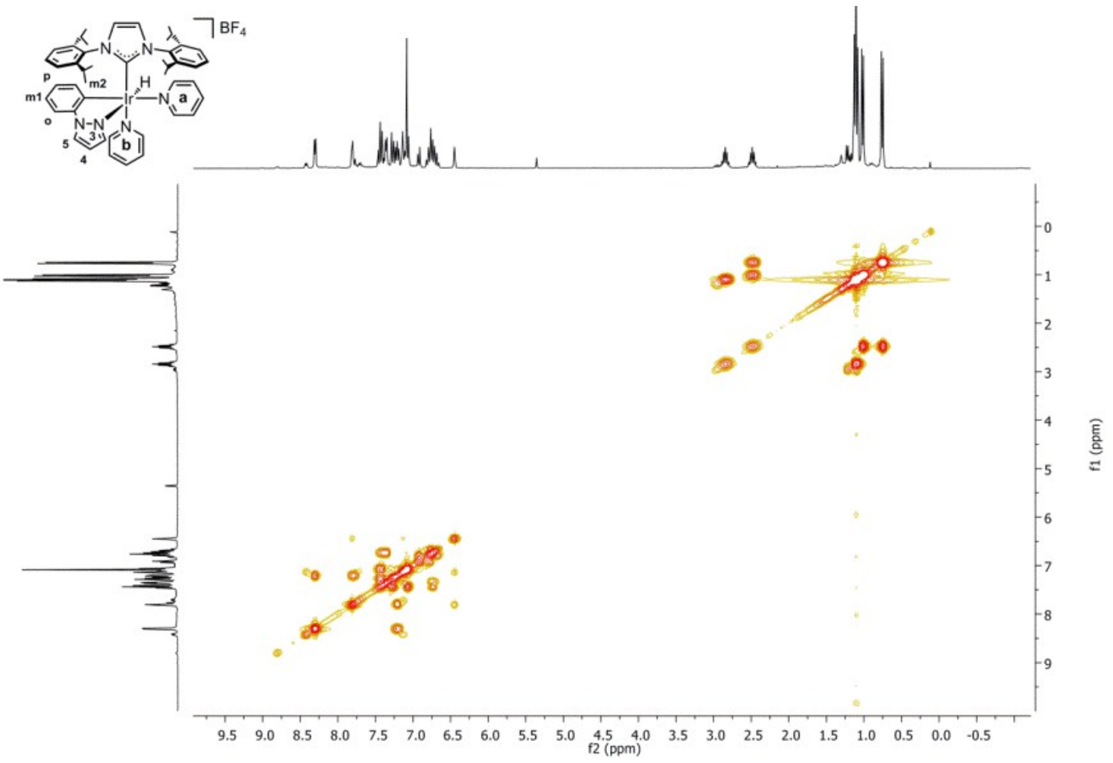

^{1}H-^{13}C HSQC

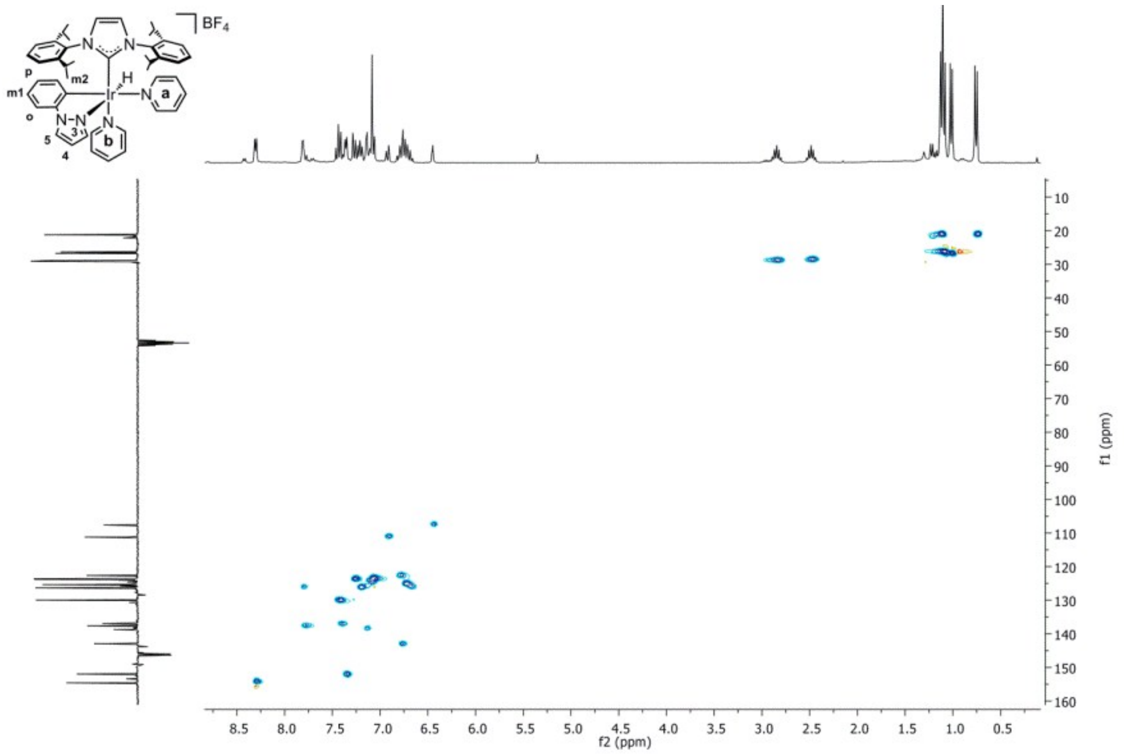

^{1}H-^{13}C HMBC

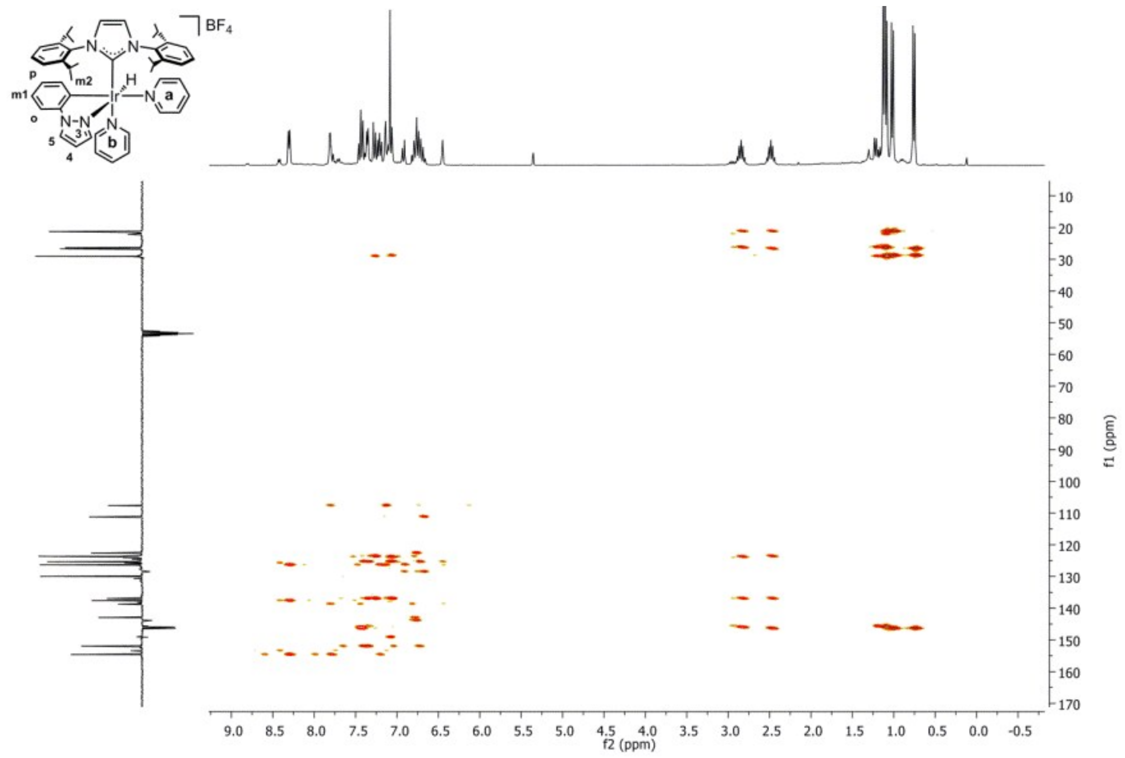

[Ir(bipy)(H)$_2$(IPr)(py)]BF$_4$ (**6**)

^{1}H NMR

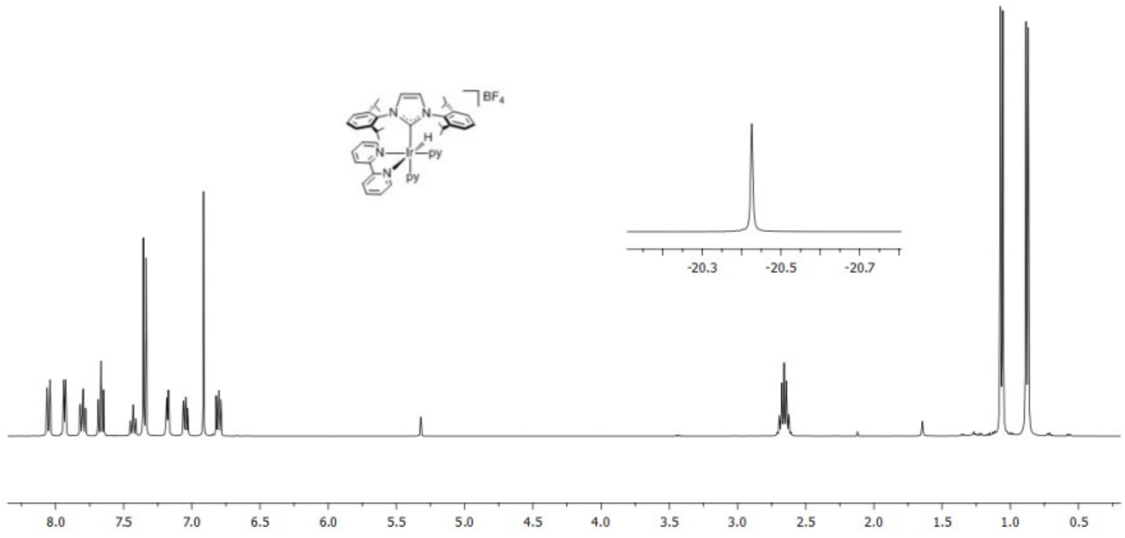

^{13}C NMR

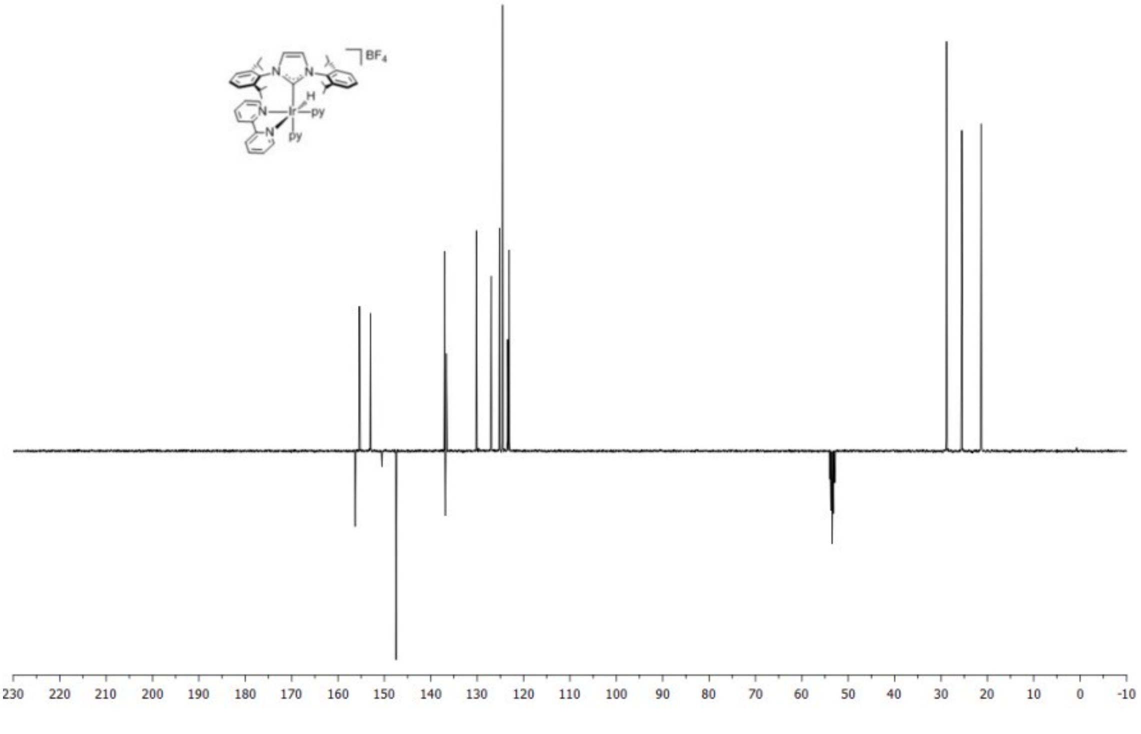

^{1}H-^{1}H COSY

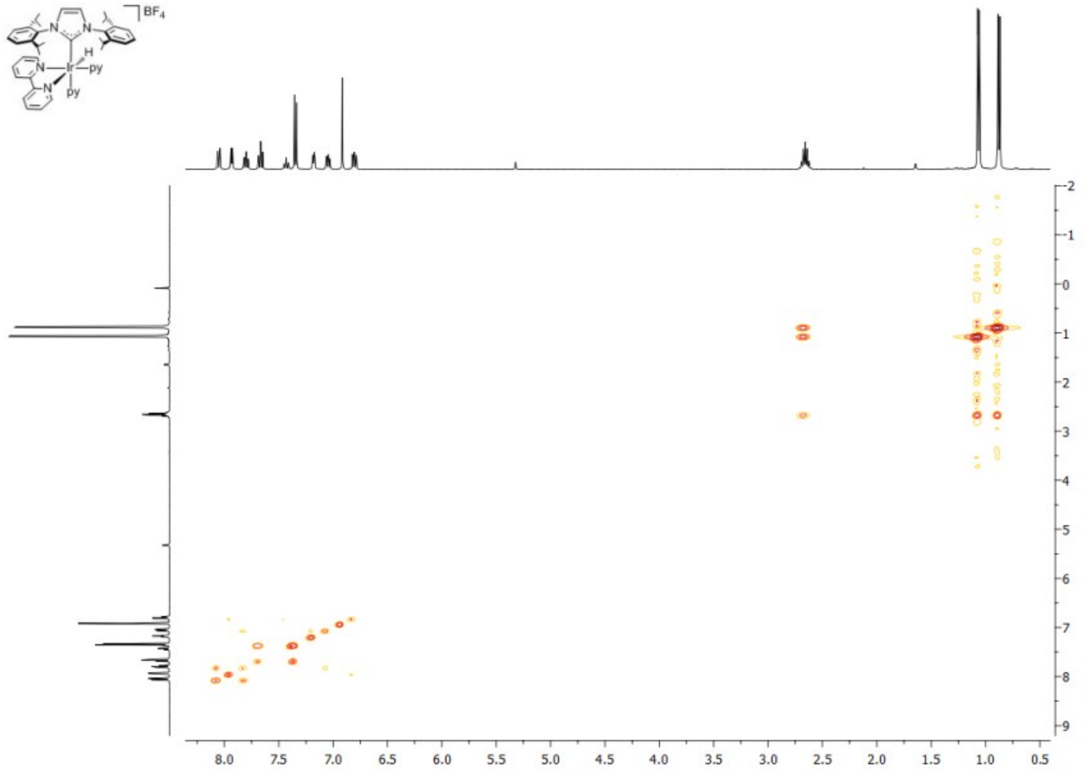

^{1}H-^{13}C HSQC

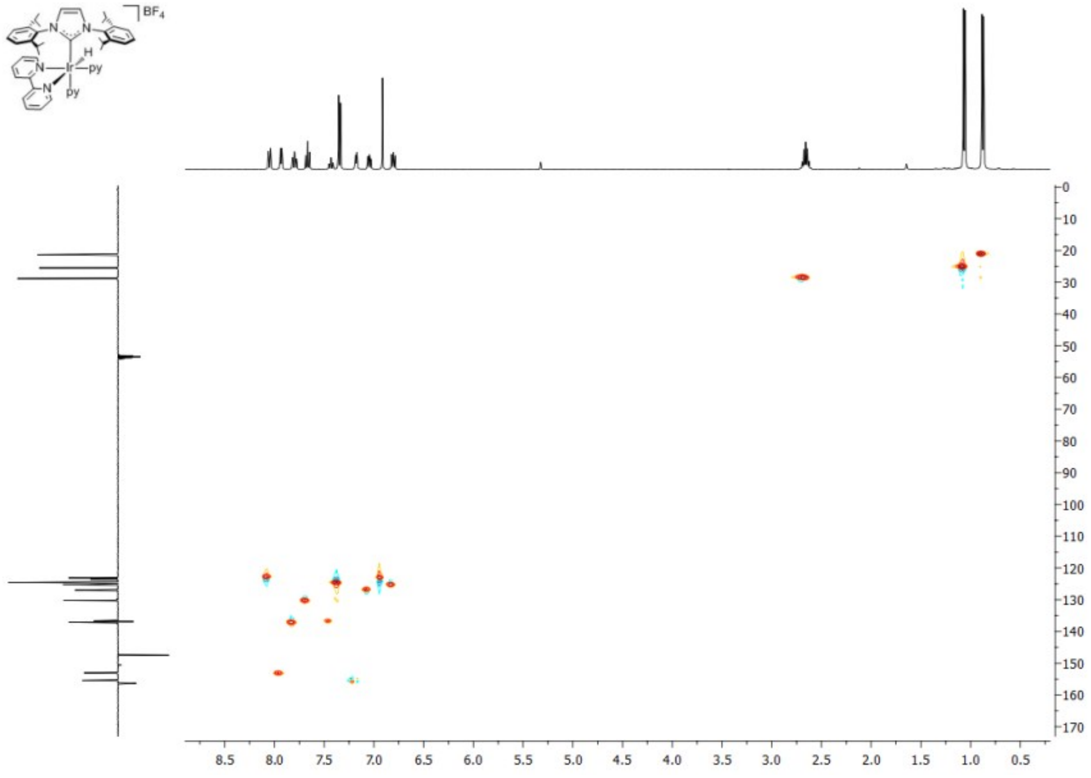

^{1}H-^{13}C HMBC

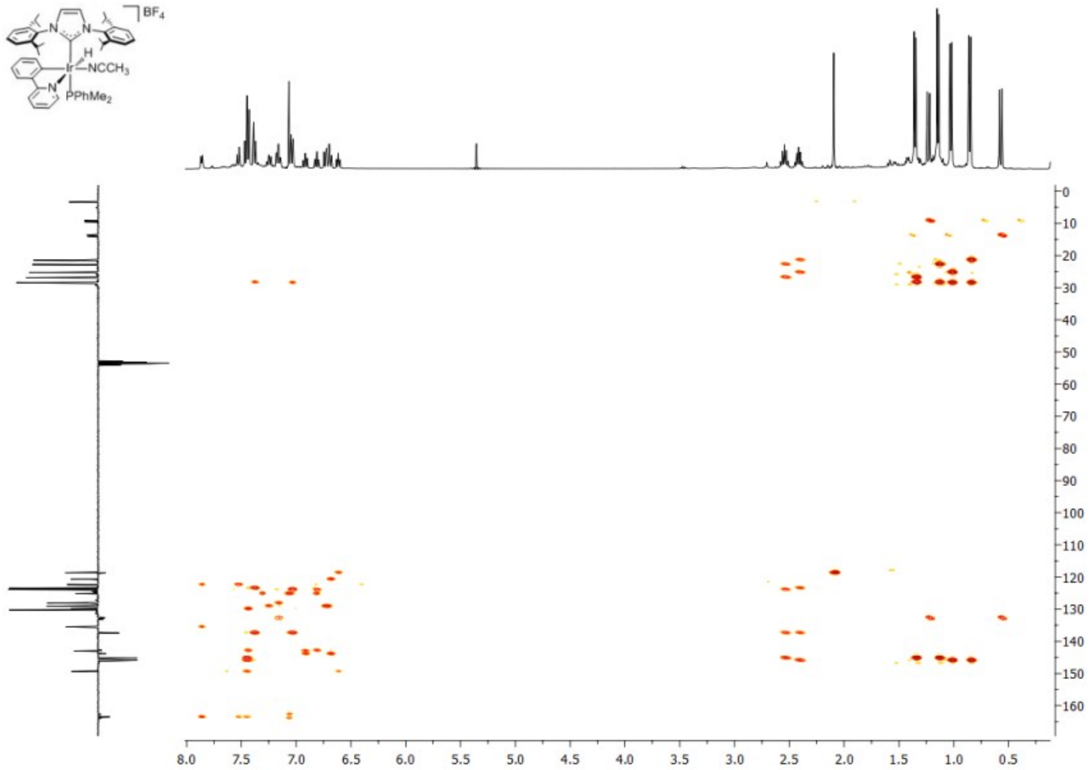

^{19}F NMR

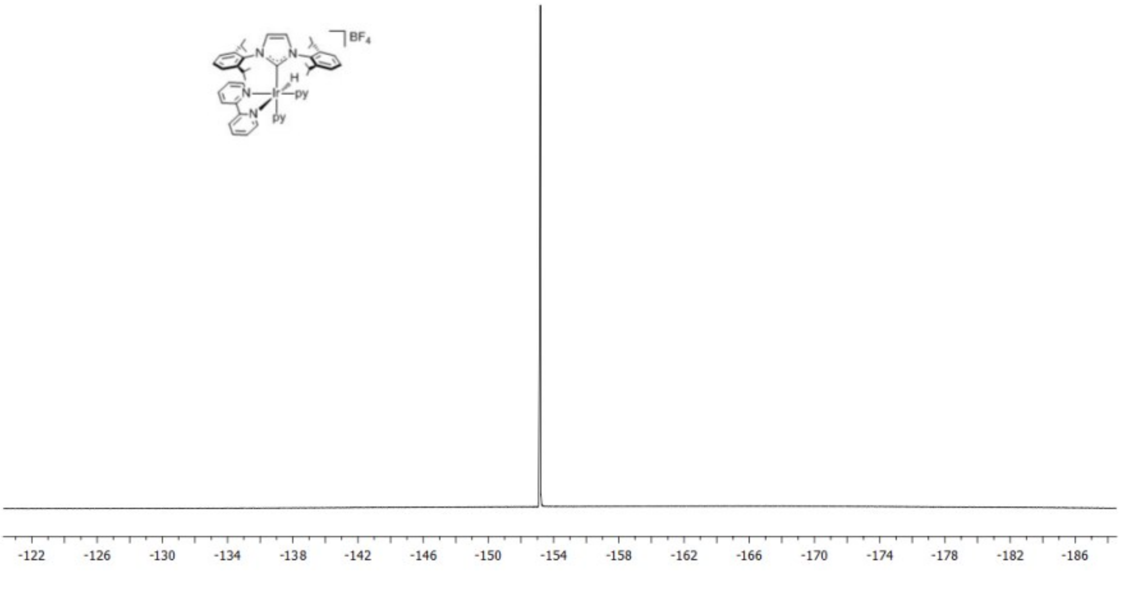

[Ir(CH_3CN)(H)(IPr)(Phpy-1H)($PPhMe_2$)][BF_4] (**7**)

^{1}H NMR

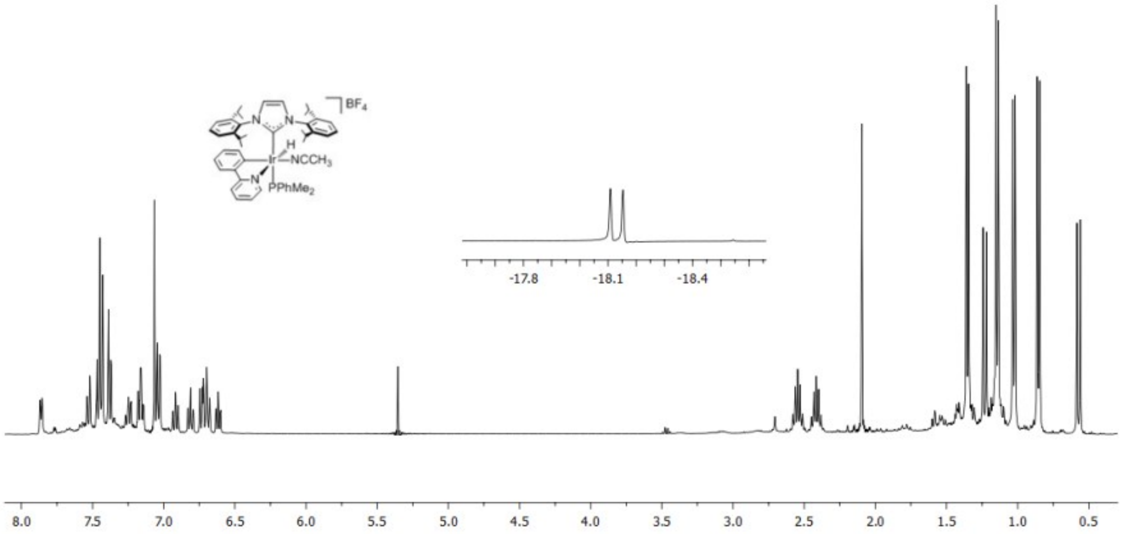

^{13}C NMR

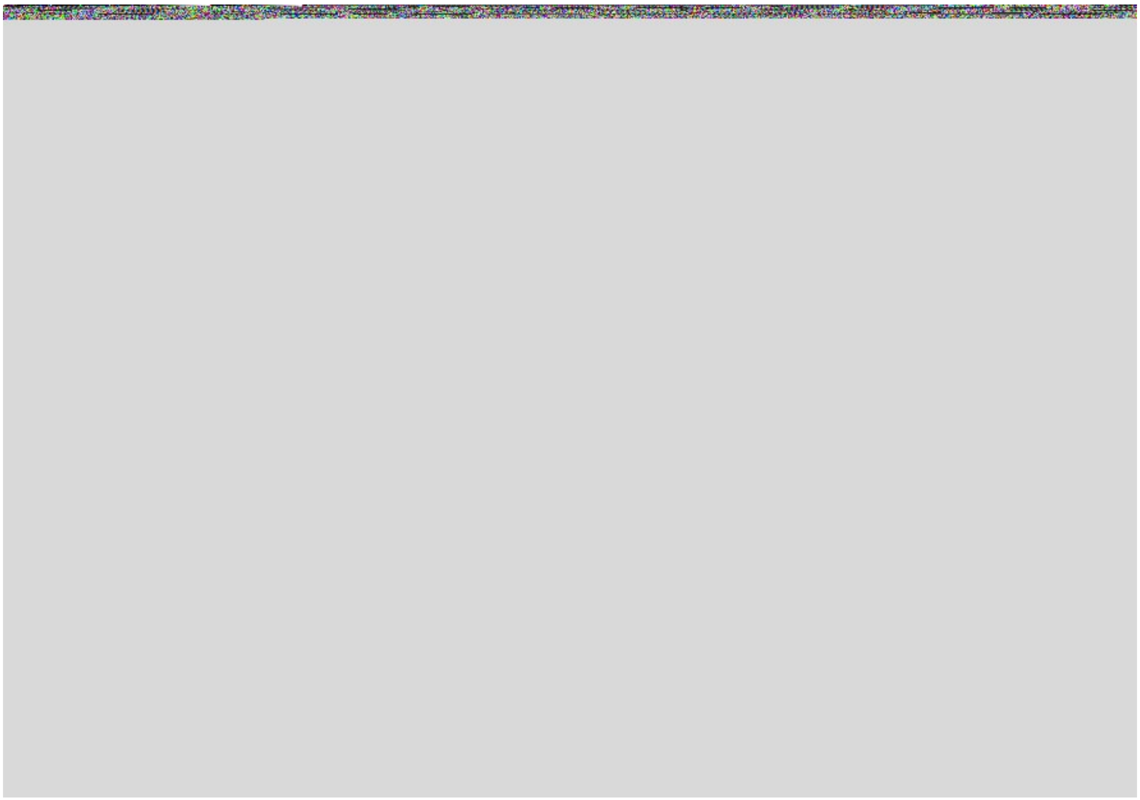

^{1}H-^{1}H COSY

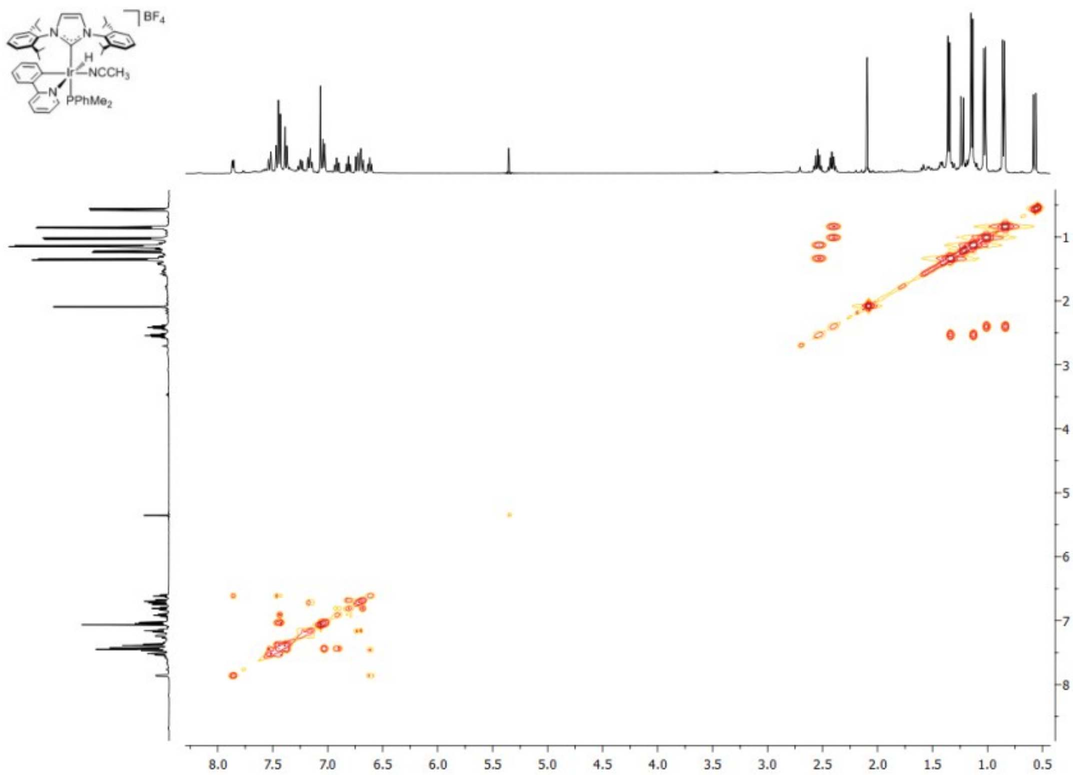

^{1}H-^{13}C HSQC

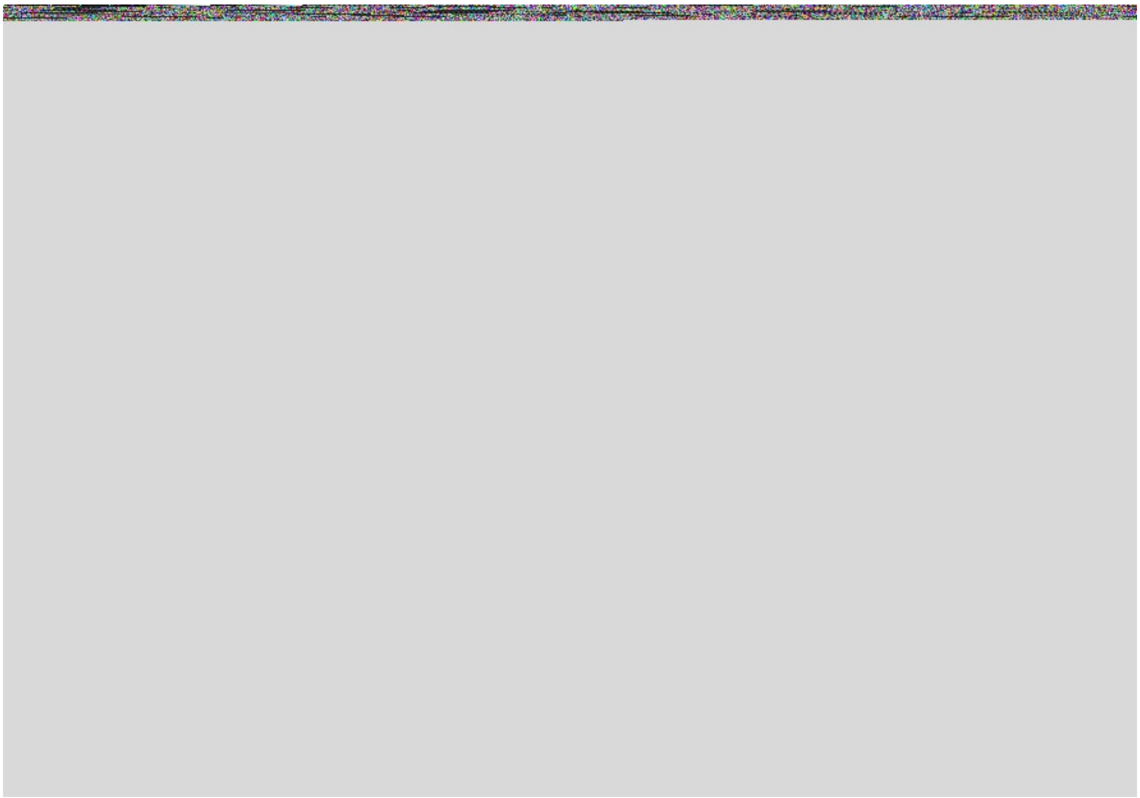

^{1}H-^{13}C HMBC

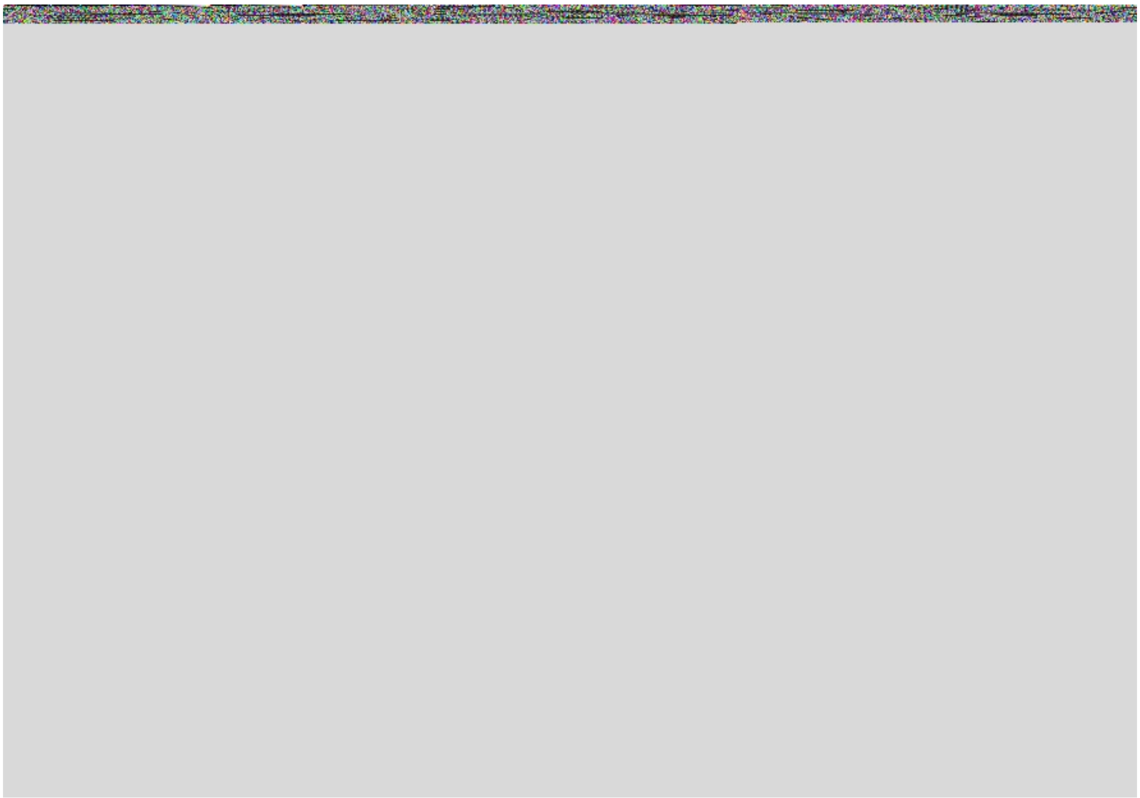

^{31}P NMR

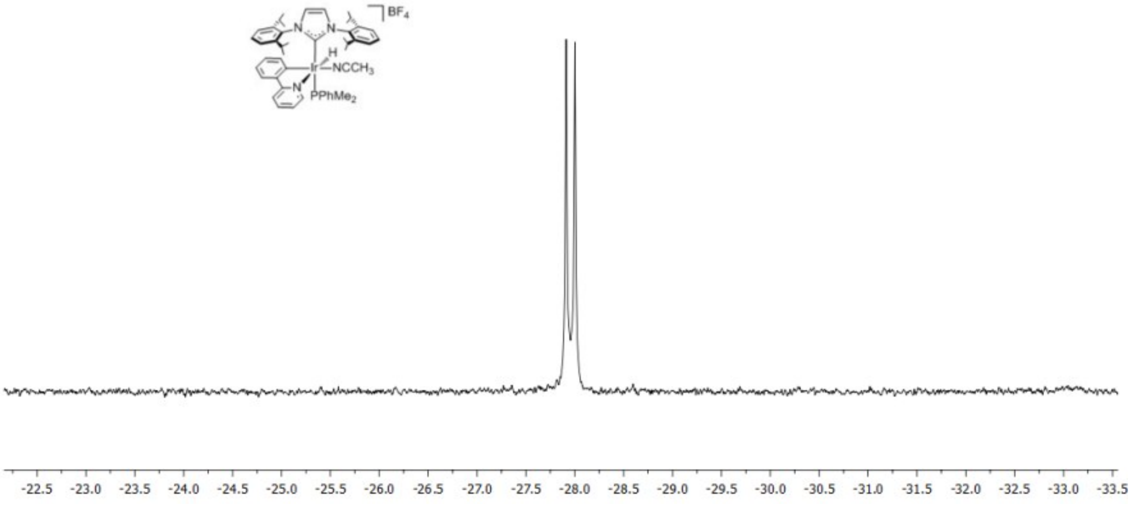

^{19}F NMR

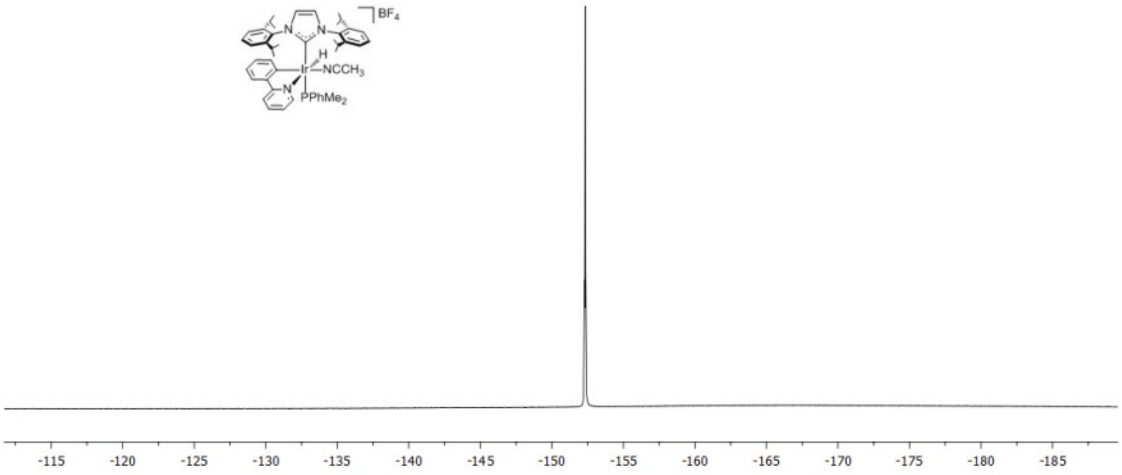
BF4
H
NCCH3
PPhMe2
-115
-120
-125
-130
-135
-140
-145
-150
-155
-160
-165
-170
-175
-180
-185

NMR spectra of organic compounds

2-(3-(triethylsilyl)thiophen-2-yl)pyridine

^{1}H-NMR

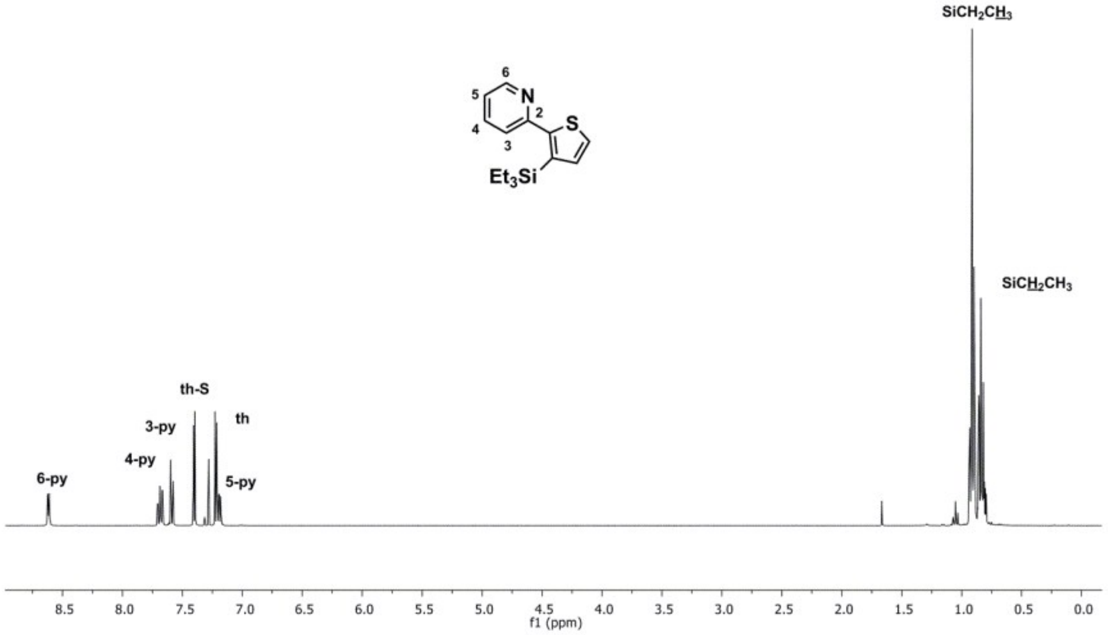

^{13}C NMR

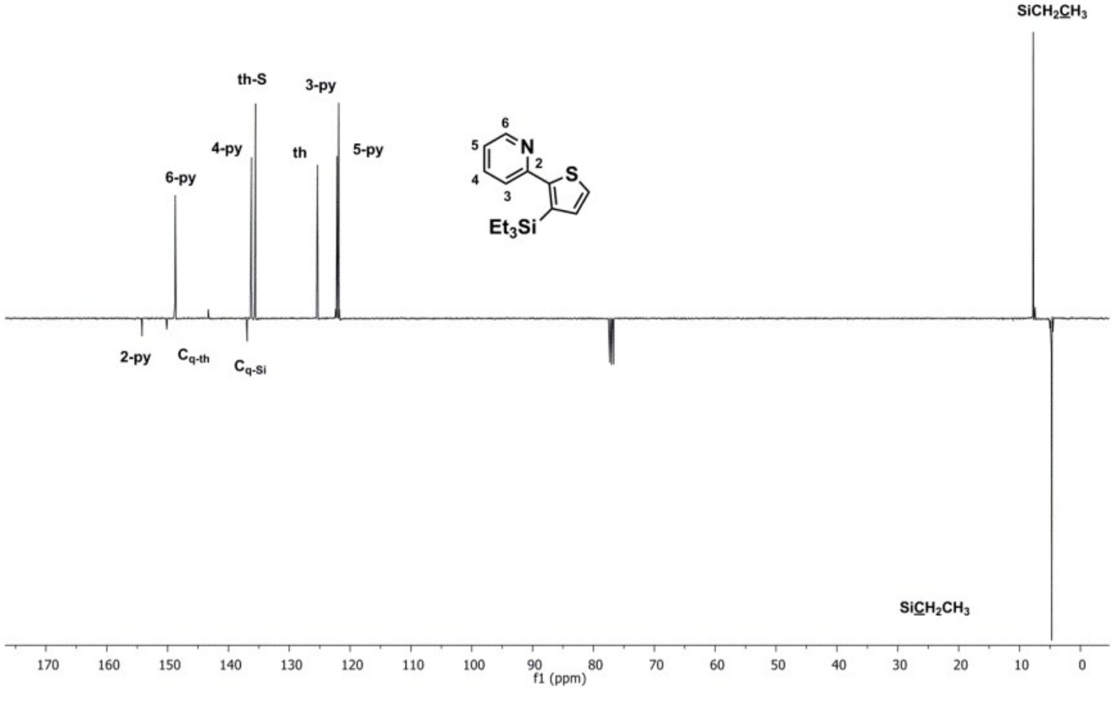

^{1}H-^{1}H COSY

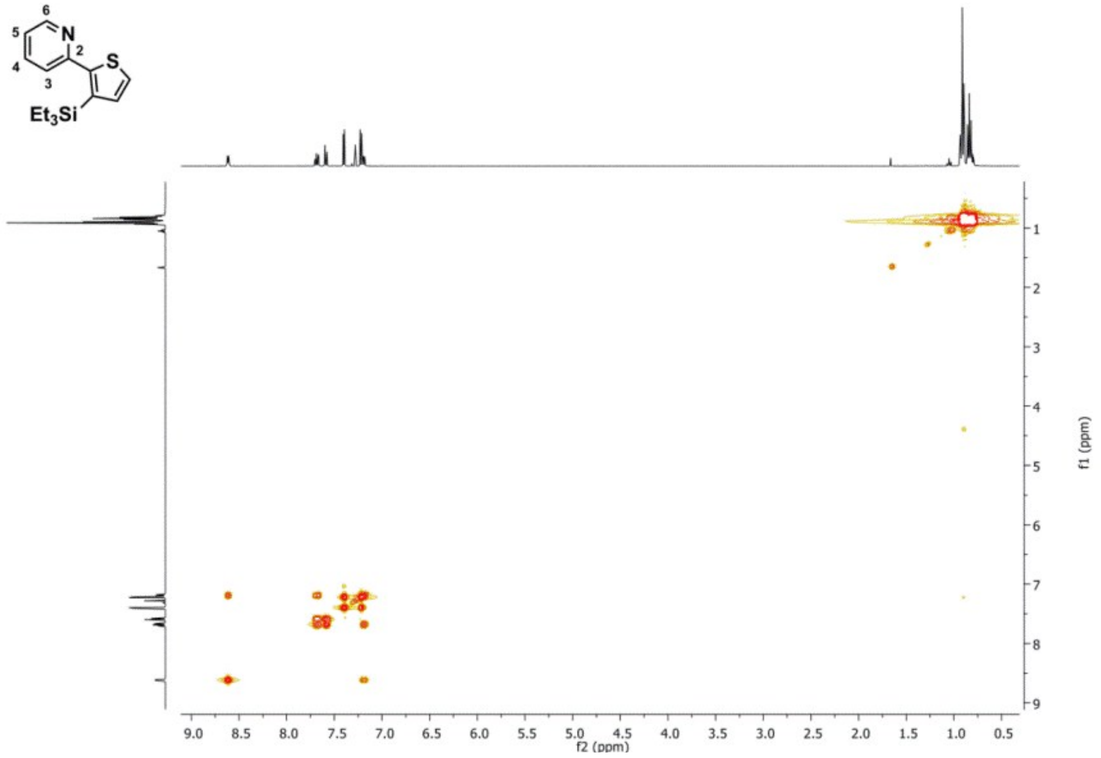

^{1}H-^{13}C HSQC

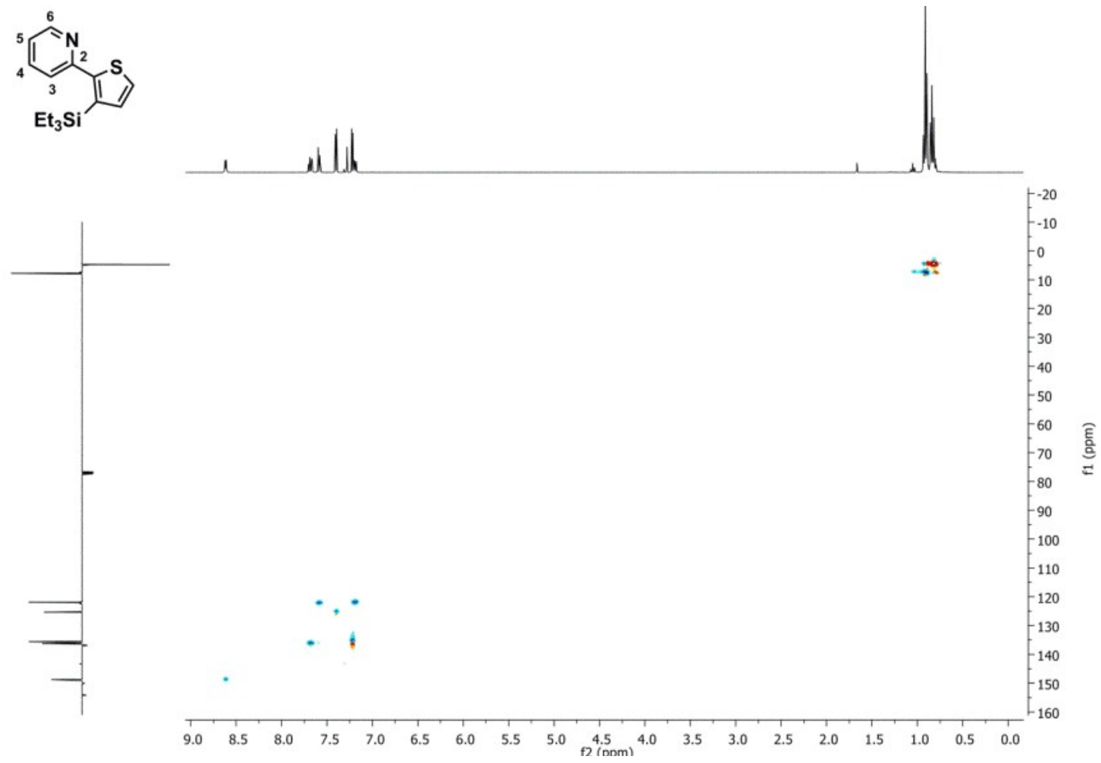

^{1}H-^{13}C HMBC

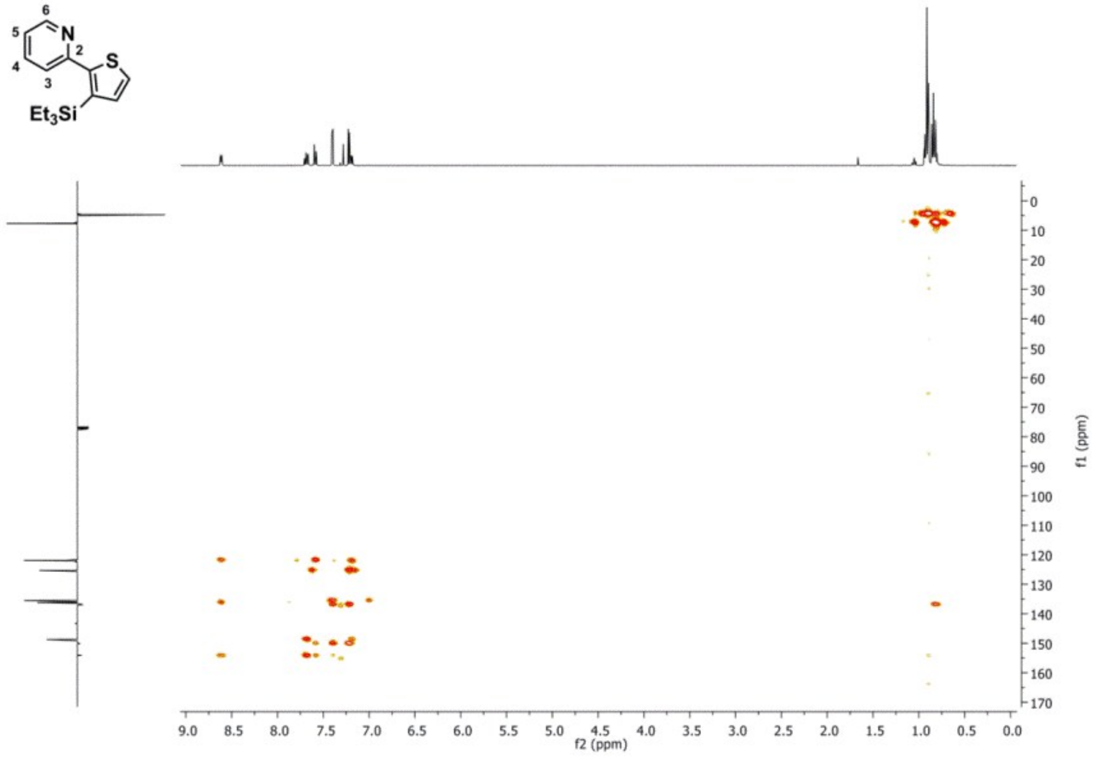

^{1}H-^{29}Si HMBC

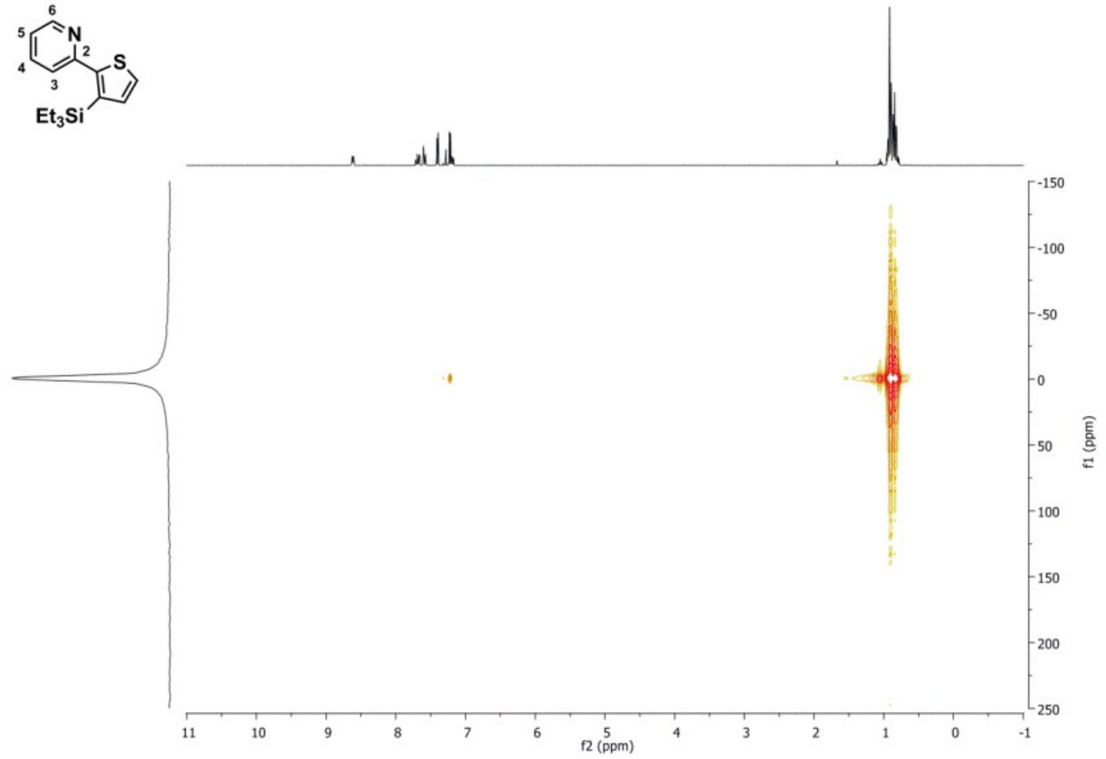

2-(3-(dimethyl(phenyl)silyl)thiophen-2-yl)pyridine

^{1}H NMR

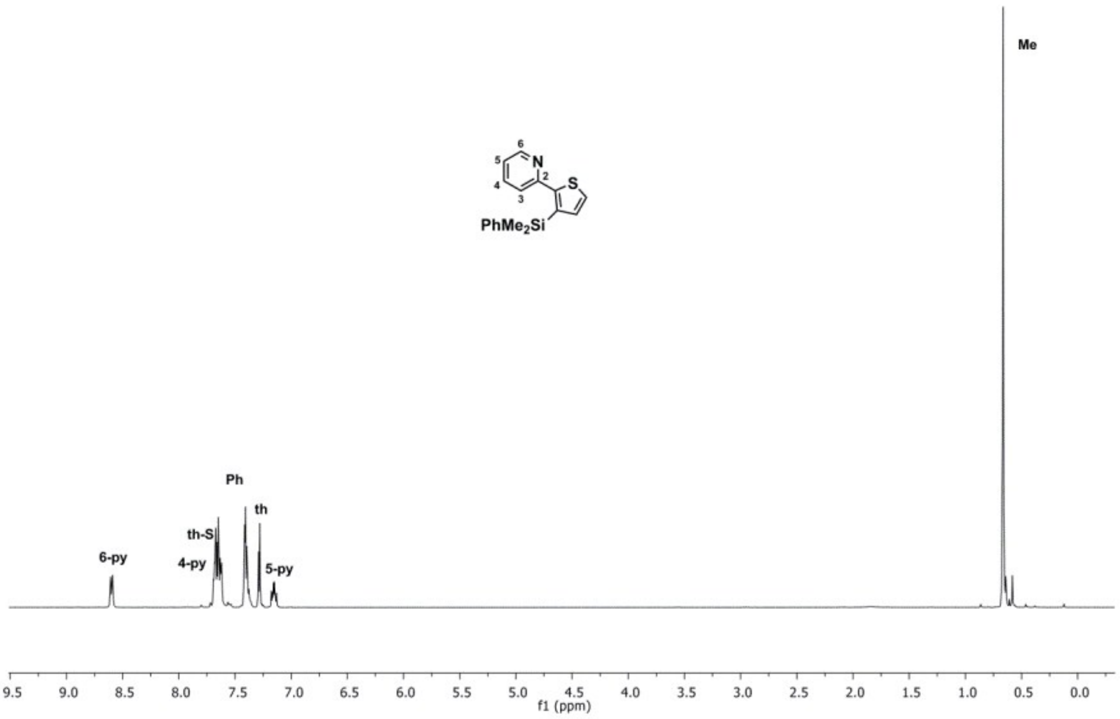

^{13}C NMR

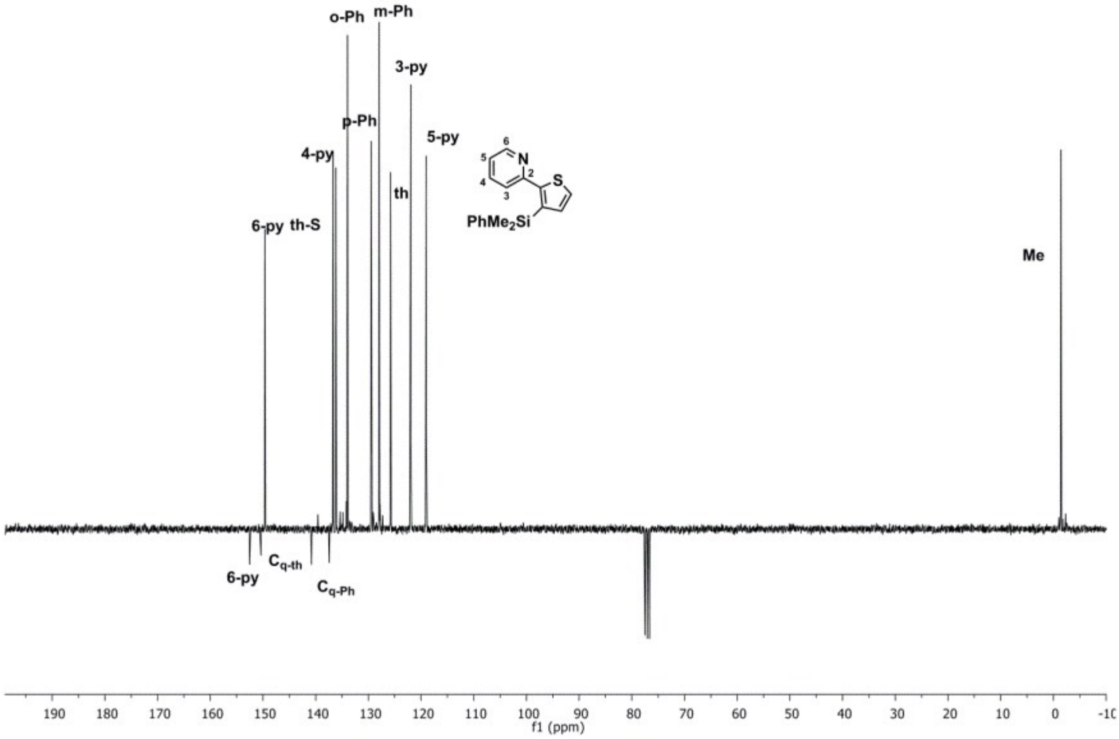

^{1}H-^{29}Si HMBC

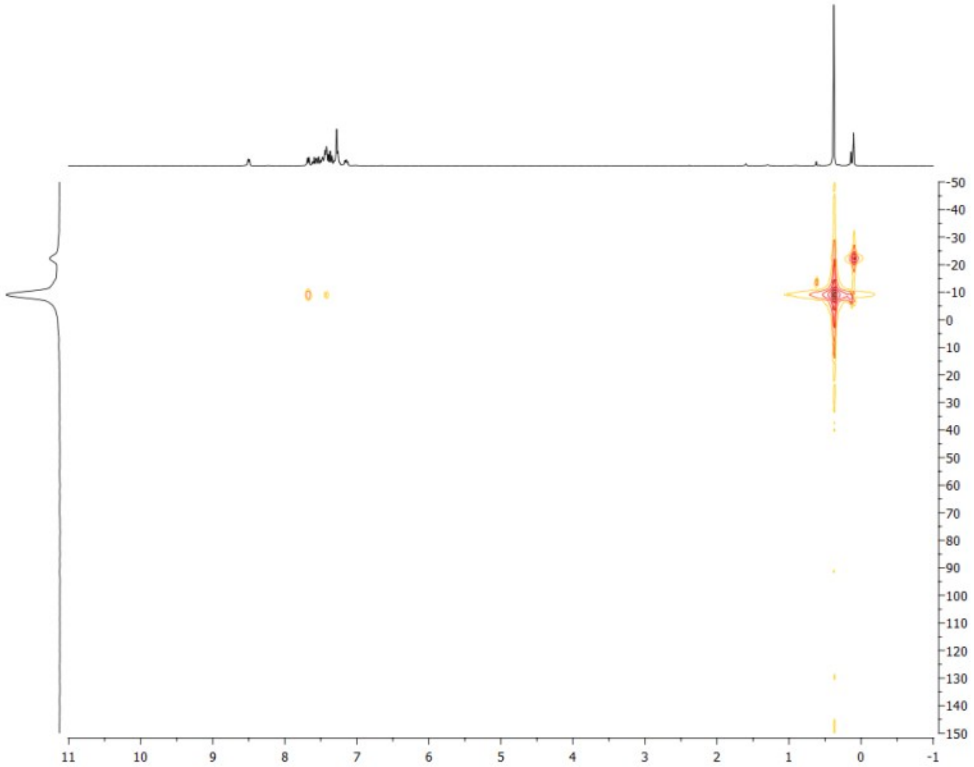

2-(3-(methyldiphenylsilyl)thiophen-2-yl)pyridine

^{1}H-NMR

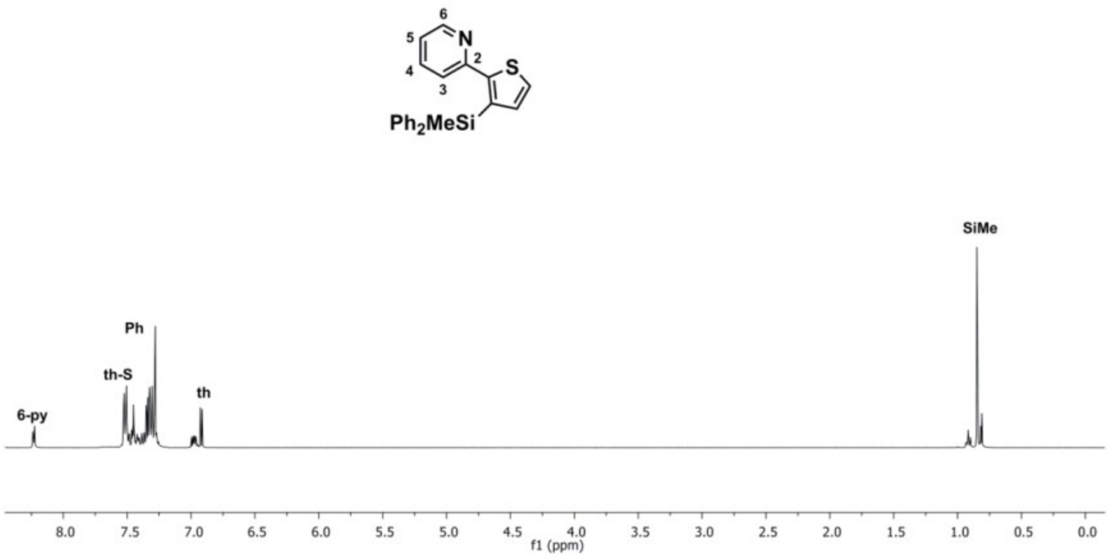

^{13}C NMR

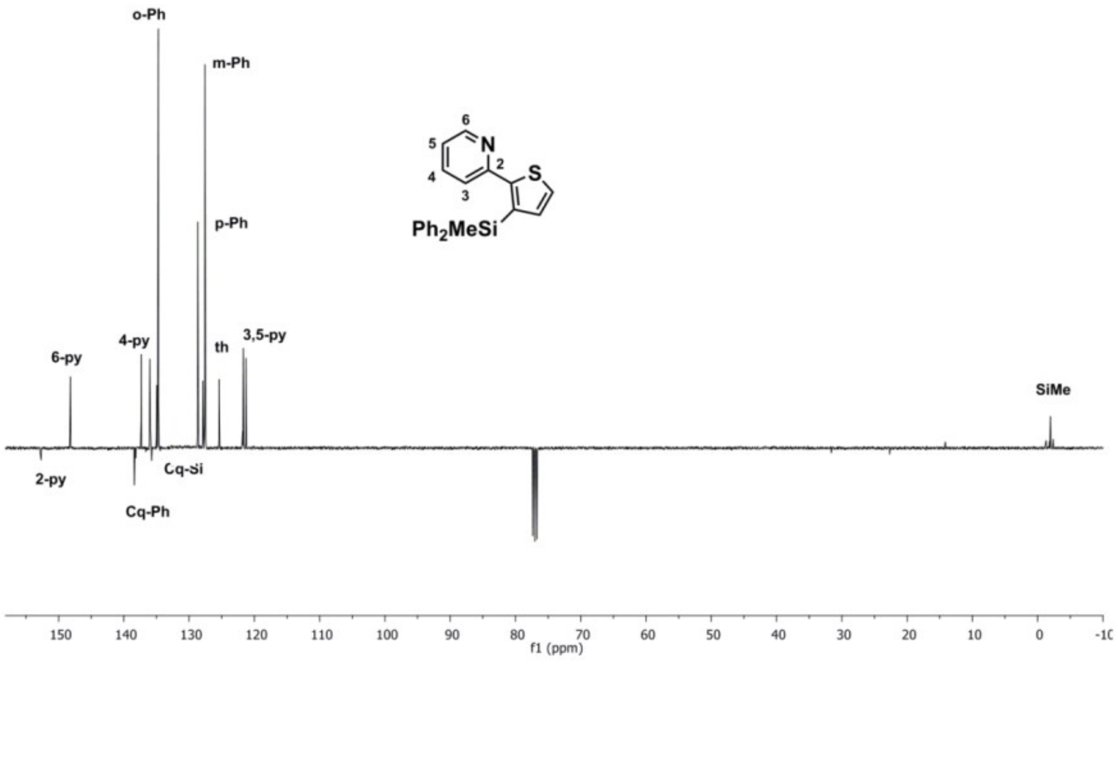

^1H-^{29}Si HMBC

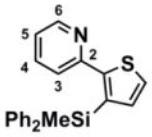

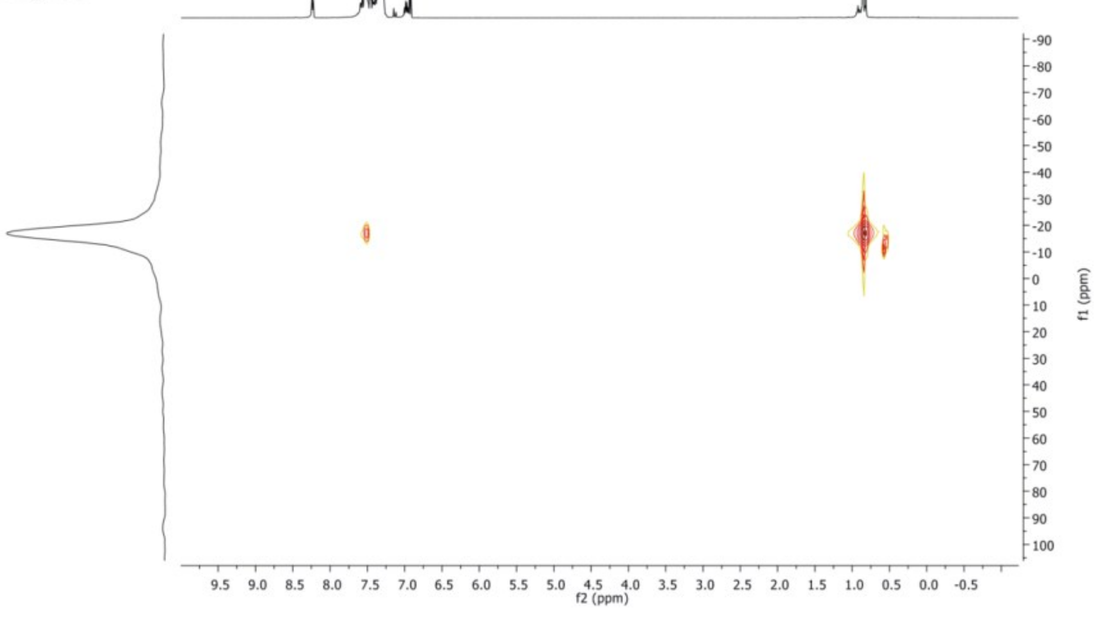

2-(3-(triphenylsilyl)thiophen-2-yl)pyridine

^{1}H-NMR

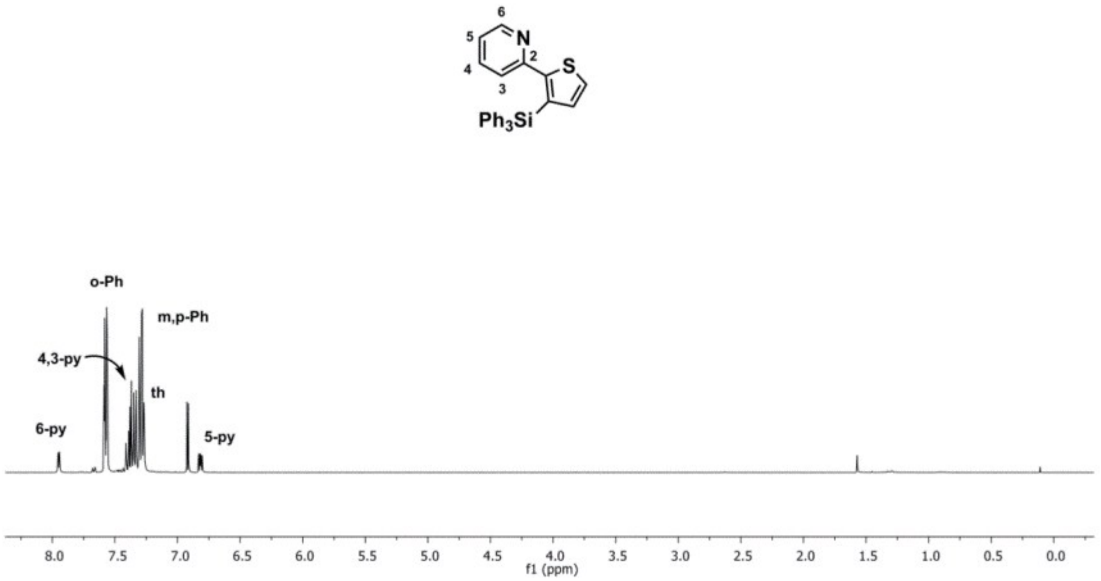

^{13}C NMR

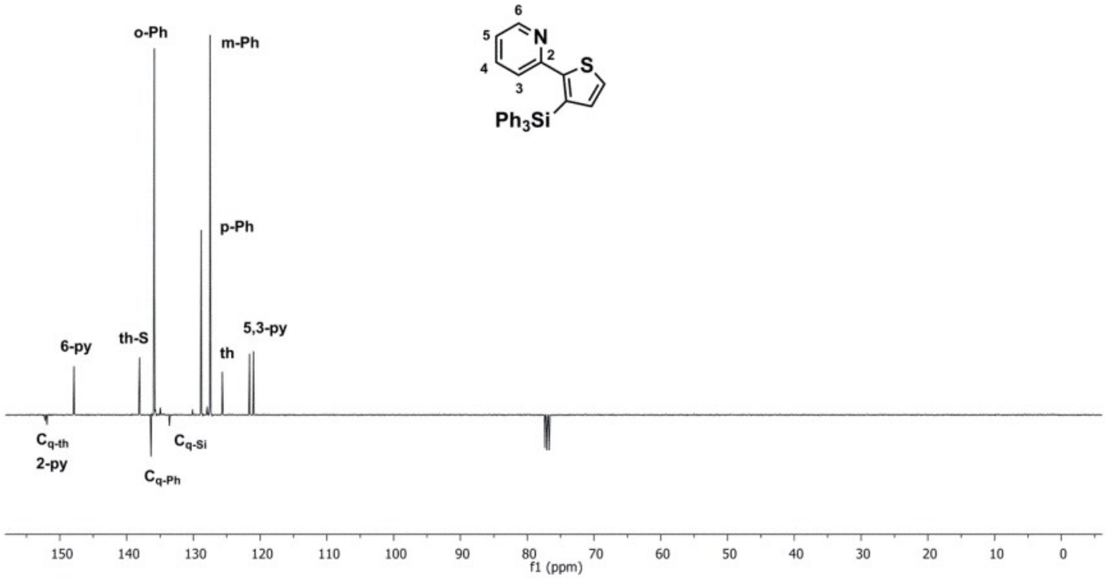

^{1}H-^{29}Si HMBC

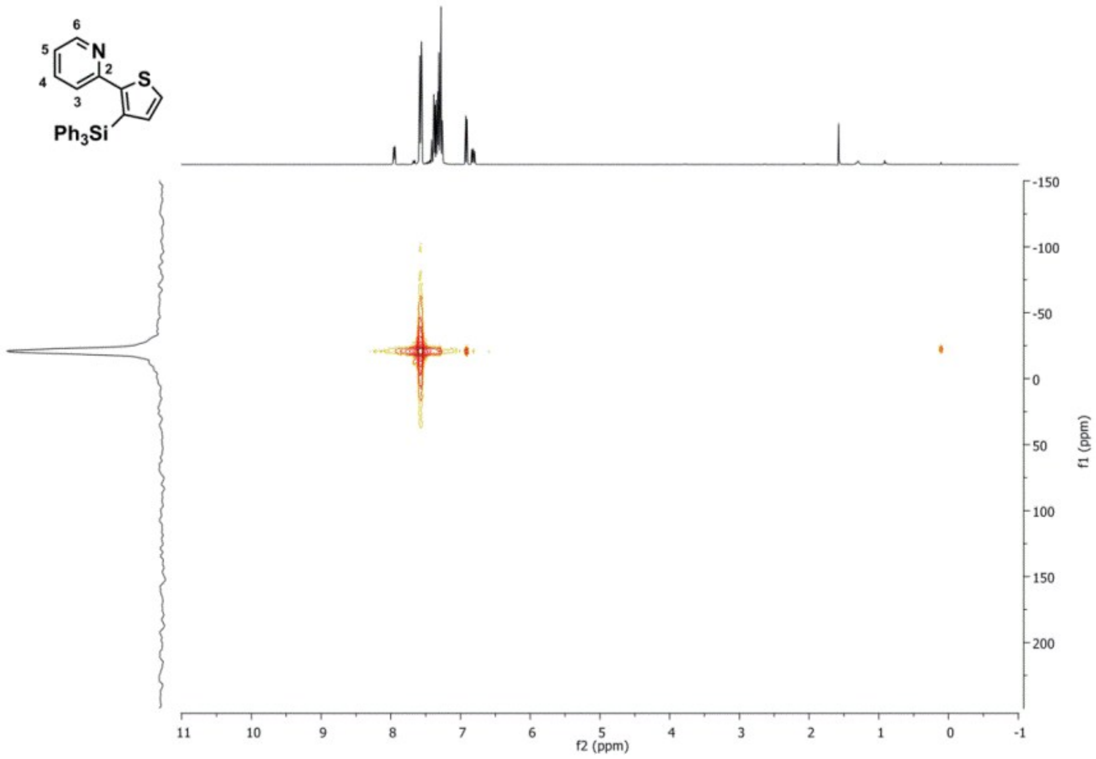
6
5
N
2
S
4
3
Ph3Si
-150
-100
-50
0
50
100
150
200
f1 (ppm)
11
10
9
8
7
6
5
4
3
2
1
0
-1
f2 (ppm)

Triethyl(2-(pyridin-2-yl)thiophen-3-yl)-l6-silanone

^{1}H NMR

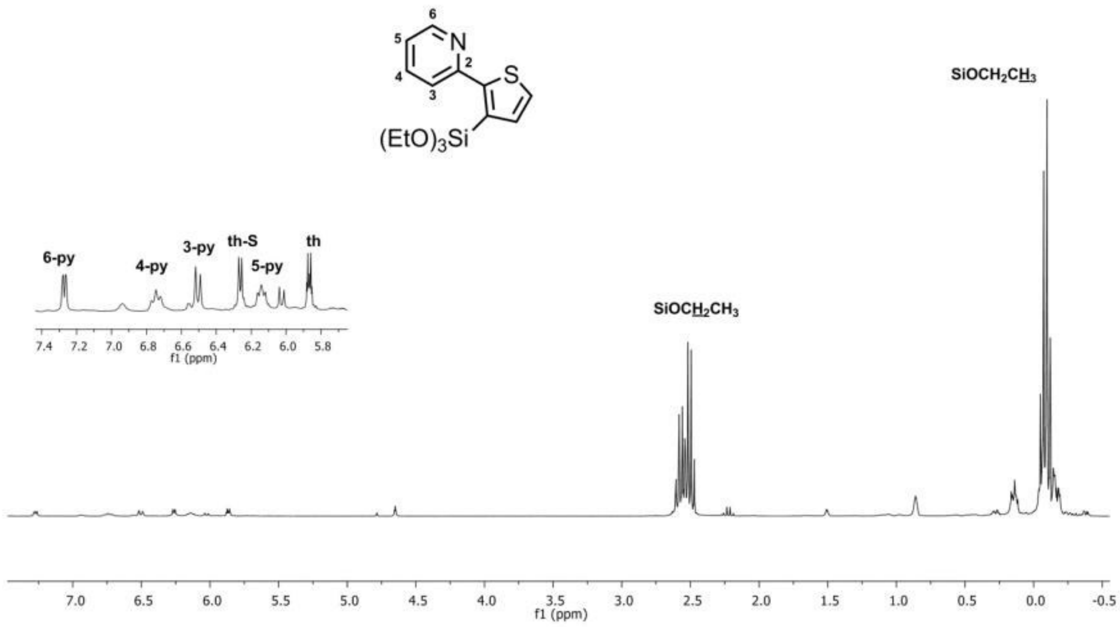

^{13}C NMR

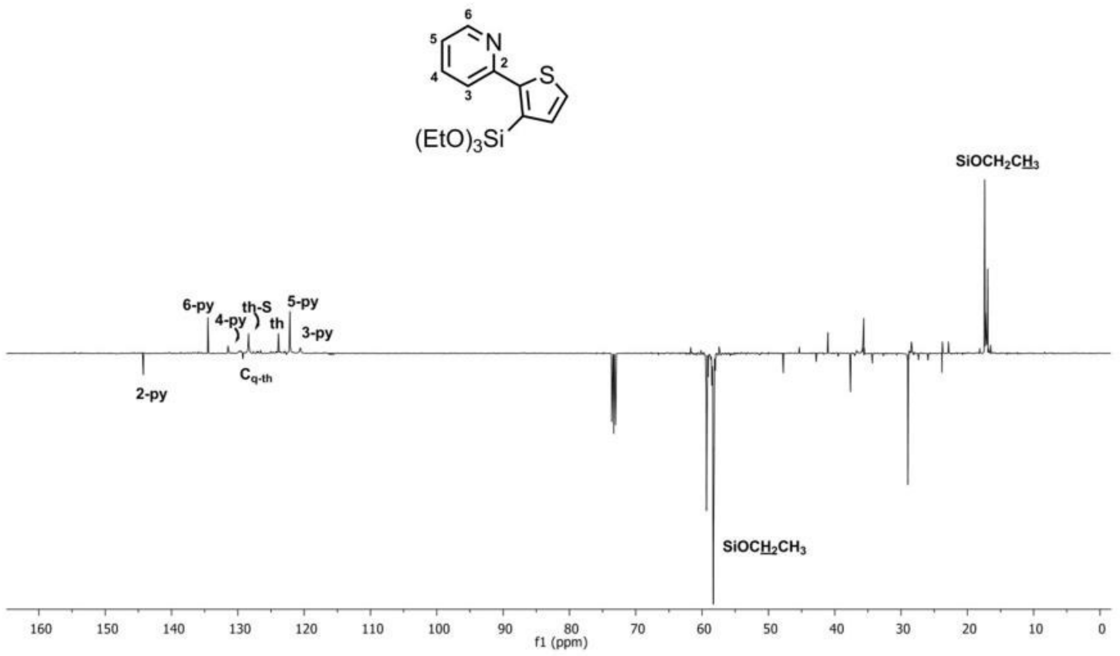

^1H-^1H COSY

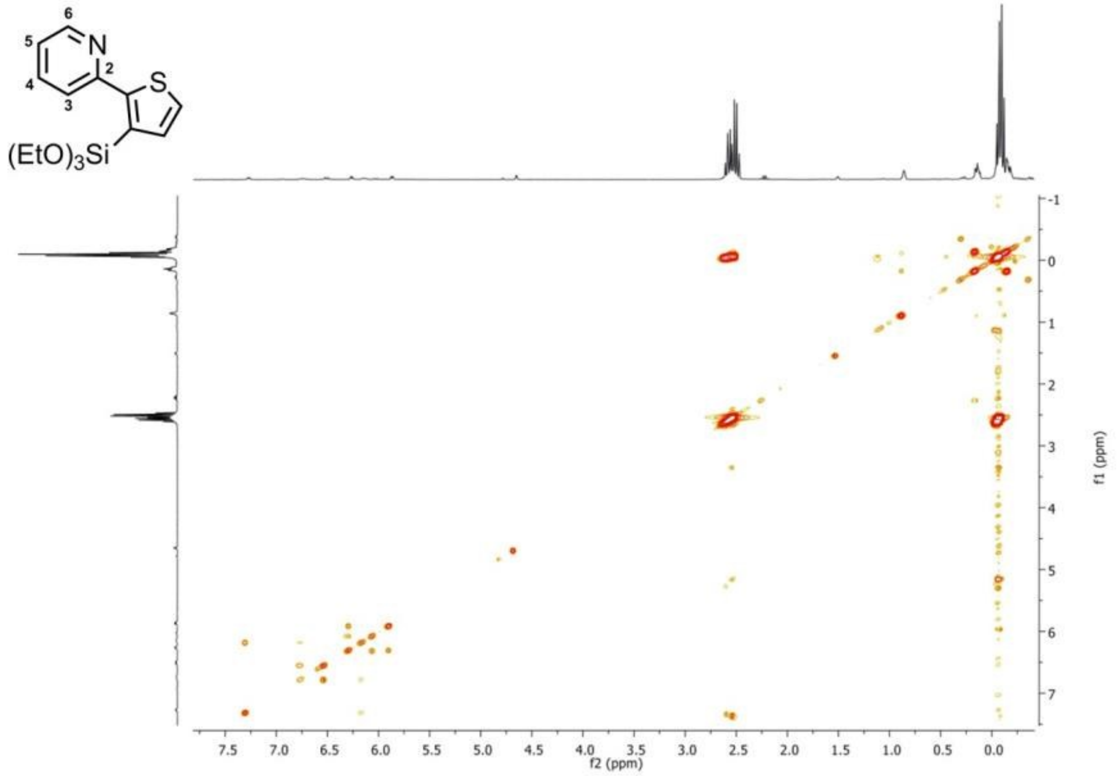

^1H-^{29}Si HMBC

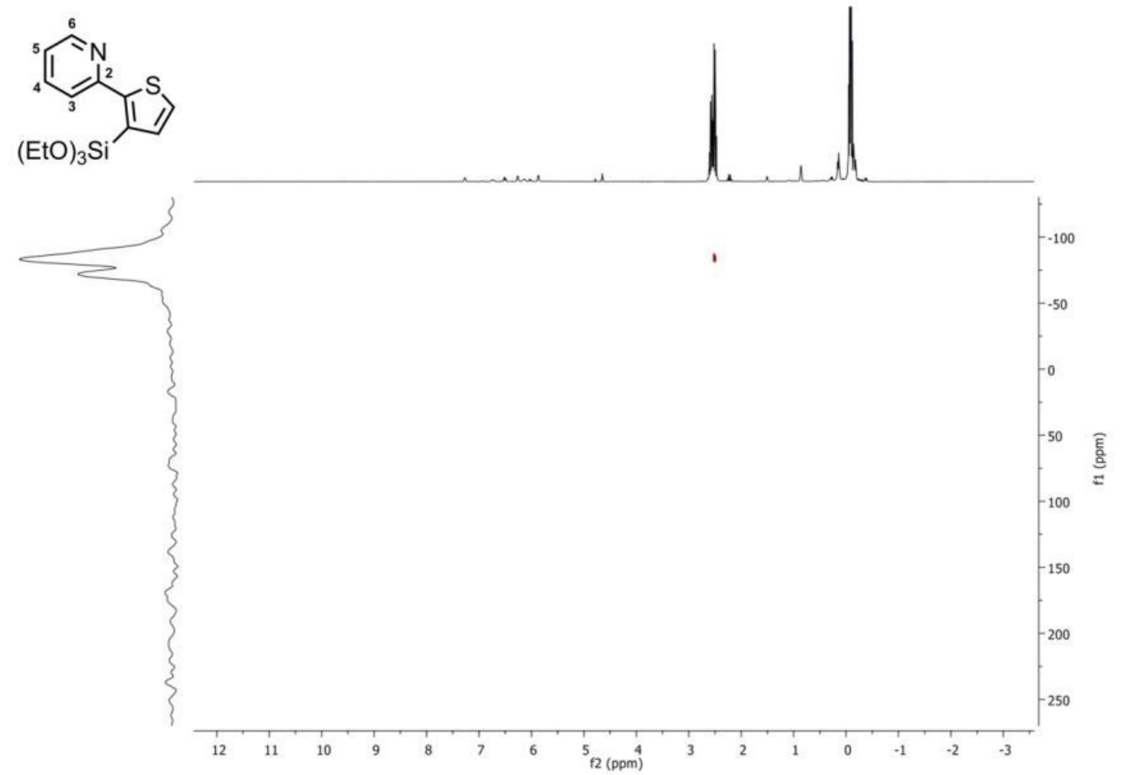

2-(2-(triethylsilyl)phenyl)pyridine

^{1}H NMR

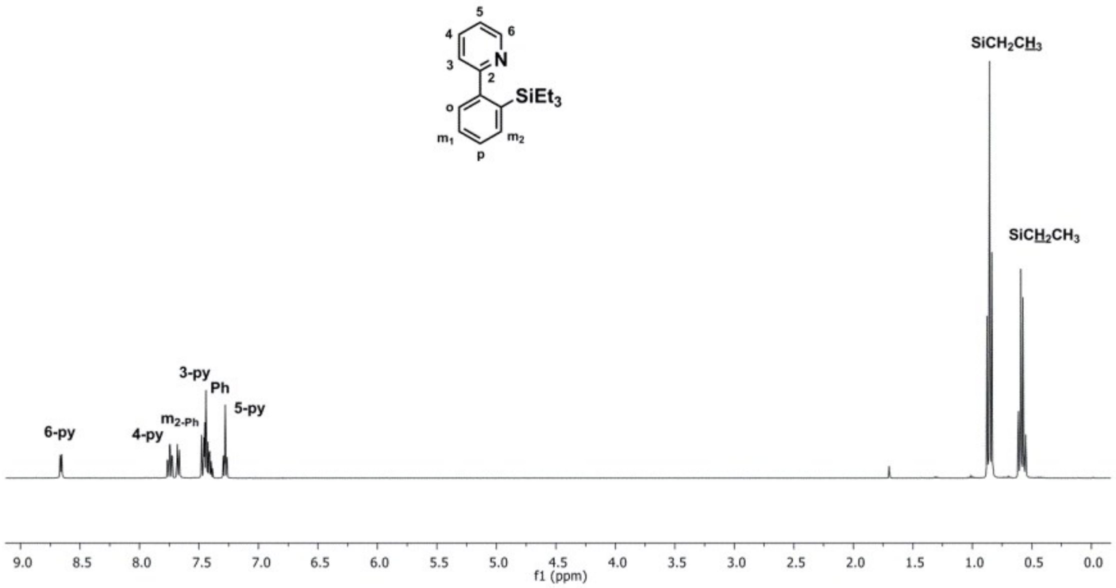

^{13}C NMR

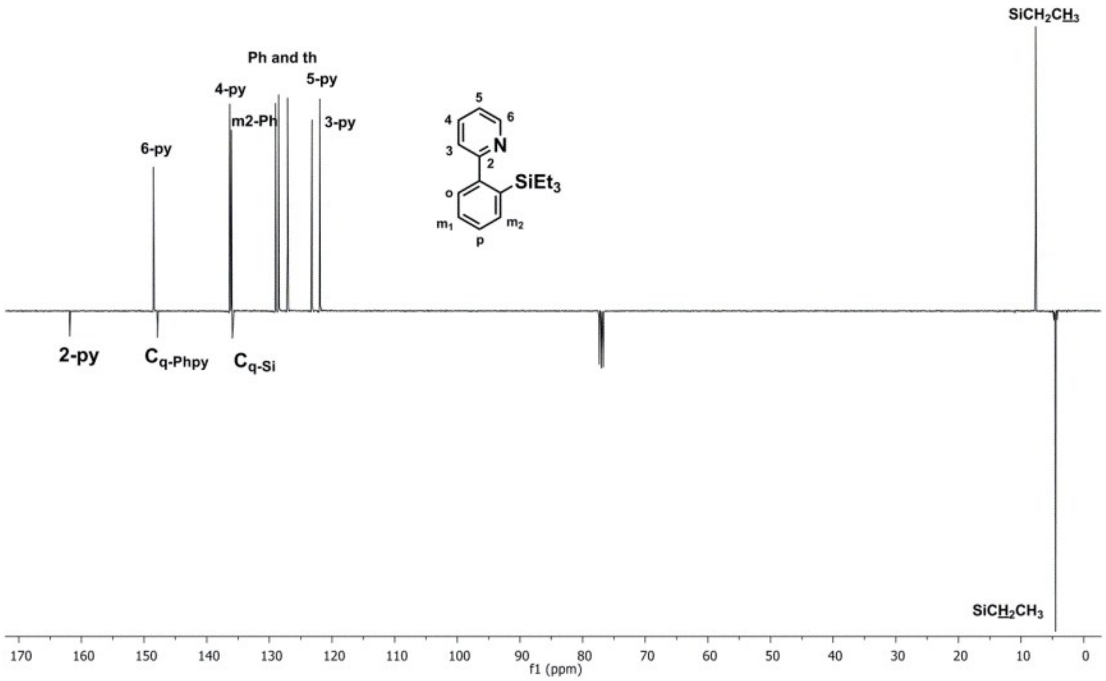

^{1}H-^{1}H COSY

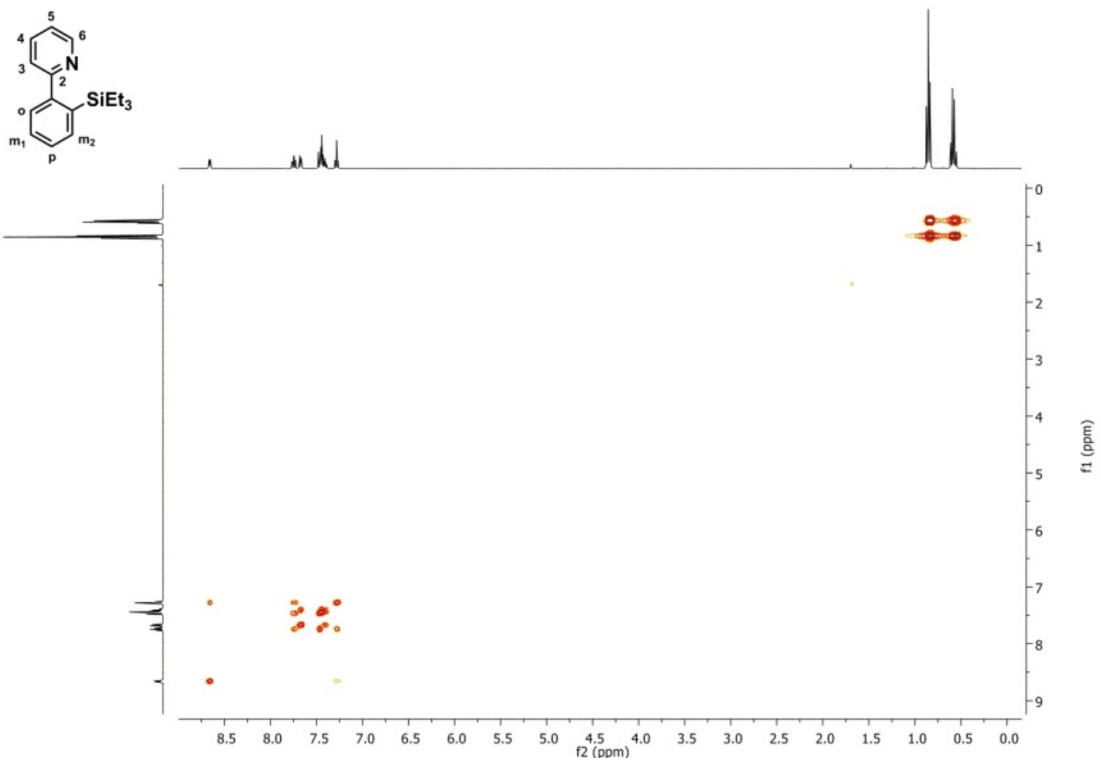

^{1}H-^{13}C HSQC

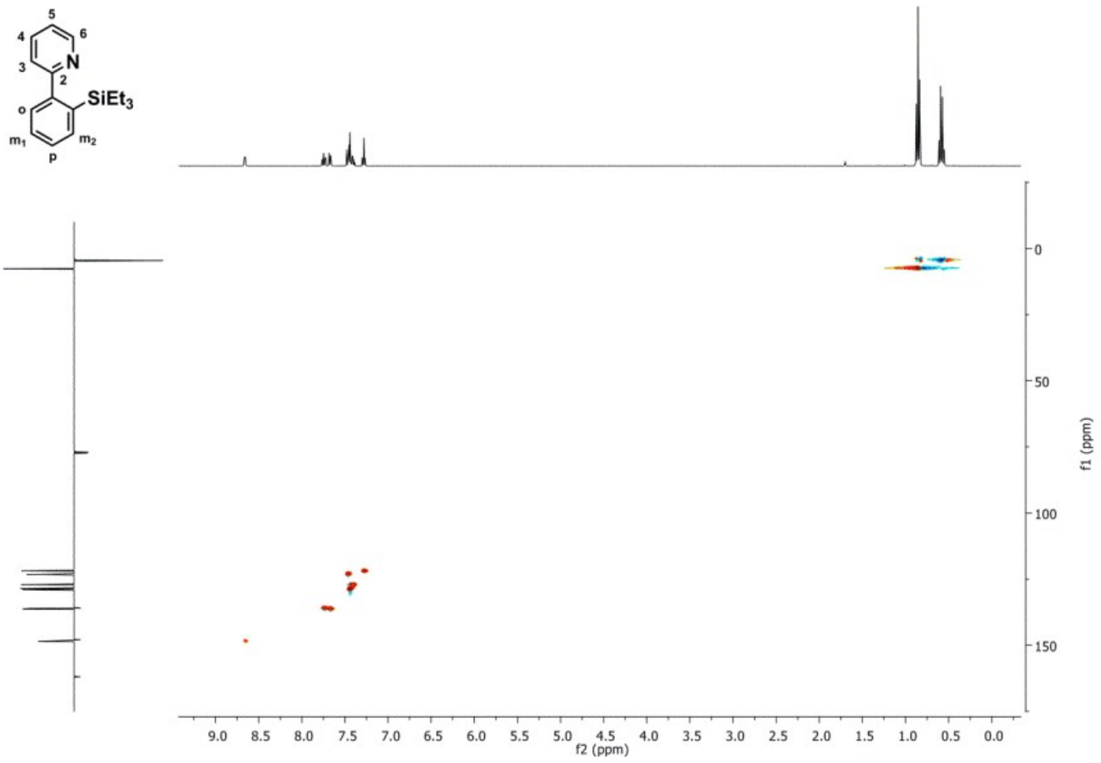

^{1}H-^{13}C HMBC

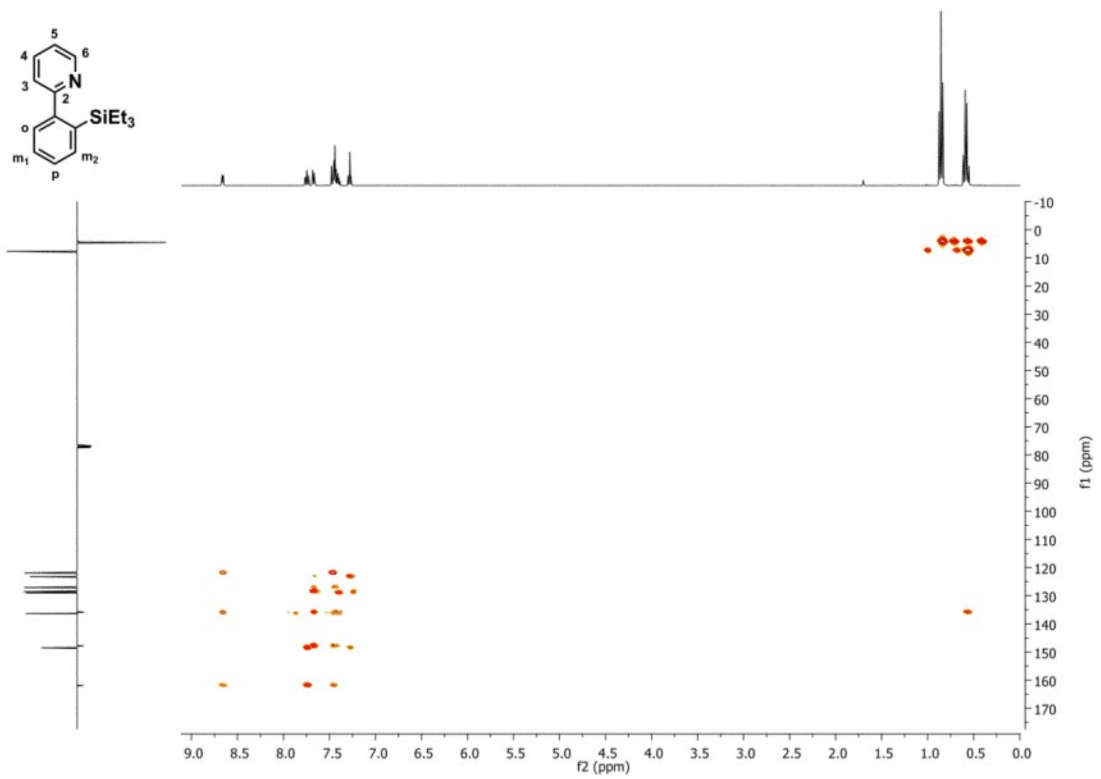

^{1}H-^{29}Si HMBC

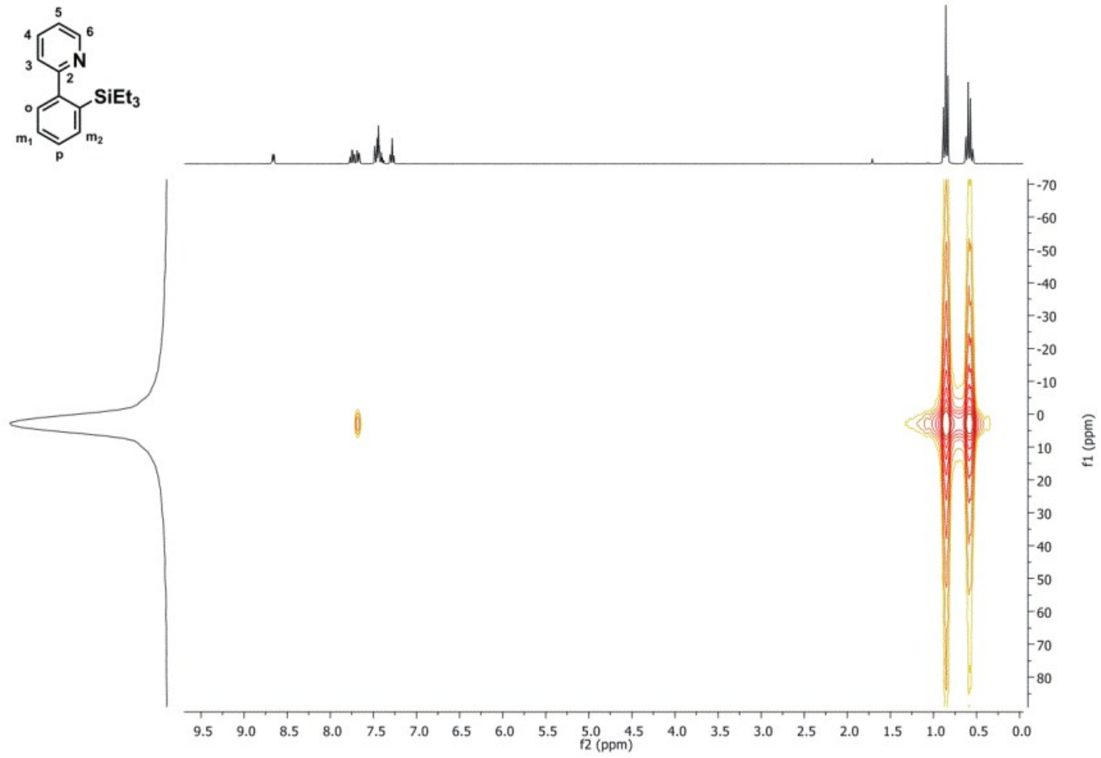

2-(2-(dimethylphenylsilyl)phenyl)pyridine

^{1}H NMR

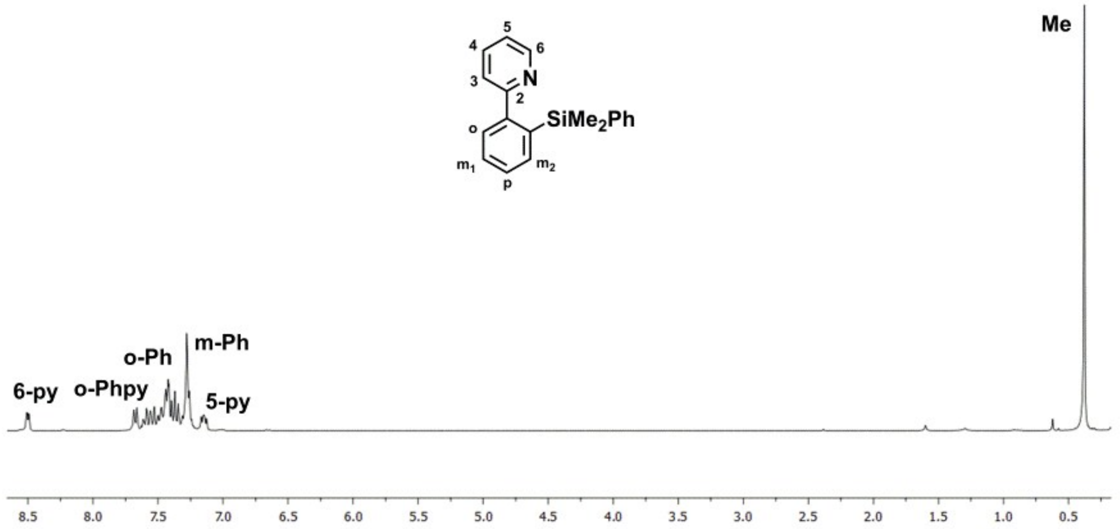

^{13}C NMR

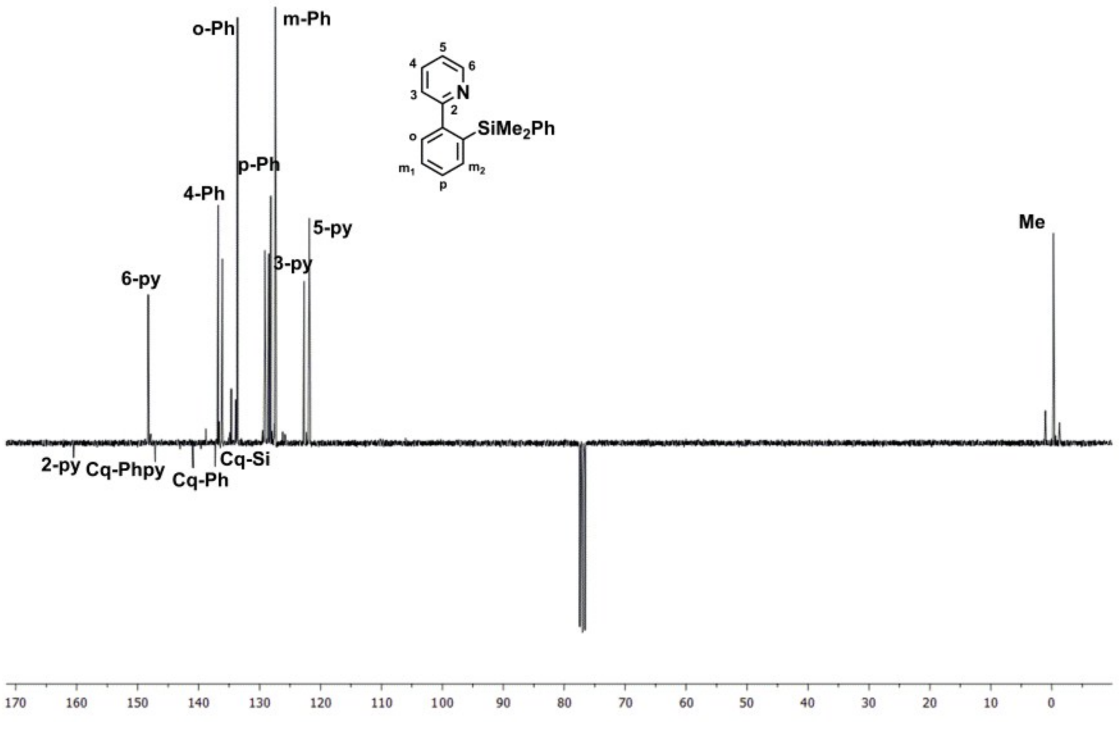

^{1}H-^{13}C HSQC

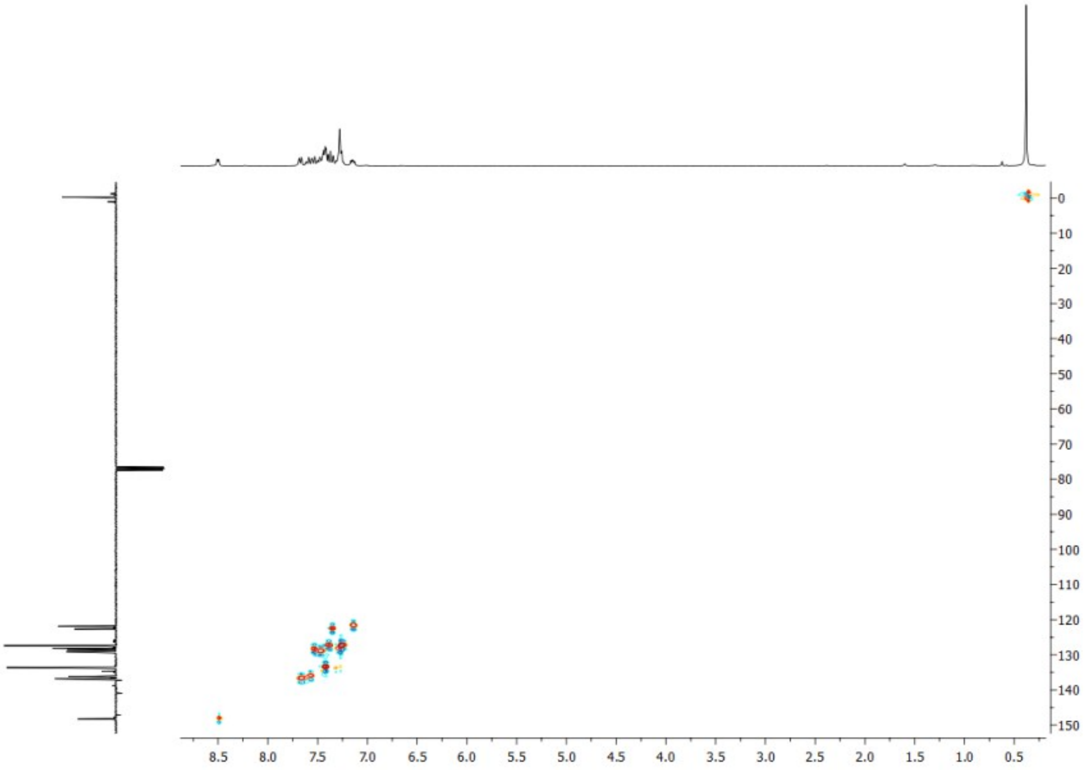

^{1}H-^{13}C HMBC

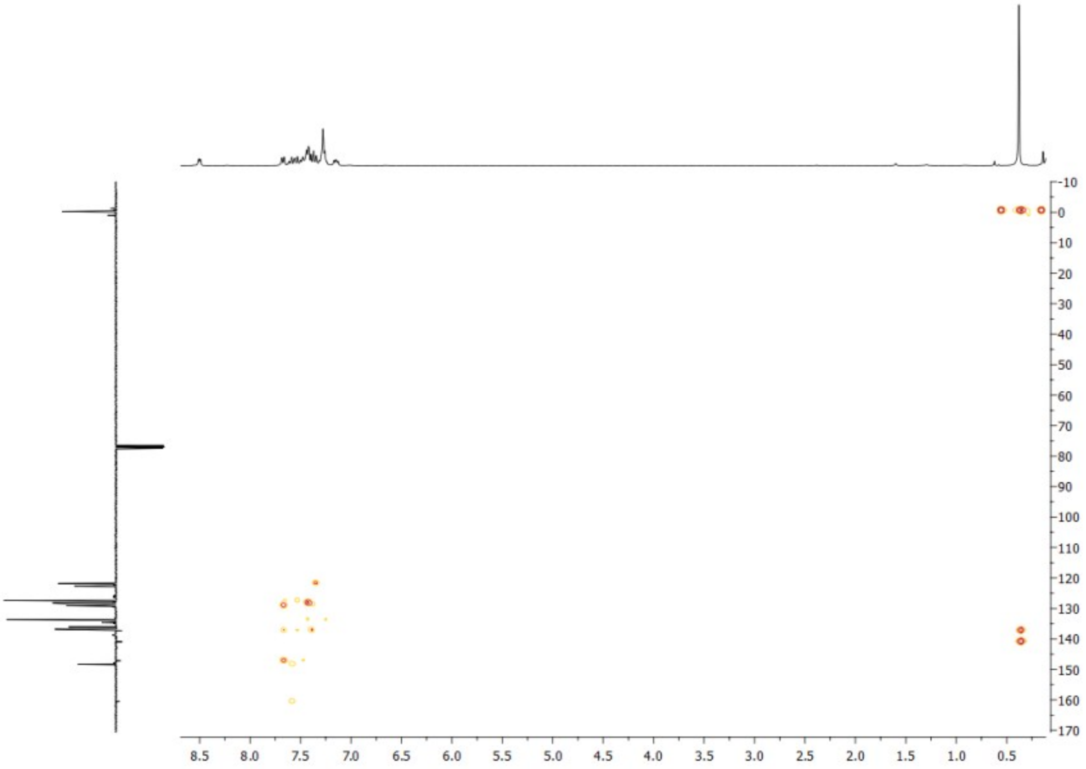

^{1}H-^{29}Si HMBC

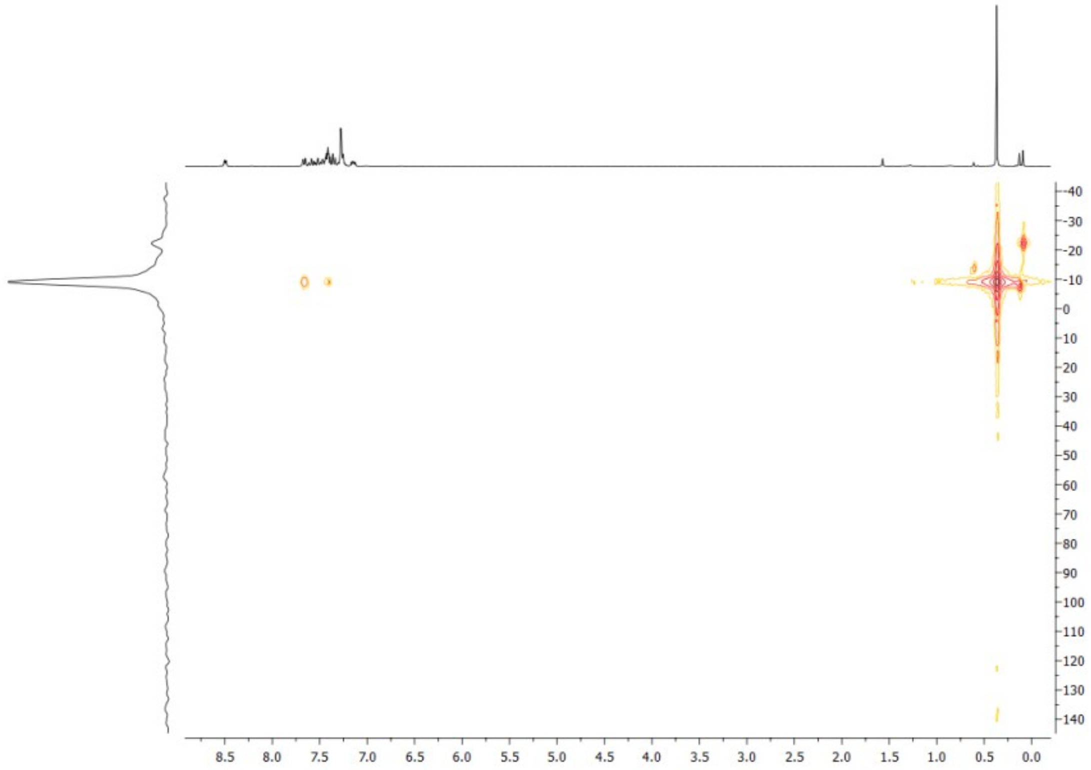

-40
-30
-20
-10
0
10
20
30
40
50
60
70
80
90
100
110
120
130
140
8.5
8.0
7.5
7.0
6.5
6.0
5.5
5.0
4.5
4.0
3.5
3.0
2.5
2.0
1.5
1.0
0.5
0.0

2-(2-(methyldiphenylsilyl)phenyl)pyridine

^{1}H NMR

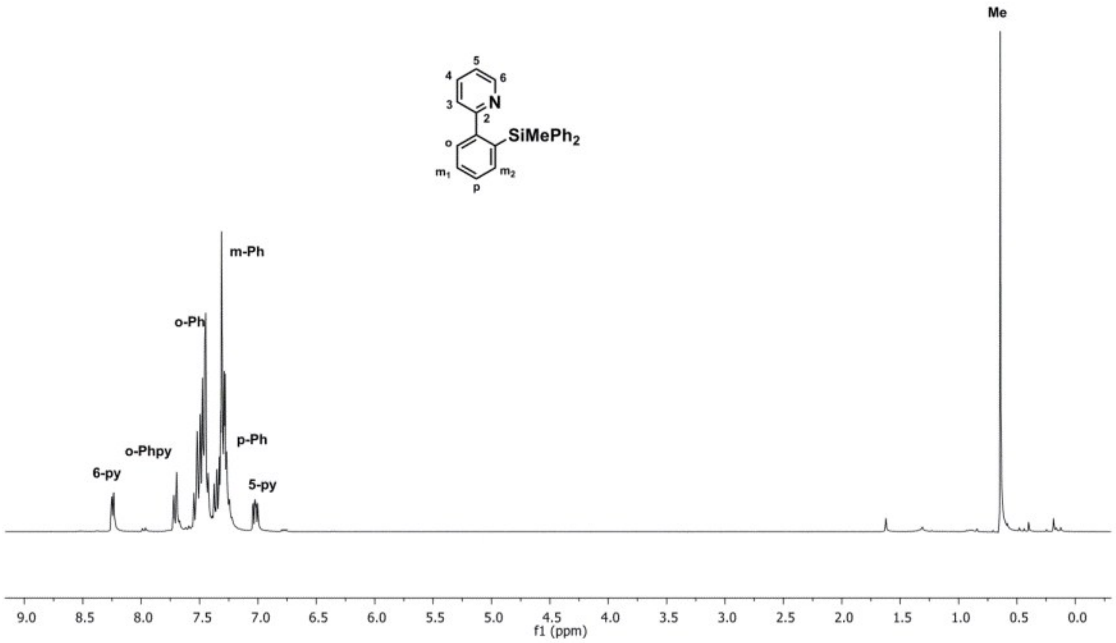

^{13}C NMR

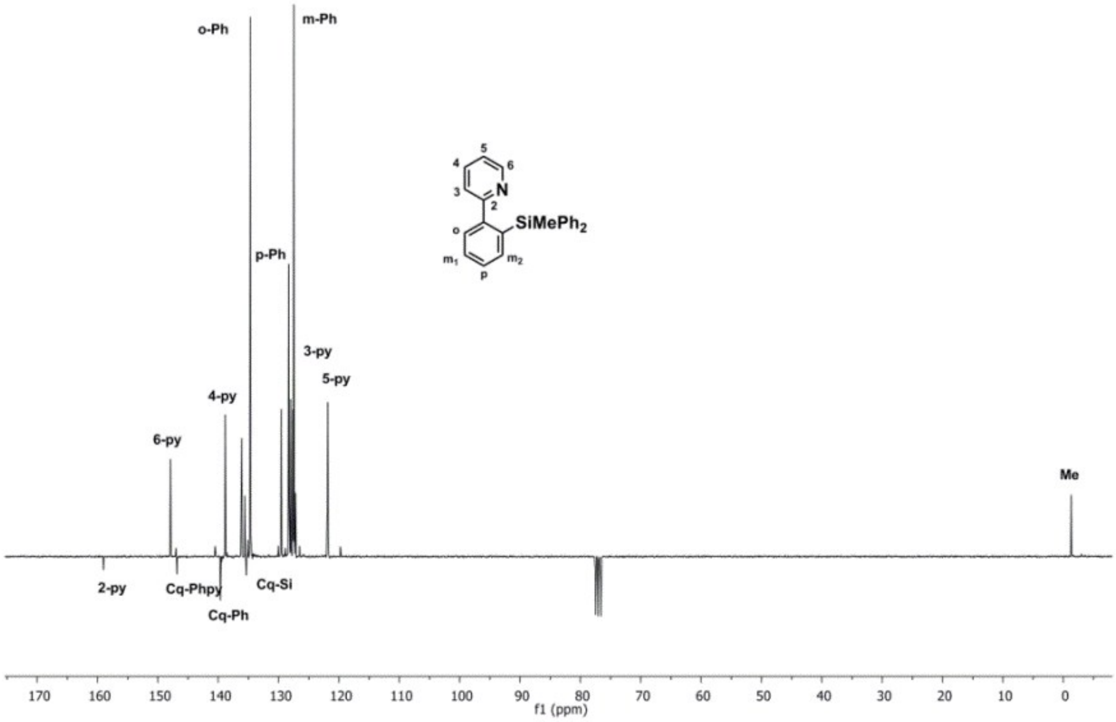

^{1}H-^{29}Si HMBC

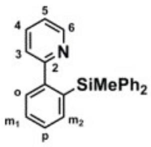

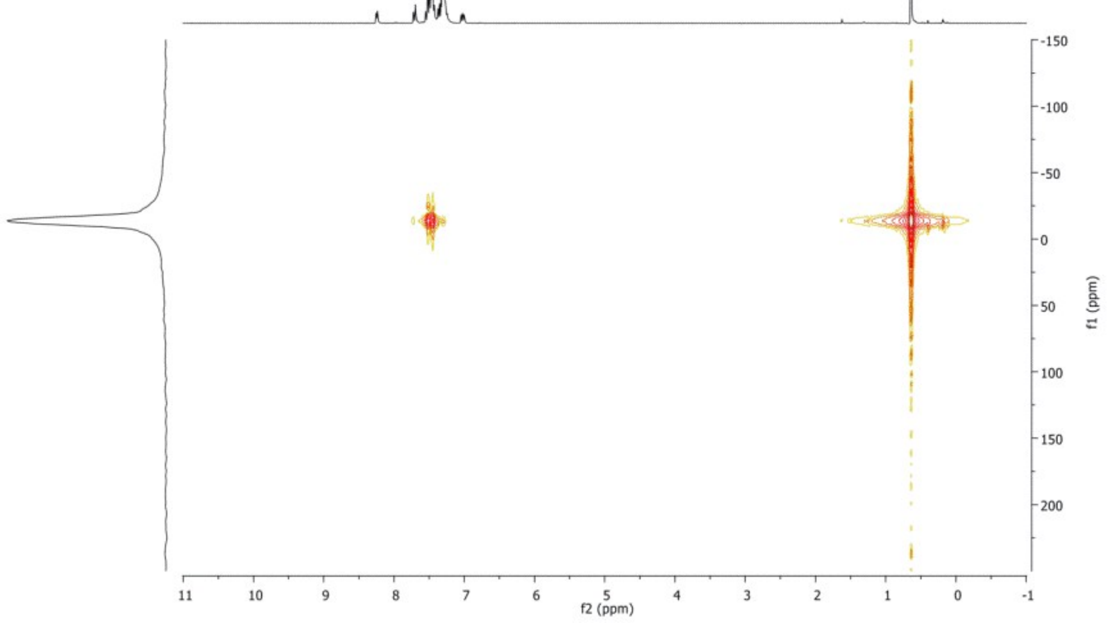

2-(2-(triphenylsilyl)phenyl)pyridine

^{1}H NMR

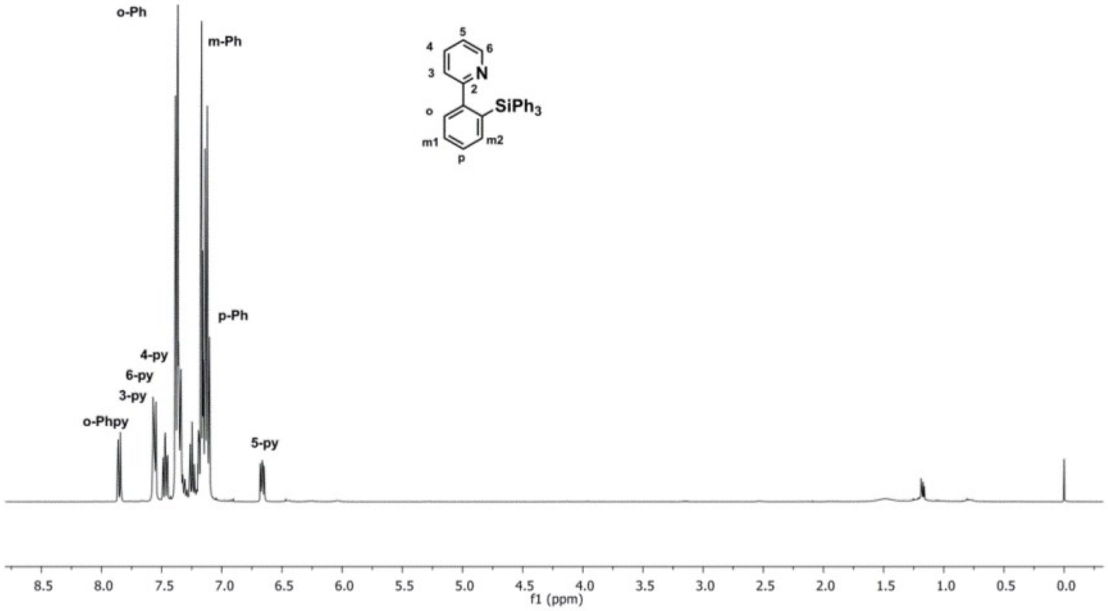

1

^{3}C NMR

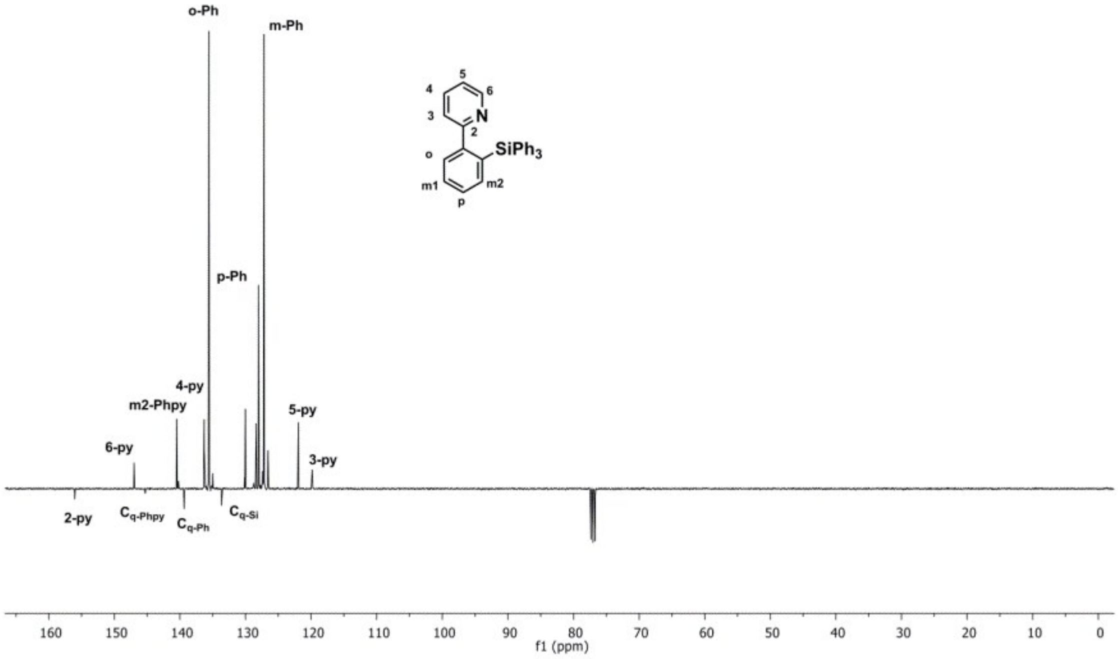

^{1}H-^{29}Si HMBC

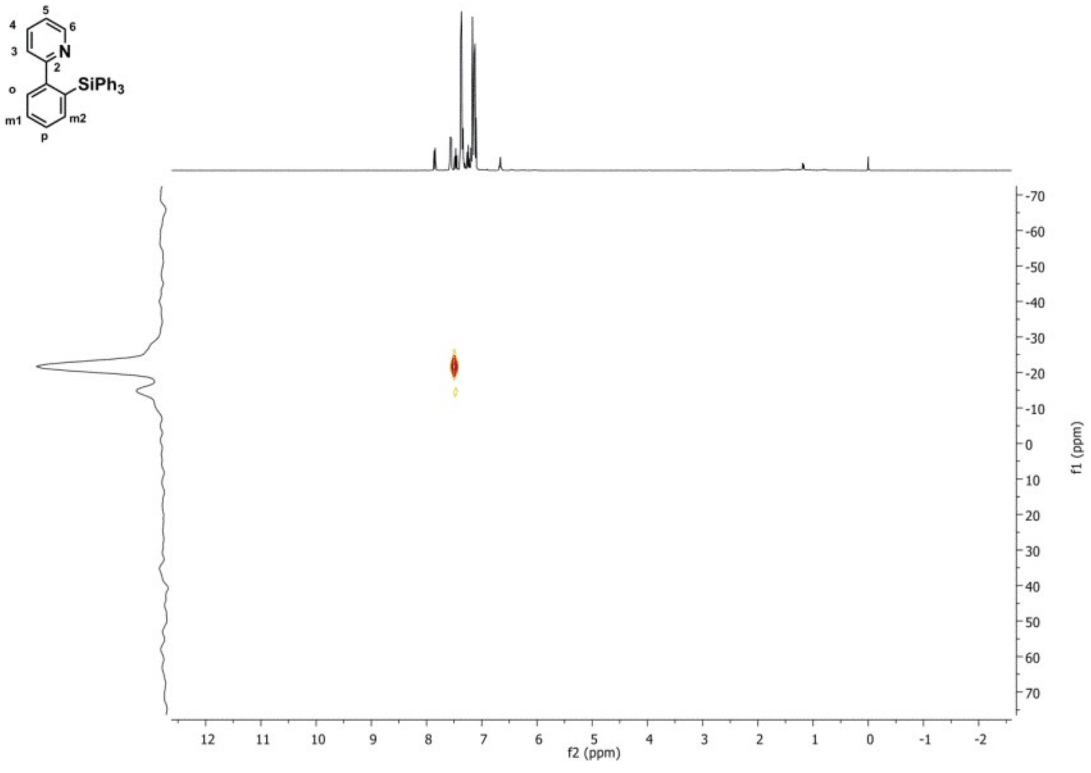

1-(2-(triethylsilyl)phenyl)-1H-pyrazole

^{1}H NMR

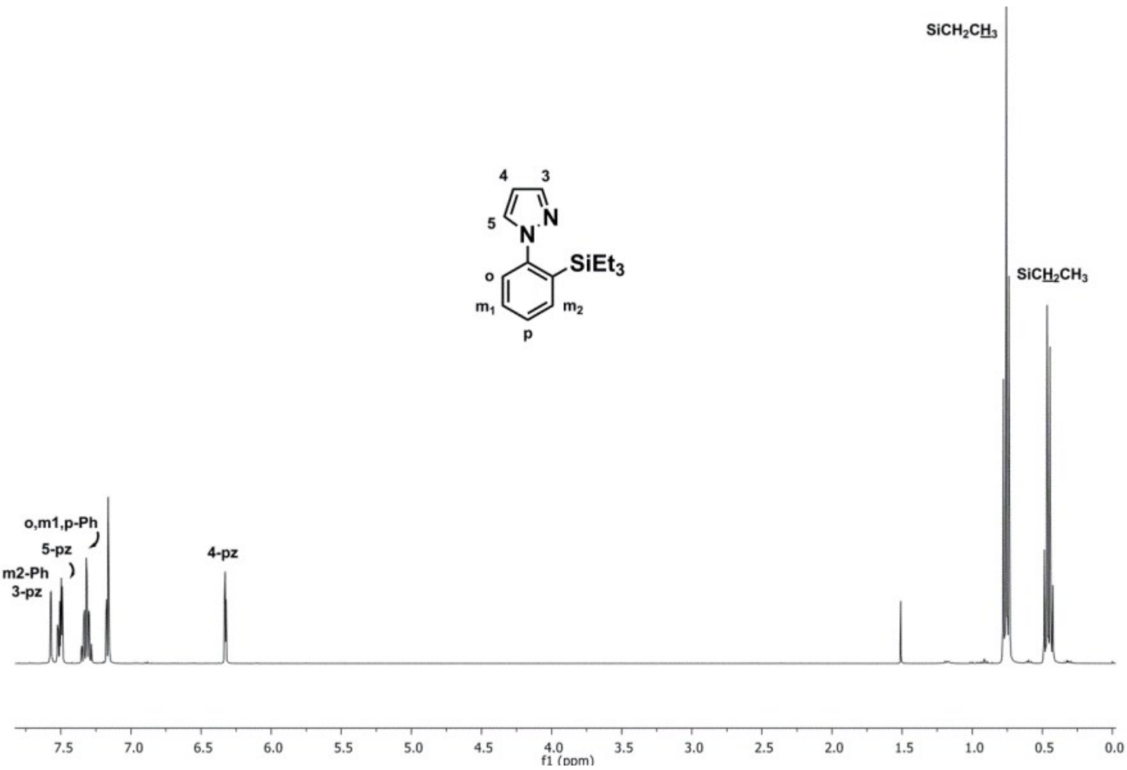

^{13}C NMR

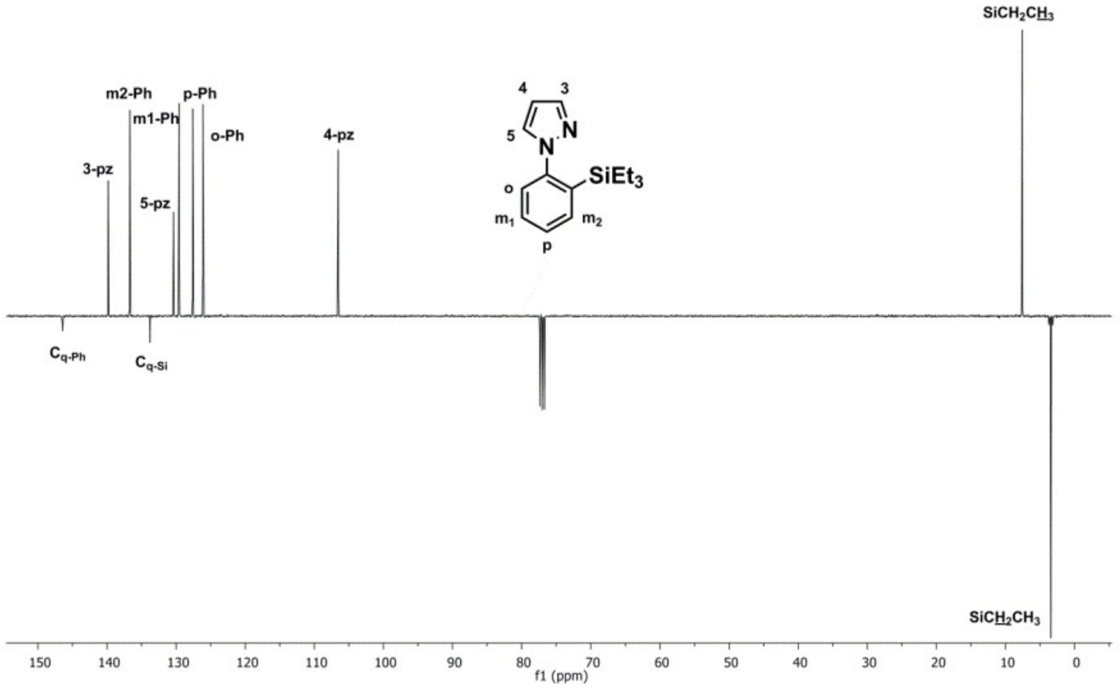

^{1}H-^{1}H COSY

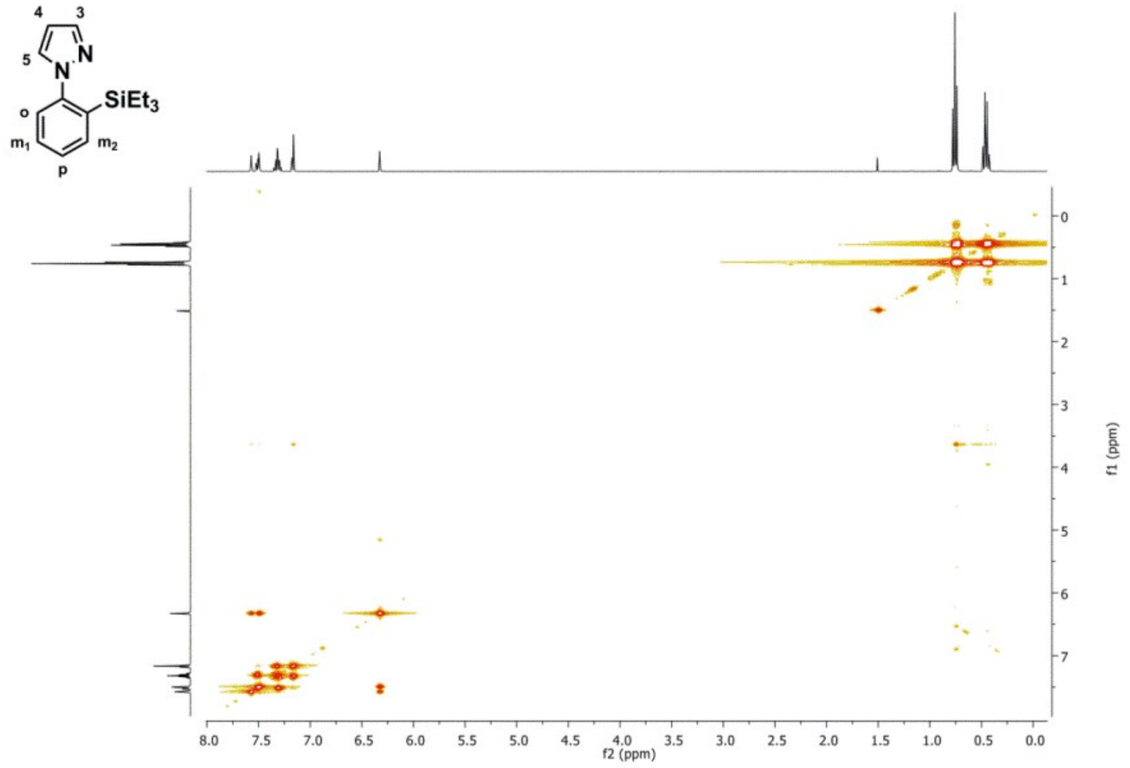

^{1}H-^{13}C HSQC

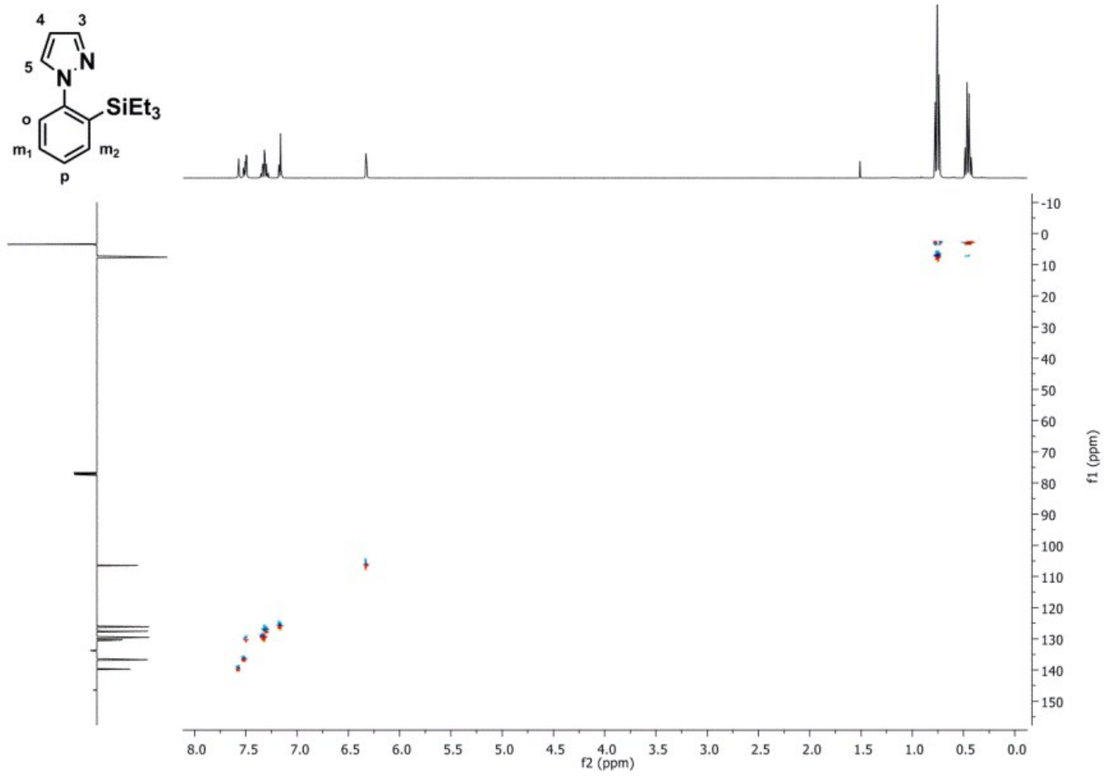

^1H-^{13}C HMBC

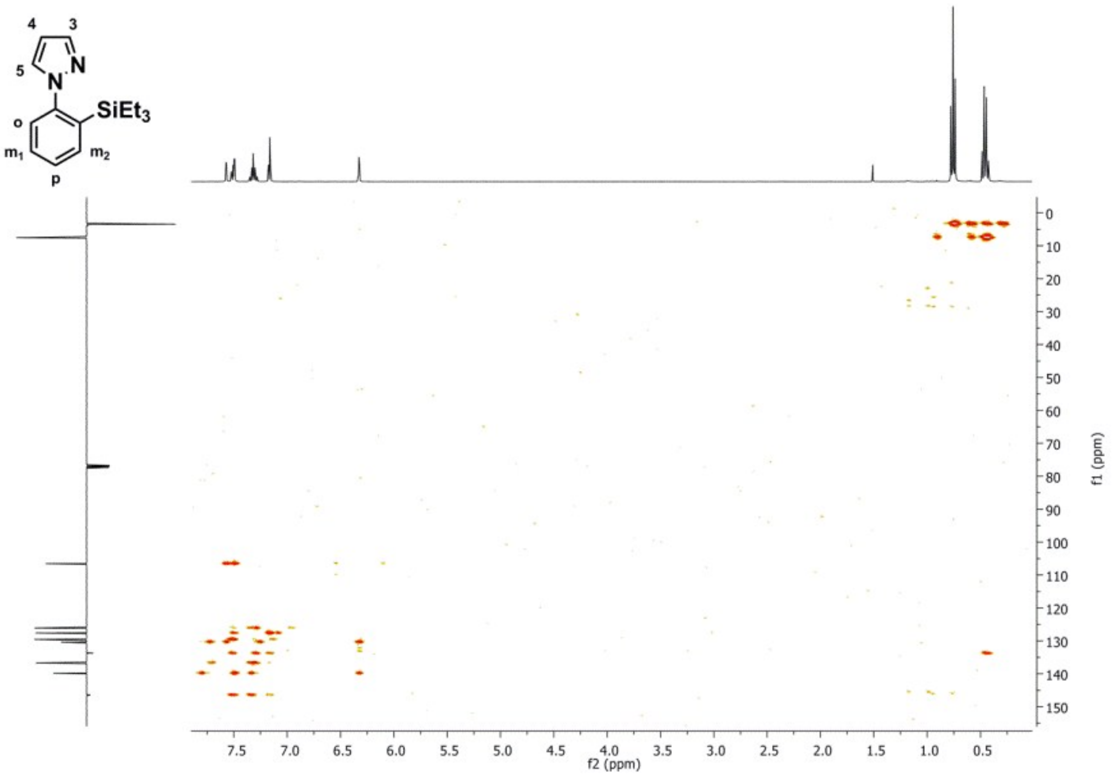

^1H-^{29}Si HMBC

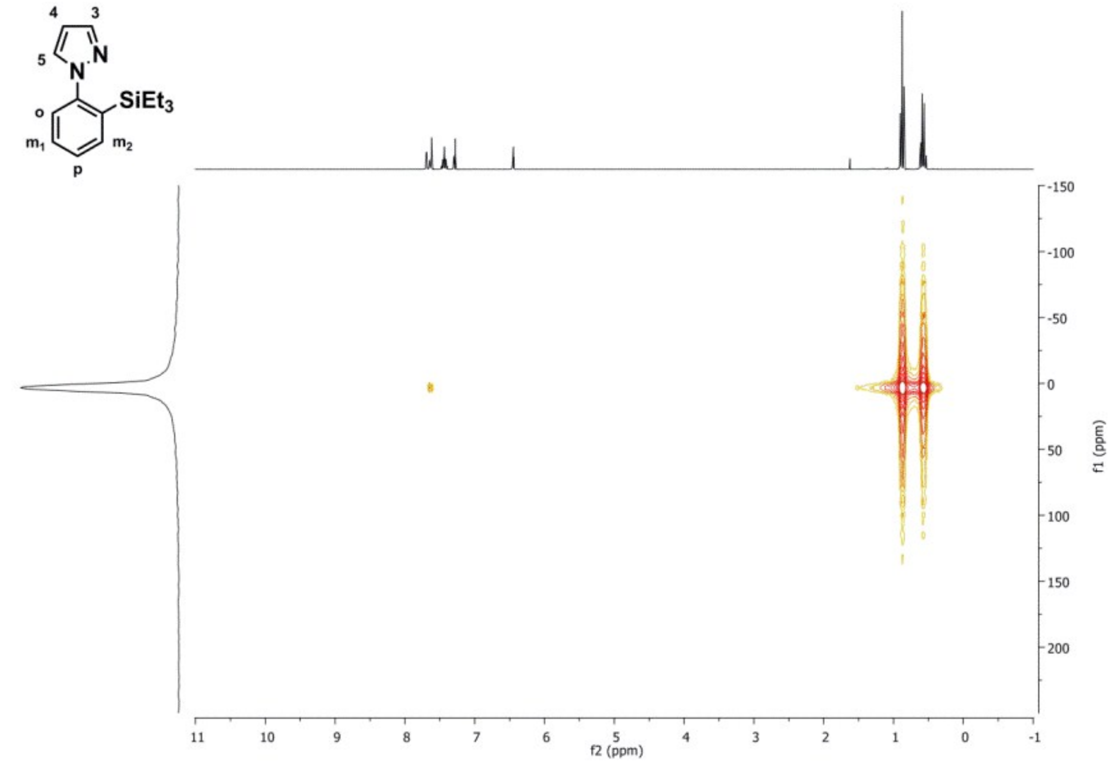

1-(2-(dimethylphenylsilyl)phenyl)-1H-pyrazole

^{1}H NMR

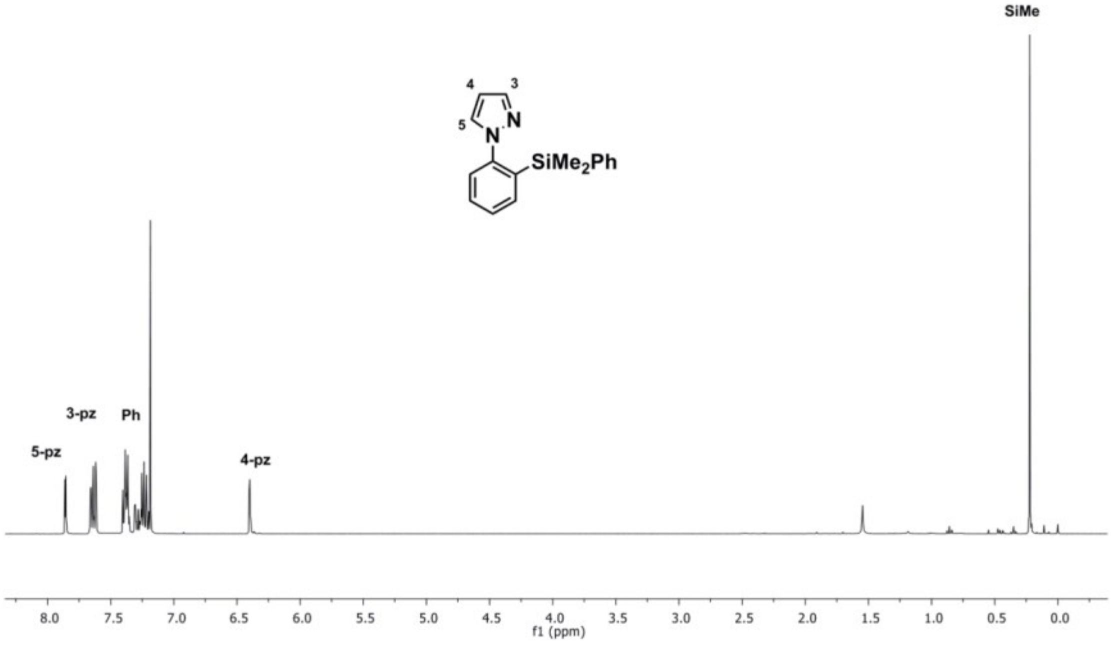

^{13}C NMR

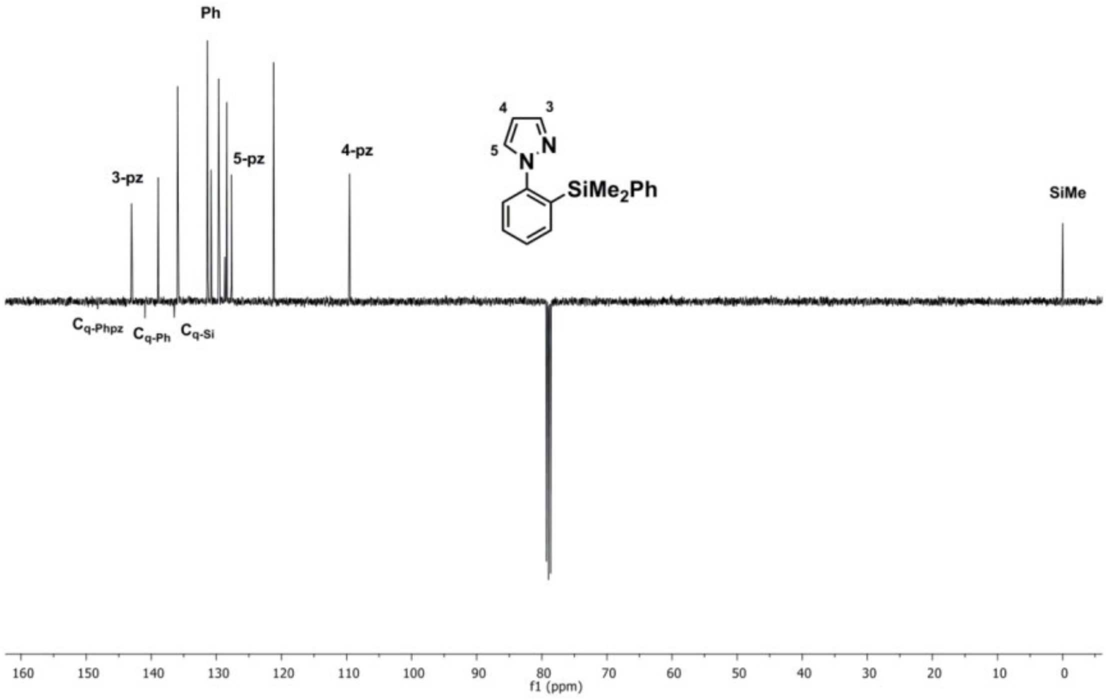

^{1}H-^{29}Si HMBC

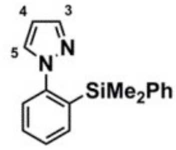

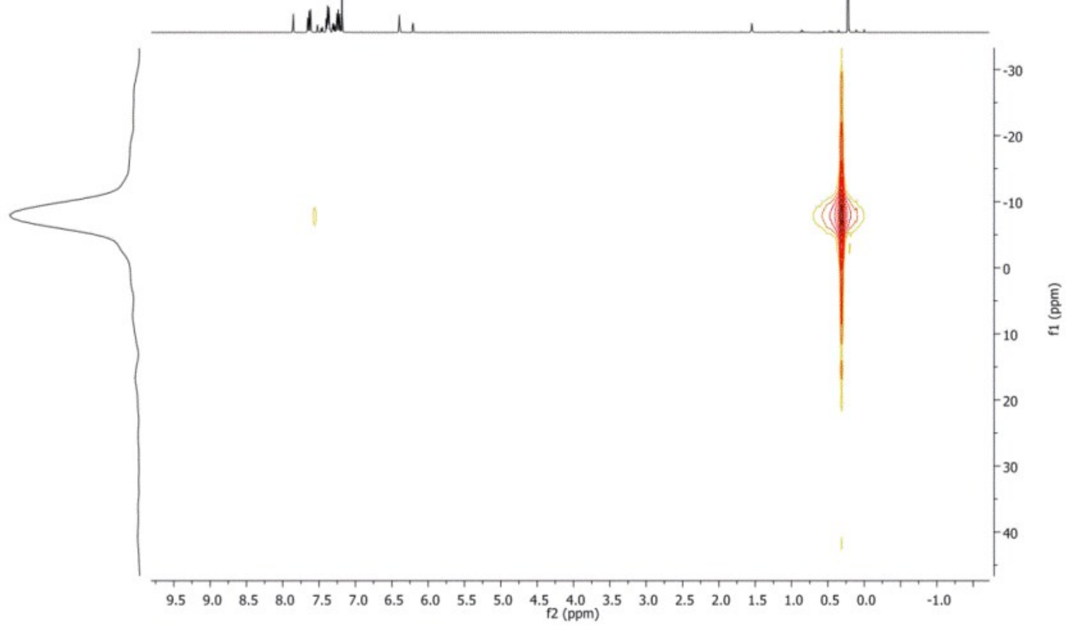

1-(2-(methyldiphenylsilyl)phenyl)-1H-pyrazole

^{1}H NMR

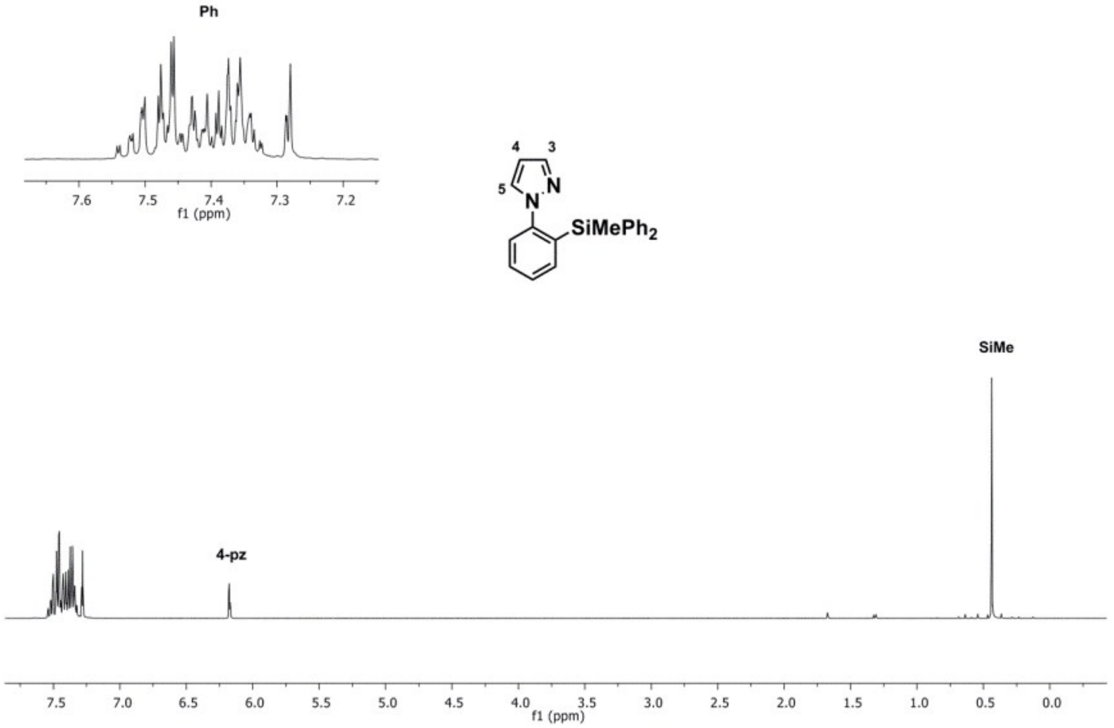

^{13}C NMR

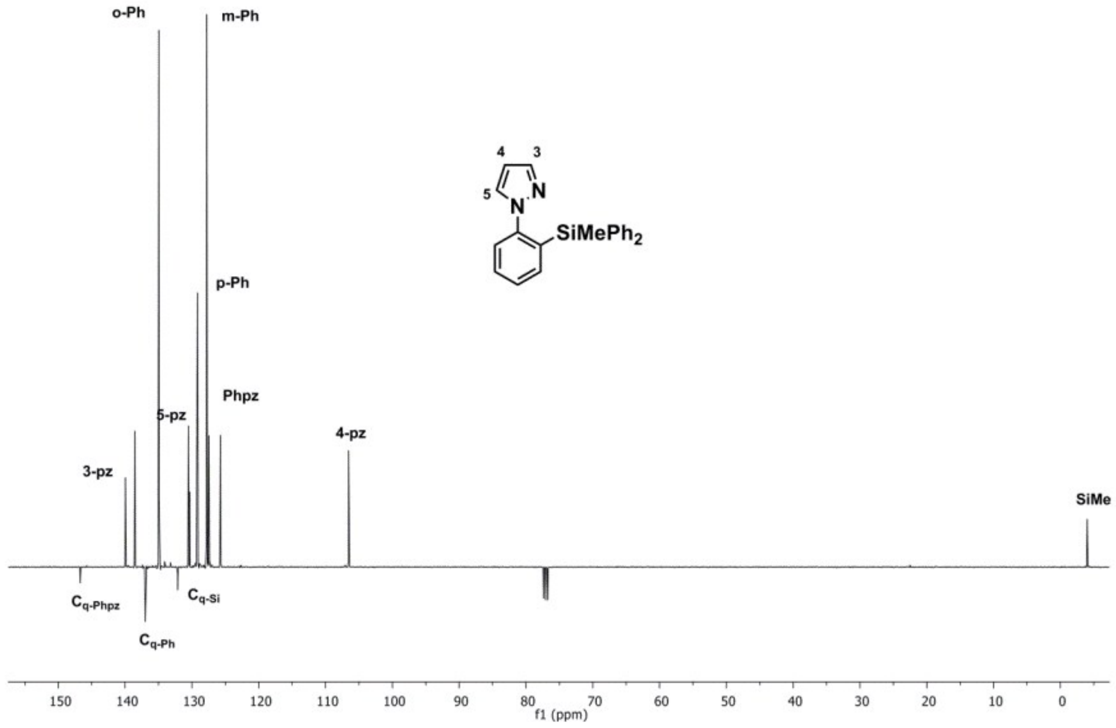

^{1}H-^{29}Si HMBC

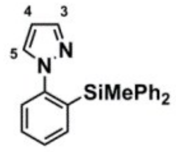
4
3
5
N
N
SiMePh2

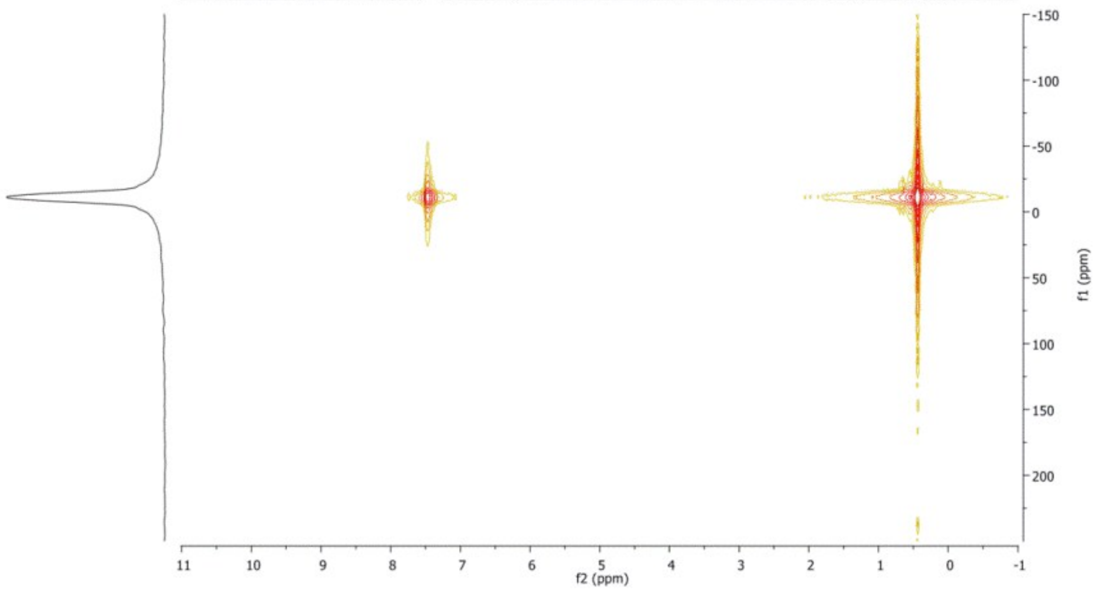
-150
-100
-50
0
50
100
150
200
f1 (ppm)
11
10
9
8
7
6
5
4
3
2
1
0
-1
f2 (ppm)

1-(2-(triphenylsilyl)phenyl)-1H-pyrazole

^{1}H NMR

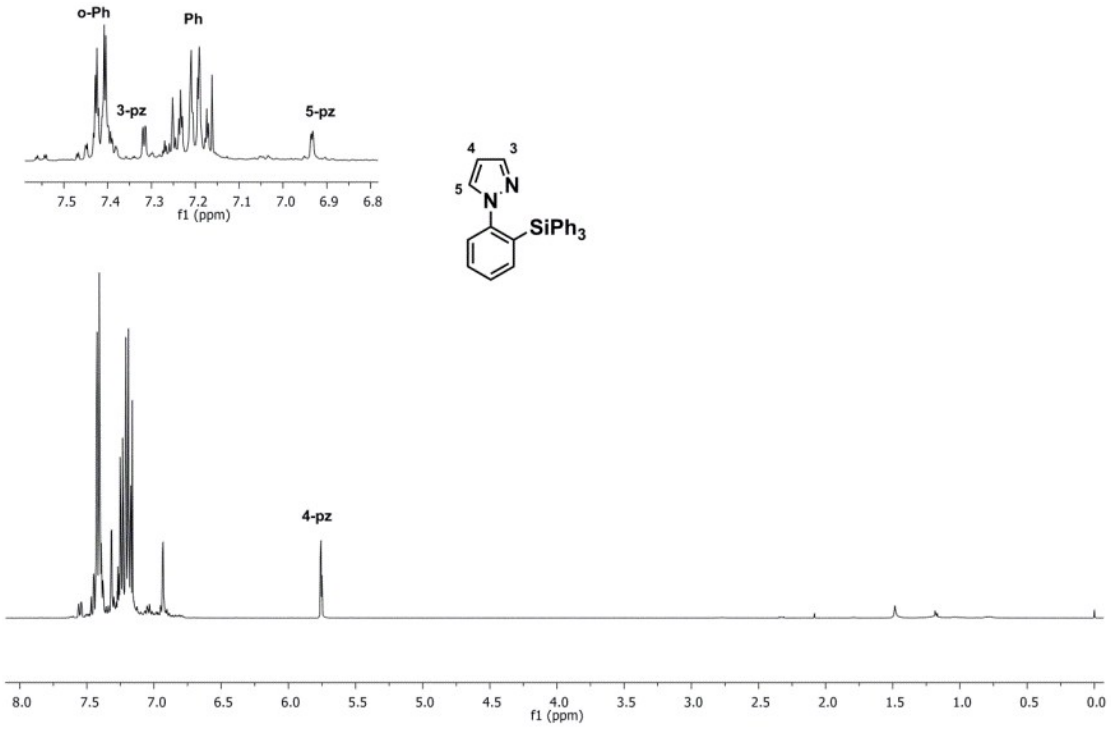

^{13}C NMR

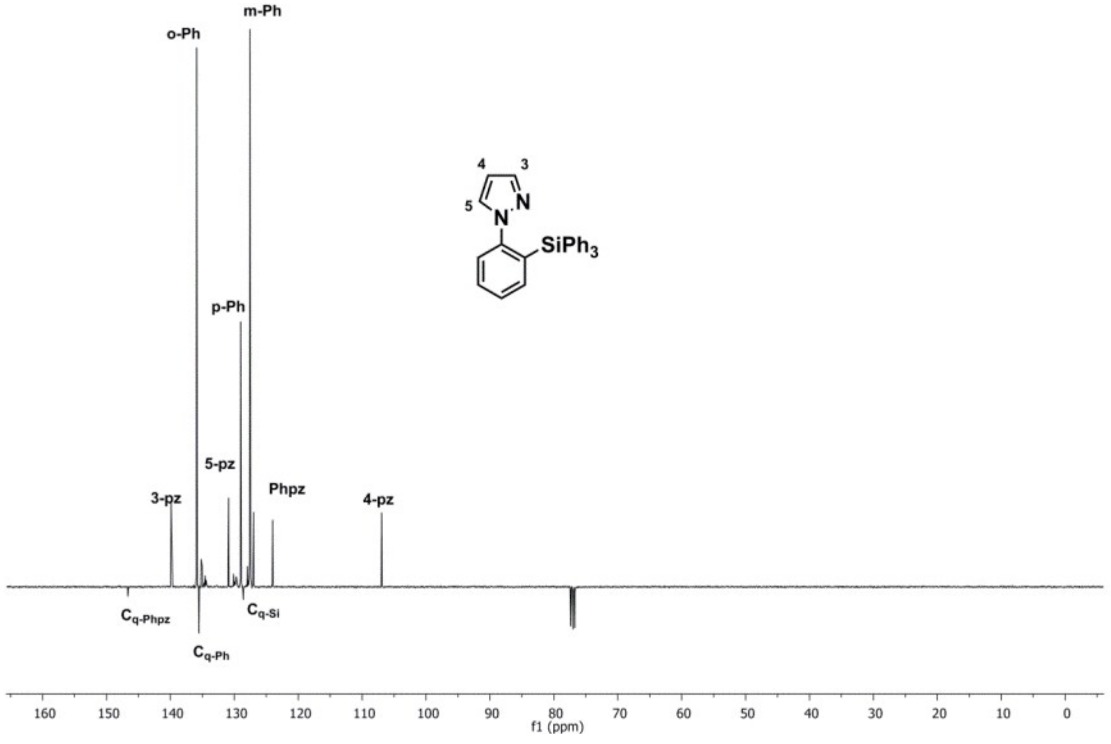

^{1}H-^{29}Si HMBC

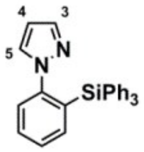

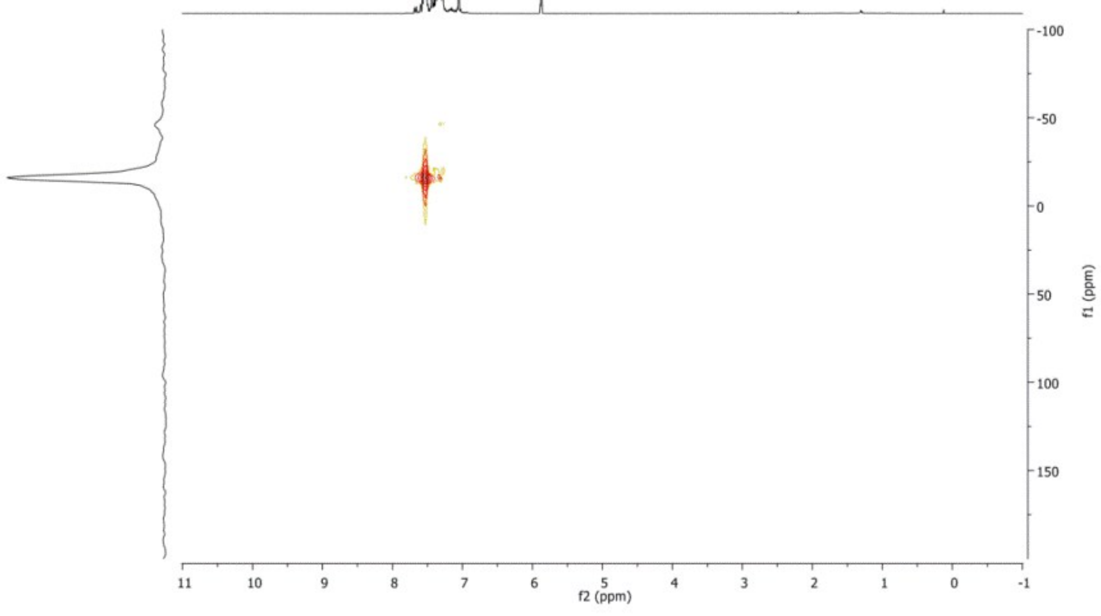

2-(4-methyl-2-(triethylsilyl)phenyl)pyridine

^{1}H NMR

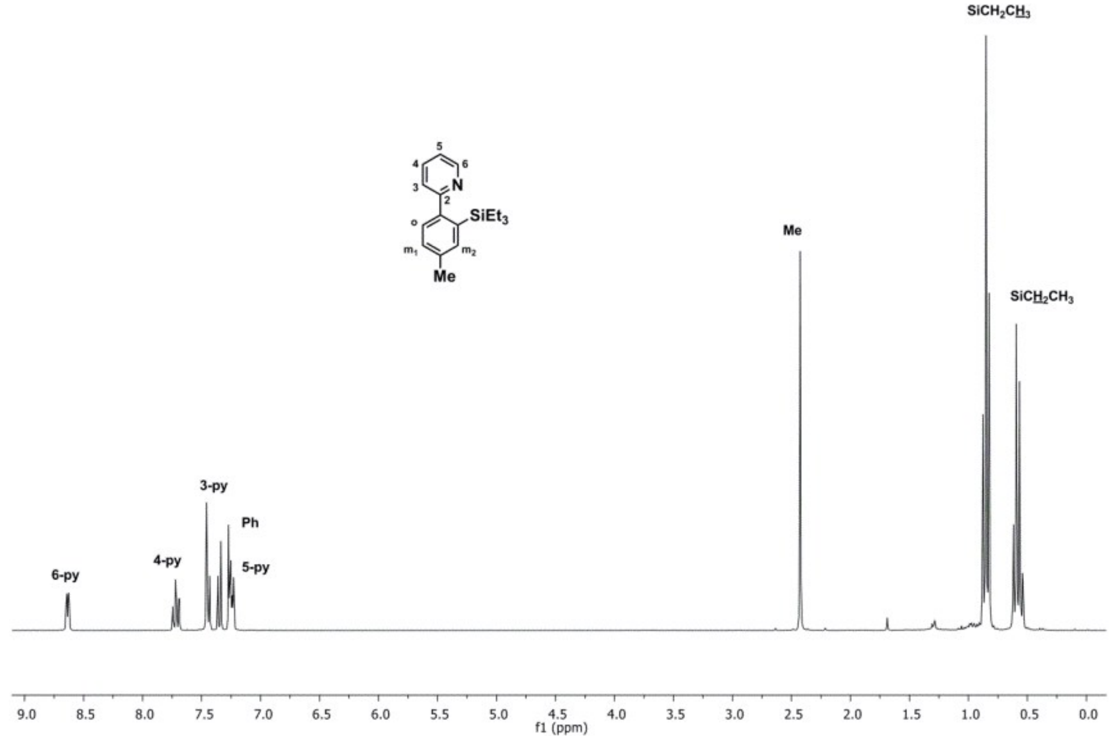

^{13}C NMR

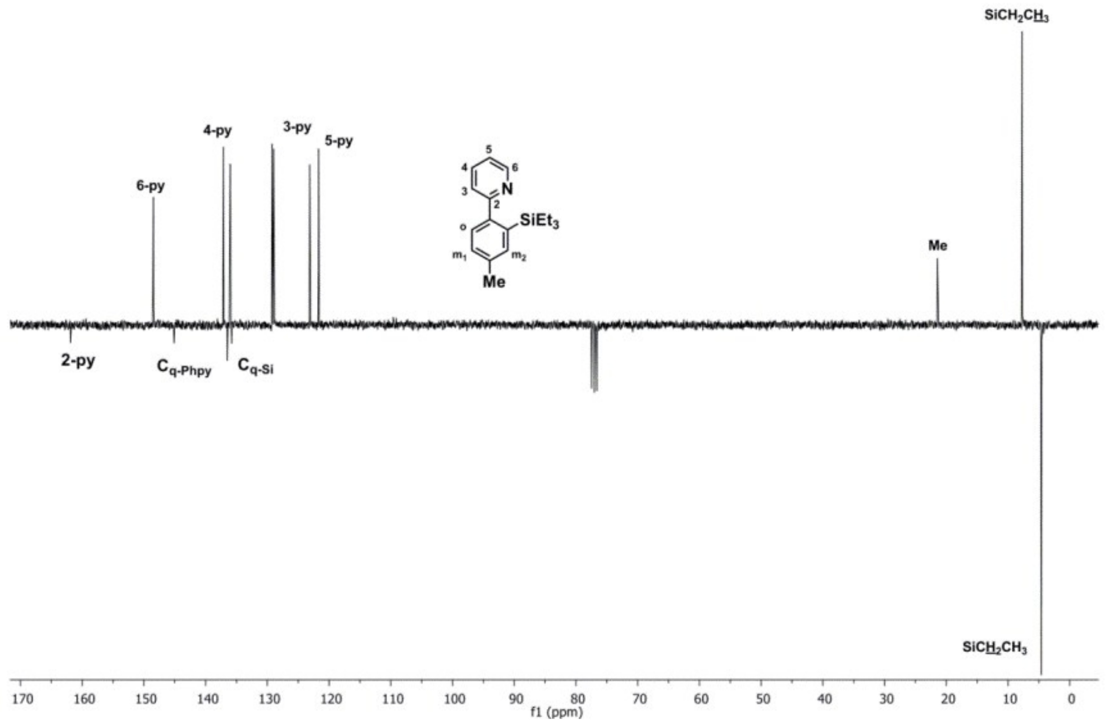

^{1}H-^{1}H COSY

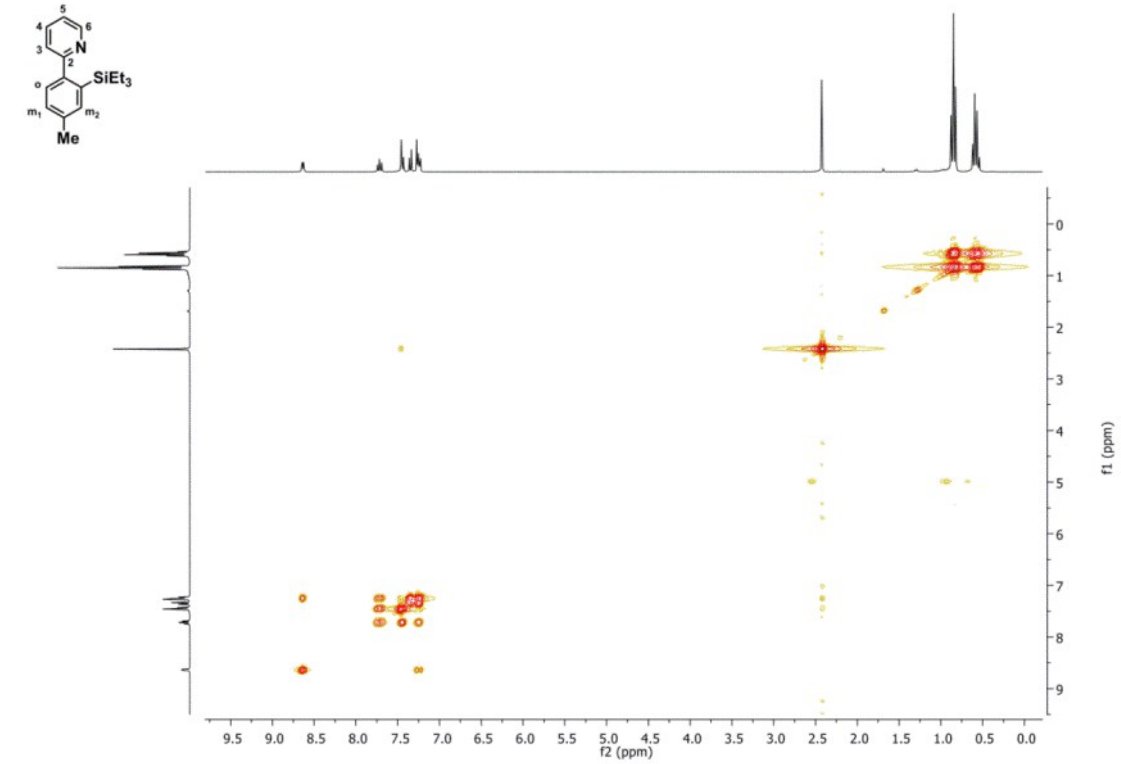

^{1}H-^{13}C HSQC

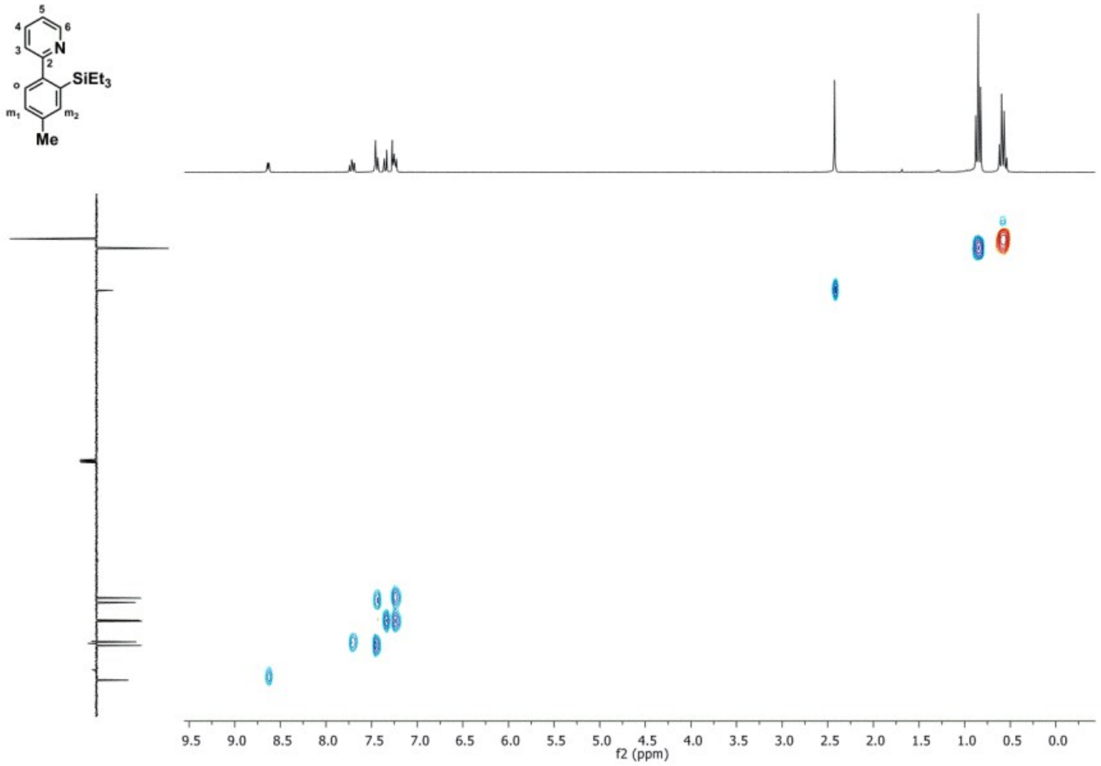

^{1}H-^{13}C HMBC

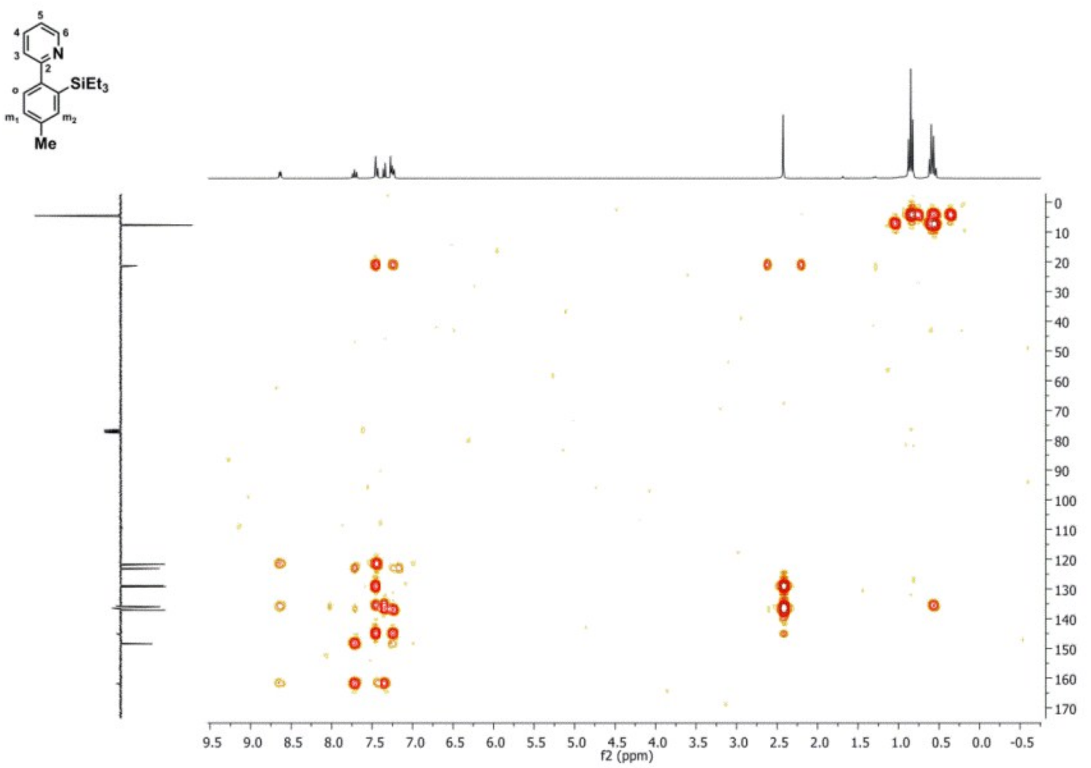

^{1}H-^{29}Si HMBC

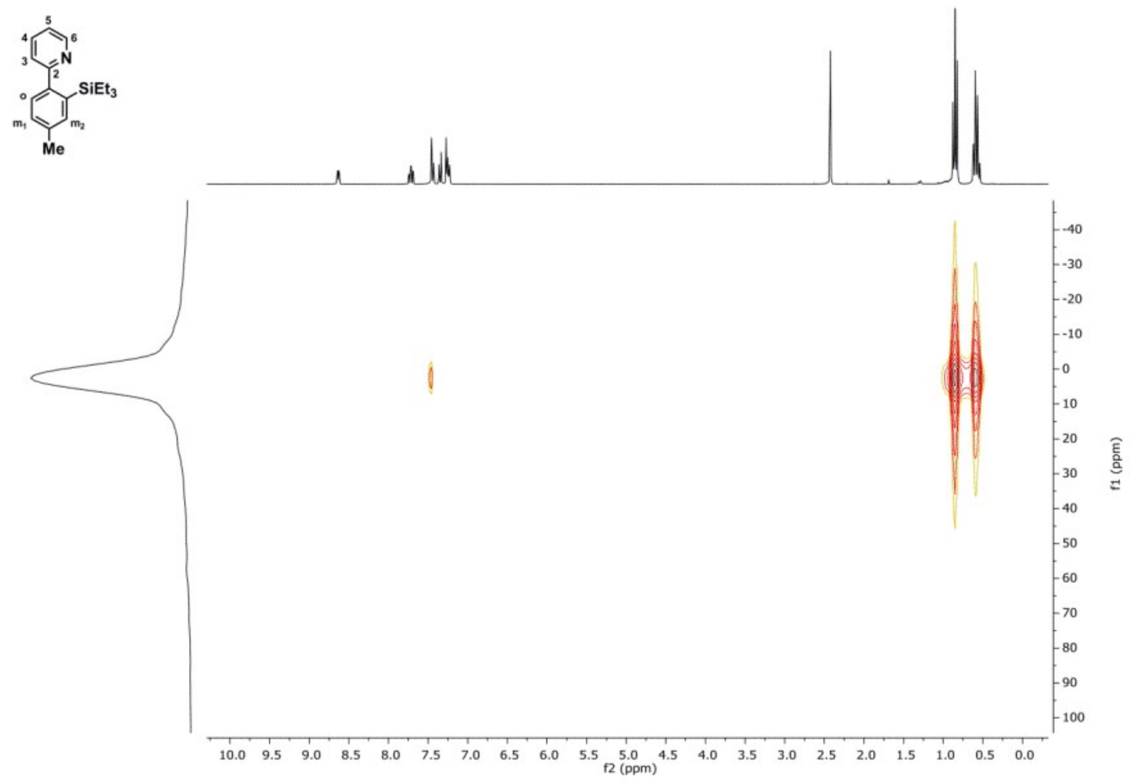

5-(dimethyl(phenyl)silyl)-2-(p-tolyl)pyridine

^{1}H NMR

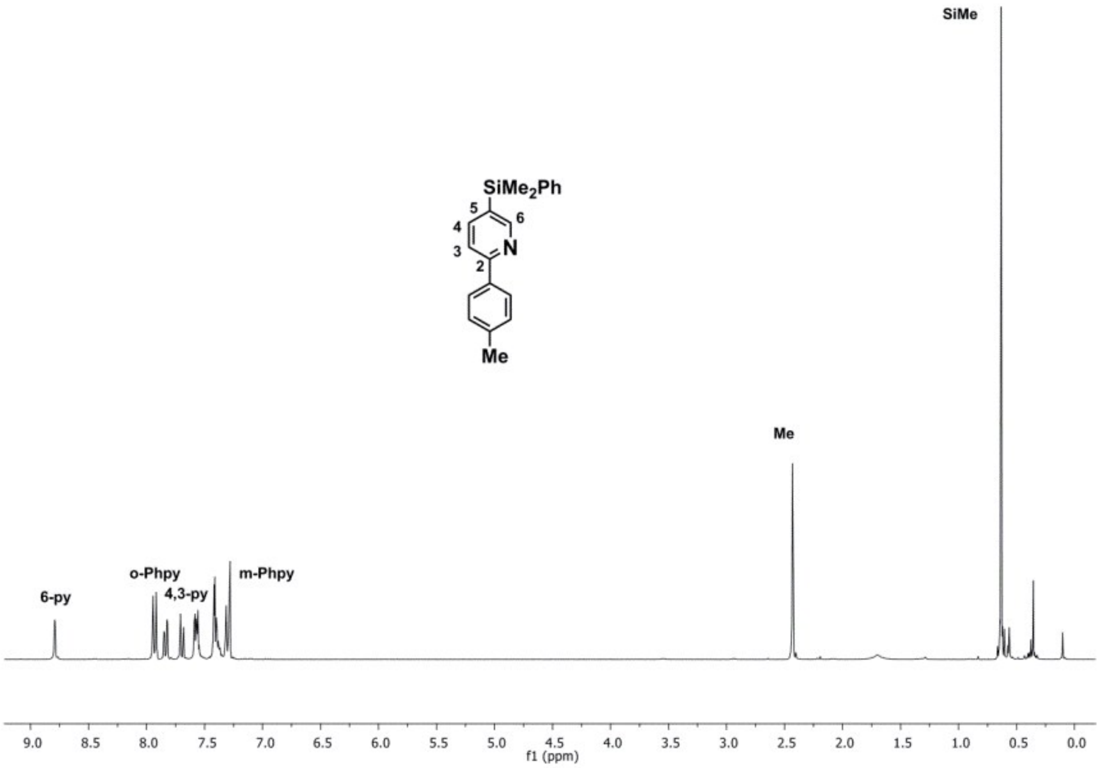

^{13}C NMR

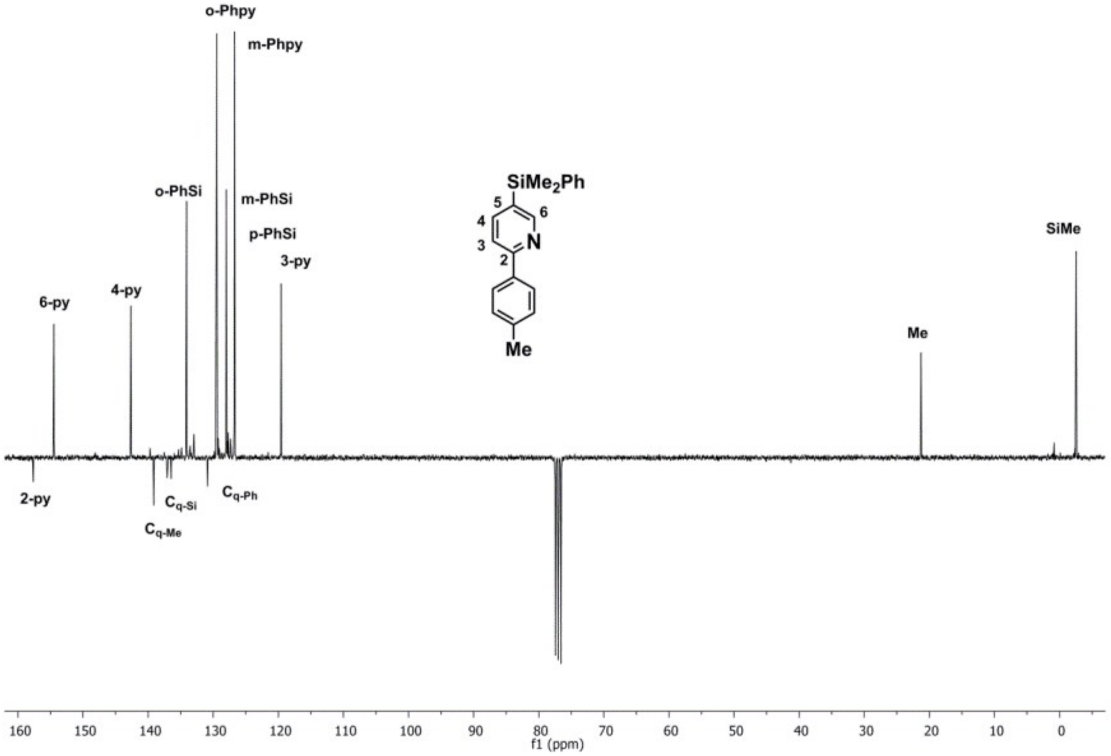

^{1}H-^{1}H COSY

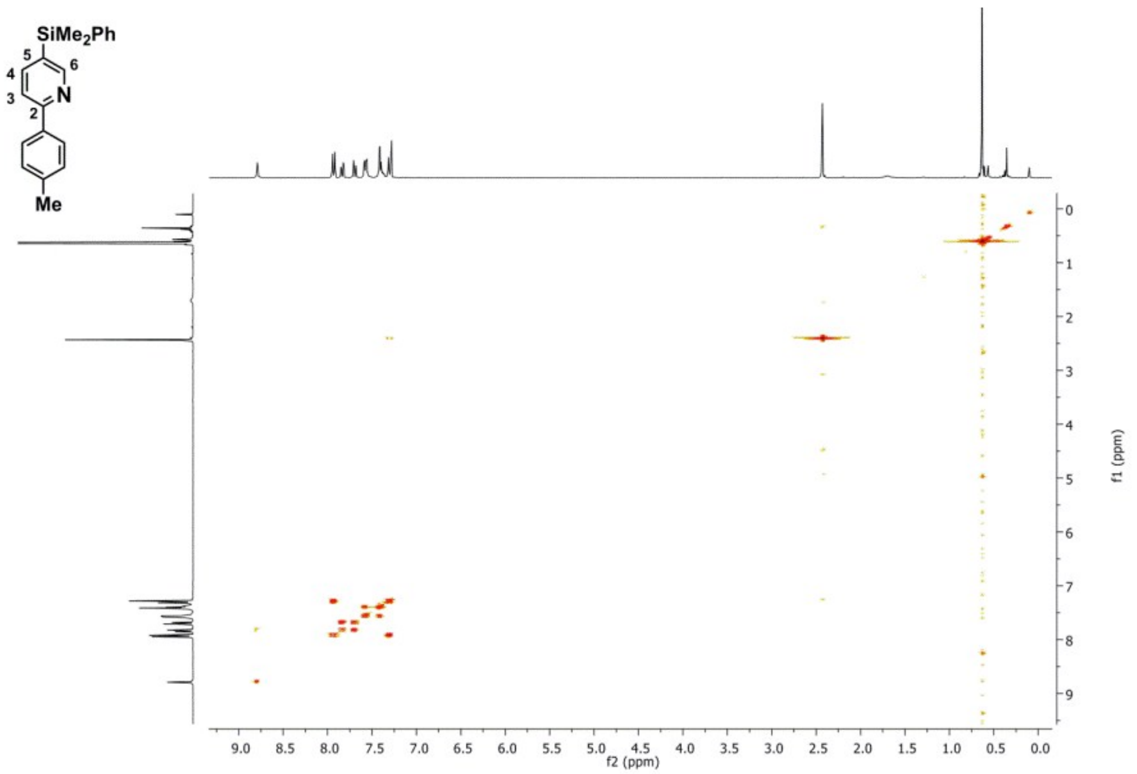

^{1}H-^{13}C HSQC

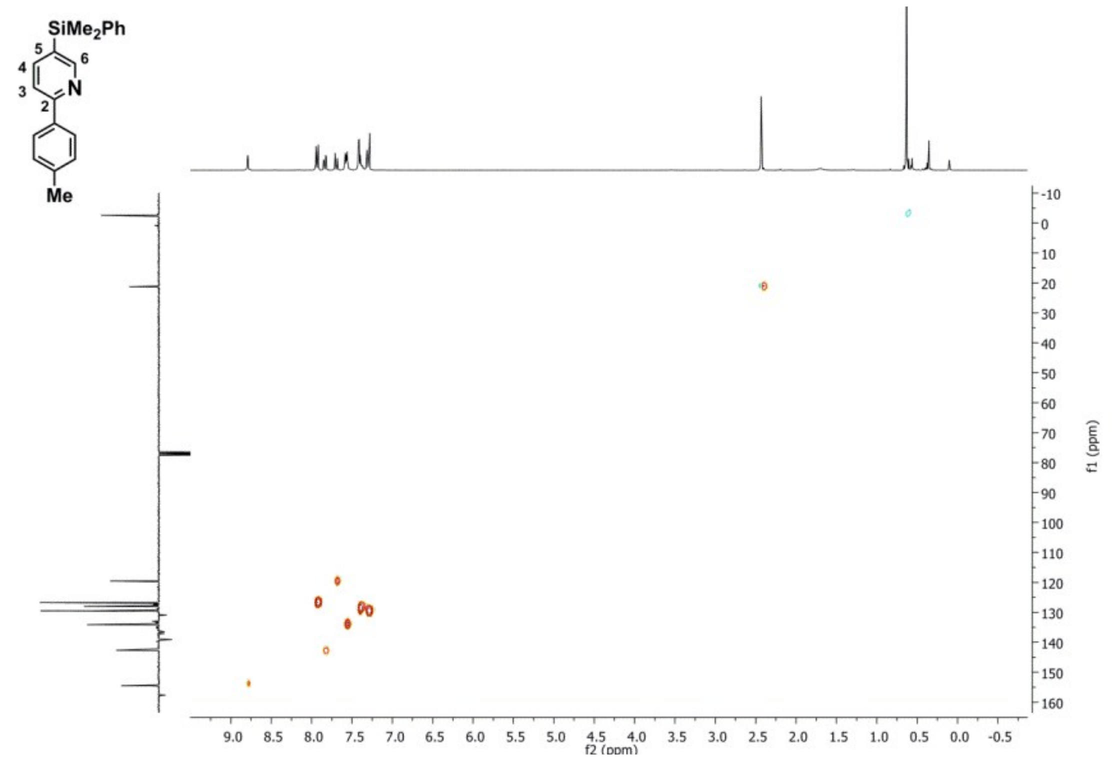

^{1}H-^{13}C HMBC

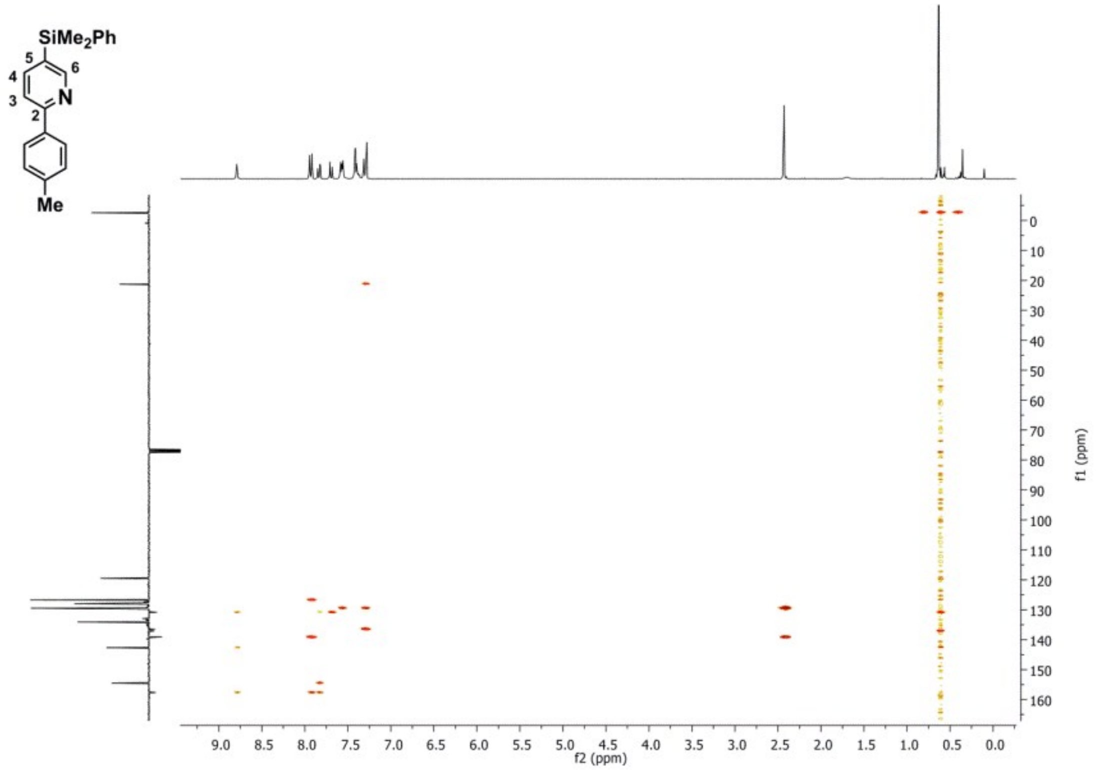

^{1}H-^{29}Si HMBC

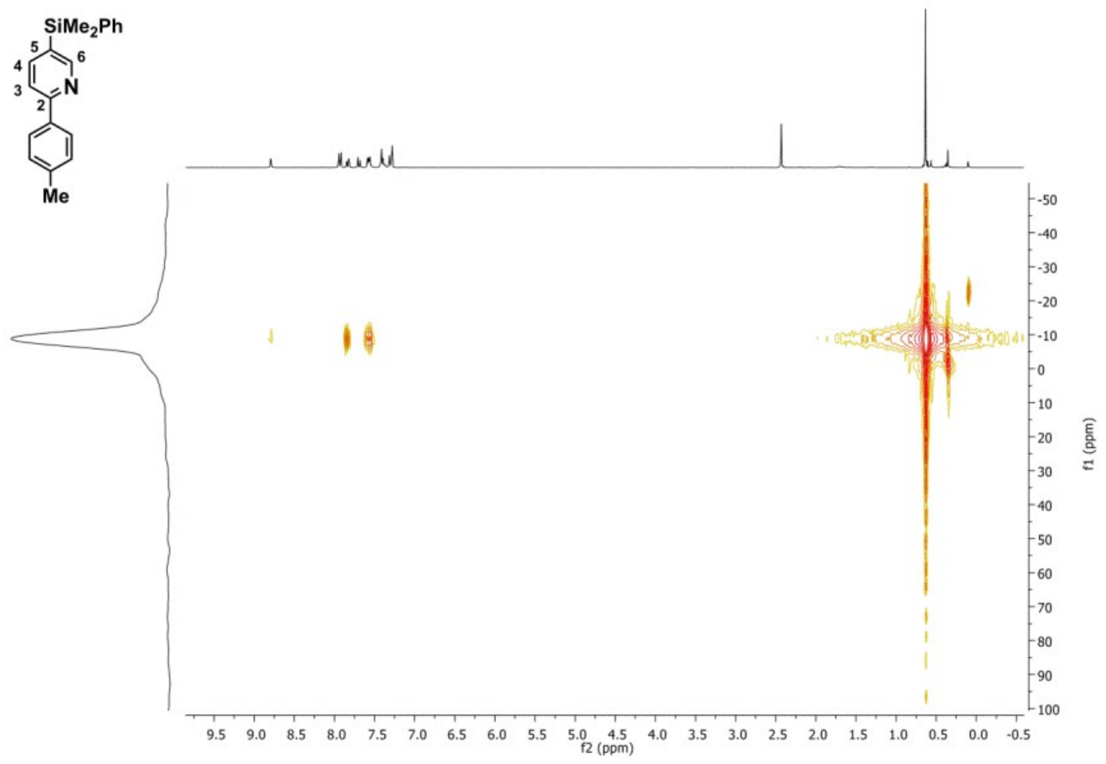

5-(methyldiphenylsilyl)-2-(p-tolyl)pyridine

^{1}H NMR

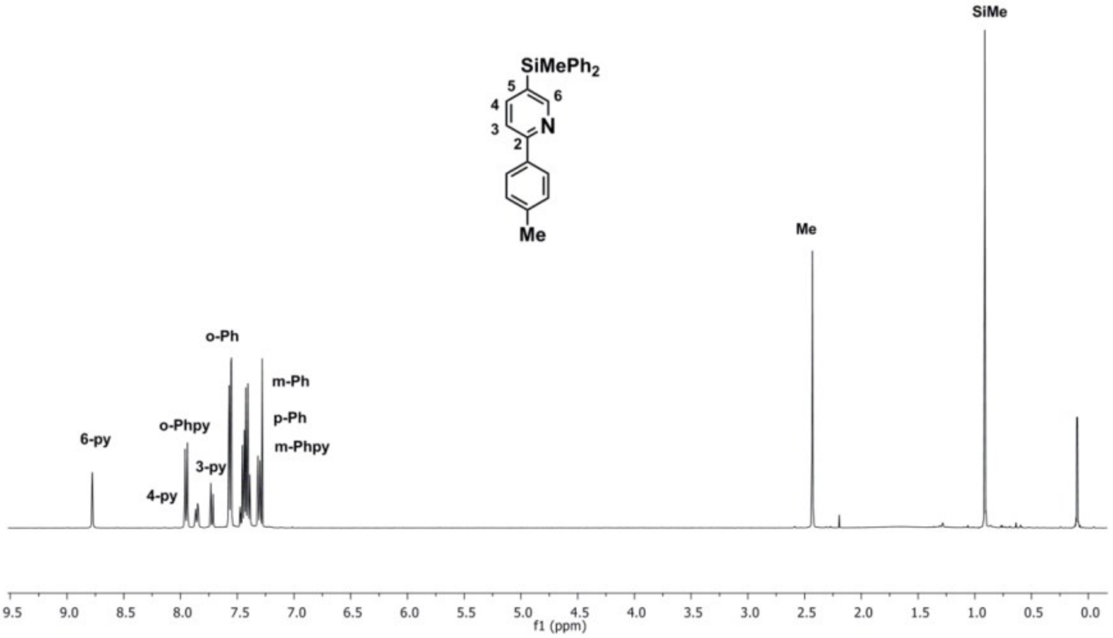

1

[3]C NMR

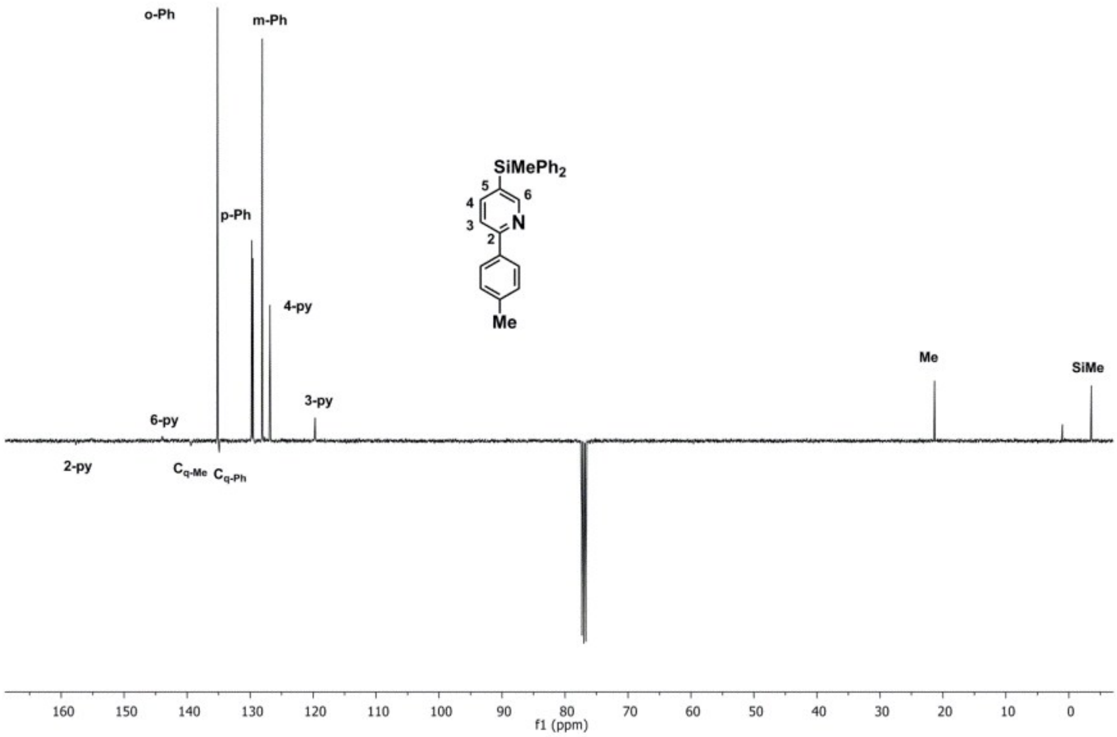

^{1}H-^{29}Si HMBC

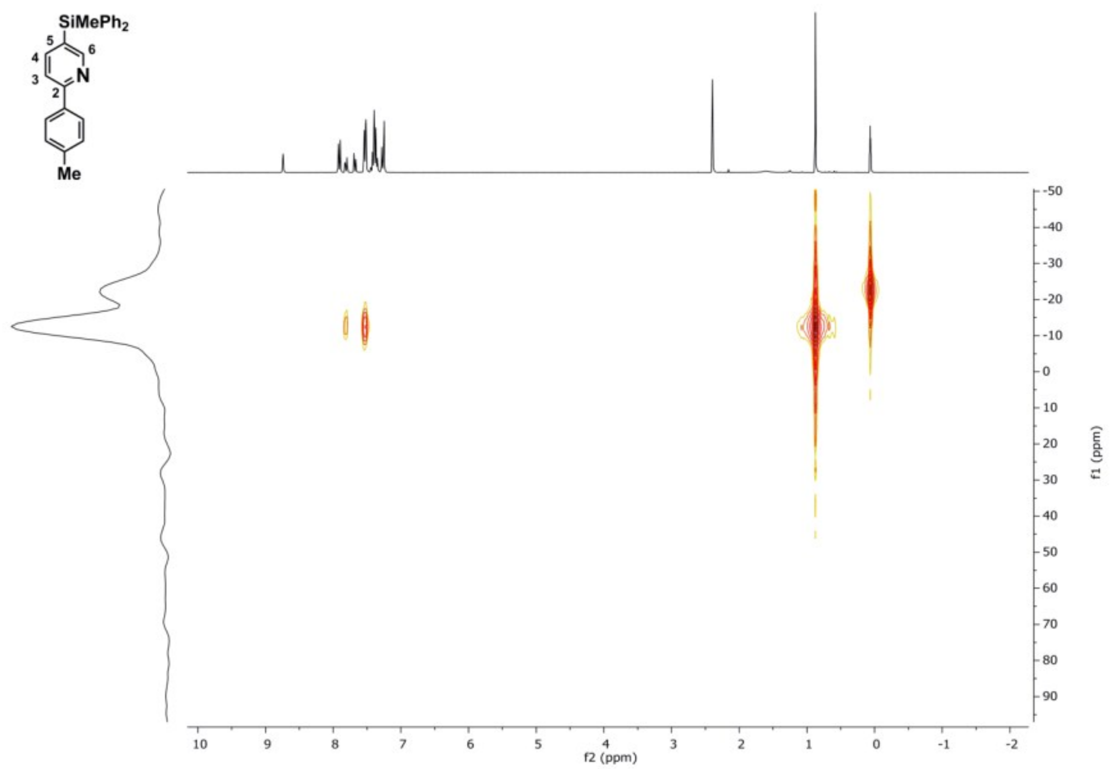

Methyltriphenylsilane

^{1}H-NMR

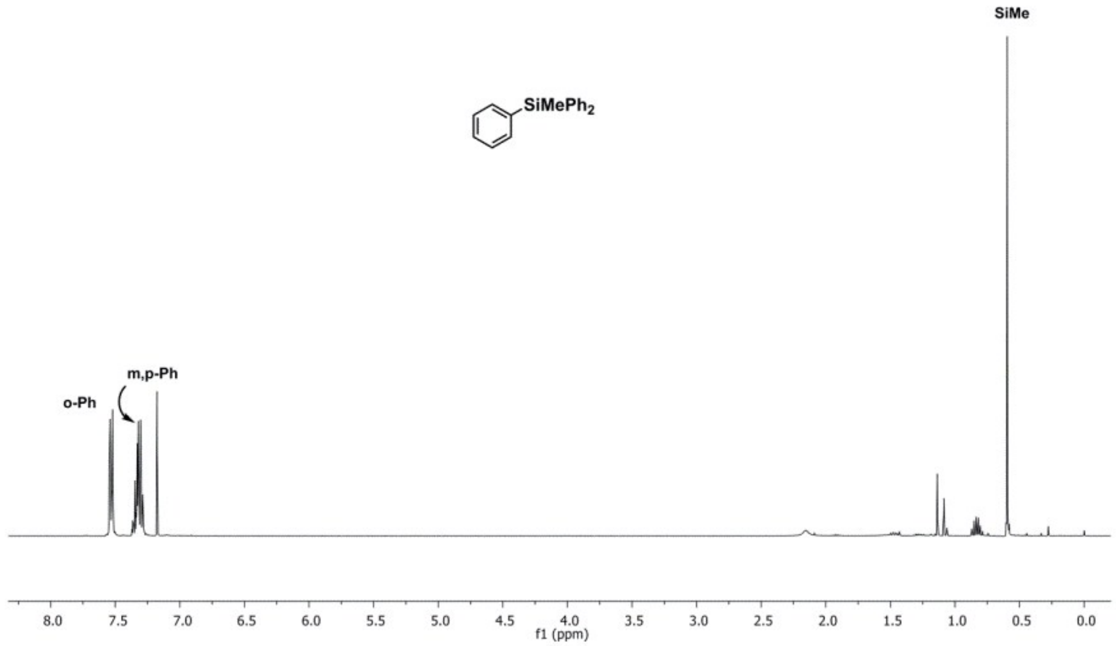

^{13}C NMR

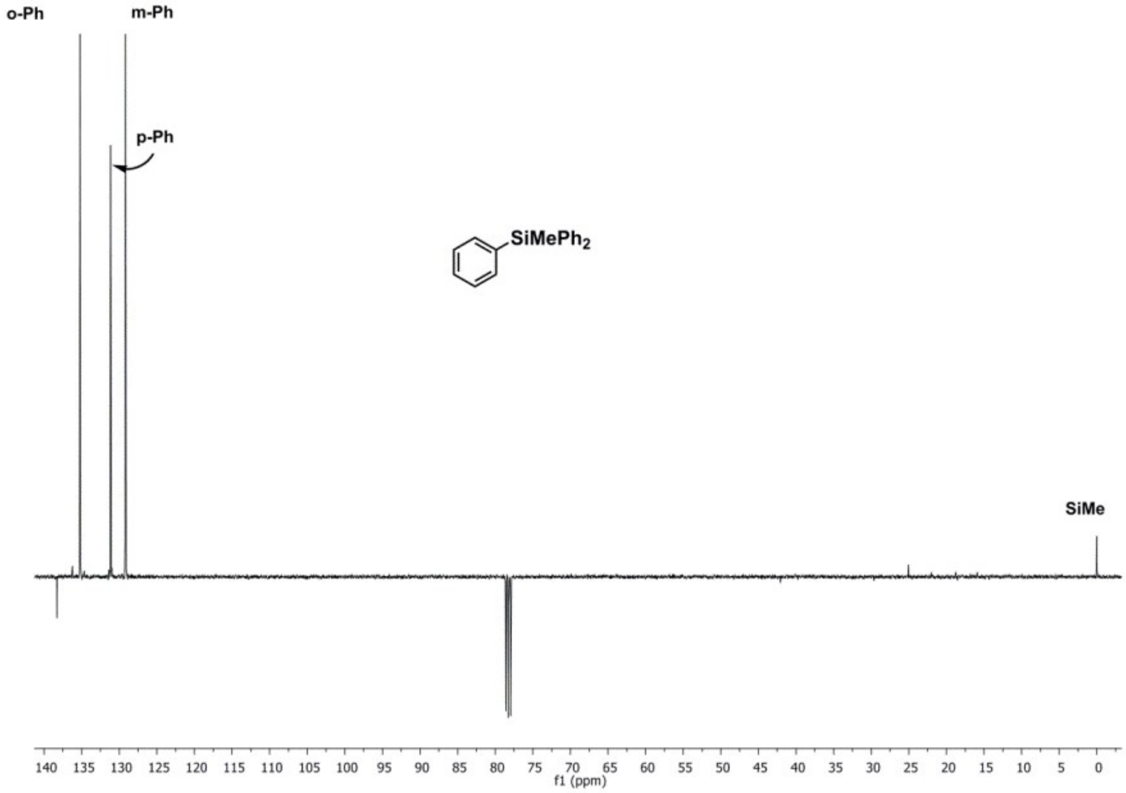

^{1}H-^{1}H-COSY

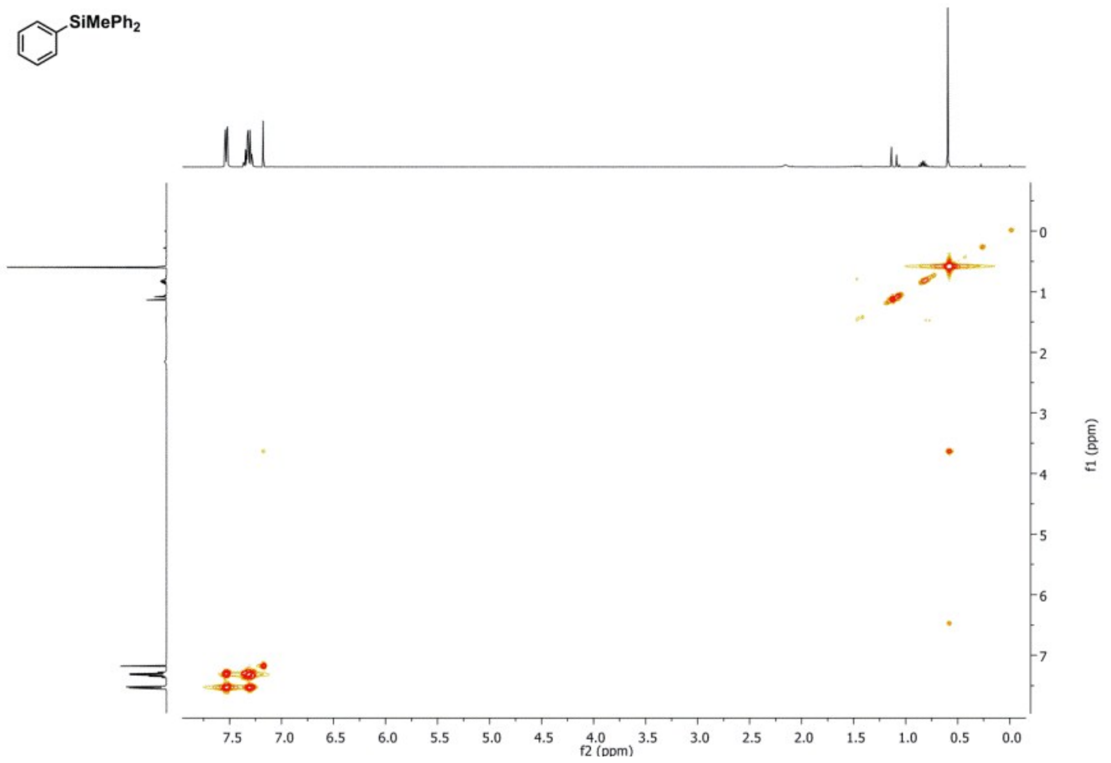

^{1}H-^{13}C HSQC

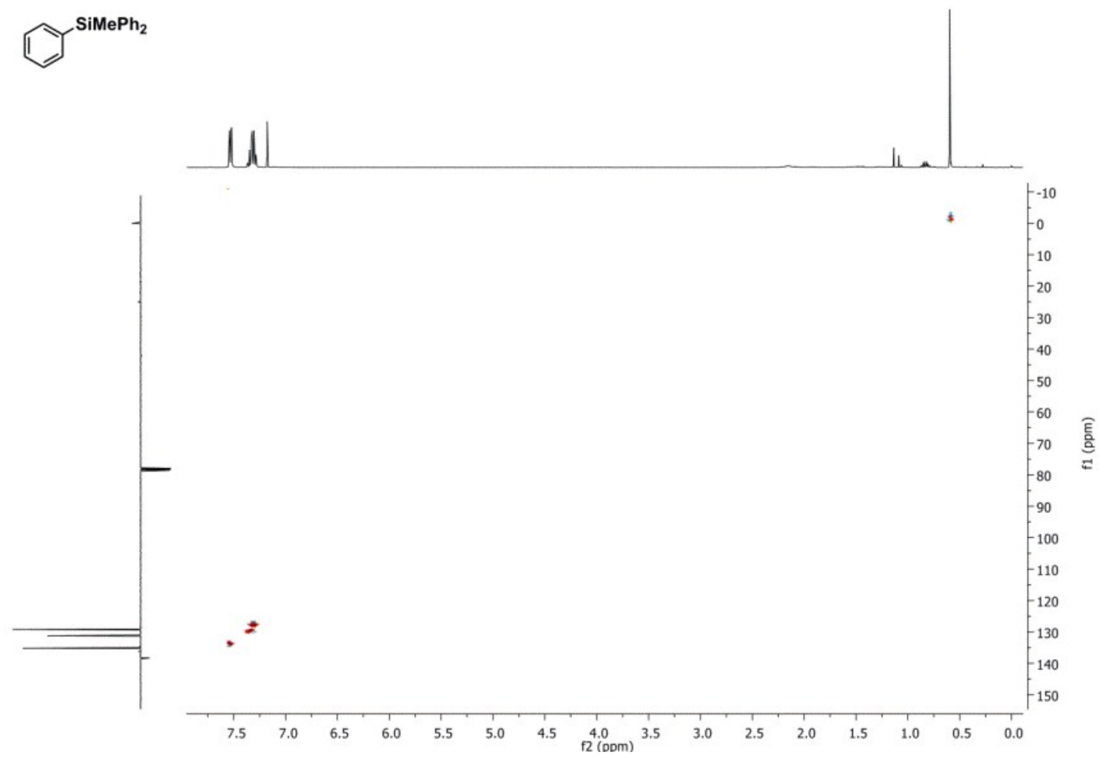

^{1}H-^{13}C HMBC

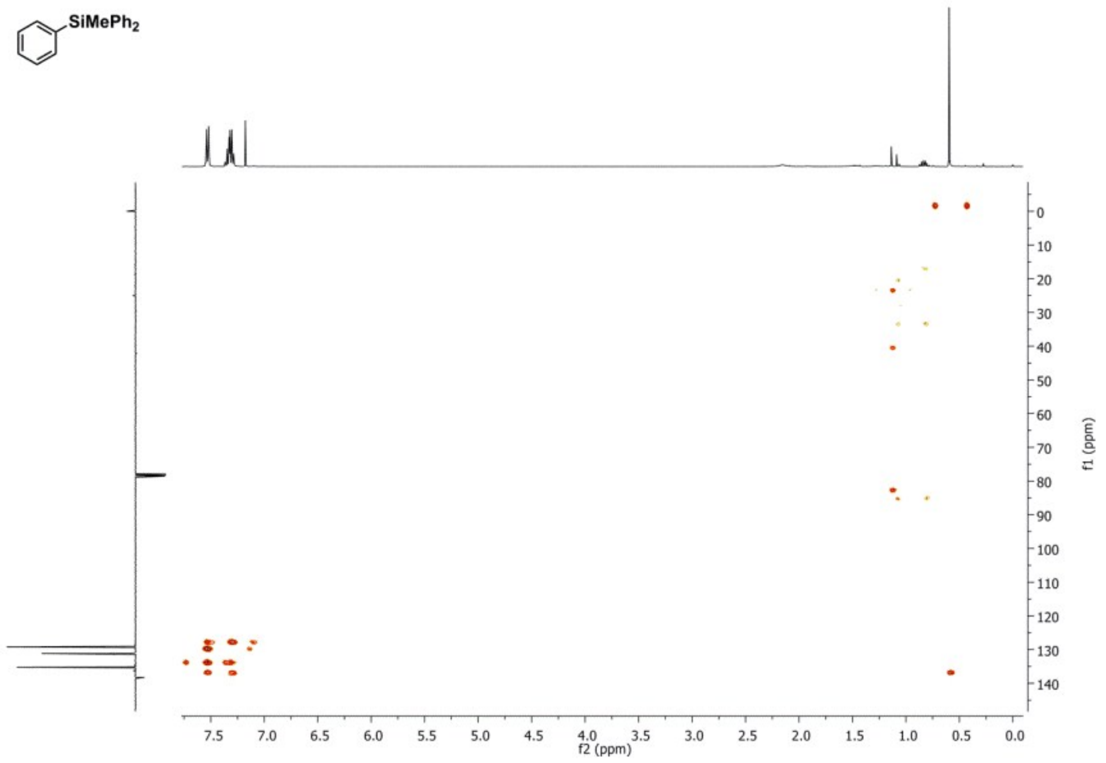

^{1}H-^{29}Si HMBC

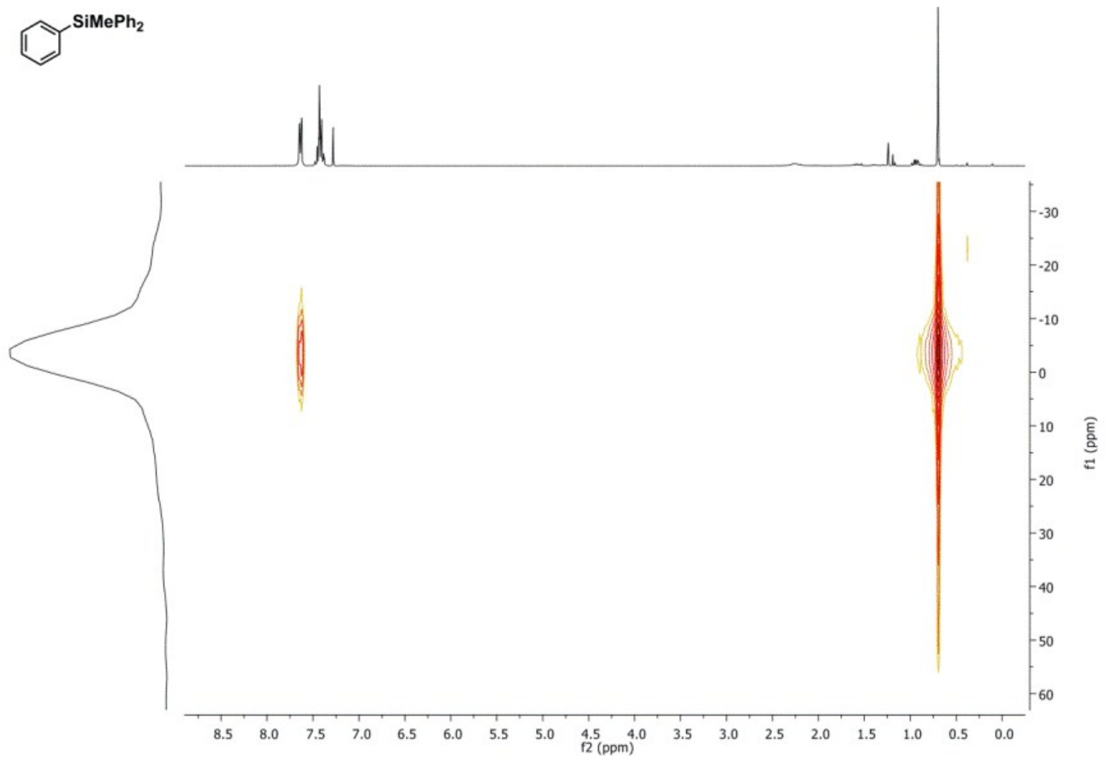

Dimethyldiphenylsilane

^{1}H NMR

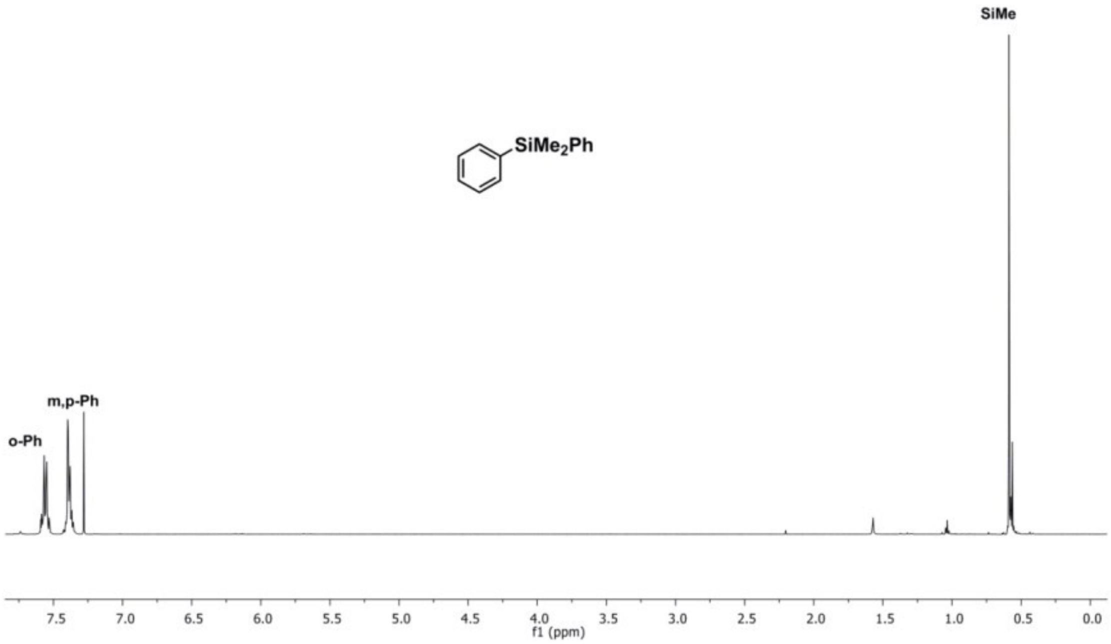

^{13}C NMR

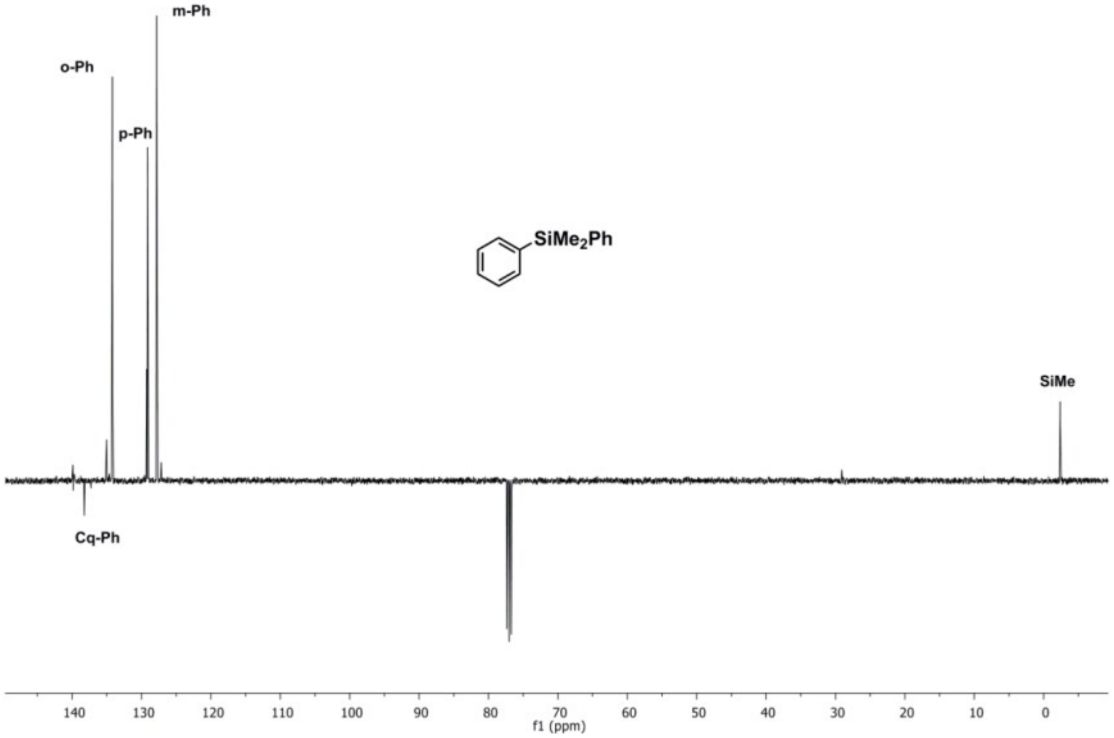

^{1}H-^{29}Si HMBC

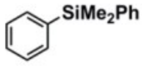

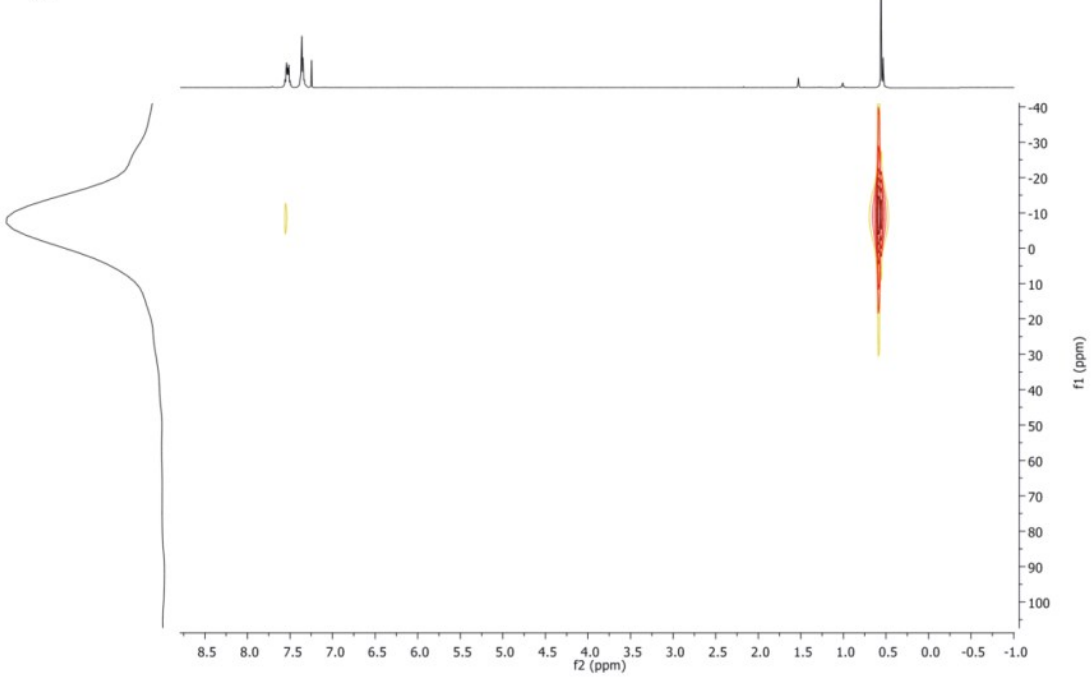

Tetraphenylsilane

^{1}H NMR

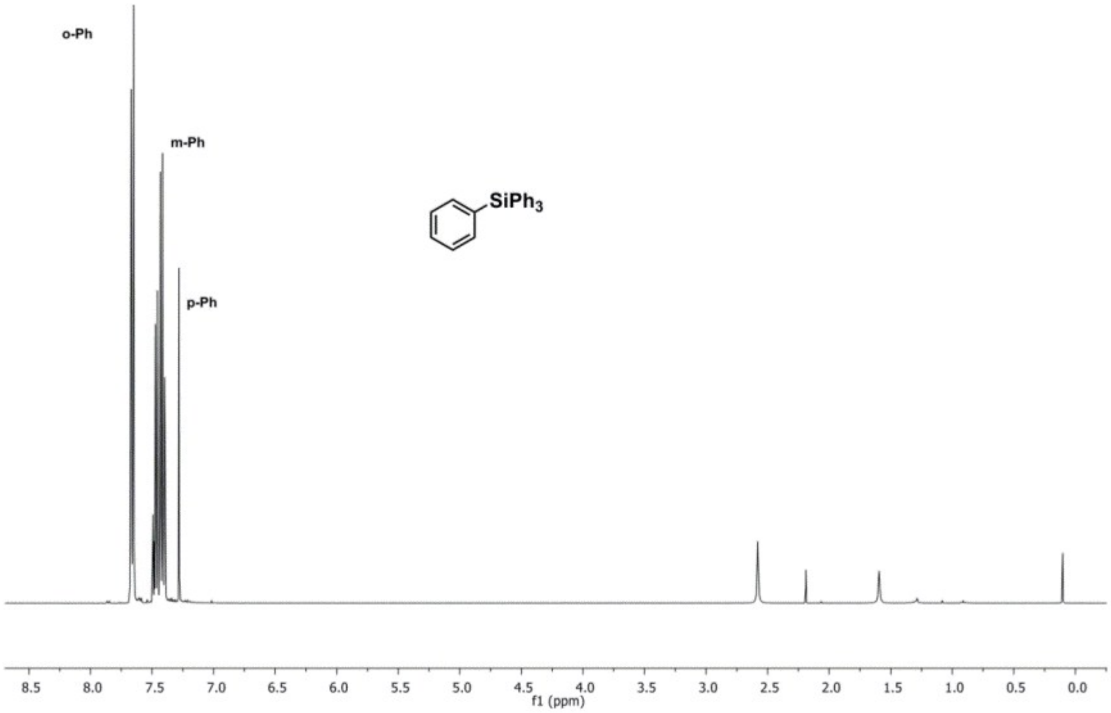

^{13}C NMR

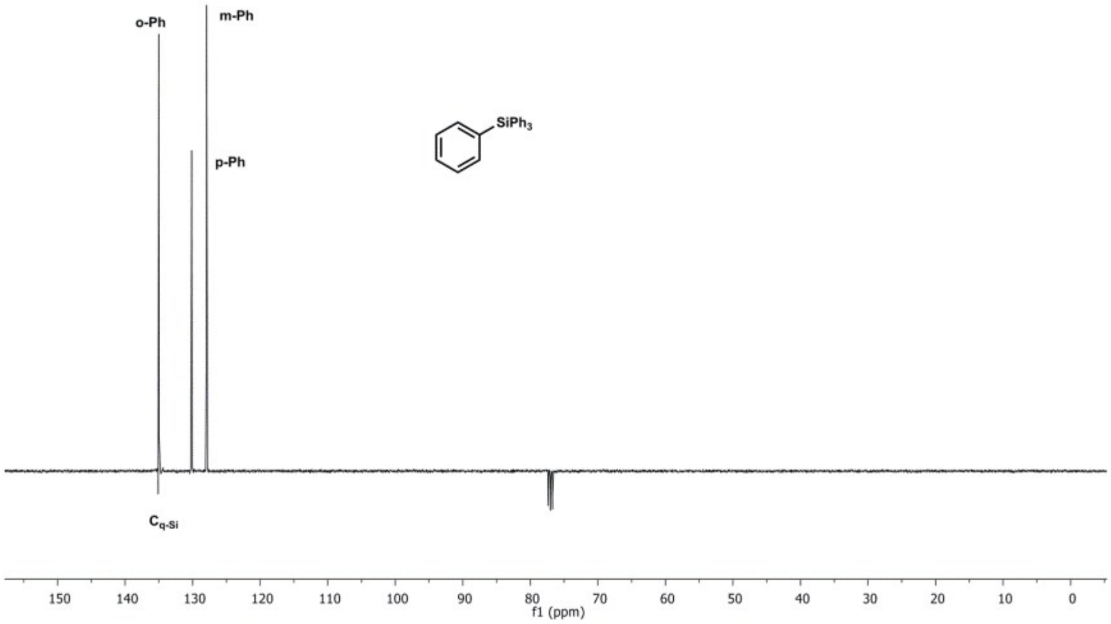

^{1}H-^{29}Si HMBC

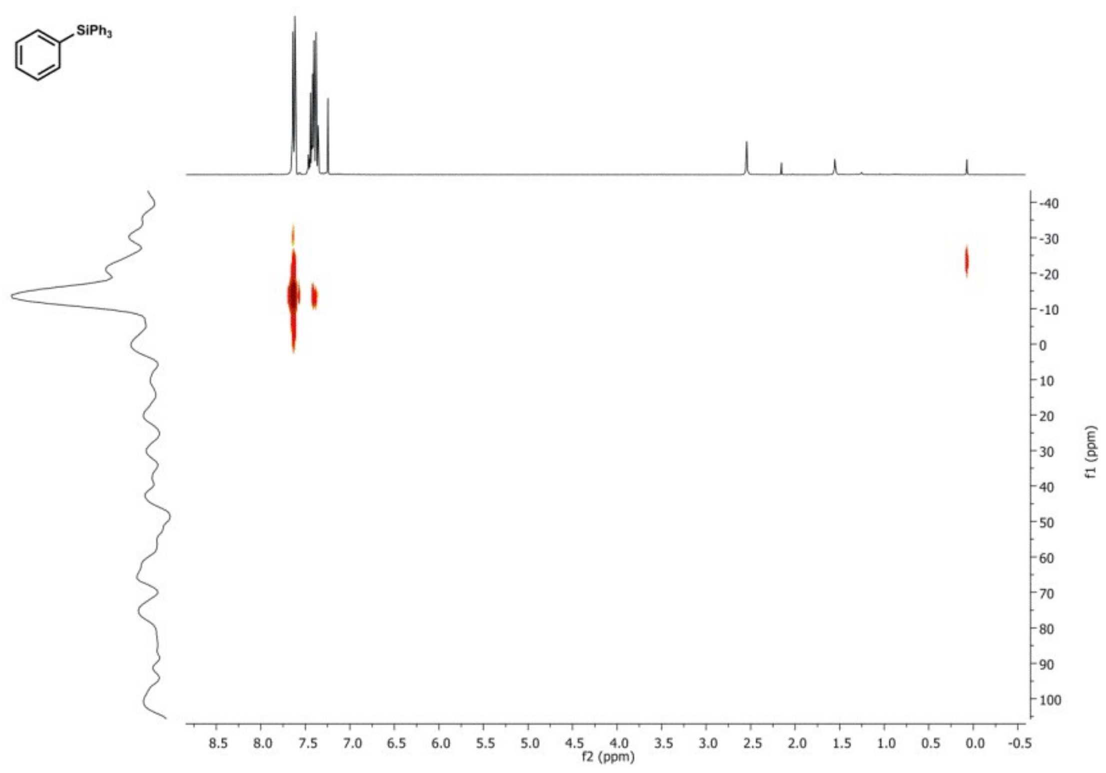

triethyl(thiophen-2-yl)silane

^{1}H NMR

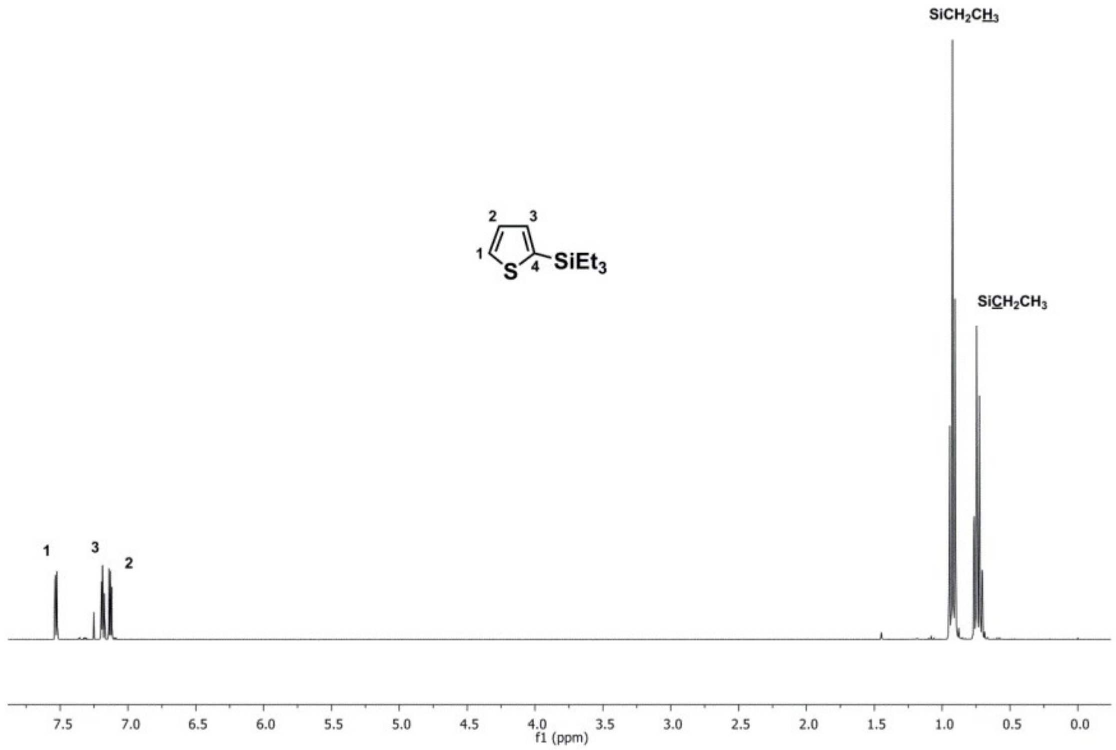

^{13}C NMR

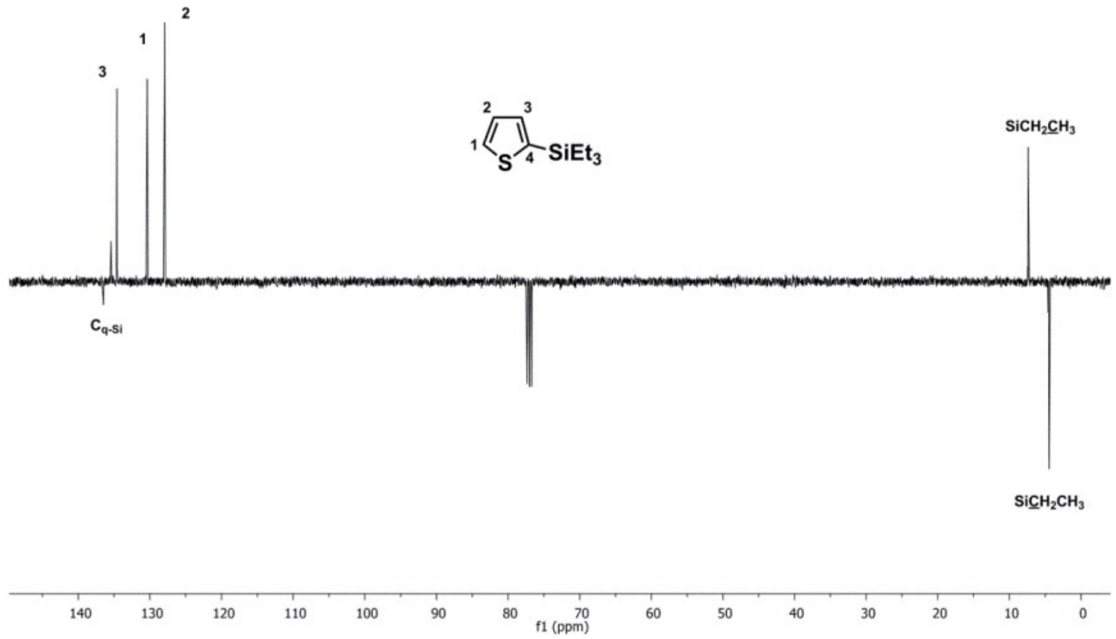

^{1}H-^{29}Si HMBC

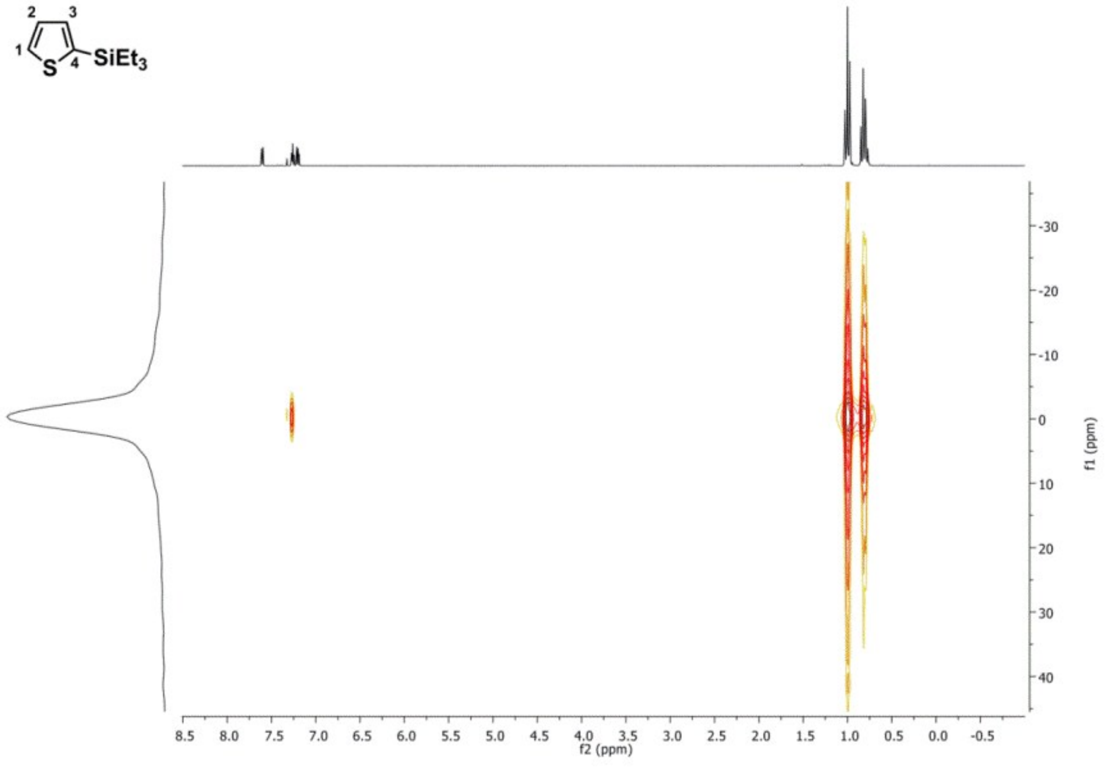

ethyl(5-methylthiophen-2-yl)-l2-silane

^{1}H NMR

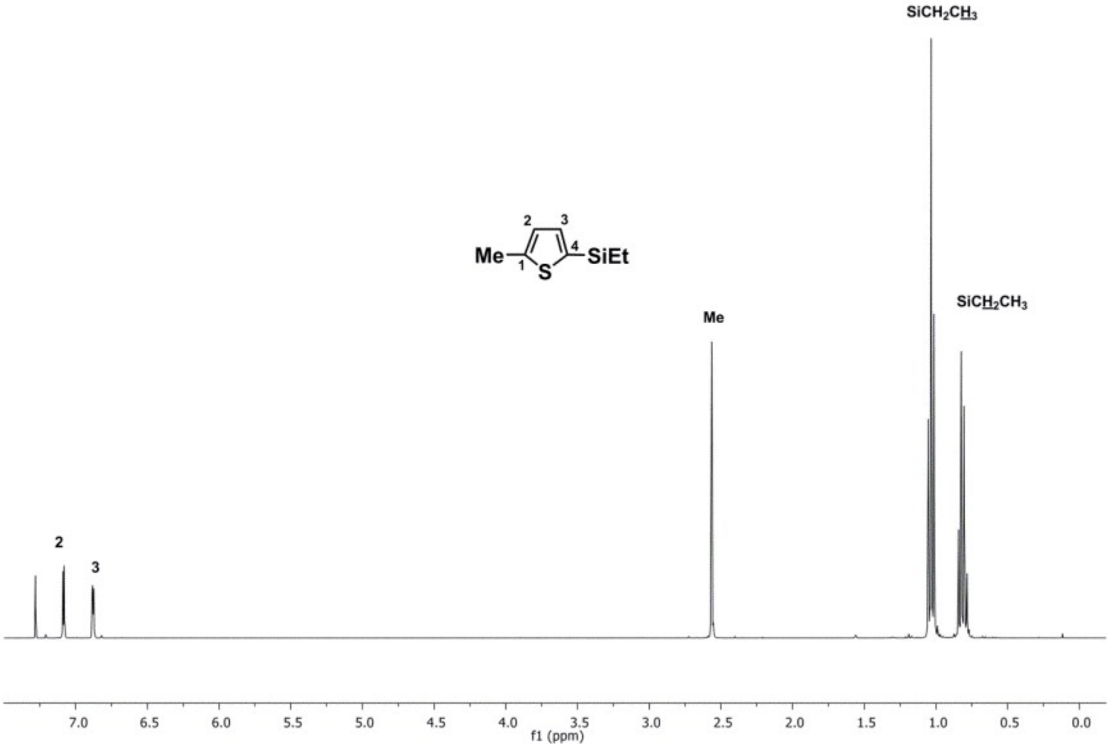

^{13}C NMR

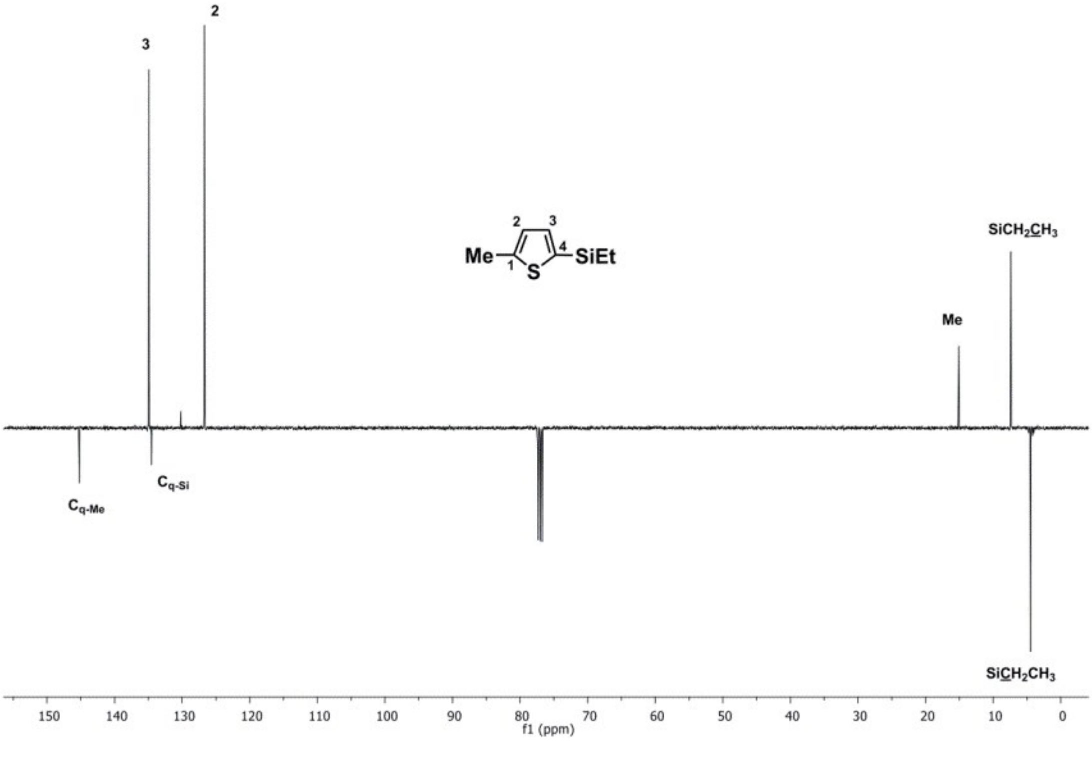

^1H-^{29}Si HMBC

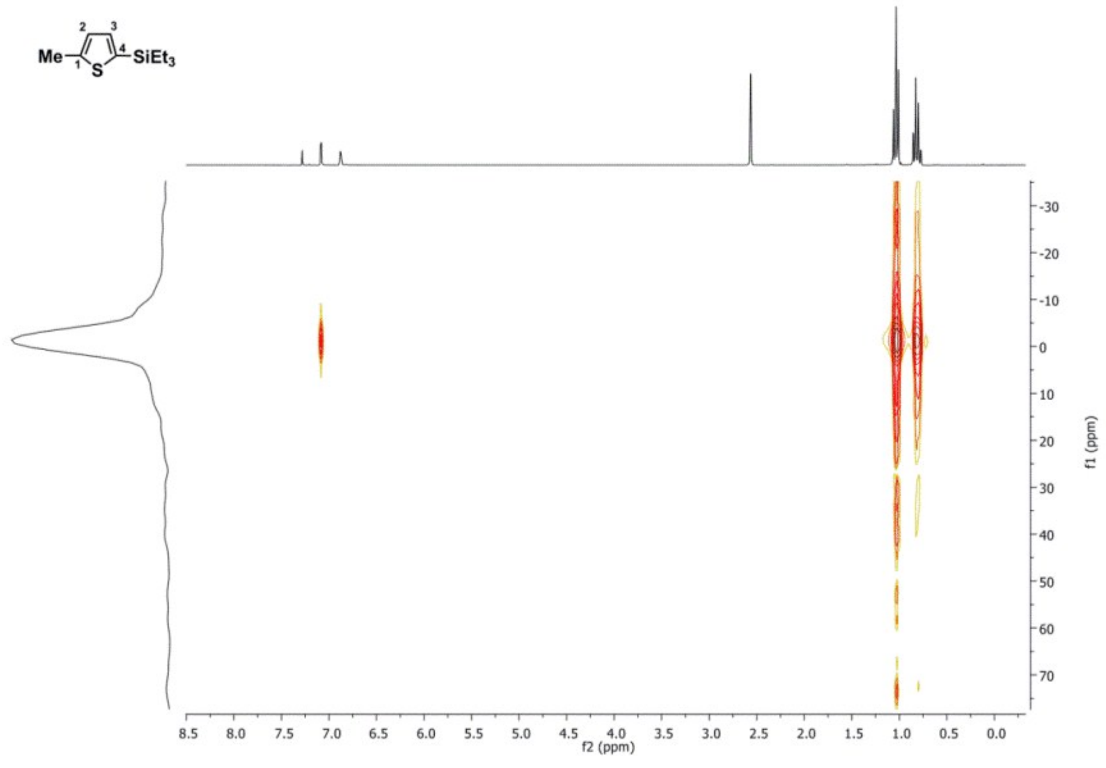

methyl(5-methylthiophen-2-yl)diphenylsilane

^{1}H NMR

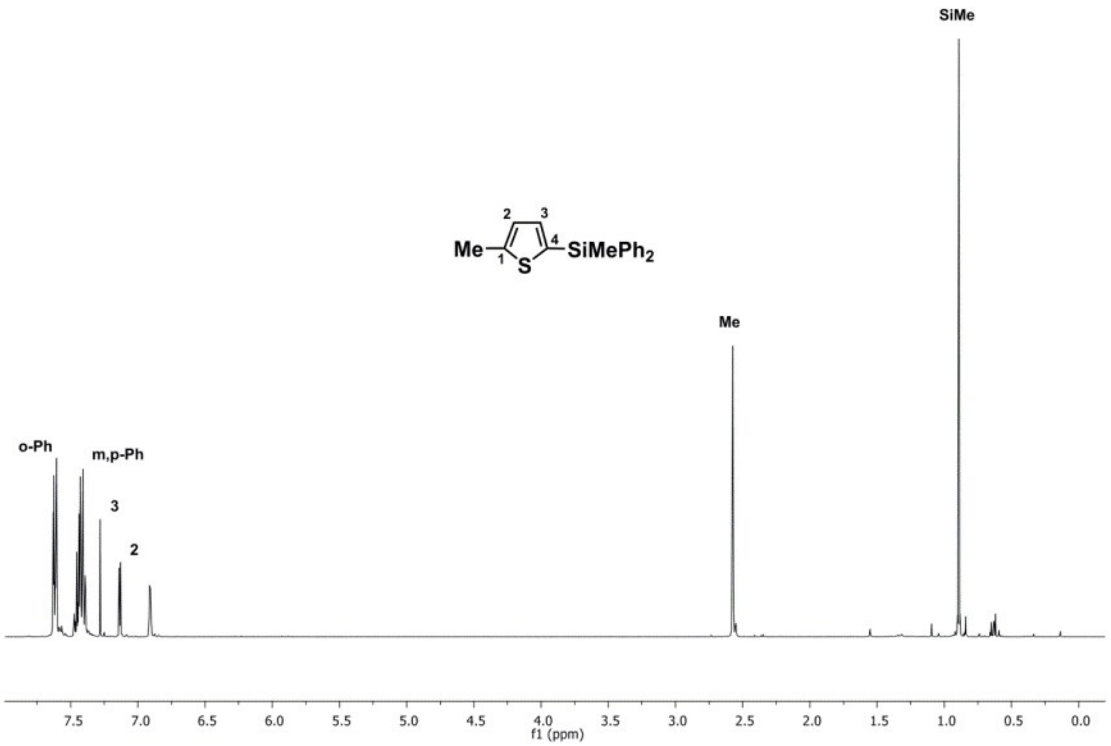

^{13}C NMR

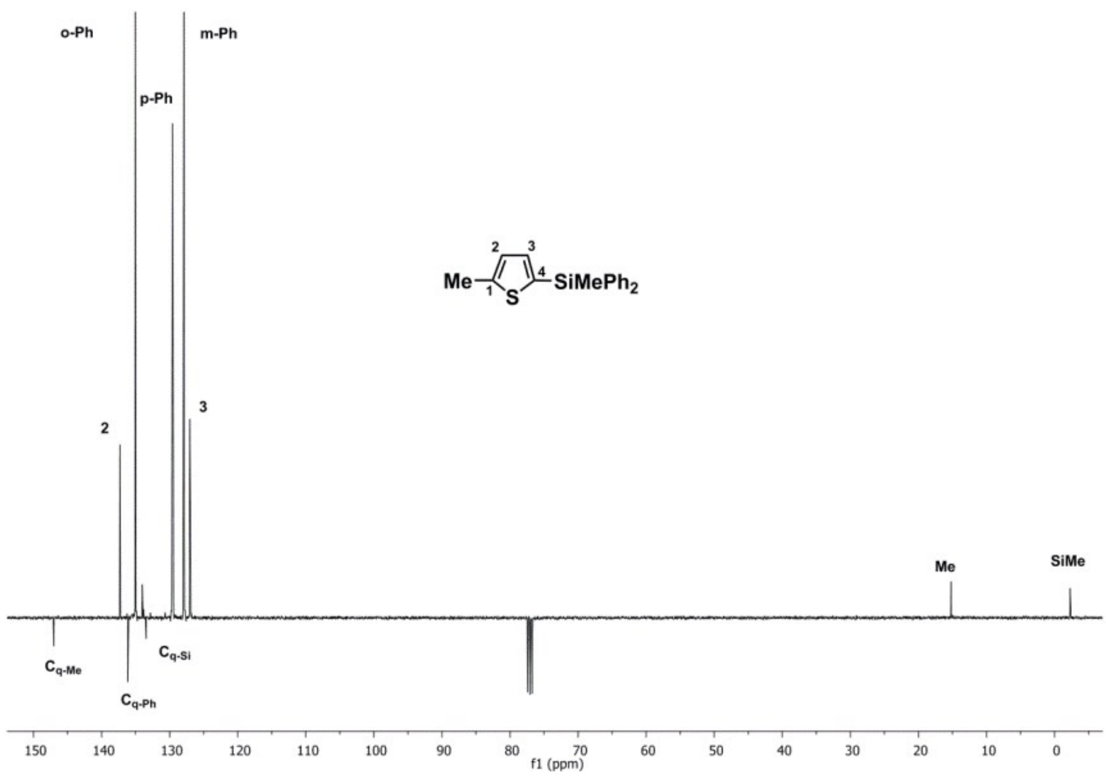

^{1}H-^{29}Si HMBC

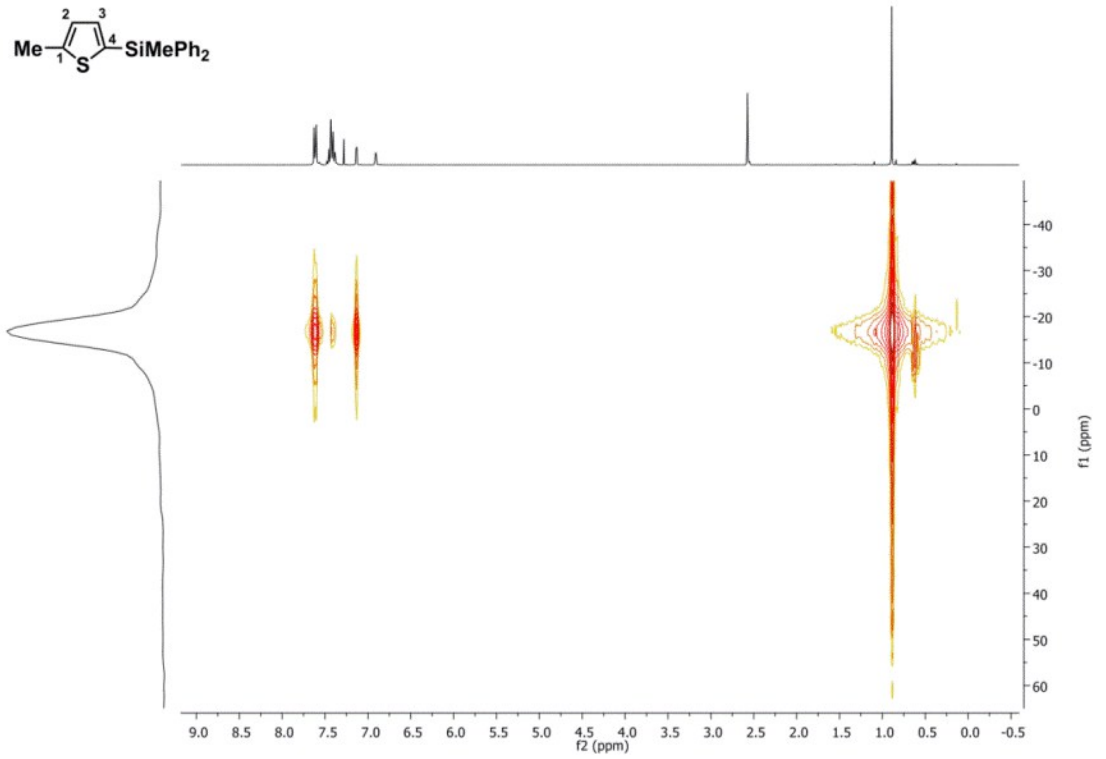

Me
2
3
4
1
S
SiMePh2
-40
-30
-20
-10
0
10
20
30
40
50
60
f1 (ppm)
9.0 8.5 8.0 7.5 7.0 6.5 6.0 5.5 5.0 4.5 4.0 3.5 3.0 2.5 2.0 1.5 1.0 0.5 0.0 -0.5
f2 (ppm)

benzo[b]thiophen-2-yltriethylsilane

^{1}H NMR

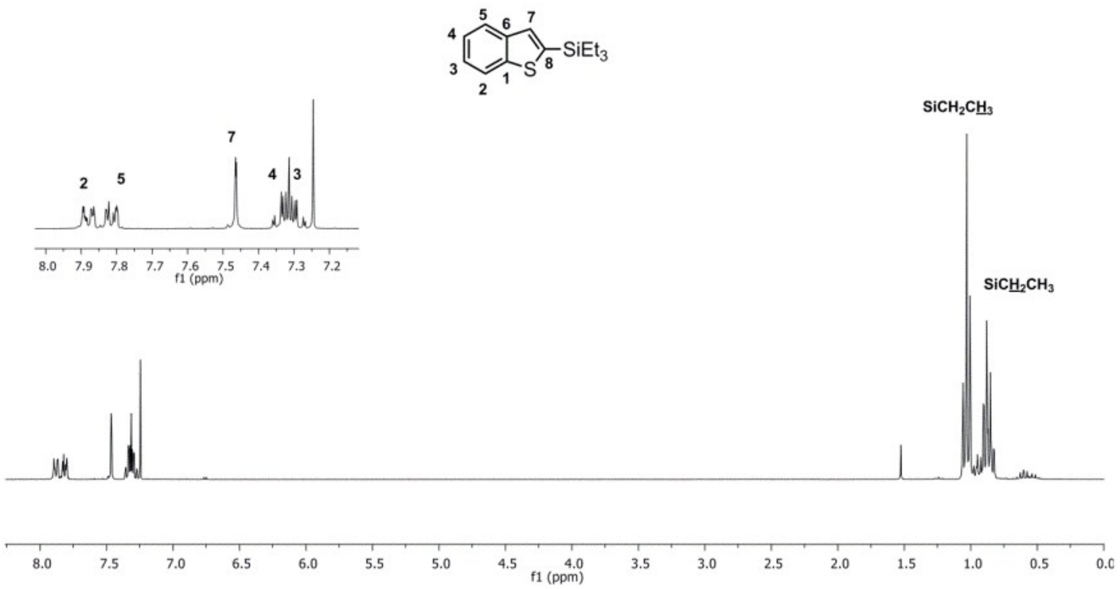

^{13}C NMR

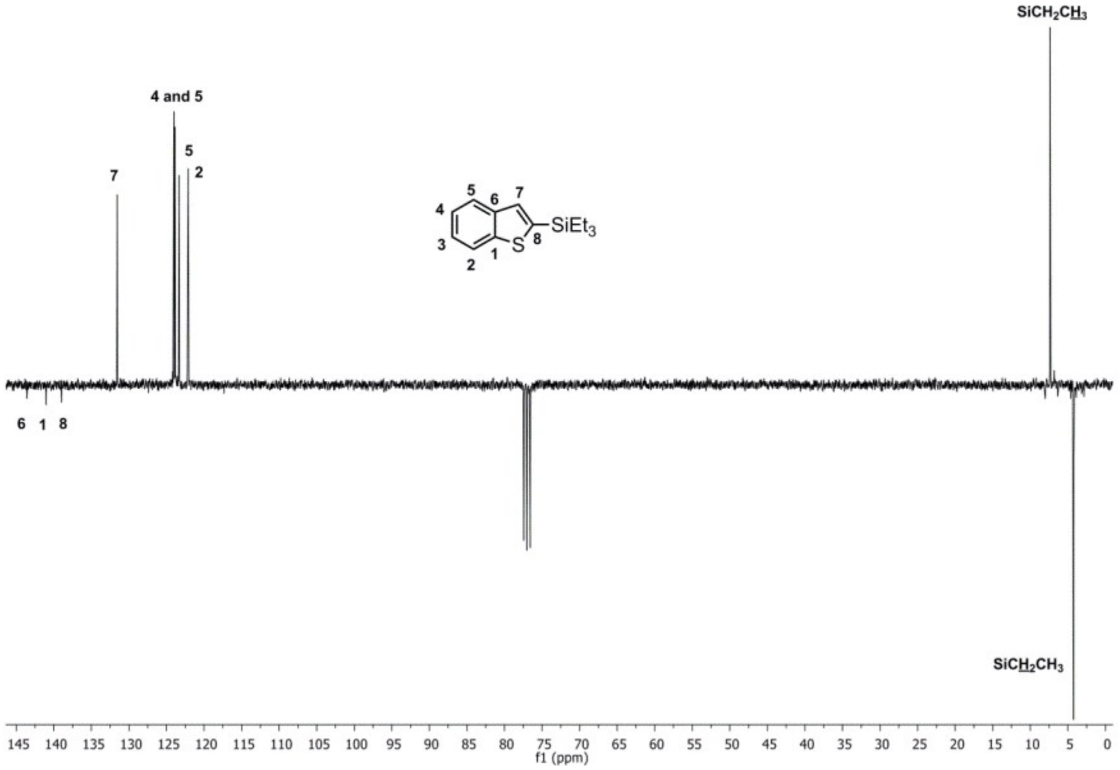

^{1}H-^{1}H COSY

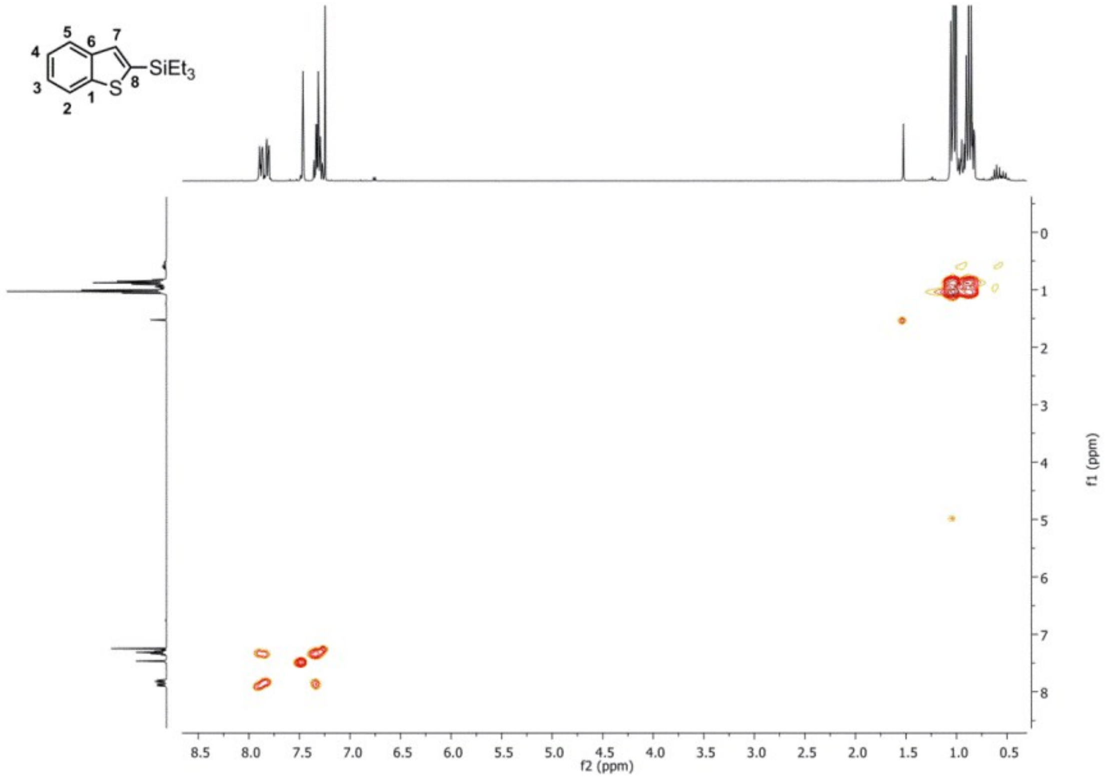

^{1}H-^{13}C HMBC

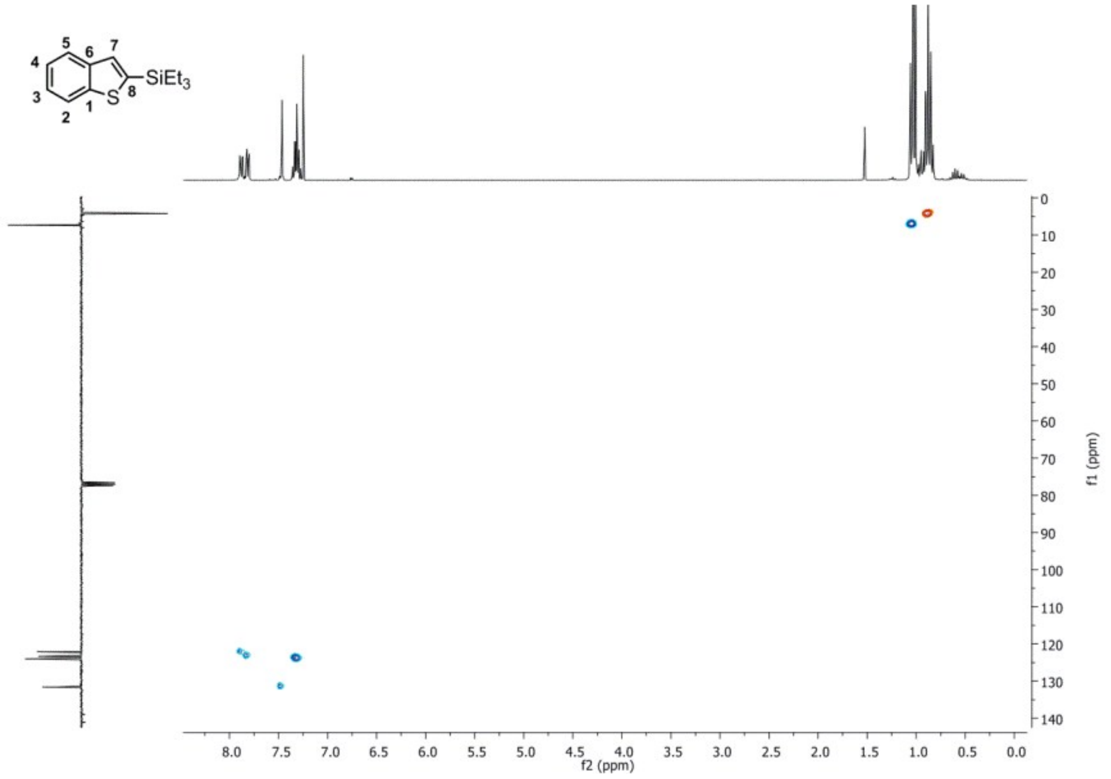

^{1}H-^{13}C HMBC

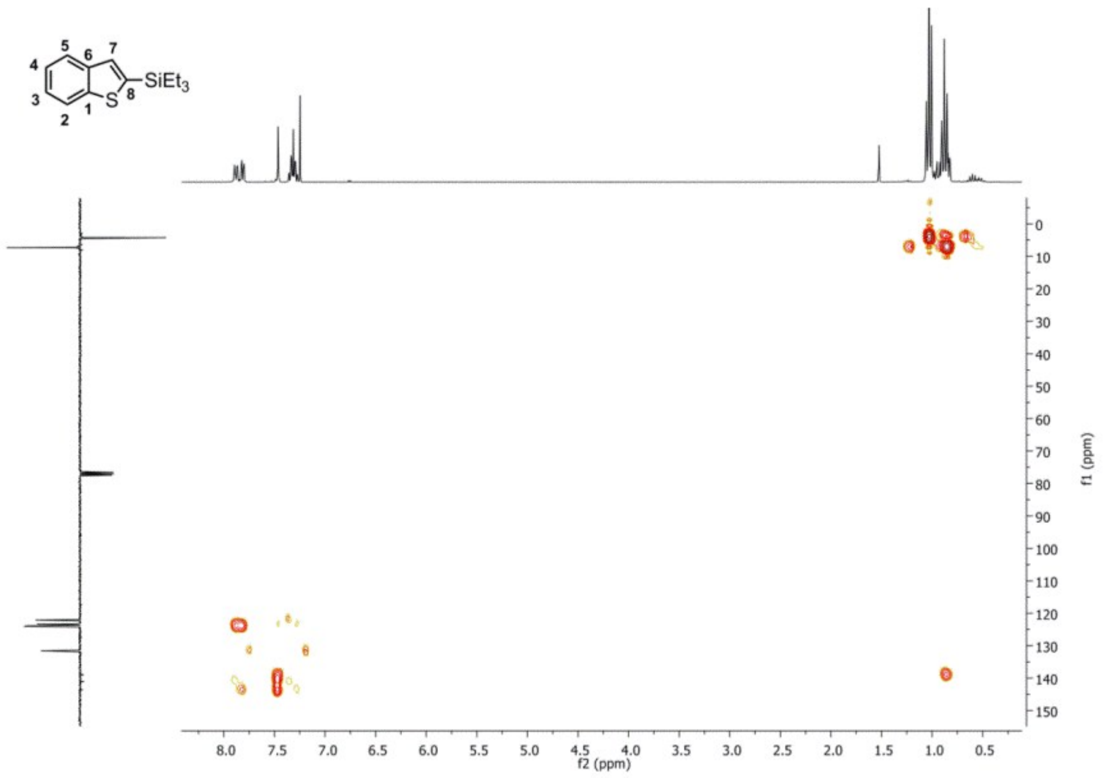

^{1}H-^{29}Si HMBC

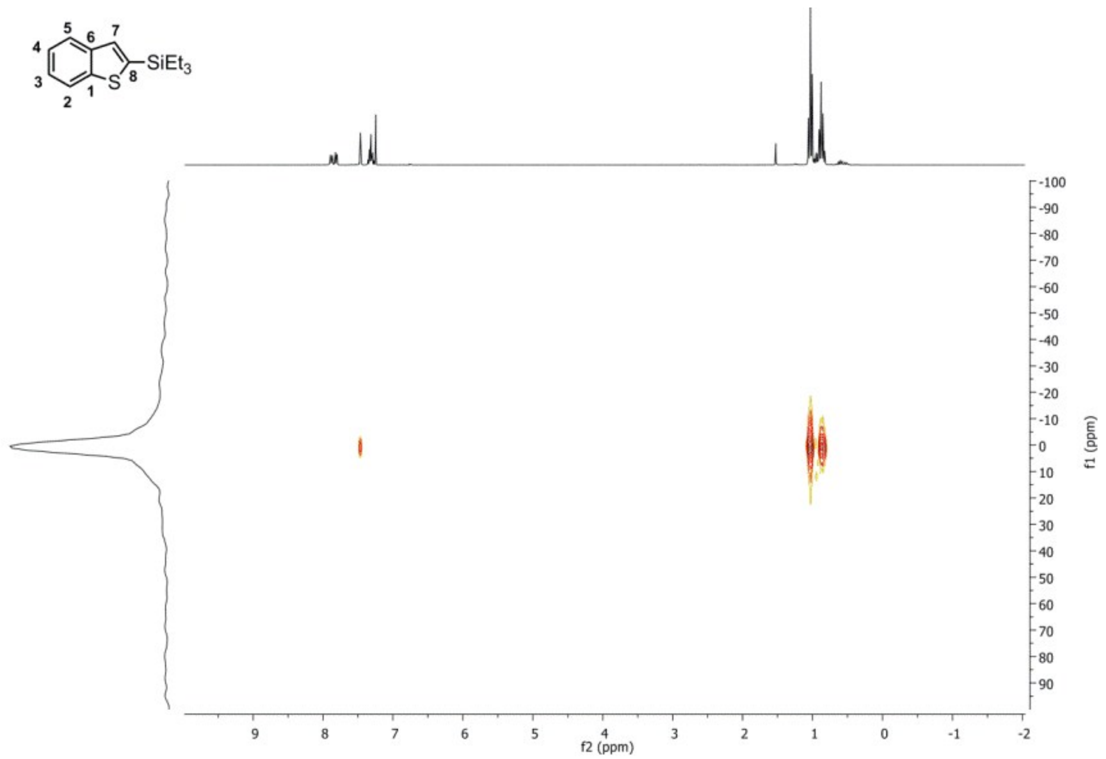

3,5-dimethylphenyl)triethylsilane

^{1}H NMR

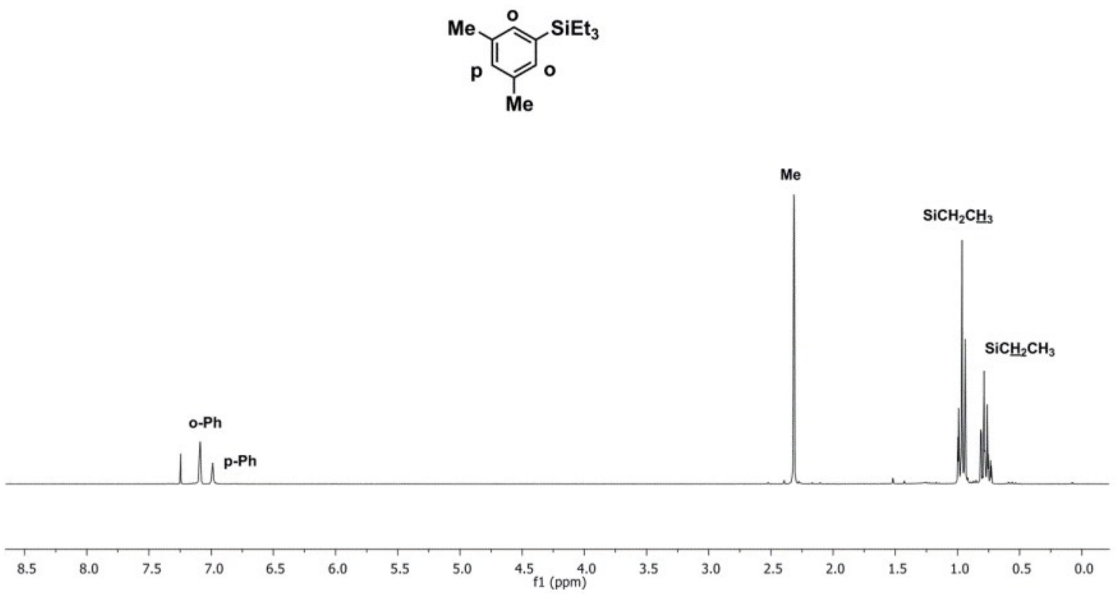

^{13}C-NMR

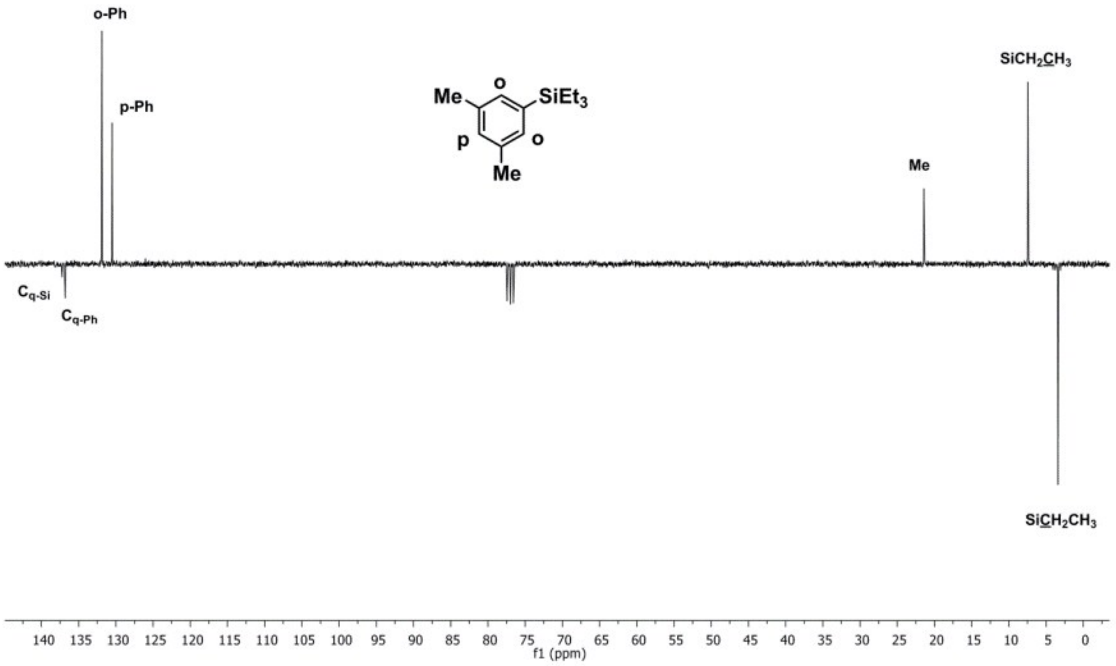

^1H-^{29}Si HMBC

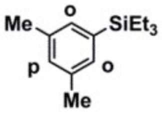

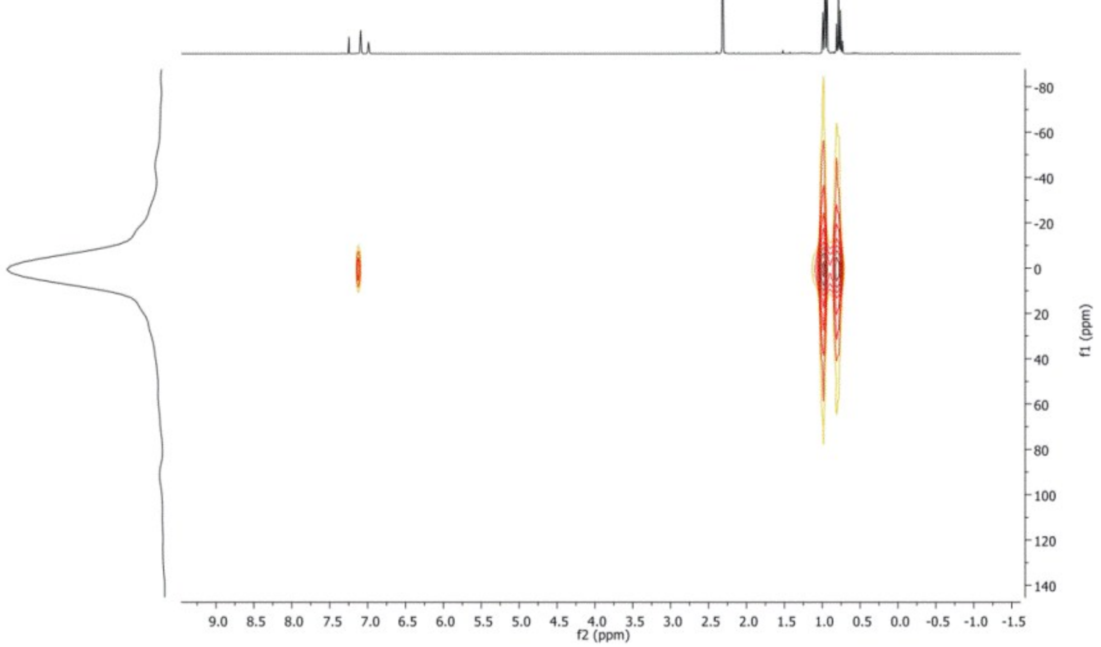

triethyl(naphthalen-2-yl)silane

^{1}H NMR

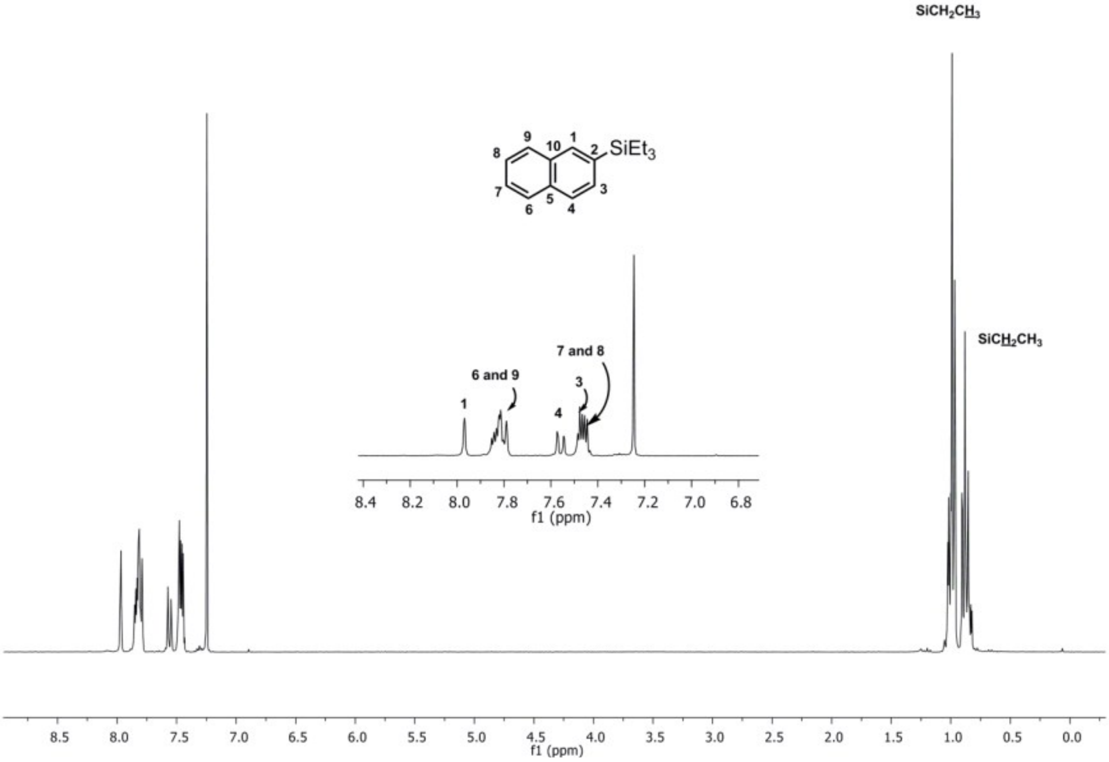

^{13}C NMR

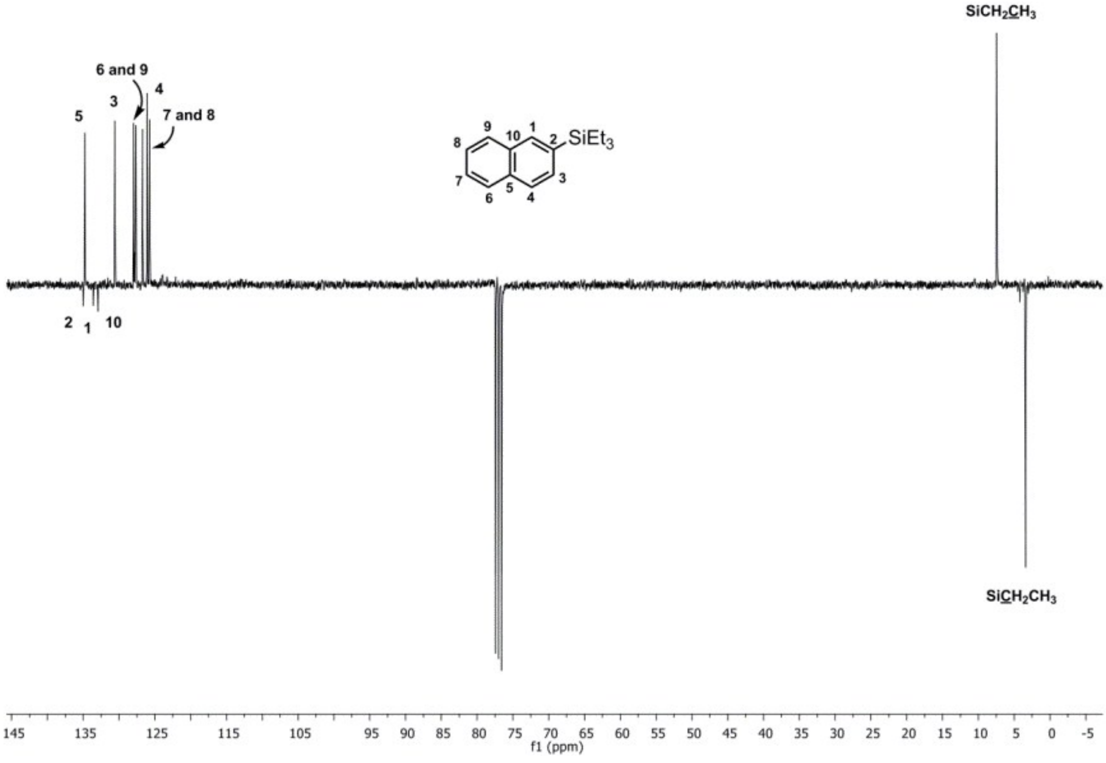

^{1}H-^{29}Si HMBC

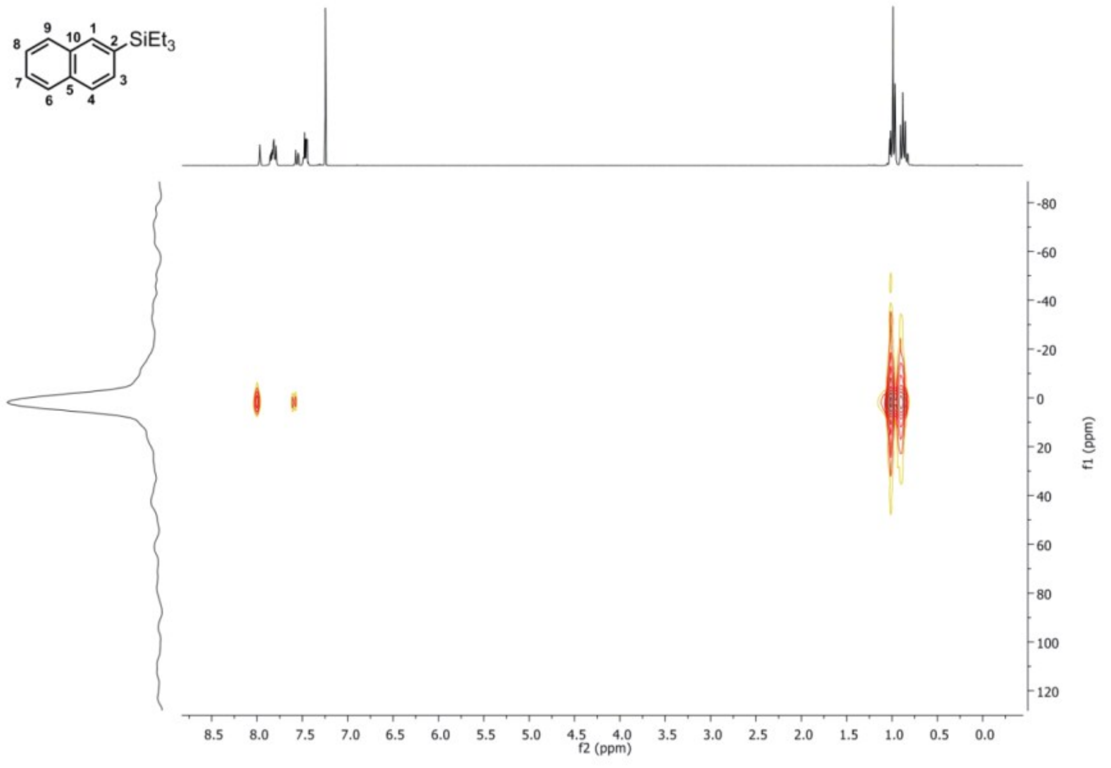

triethyl(fluorophenyl)silane

^{1}H NMR

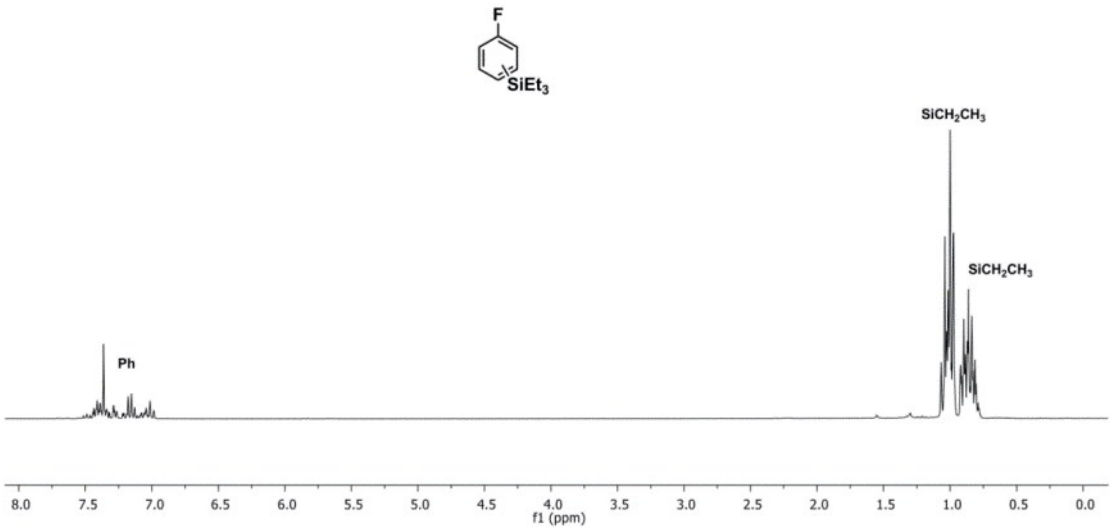

^{13}C NMR

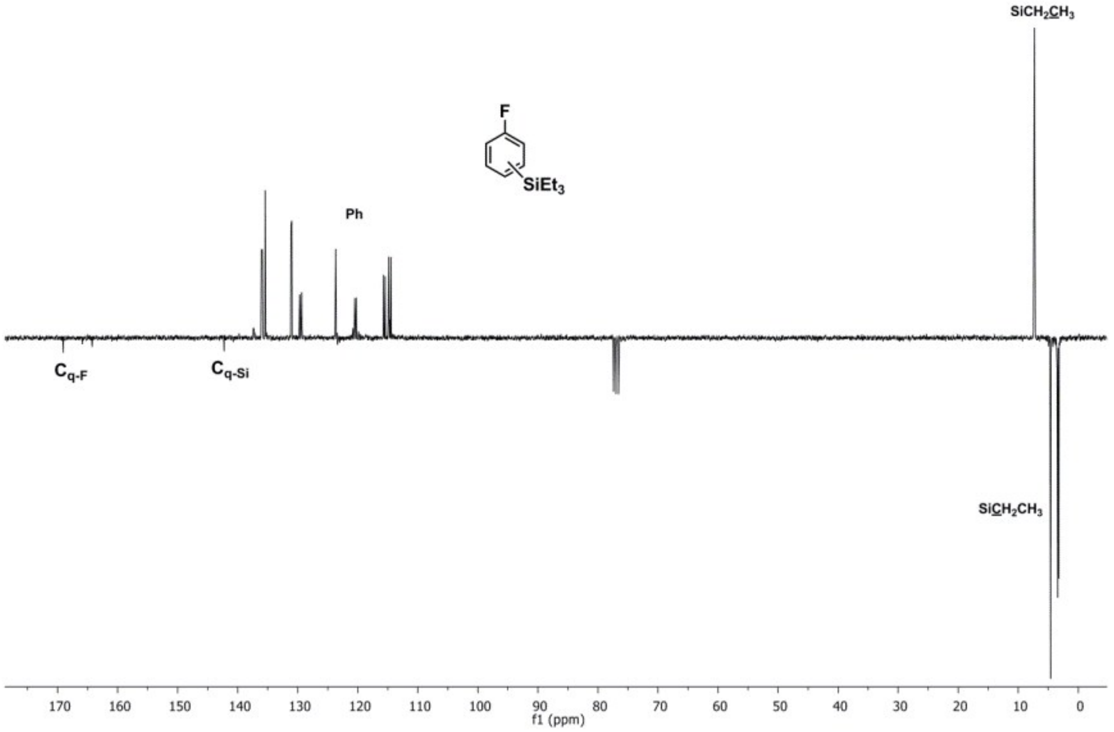

^1H-^{29}Si HMBC

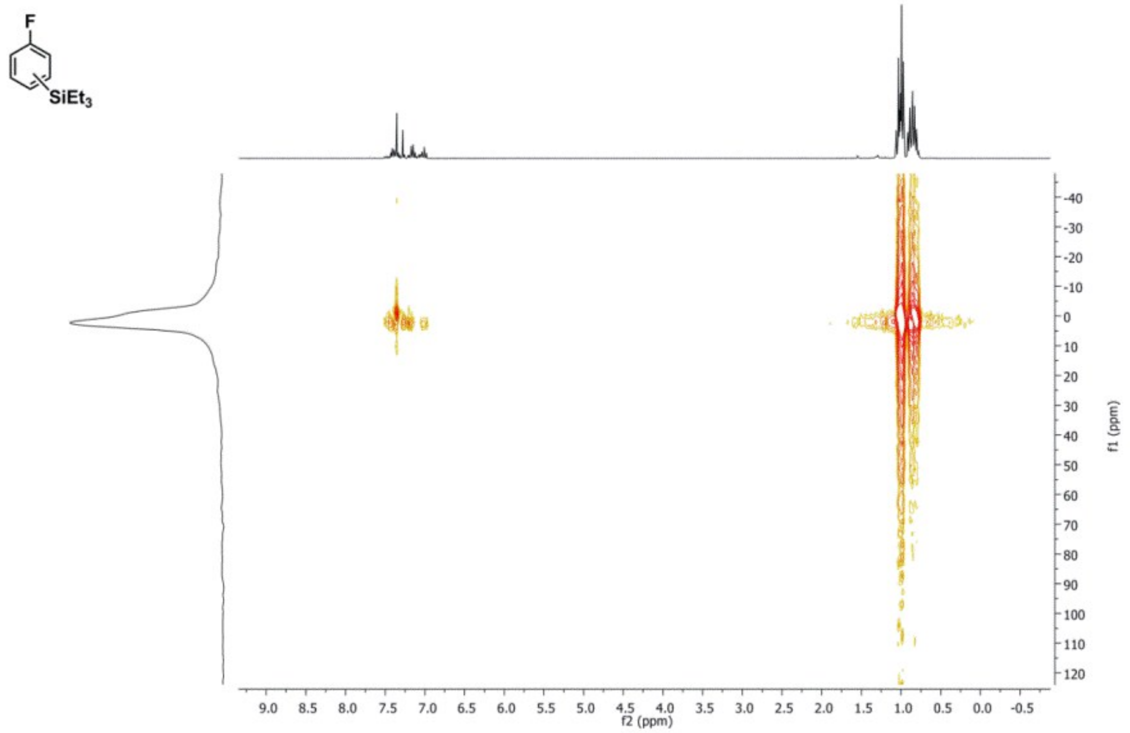

Chlorophenyltriethylsylane

^{1}H NMR

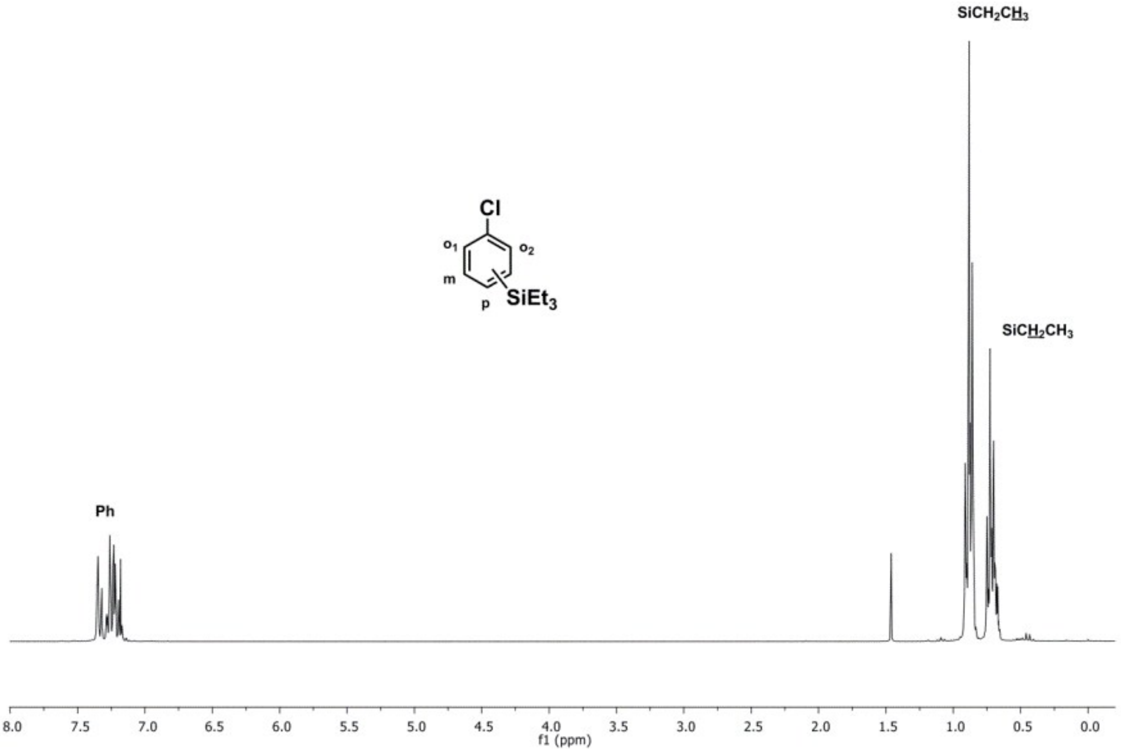

^{13}C NMR

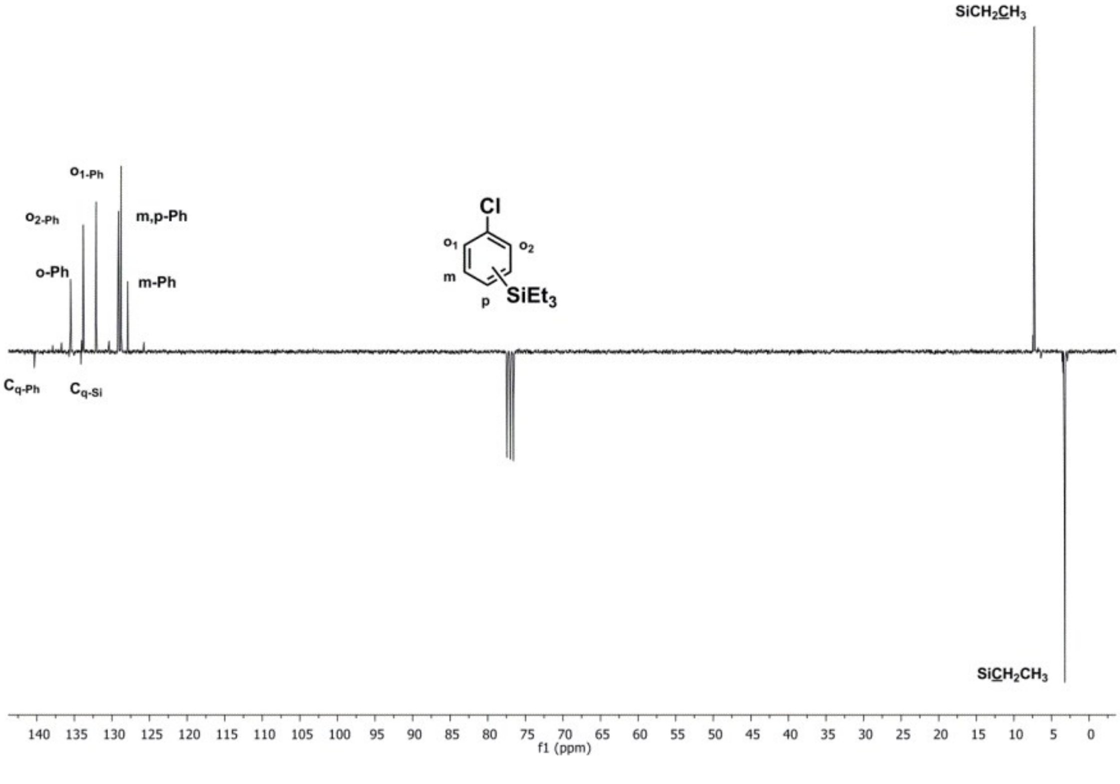

^{1}H-^{29}Si HMBC

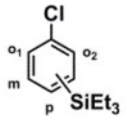
Cl
o1
o2
m
p
SiEt3

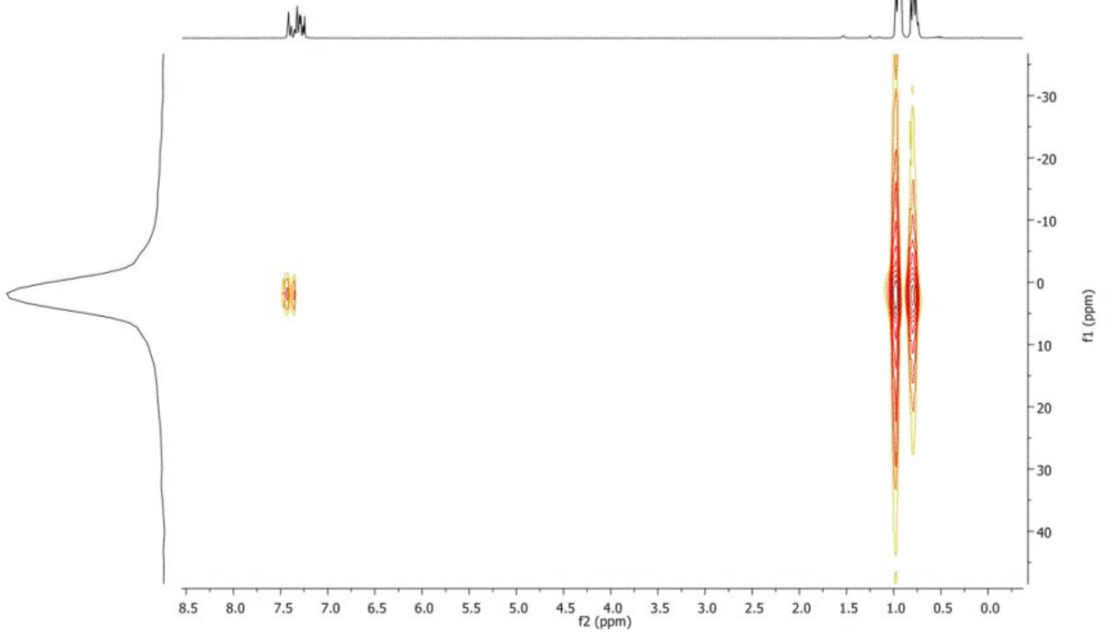
8.5 8.0 7.5 7.0 6.5 6.0 5.5 5.0 4.5 4.0 3.5 3.0 2.5 2.0 1.5 1.0 0.5 0.0
f2 (ppm)
-30 -20 -10 0 10 20 30 40
f1 (ppm)

Triethyl(ethylphenyl)silane

^{1}H NMR

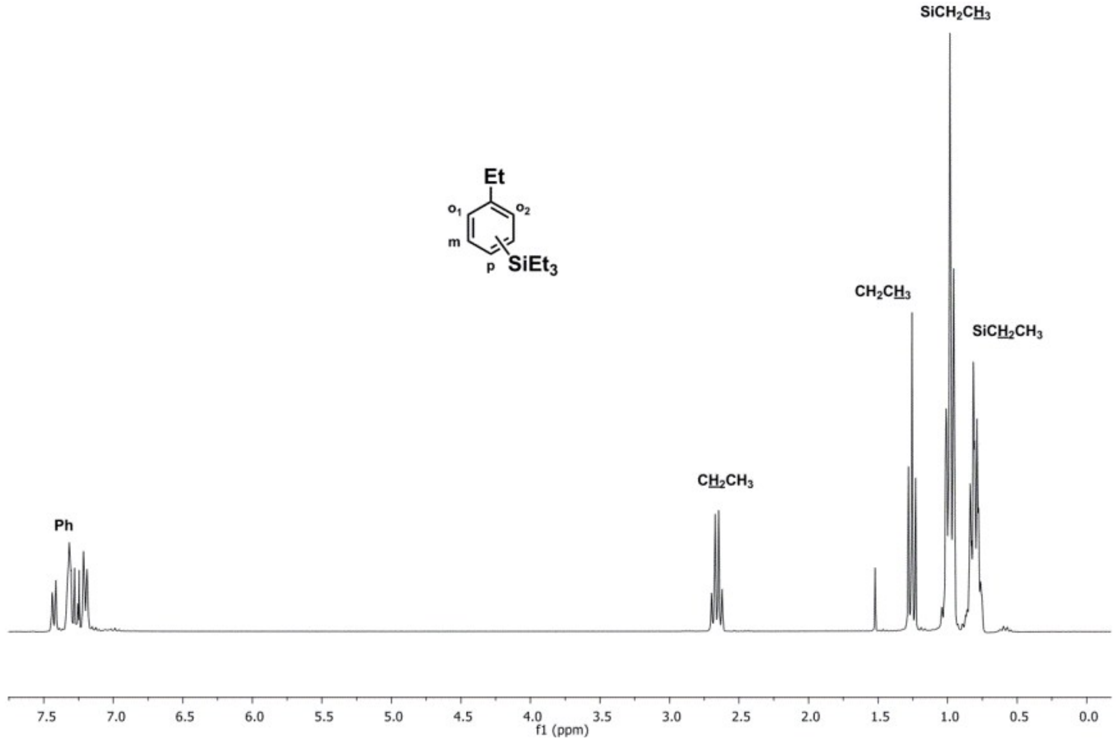

^{13}C NMR

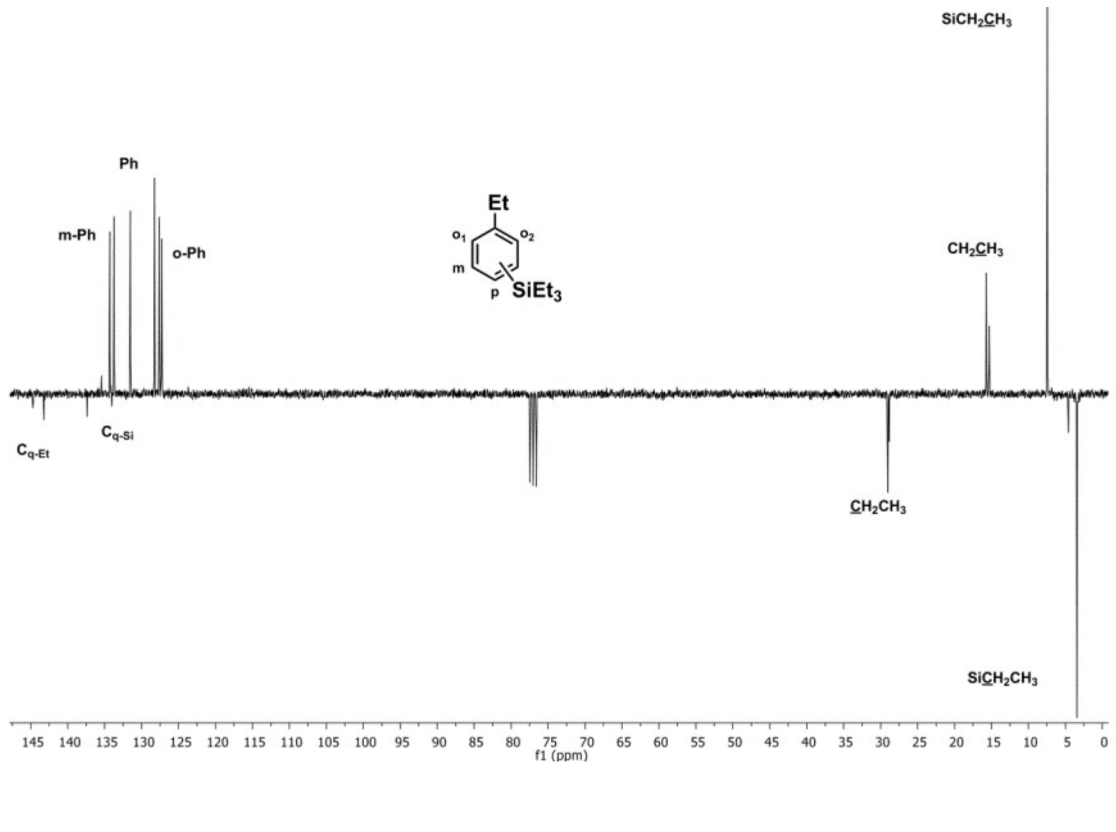

^{1}H-^{29}Si HMBC

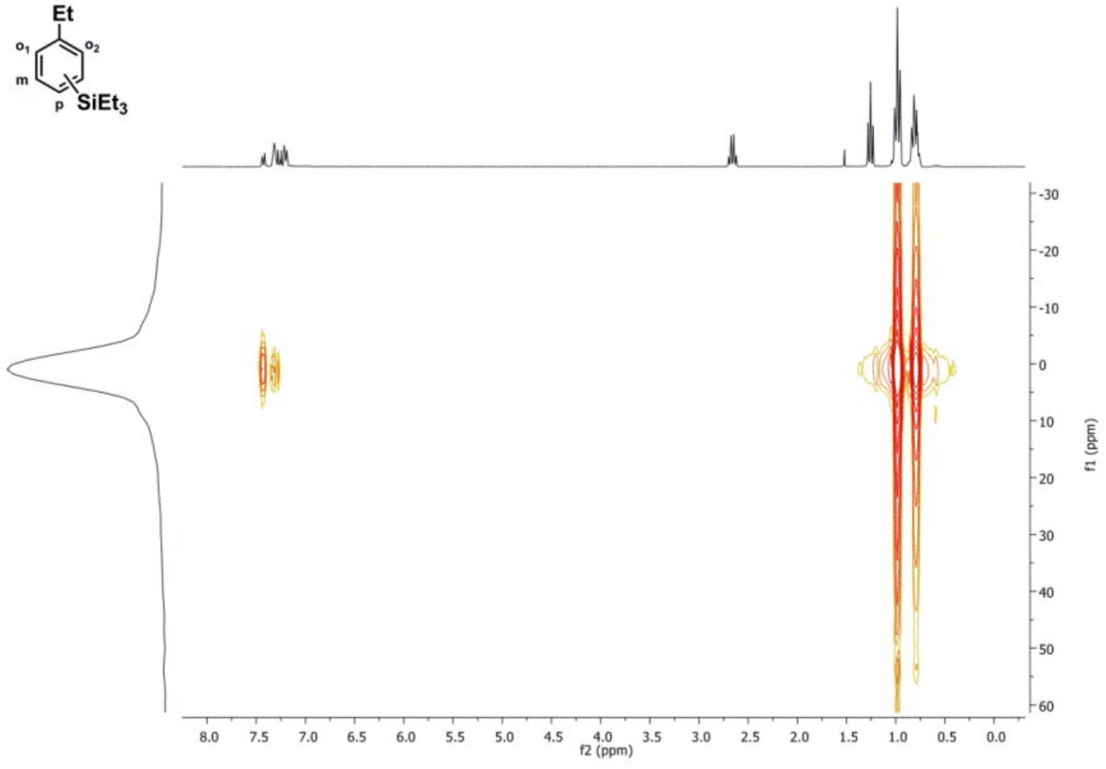

(sec-butylphenyl)triethylsilane

^{1}H NMR

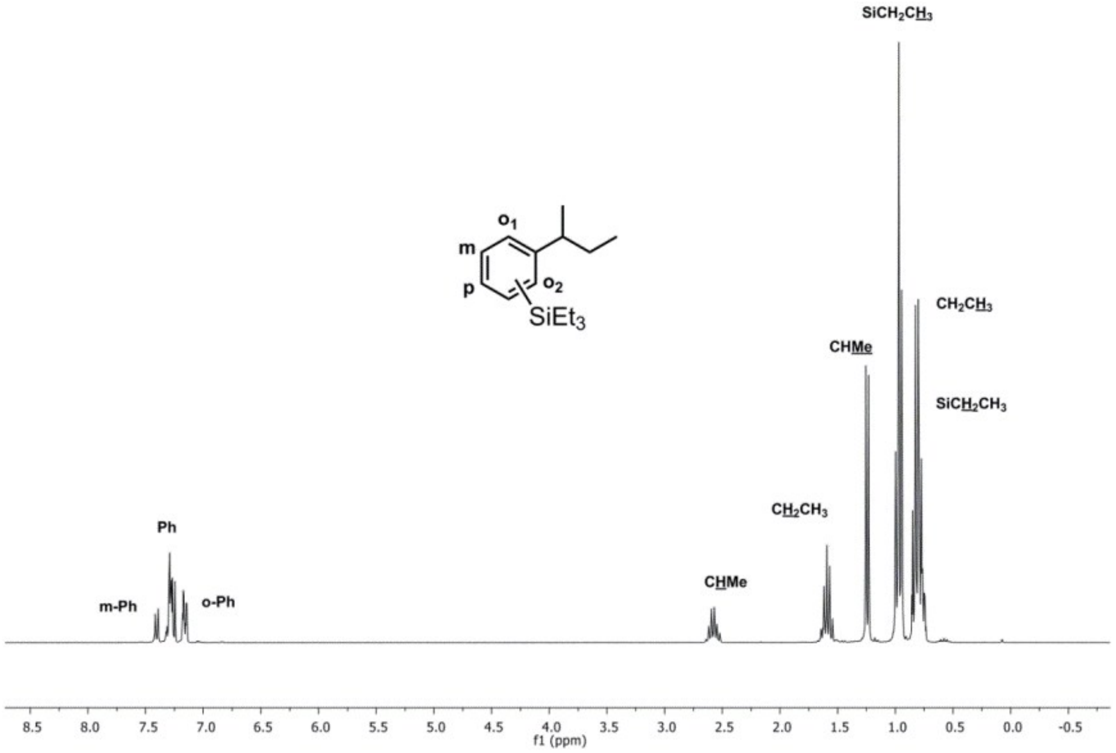

^{13}C NMR

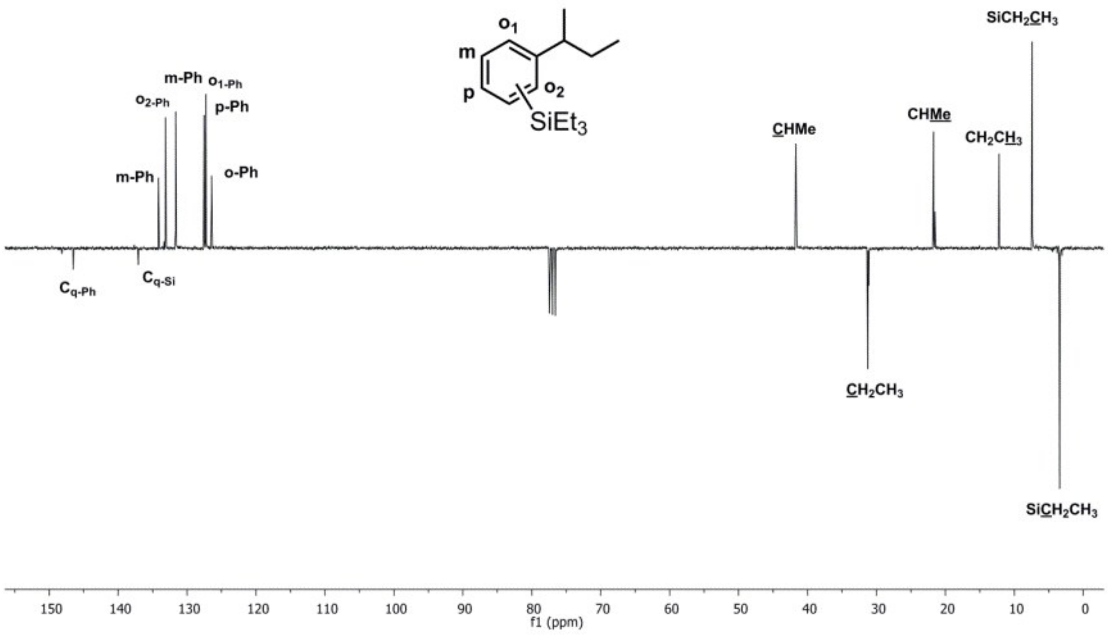

^{1}H-^{29}Si HMBC

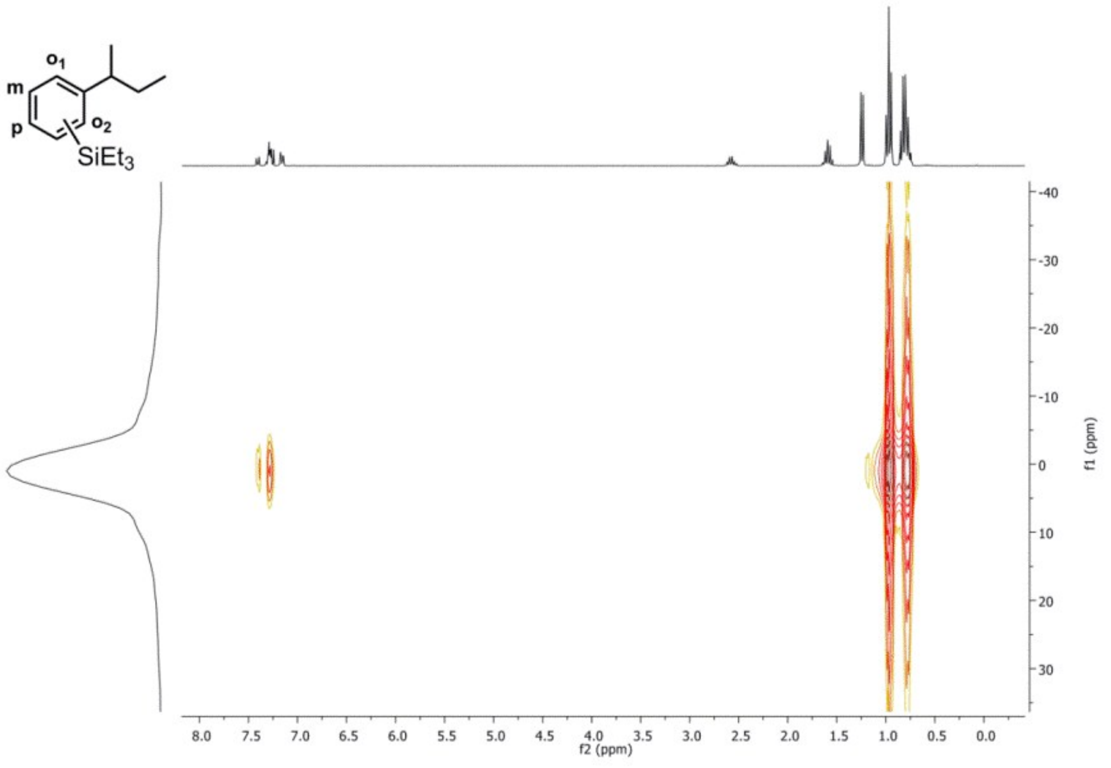
o1
m
p
o2
SiEt3
-40
-30
-20
-10
0
10
20
30
f1 (ppm)
8.0
7.5
7.0
6.5
6.0
5.5
5.0
4.5
4.0
3.5
3.0
2.5
2.0
1.5
1.0
0.5
0.0
f2 (ppm)

4-chlorophenyldimethylphenylsilane

^{1}H NMR

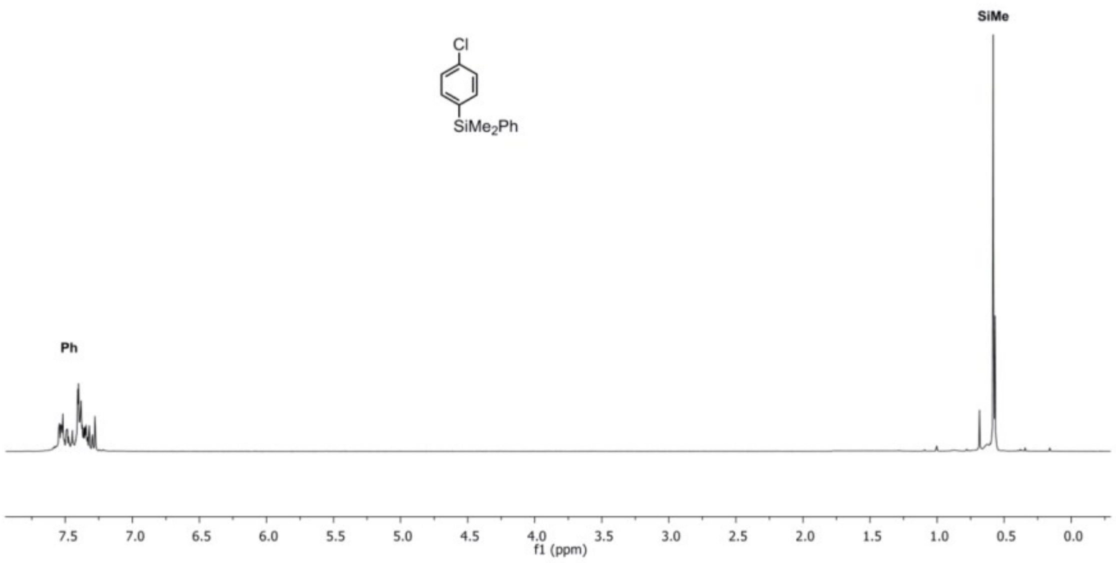

^{13}C NMR

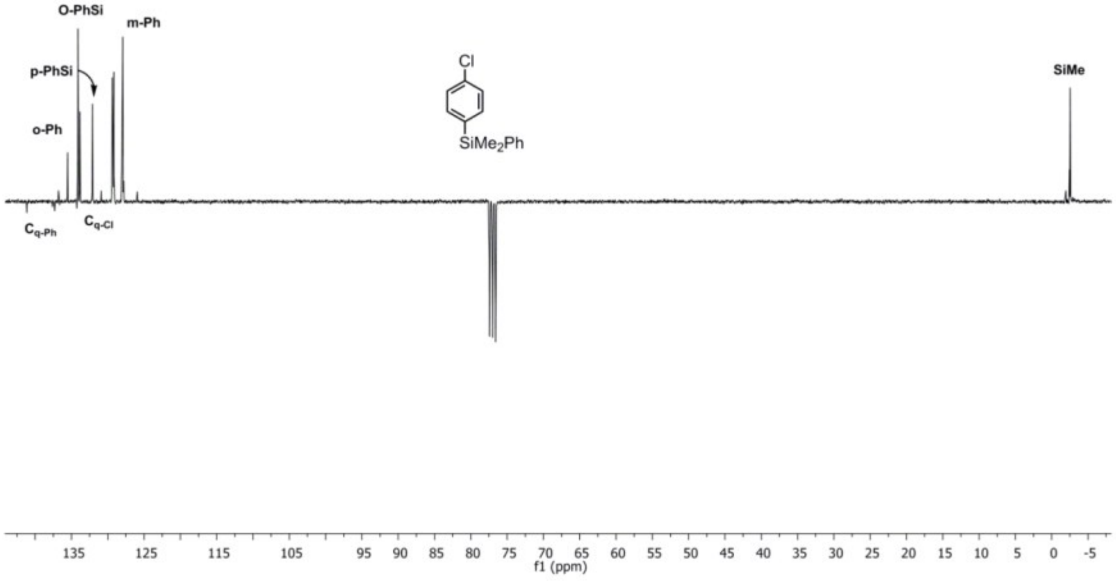

^{1}H-^{29}Si HMBC

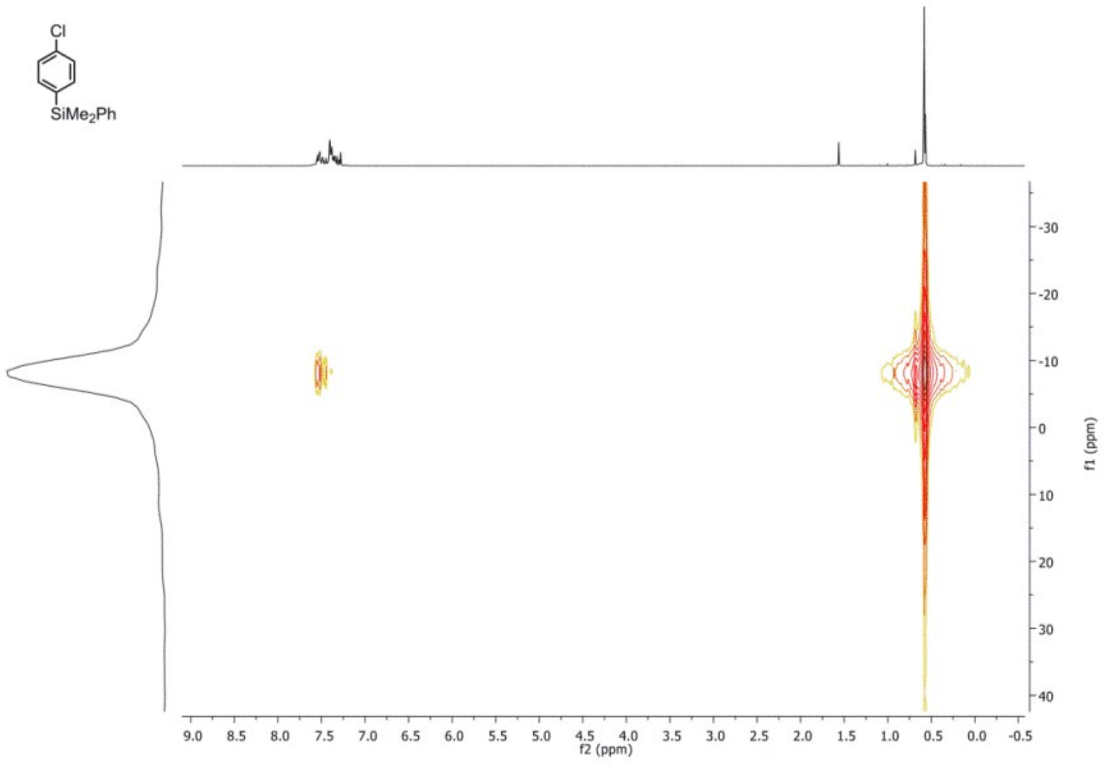

1,4-bis(dimethyl(phenyl)silyl)benzene

^{1}H NMR

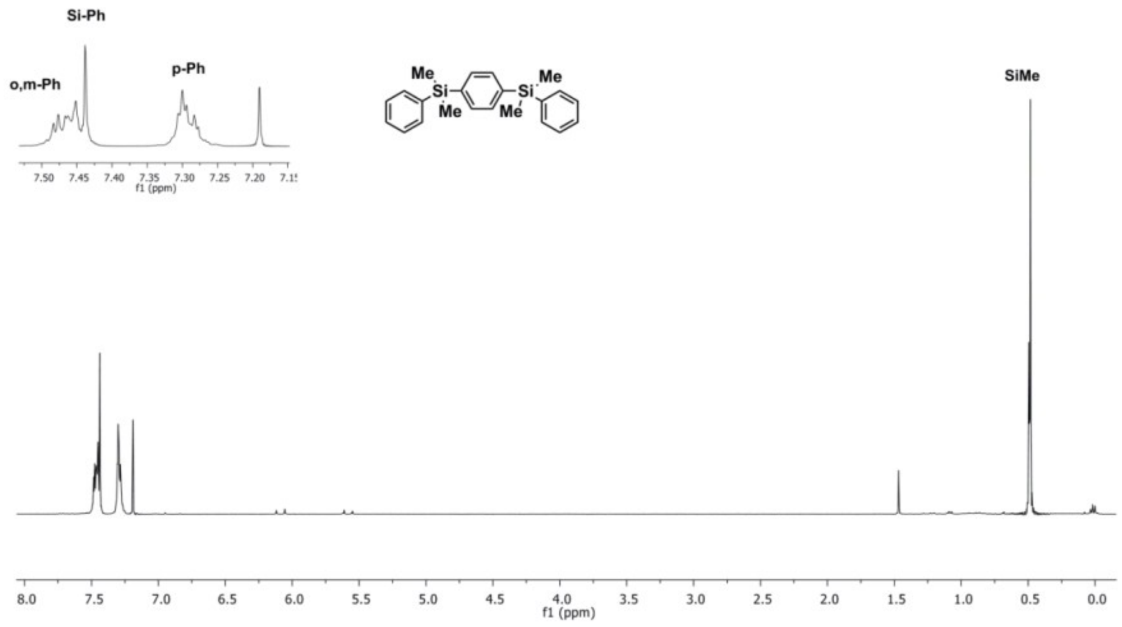

^{13}C NMR

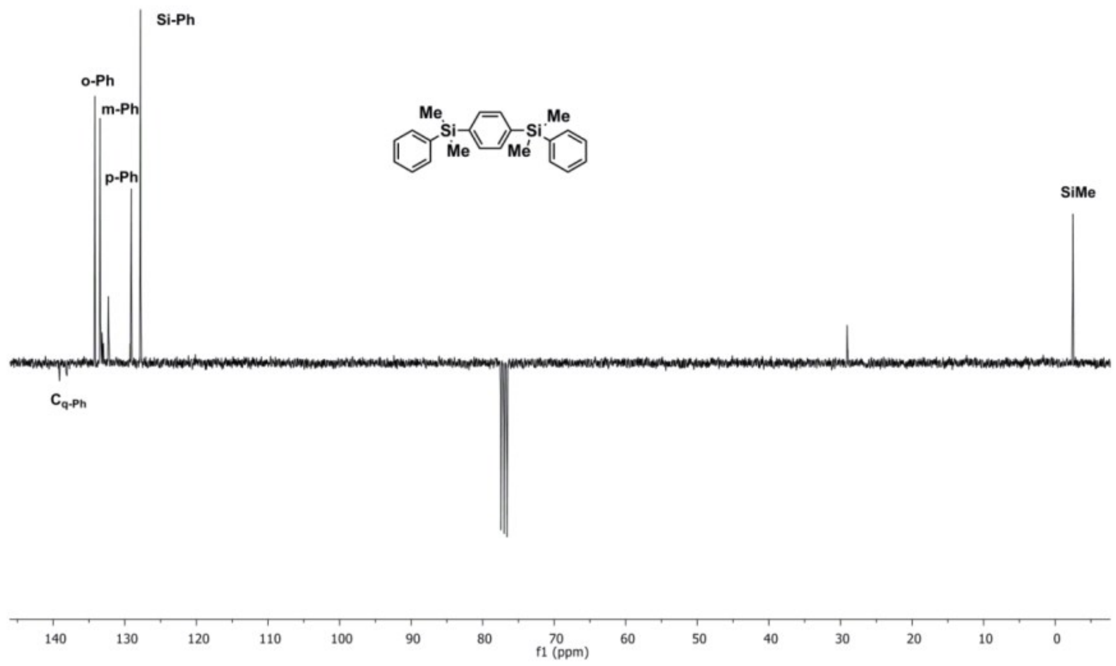

^{1}H-^{1}H COSY

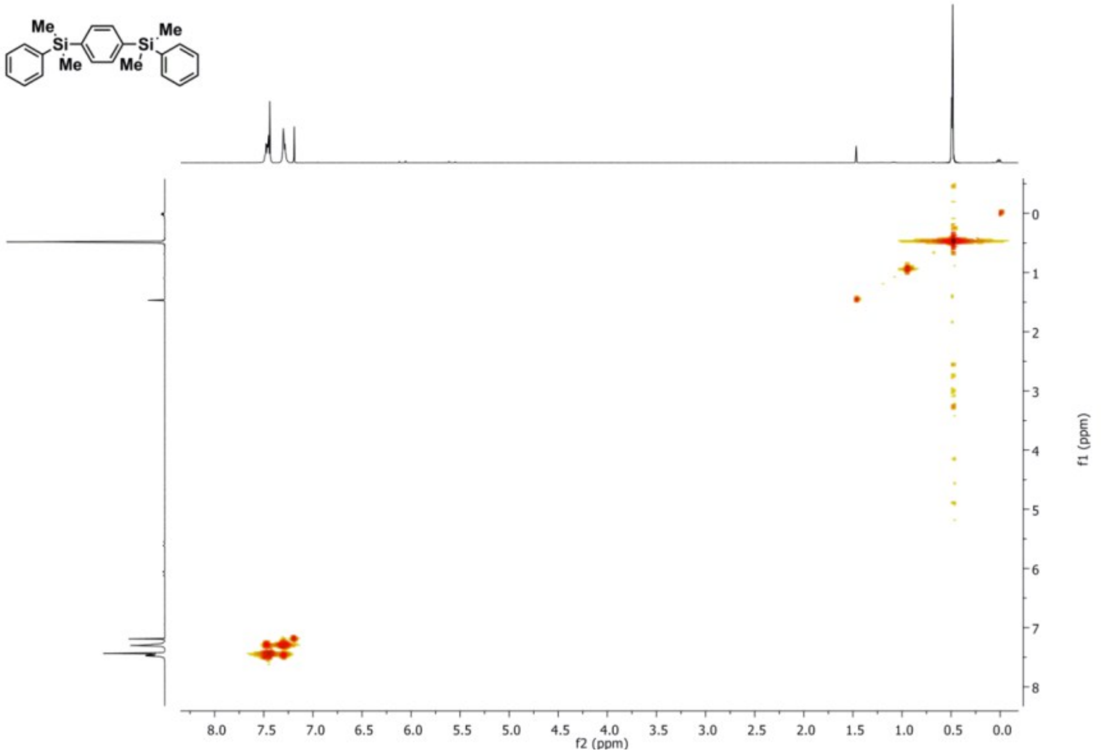

^{1}H-^{13}C HSQC

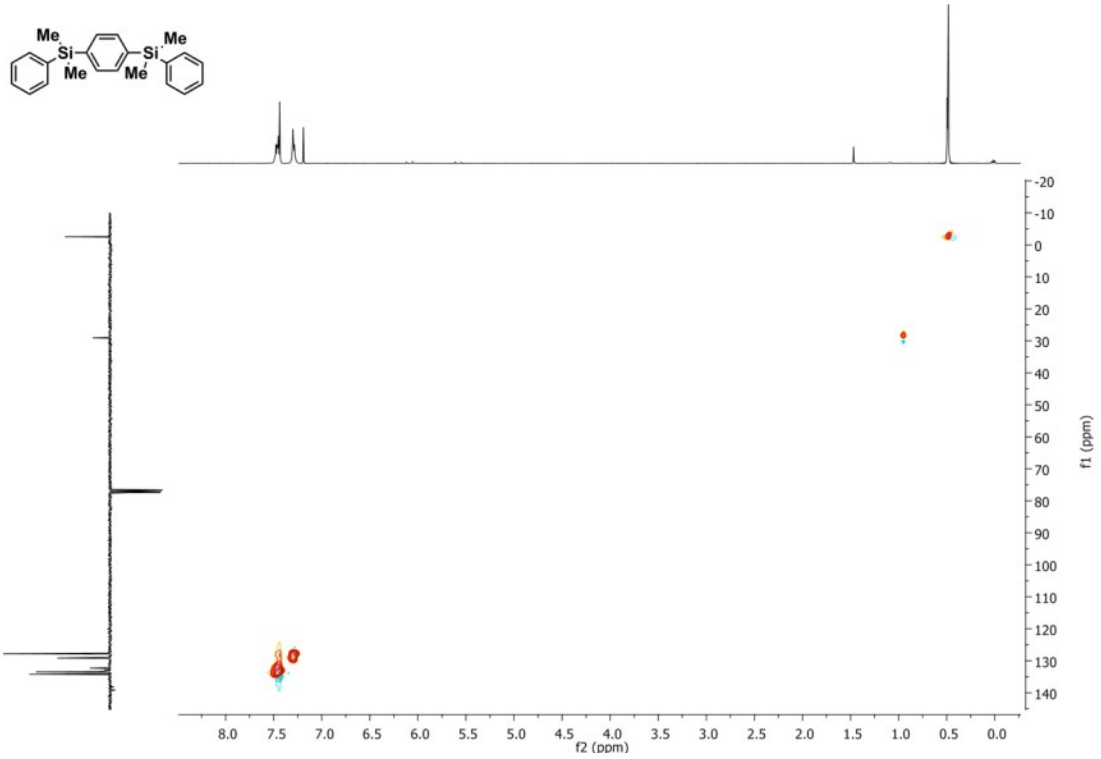

^{1}H-^{13}C HMBC

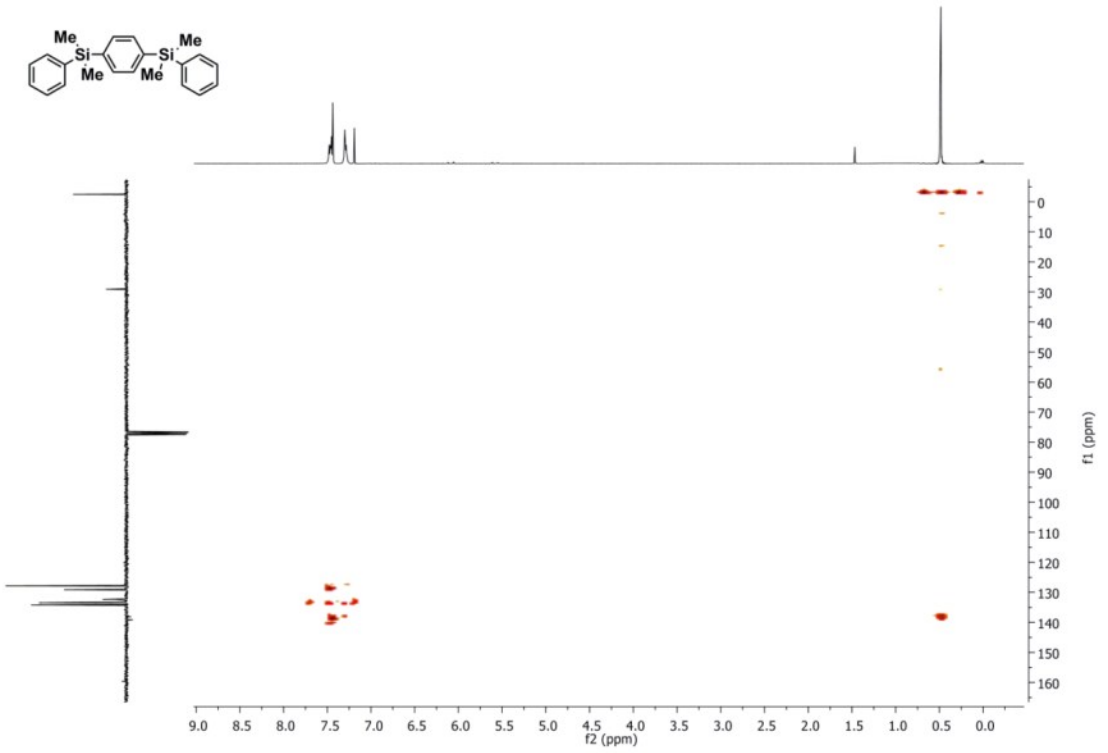

^{1}H-^{29}Si HMBC

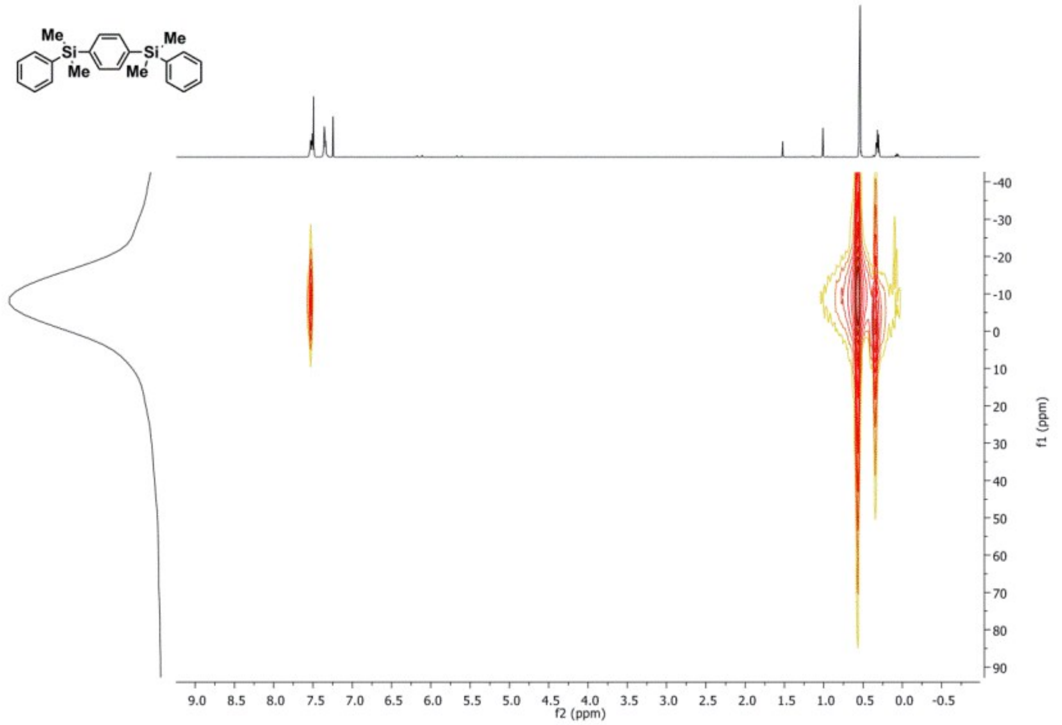

1,4-bis(dimethyl(2-(pyridin-2-yl)thiophen-3-yl)silyl)benzene

^{1}H NMR

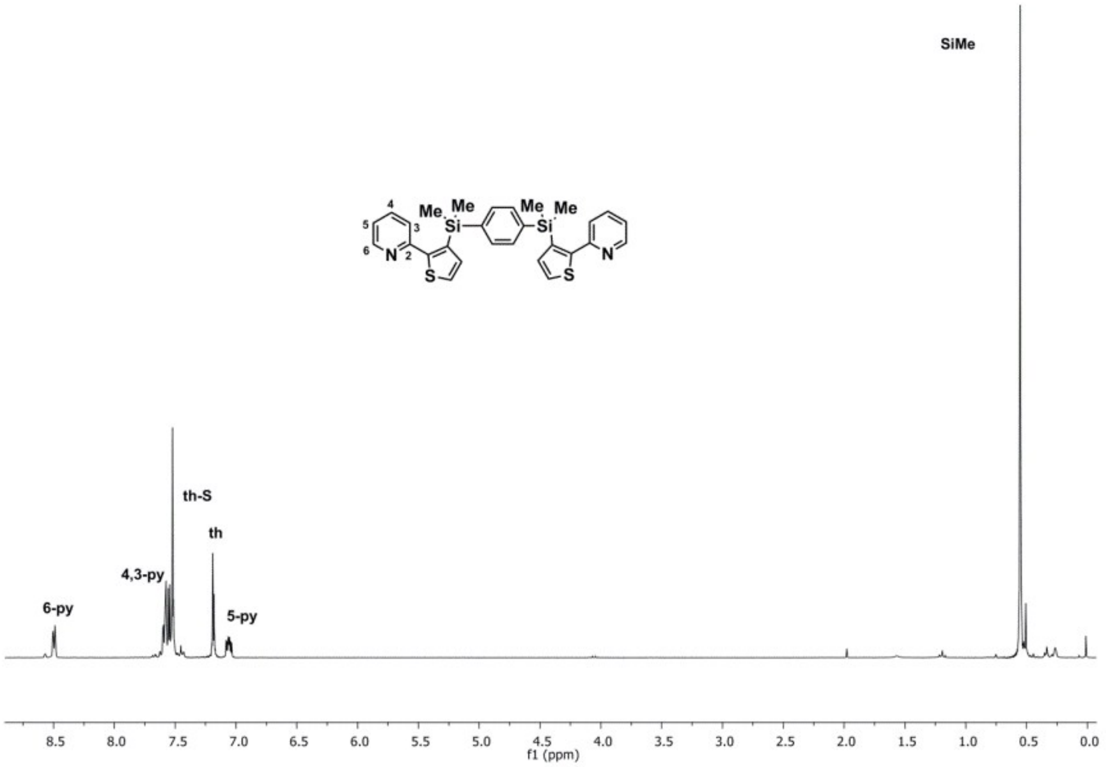

^{13}C NMR

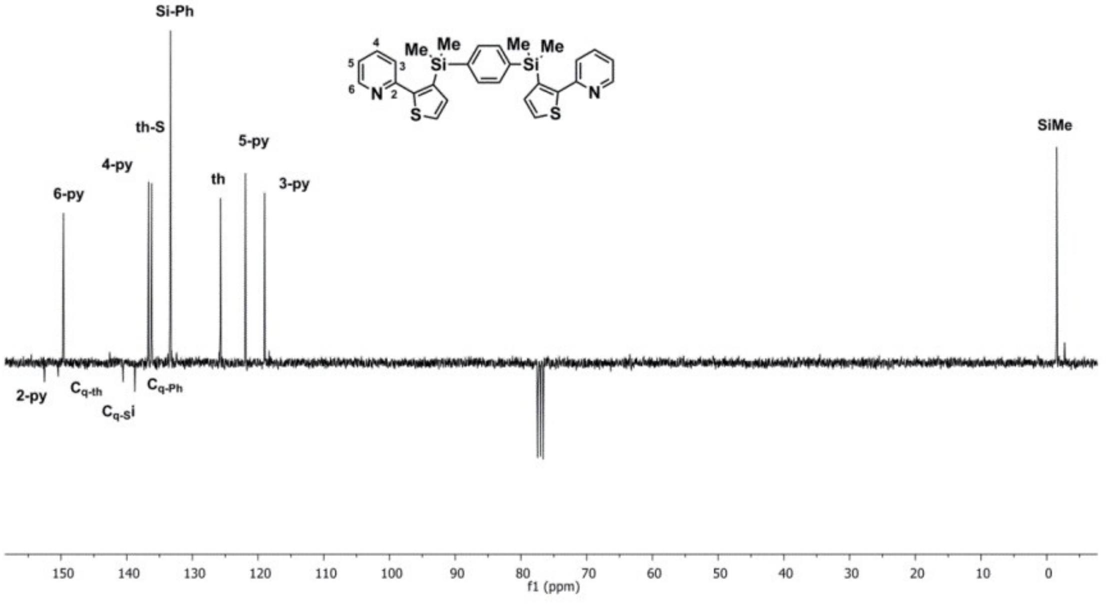

^{1}H-^{1}H COSY

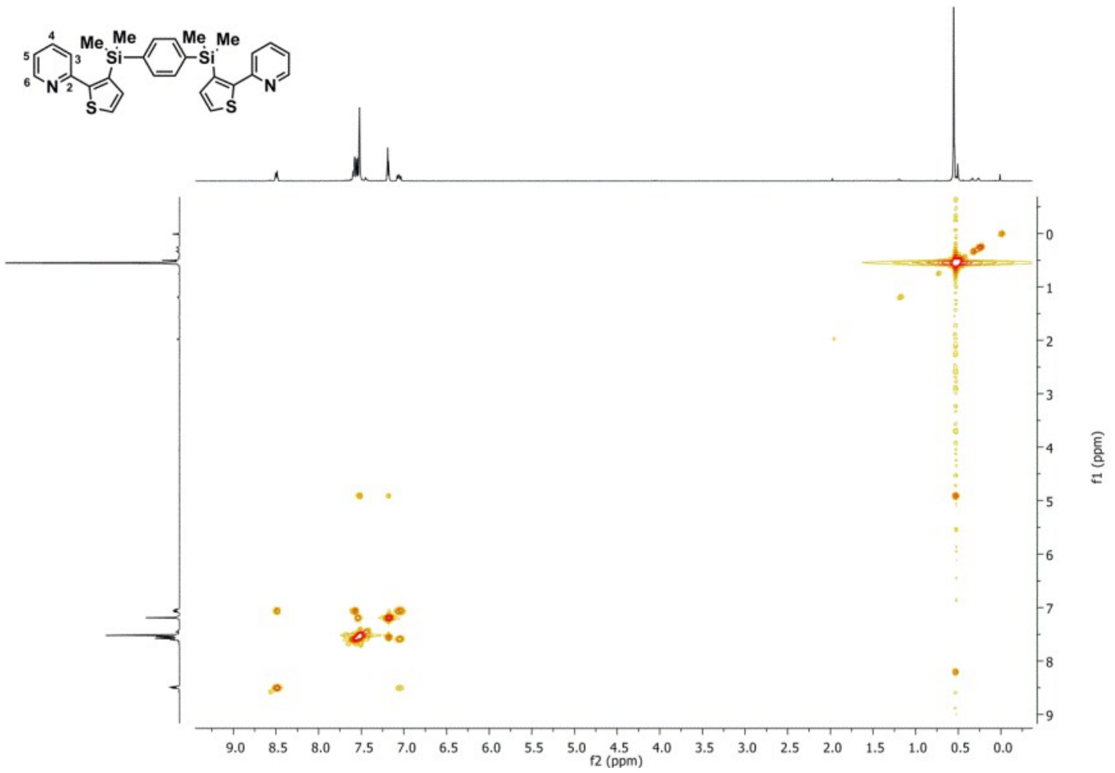

^{1}H-^{13}C HSQC

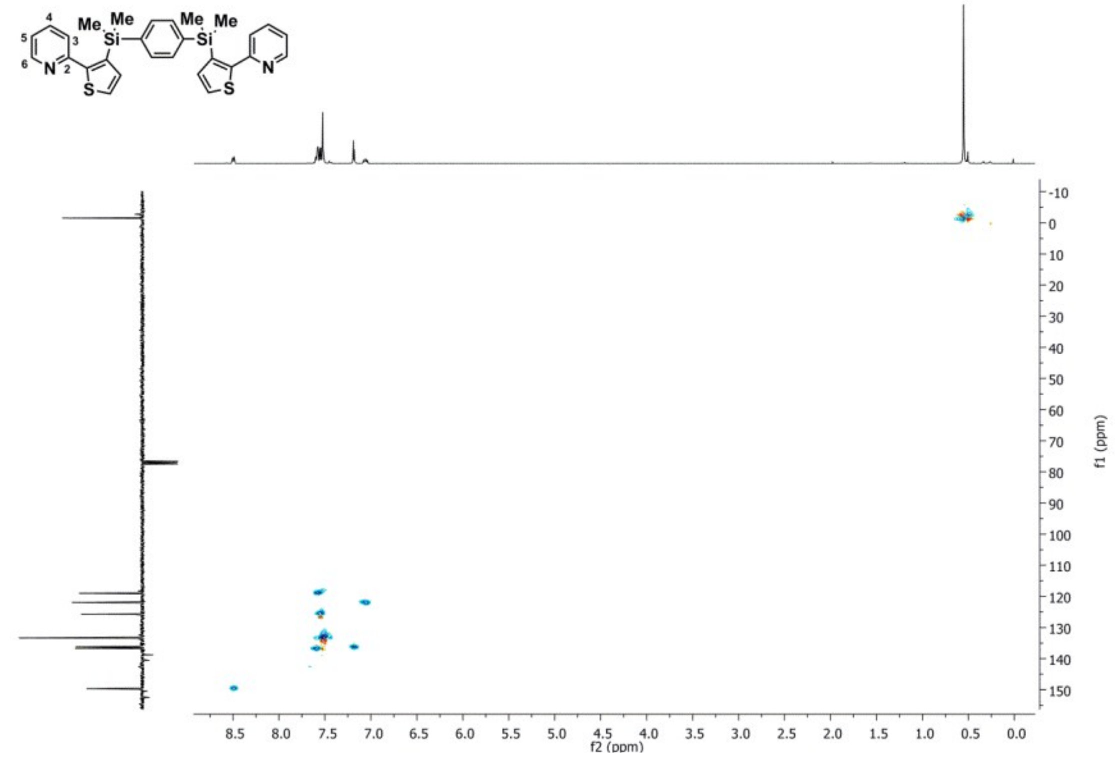

^{1}H-^{13}C HMBC

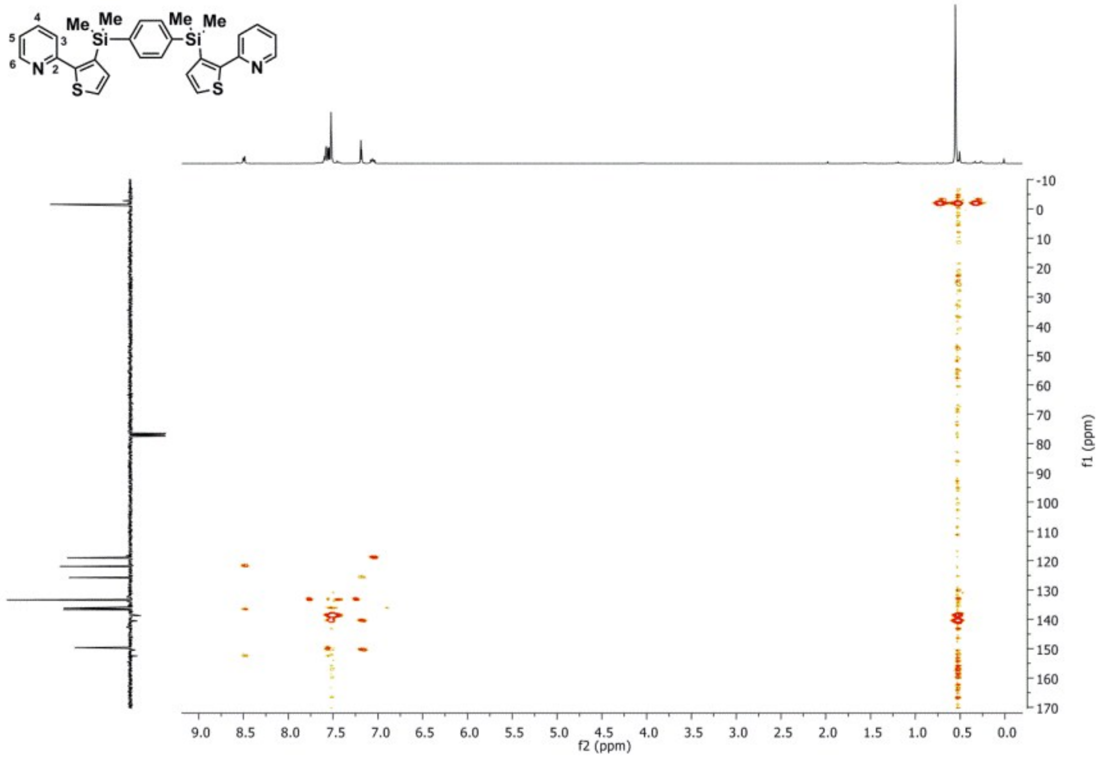

^{1}H-^{29}Si HMBC

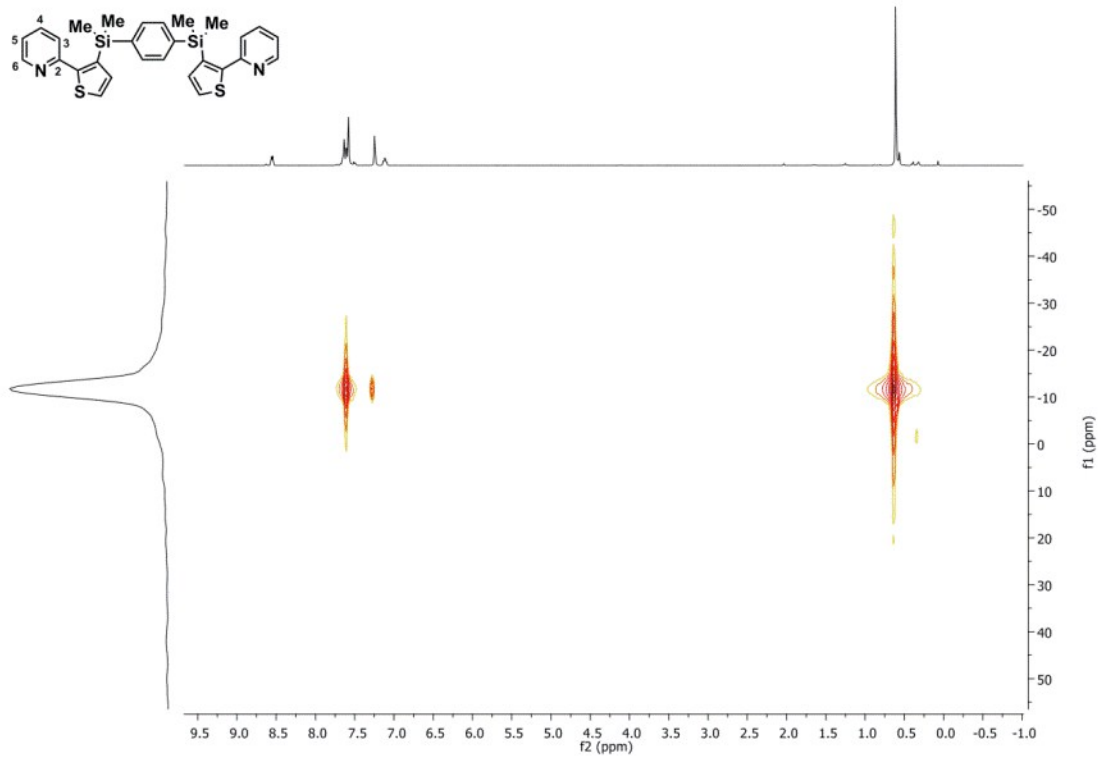

1,4-bis(dimethyl(5-methylthiophen-2-yl)silyl)benzene

^{1}H NMR

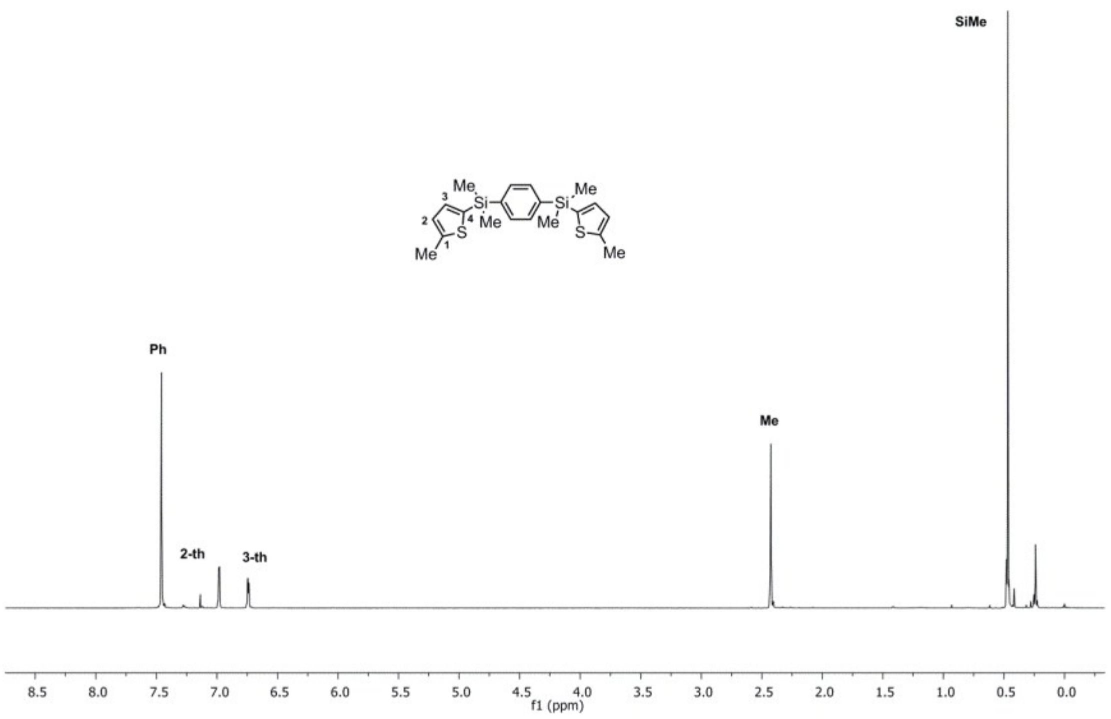

^{13}C NMR

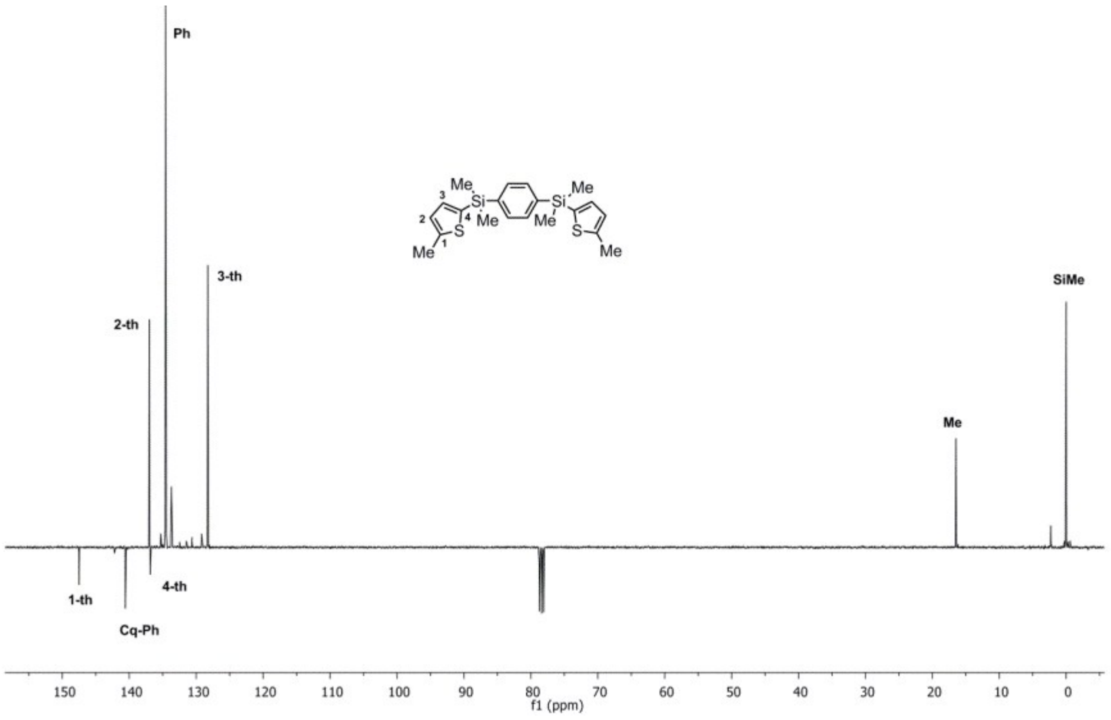

^1H-^1H COSY

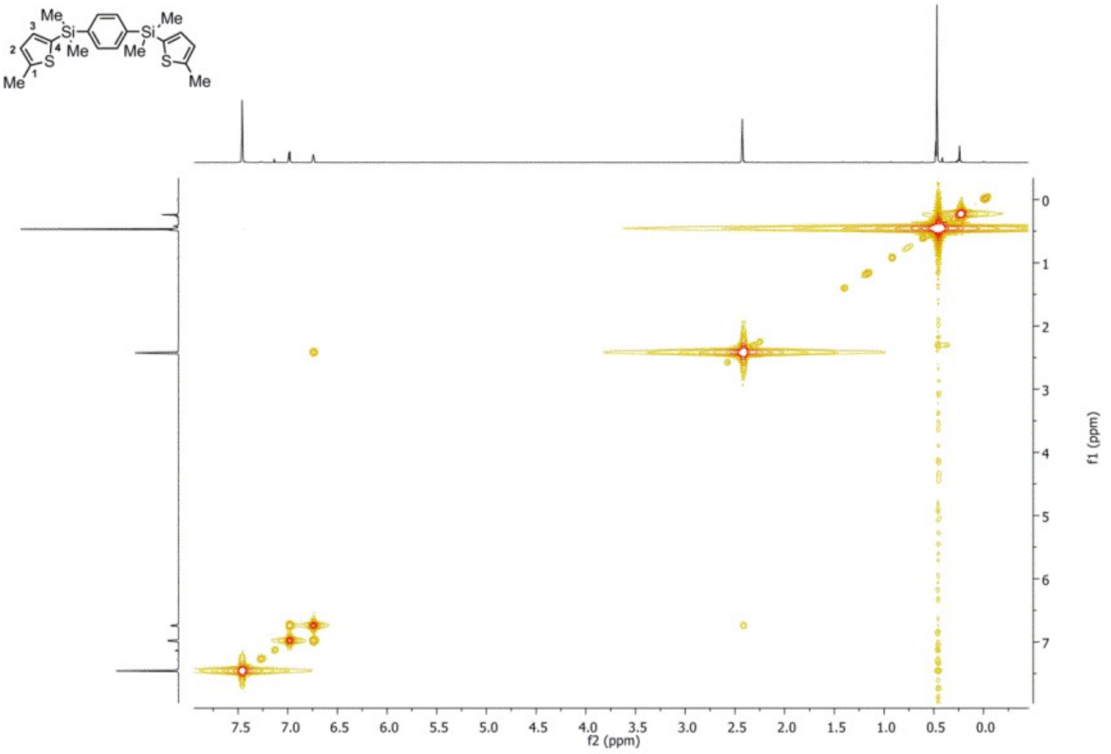

^1H-^{13}C HSQC

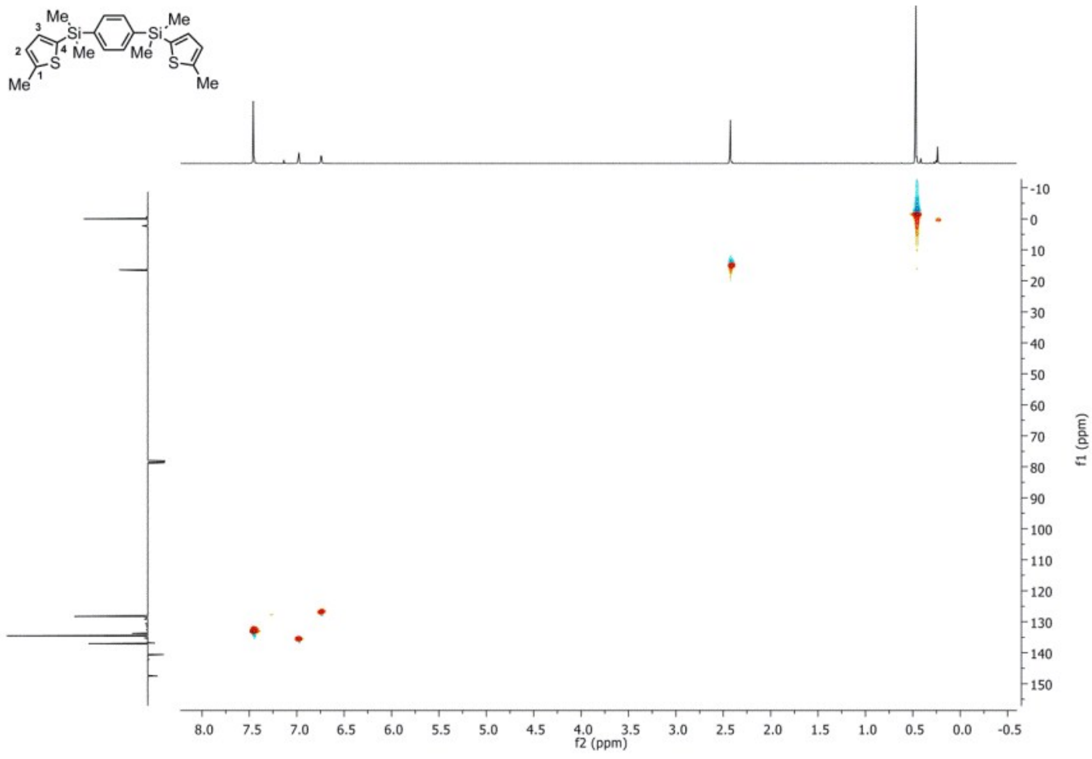

^{1}H-^{13}C HMBC

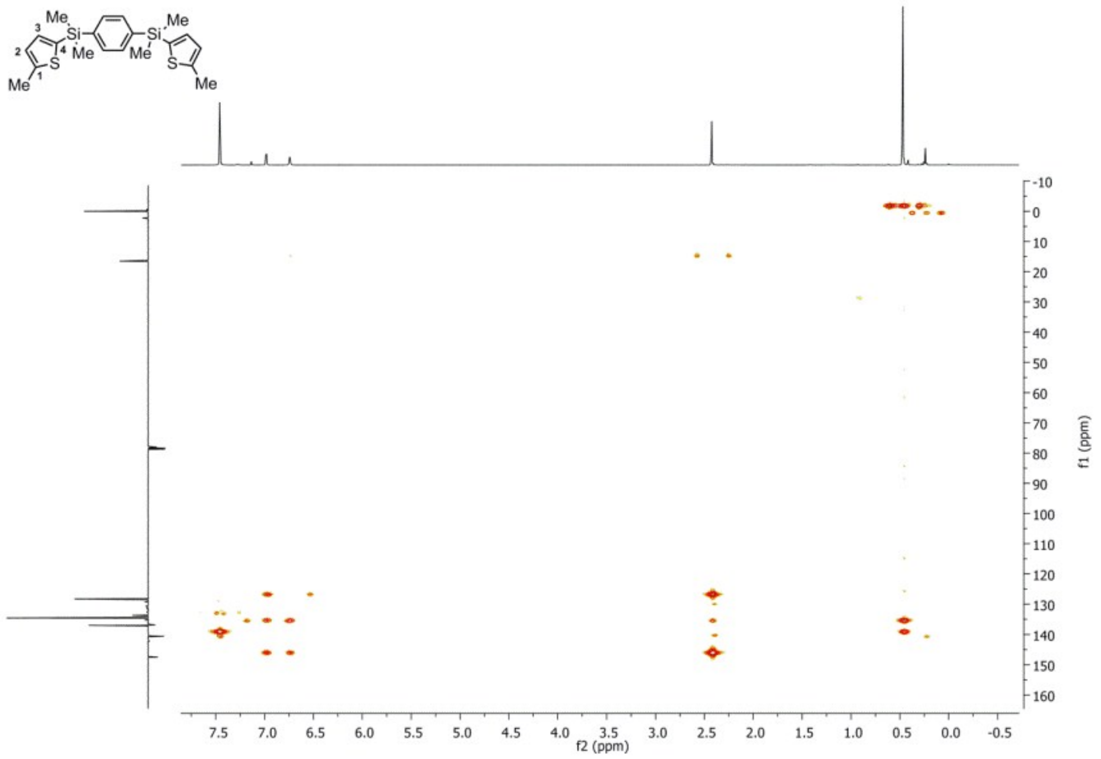

^{1}H-^{29}Si HMBC

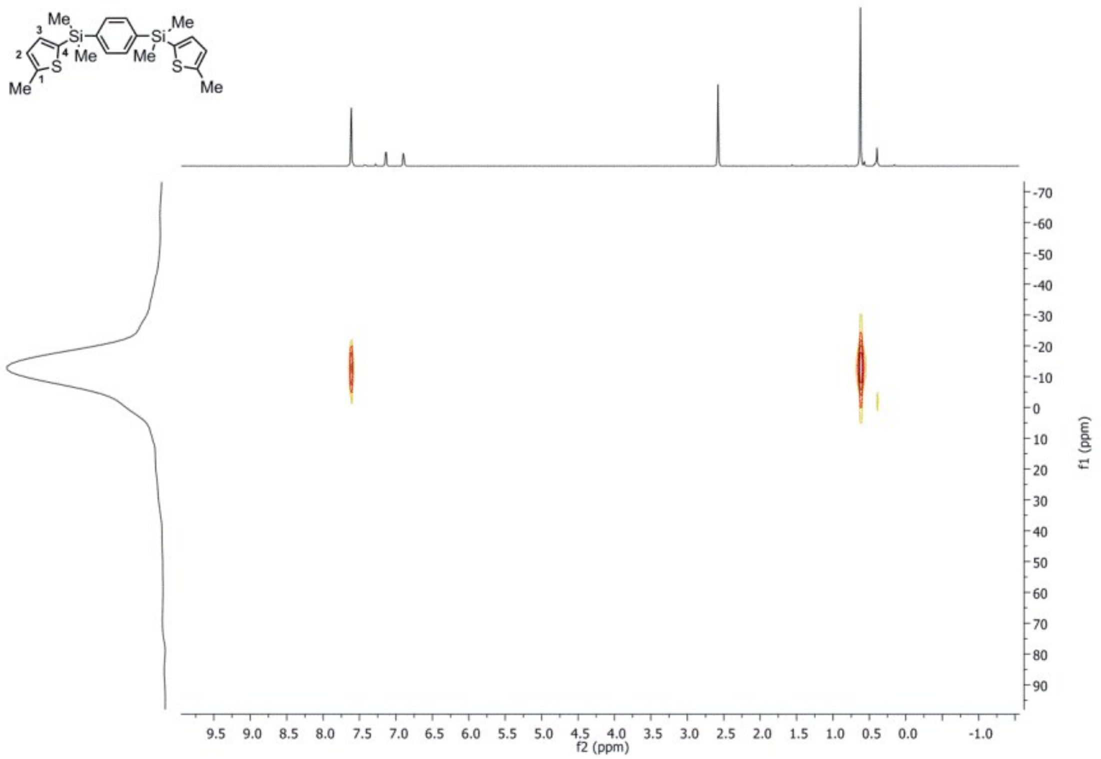

1,4-bis(benzo[b]thiophen-2-yldimethylsilyl)benzene

^{1}H NMR

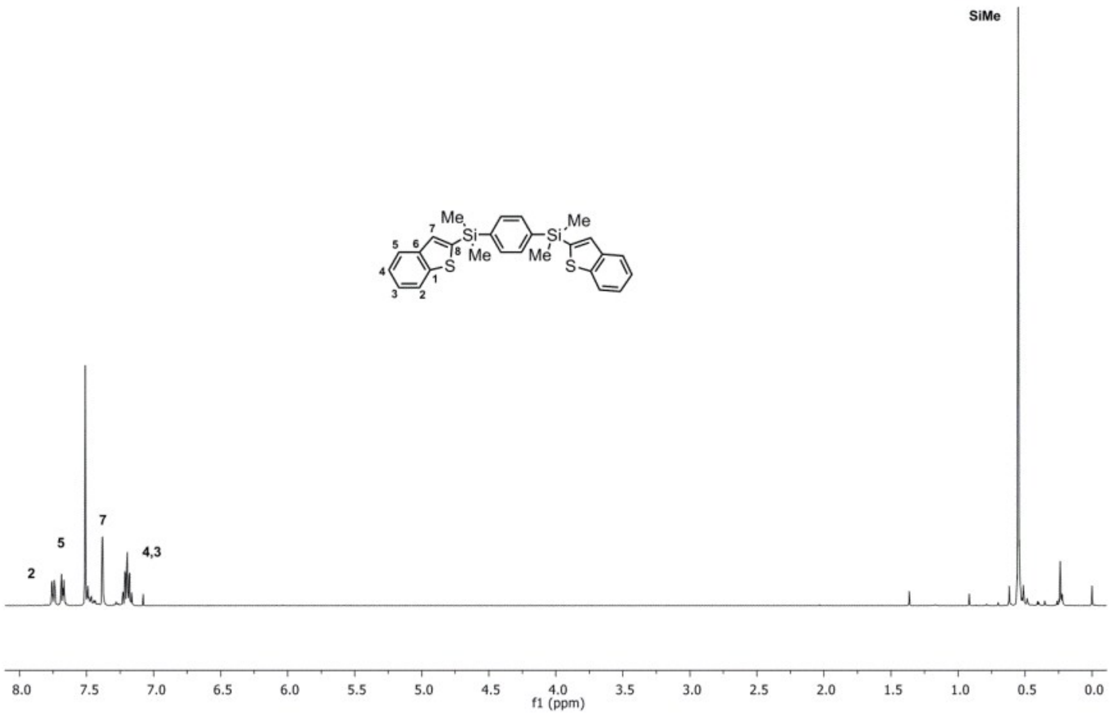

^{13}C NMR

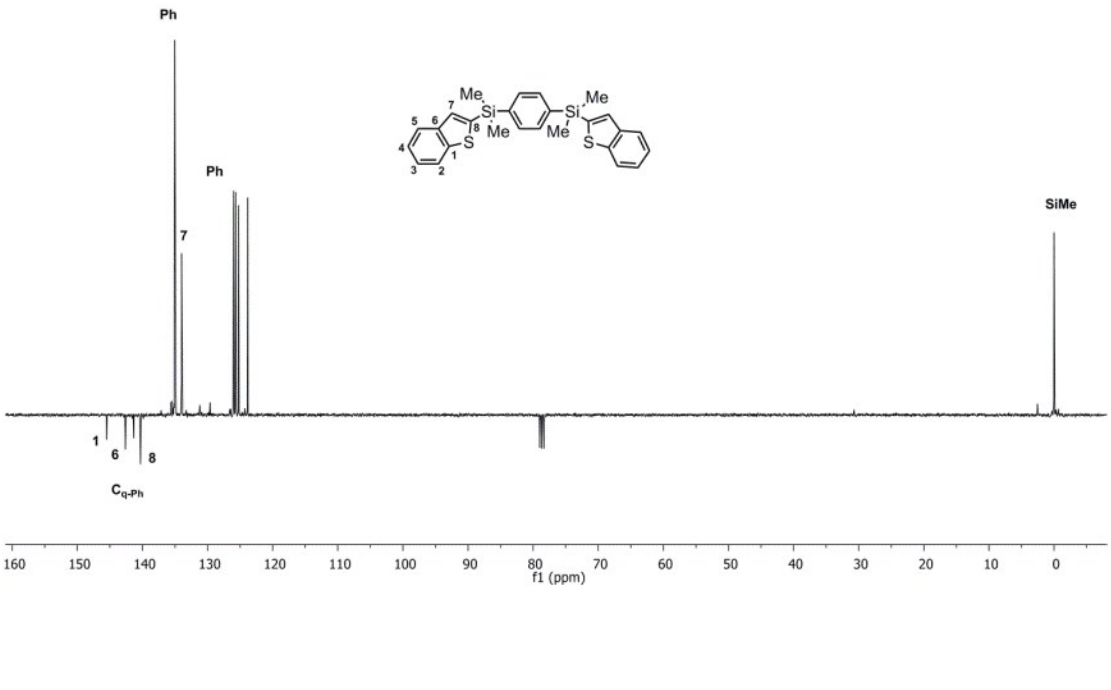

^{1}H-^{1}H COSY

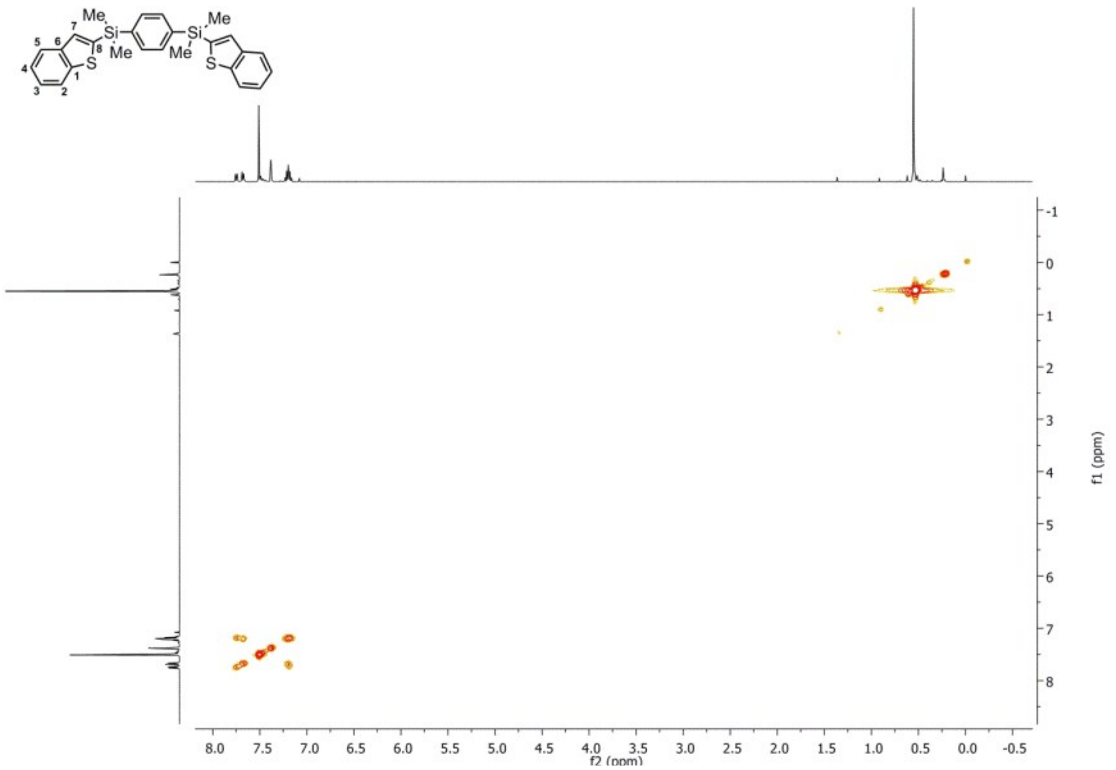

^{1}H-^{13}C HSQC

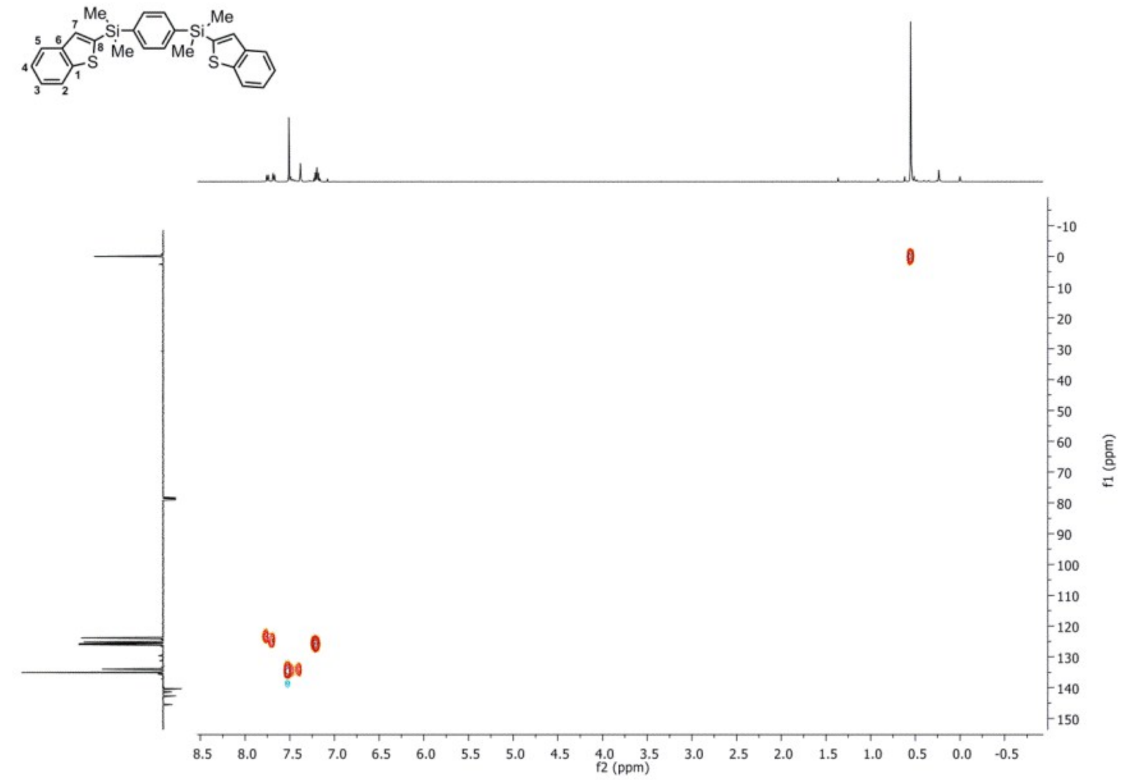

^{1}H-^{13}C HMBC

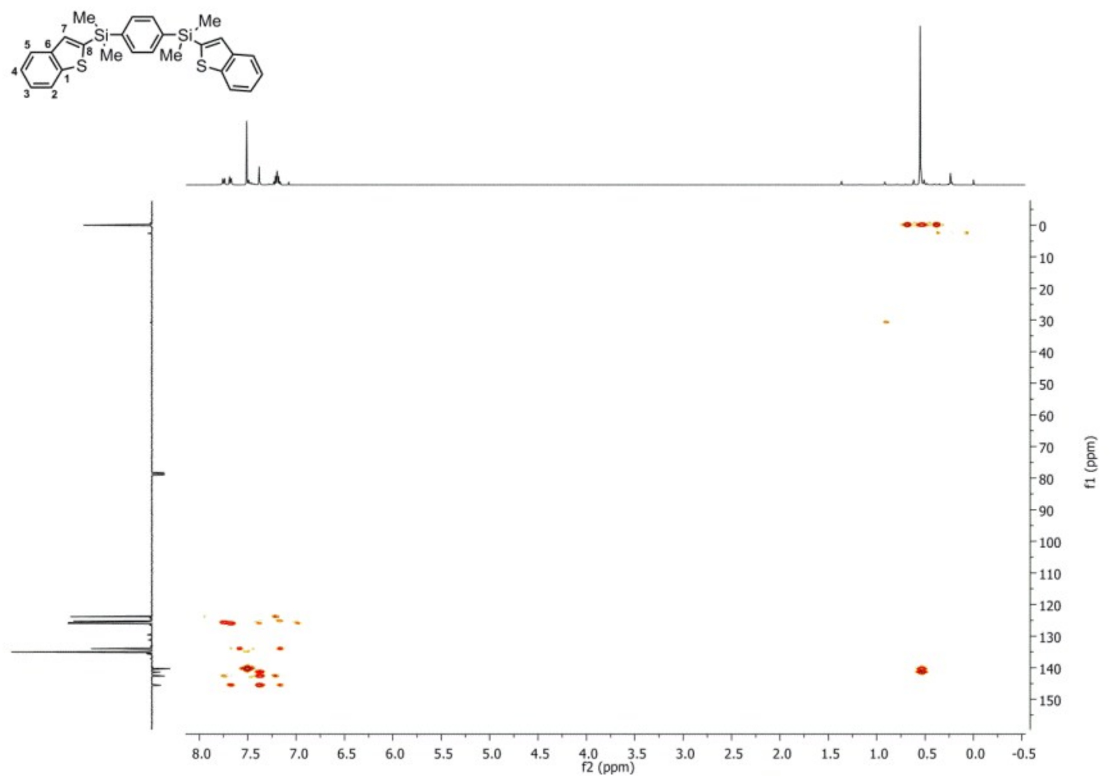

^{1}H-^{29}Si HMBC

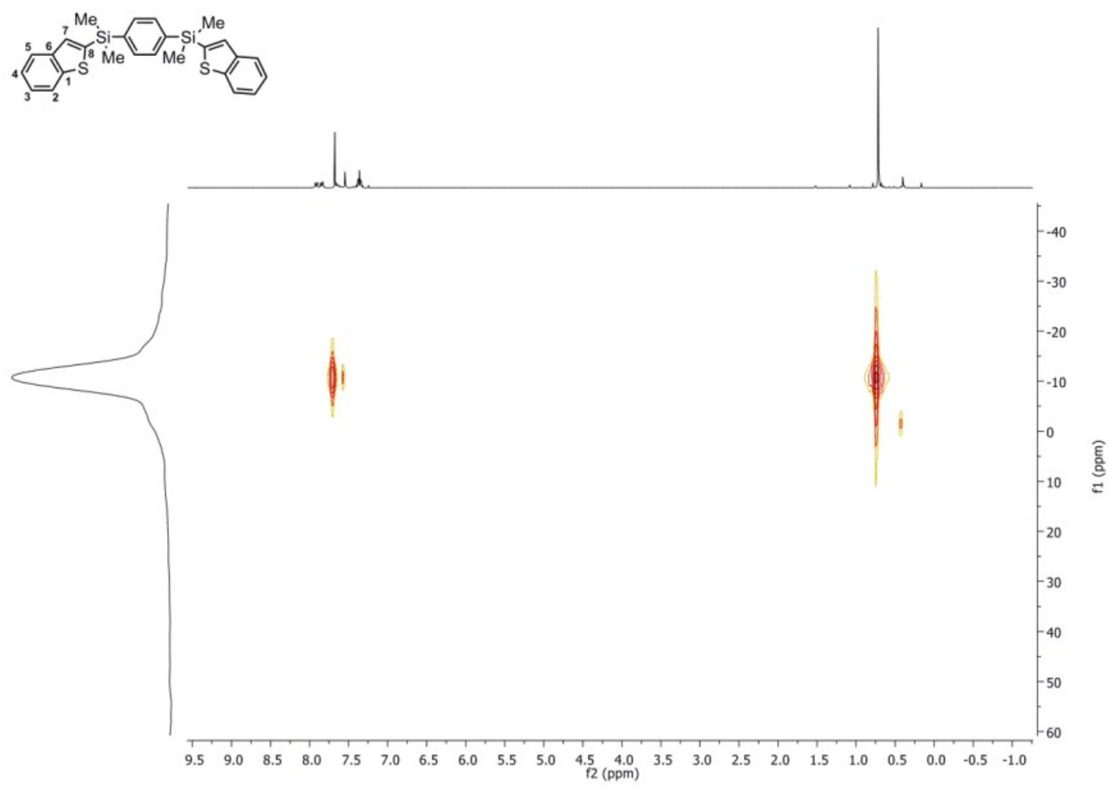

Solid state structure determination. Intensities were collected at 100(2) K on a Bruker APEX diffractometer with graphite–monochromated Mo–Kα radiation (λ = 0.71073 Å) using narrow ω rotations. Intensities were integrated and corrected for absorption effects using the SAINT+[1] and SADABS[2] programs, included in the APEX2 package. The structure was solved by the Patterson's method. Refinement was carried out by full–matrix least–square procedure (based on F_0^2) using anisotropic temperature factors for all non-hydrogen atoms. Calculations were performed with SHELX–97[3] program implemented in the WinGX package.[4]

Crystal data and structure refinement for **[Ir(H)$_2$(IPr)(py)$_3$][BF$_4$]** (**1**). $C_{42}H_{53}BF_4IrN_5$, M_r = 906.90, orthorhombic, *Pbca*, a = 20.4440(10) Å, b = 18.9570(10) Å, c = 20.8205(11) Å, V = 8069.1(7) Å^3, Z = 8, D_{calc} = 1.493 g/cm^3, μ = 3.364 mm^{-1}, F(000) = 3664, 0.250 x 0.220 x 0.140 mm^3, θ range: 2.149 to 28.667°, index ranges $-27 \le h \le 14$, $-24 \le k \le 24$, $-26 \le l \le 18$, reflections collected: 45462, independent reflections: 9533 (R_{int} = 0.0309), Data/restraints/parameters: 9533/60/495, GOF(F^2)=1.030, R_1 = 0.0310 [I>2$\sigma(I)$], wR_2 = 0.0789 (all data). CCDC 1507888.

Crystal data and structure refinement for **[Ir(H)(IPr)(Phpy-1H)(py)$_2$][BF$_4$]**·CH_2Cl_2 (**2**·CH_2Cl_2). $C_{49}H_{57}BCl_2F_4IrN_5$, M_r = 1065.90, triclinic, $P\bar{1}$, a = 11.1839(7) Å, b = 11.2131(7) Å, c = 19.4442(12) Å, α = 89.5340(10)° β= 80.5790(10)° γ = 85.2210(10)°, V = 2397.1(3) Å^3, Z = 2, D_{calc} = 1.477 g/cm^3 , μ = 2.951 mm^{-1}, F(000) = 1076, 0.160 x 0.140 x 0.140 mm^3, θ range: 2.104 to 28.573°, index ranges: $-14 \le h \le 14$, $-14 \le k \le 15$, $-25 \le l \le 25$, reflections collected: 28054, independent reflections: 11120 (R_{int} = 0.0317), data/restraints/parameters: 11120/16/596, GOF(F^2) = 1.066, R_1 = 0.0331 [I>2$\sigma(I)$], wR_2 = 0.0820 (all data). CCDC 1507890.

Crystal data and structure refinement for **[Ir(H)(IPr)(py)$_2$(Thpy-1H)][BF$_4$]**·1.5·CH_2Cl_2 (**3**·1.5·CH_2Cl_2). $C_{95}H_{112}B_2Cl_6F_8Ir_2N_{10}S_2$, M_r = 2228.78, monoclinic, $P2_1/c$, a = 21.1207(14) Å, b = 11.2623(8) Å, c = 22.7007(15) Å, β = 114.3290(10)°, V = 4920.2(6) Å^3, Z = 2, D_{calc} = 1.504 g/cm^3, μ = 2.972 mm^{-1}, F(000) = 2244, 0.250 x 0.160 x 0.100 mm^3, θ range: 2.059 to 28.570°, index ranges $-28 \le h \le 27$, $-15 \le k \le 14$, $-29 \le l \le 30$, reflections collected: 55195, independent reflections: 11624 (R_{int} = 0.0434), data/restraints/parameters: 11624/50/591, GOF(F^2) = 1.039, R_1 = 0.0406 [I>2$\sigma(I)$], wR_2 = 0.0971 (all data). CCDC 1507889.

Computational Methods.

All DFT theoretical calculations were carried out using the Gaussian program package.[5] The B3LYP method,[6] including the D3 dispersion correction scheme developed by Grimme[7] with Becke Johnson damping, has been used for both energies and gradient calculations. All atoms were treated with the def2-SVP basis set[8] together with the corresponding core potential for Ir for geometry optimizations. Energies were further refined by single point calculations using the def2-TZVP basis set and solvent corrections using the PCM[9] approach for tetrahydrofuran (ε= 7.4257) as implemented in G09. The "ultrafine" grid was employed in all calculations. All reported energies are Gibbs free energies referred to a 1 M standard state at 110º C removing the contribution to the translational entropy, as indicated by Morokuma et al[10] and including basis set and solvent corrections. The nature of the stationary points was confirmed by analytical frequency analysis, and transition states were characterized by a single imaginary frequency corresponding to the expected motion of the atoms.

No transition structures could be found for oxidative addition of the C–H bond of phenyl-pyridine by **E** to yield the Ir(III) intermediate **F** and for the reductive elimination of H_2 from **A** to form D' plus hydrogen. In order to show that they are barrierless processes, relaxed partial optimisation has been performed along the C-H and H-H key bond distances. In Figure **S5**, the conventional three-centre transition state transition states for orto, meta and para CH bond activation without coordination of the nitrogen atom of the 2-phenylpyridine are shown. The orto-CH oxidative addition requires larger activation energy (15.9 kcal/mol relative **7**). Moreover, the activation of meta-CH and para-CH were calculated at 18.9 kcal/mol and 16.9 kcal/mol (relative to 7) and, therefore, some amount of para or meta product should be observed. Therefore, we can conclude that coordination of the phenyl pyridine nitrogen to the metal and activation of orto-CH bond is consistent with the experimental results. Moreover, this also agrees with the competitive experiment described in page 3 of the manuscript, where selective silylation of 2-phenylpyridine was observed in the presence of equimolar amounts of ethylbenzene.

Table S1. Energetic values for DFT calculated structures. Geometrical optimizations using the def2-SVP, E(DZ), basis set and single point energies using solvent corrections at the PCM level, E(PCM), and def2-TZVP, E(TZ). Correction to the Gibbs free energy without translational entropy at 110 ºC in a 1 M standard state calculated at the def2-SVP level, G'corr. All absolute energies in a.u. Gibbs free energies relative to **A** and isolated molecules considering solvent and triple-Z corrections in kcal mol^{-1}, S (cal/mol·K), CV(cal/mol·K)

	E(DZ)	G'corr (Mor 1M)	CV	S	E(TZ,PCM)	G(E(TZ,PCM) +G'corr)	ΔE(TZ,PCM)	ΔG(E(TZ),PCM+G'corr)
**1**	-2009.35388	0.77405	185.493	274.848	-2011.4613	-2010.68726	0	0
**2**	-2033.77216	0.83433	193.461	284.295	-2035.9008	-2035.06647	10.2	9
**3‡**	-2033.76147	0.83911	191.482	272.942	-2035.88791	-2035.05061	17.2	19
**4**	-2281.93297	0.92484	213.577	299.717	-2284.32509	-2283.40025	-5.2	0.1
**5‡**	-2033.7595	0.83788	191.651	279.742	-2035.88525	-2035.04737	20	21
**6**	-2008.14598	0.75377	184.737	278.932	-2010.24654	-2009.49277	-17	-15.2
**7**	-2239.06615	0.83161	203.657	291.264	-2241.40847	-2240.57686	-14.5	-10.1
**8**	-2239.06305	0.82506	256.074	352.836	-2241.41257	2240.58751	-17	-16.7
**9**	-2239.09188	0.83375	203.123	285.456	-2241.43758	-2240.60383	-32.7	-27
**10**	-2400.65792	0.85939	211.124	295.014	-2402.97581	-2402.11642	-15.3	-8.9
**11‡**	-2400.63962	0.85596	211.543	300.704	-2402.95788	-2402.10192	-4.1	0.2
**12**	-2648.81893	0.93902	234.156	325.616	-2651.40378	-2634.5839	-33.1	-27
**1+prod**	-2896.93929	1.00776	249.125	405.391	-2899.80413	-2898.79637	-33.5	-32.2
**1**	-2009.35388	0.77405	185.493	274.848	-2011.4613	-2010.68726	0	0
**6**	-2008.14598	0.75377	184.737	278.932	-2010.24654	-2009.49277	22	19.2
**7**	-2239.06615	0.83161	203.657	291.264	-2241.40847	-2240.57686	24.6	24.4
**7'**	-2648.8133	0.94637	234.143	319.099	-2651.39635	-2650.44998	10.6	16.7
**8**	-2239.06305	0.82506	256.074	352.836	-2241.41257	2240.58751	6.3	17.7
**9**	-2239.09188	0.83375	203.123	285.456	-2241.43758	-2240.60383	6.3	7.4
**10**	-2400.65792	0.85939	211.124	295.014	-2402.97581	-2402.11642	23.7	25.5
**11‡**	-2400.63962	0.85596	211.543	300.704	-2402.95788	-2402.10192	34.9	34.6
**12**	-2648.81893	0.93902	234.156	325.616	-2651.40378	-2634.5839	5.9	7.4

**1+prod**	-2896.93929	1.00776	249.125	405.391	-2899.80413	-2898.79637	5.5	2.3
**18‡-o**	-2239.03415	0.82689	203.211	290.249	-2241.37848	-2240.55159	4.4	5.8
**18‡-m**	-2239.02824	0.82696	201.211	289.325	-2241.3751	-2240.54813	6.5	8.0
**18‡-p**	-2239.02599	0.82507	201.121	292.498	-2241.37512	-2240.55005	6.5	6.8
H_2	-1.17393	0.01583	4.968	31.228	-1.17974	-1.16391		
NBE	-272.56072	0.14237	22.337	73.03	-272.85549	-272.71312		
NBA	-273.80266	0.1655	23.724	74.987	-274.0974	-273.9319		
py	-248.12242	0.08018	16.079	68.625	-248.39969	-248.31951		
Ph-py	-479.04528	0.15388	33.171	88.383	-479.56572	-479.41185		
$HSiR_3$	-409.72219	0.11178	26.304	81.301	-409.96561	-409.86092		

Figure S1. Geometrical representation for the DFT optimized structures. Only most relevant hydrogen atoms are shown for clarity.

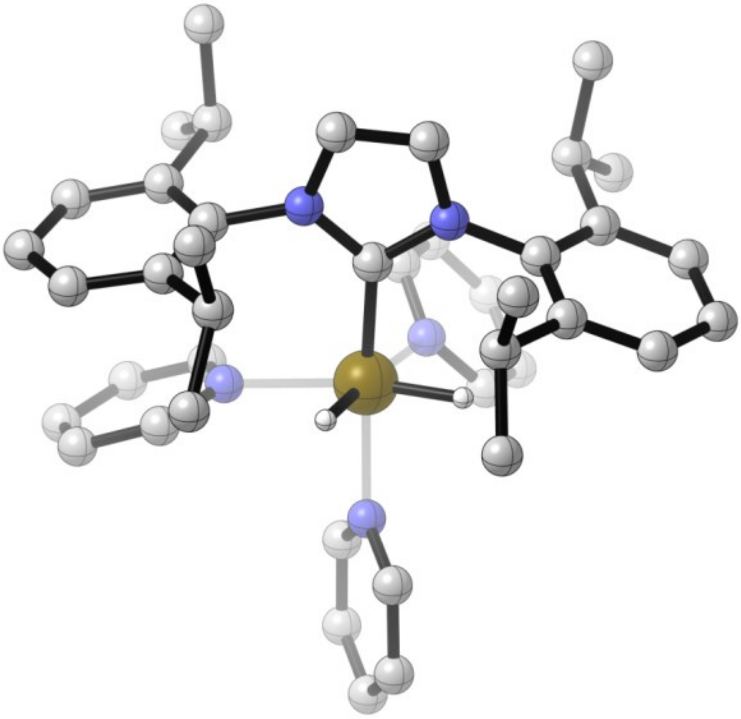

1

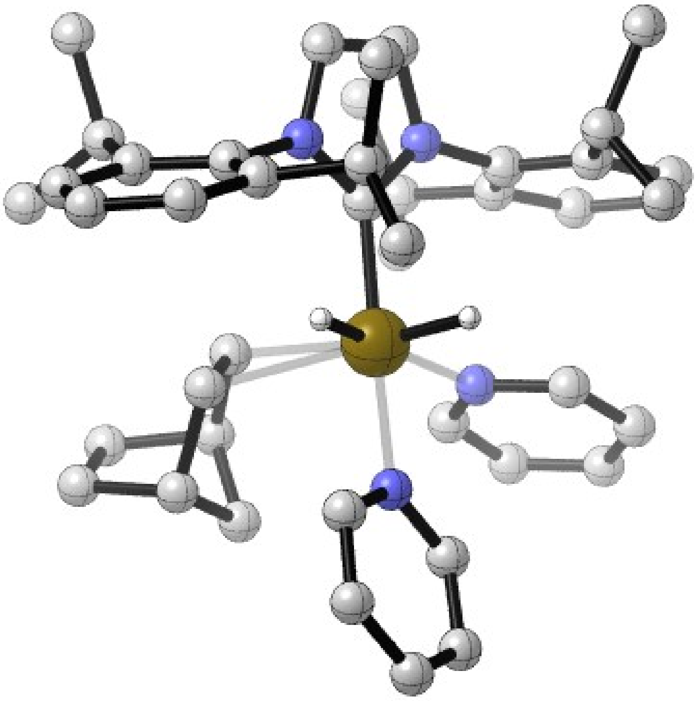

2

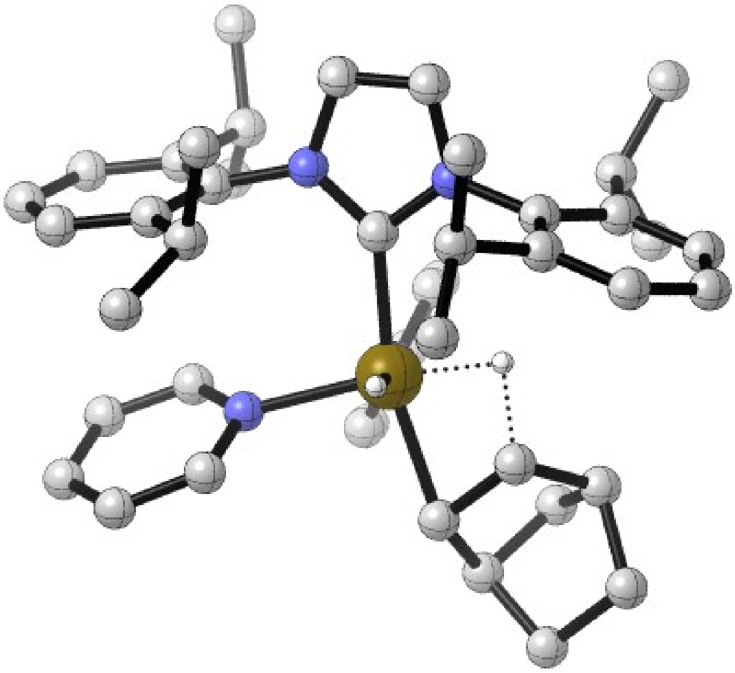

3‡

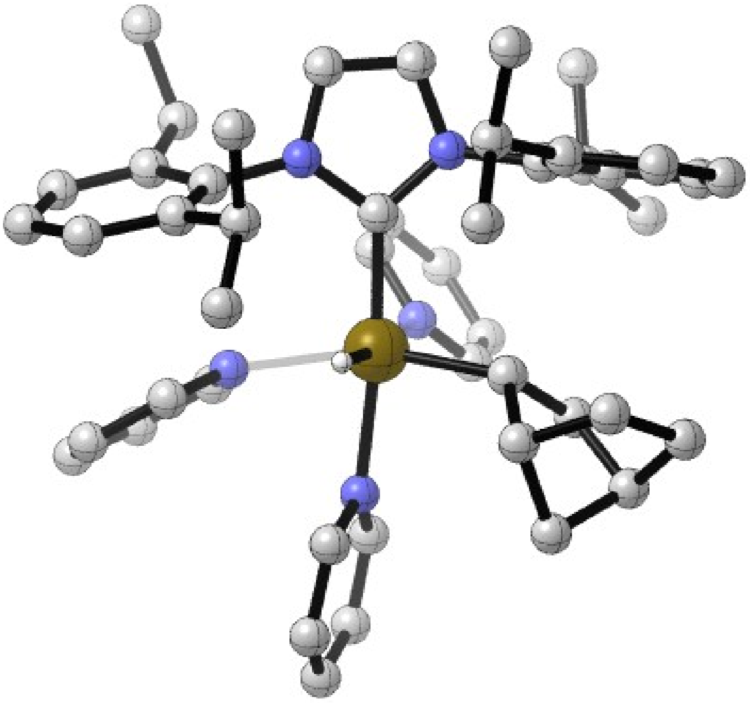

4

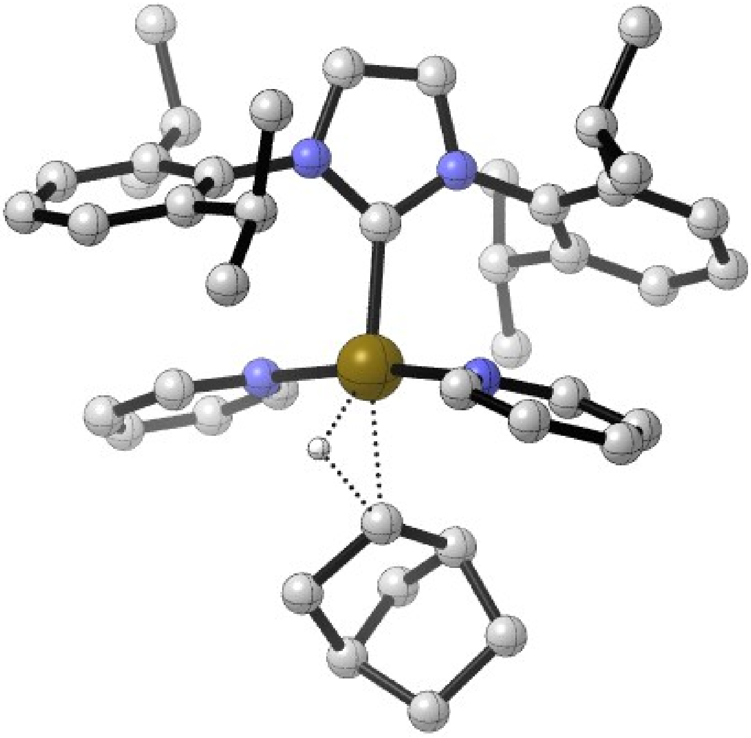

5‡

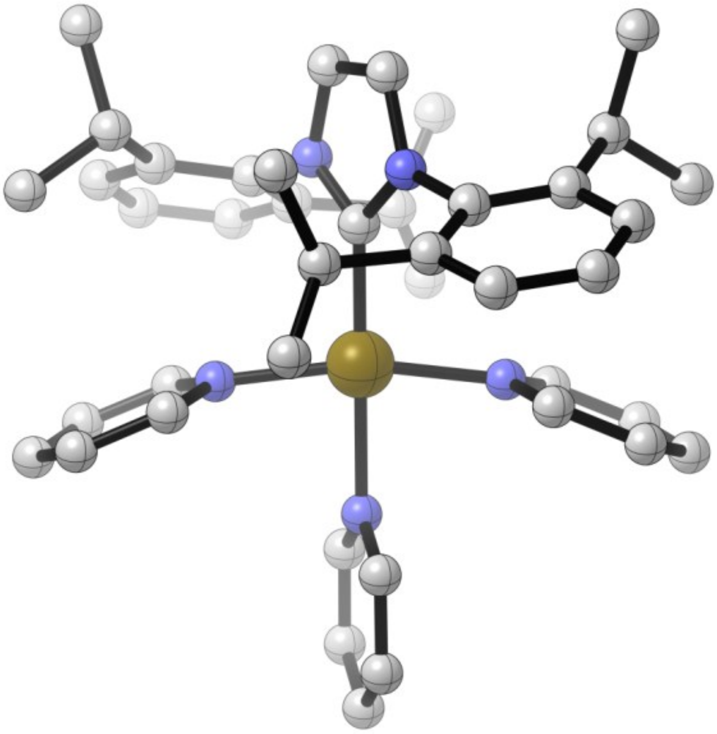

6

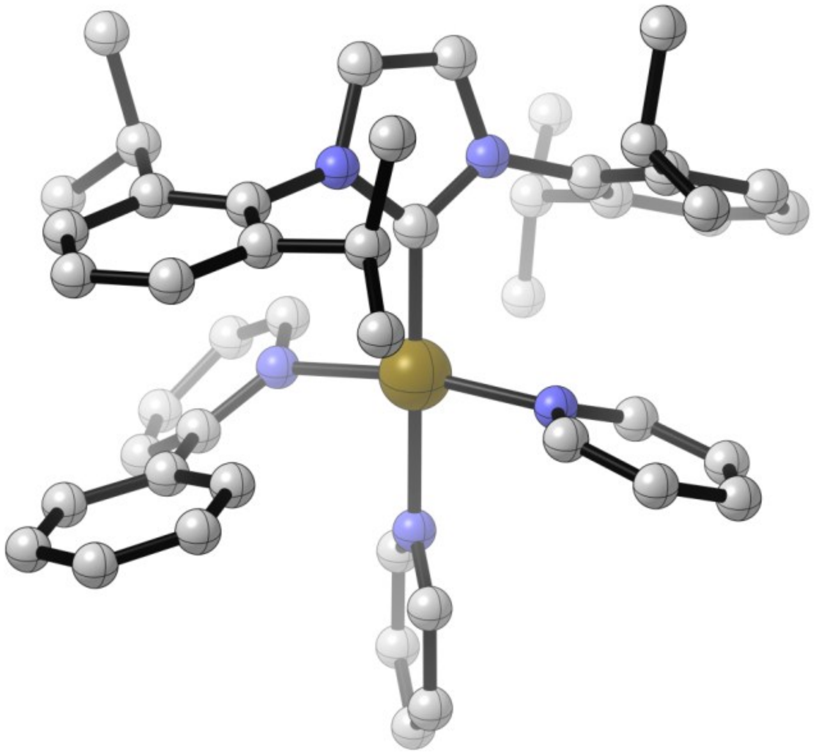

7

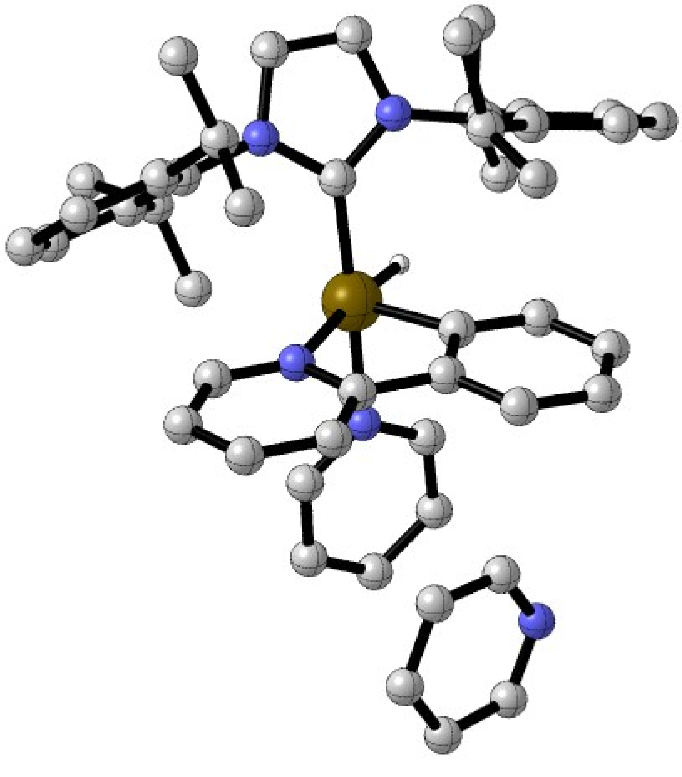

8

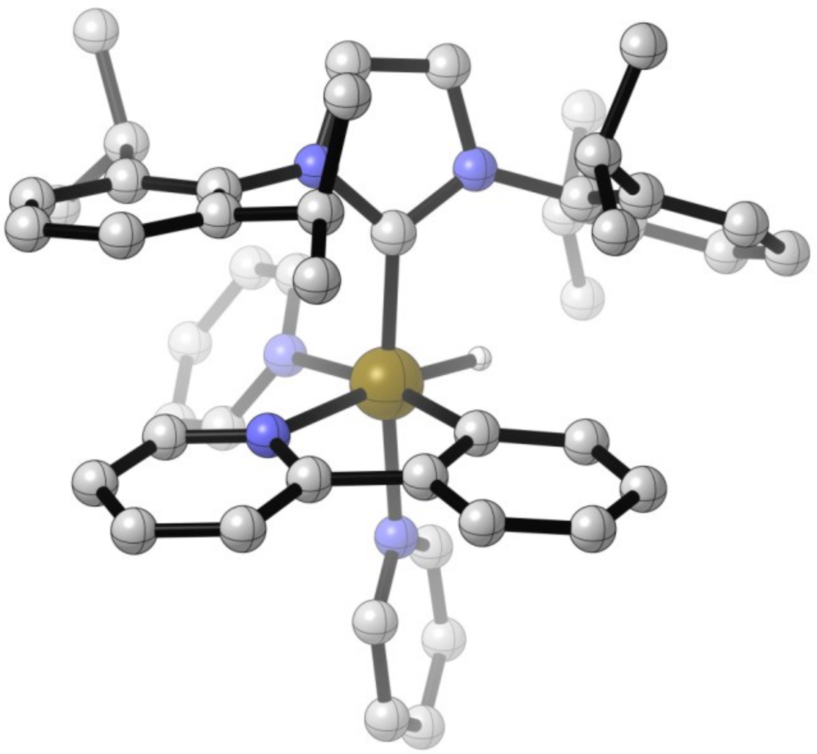

9

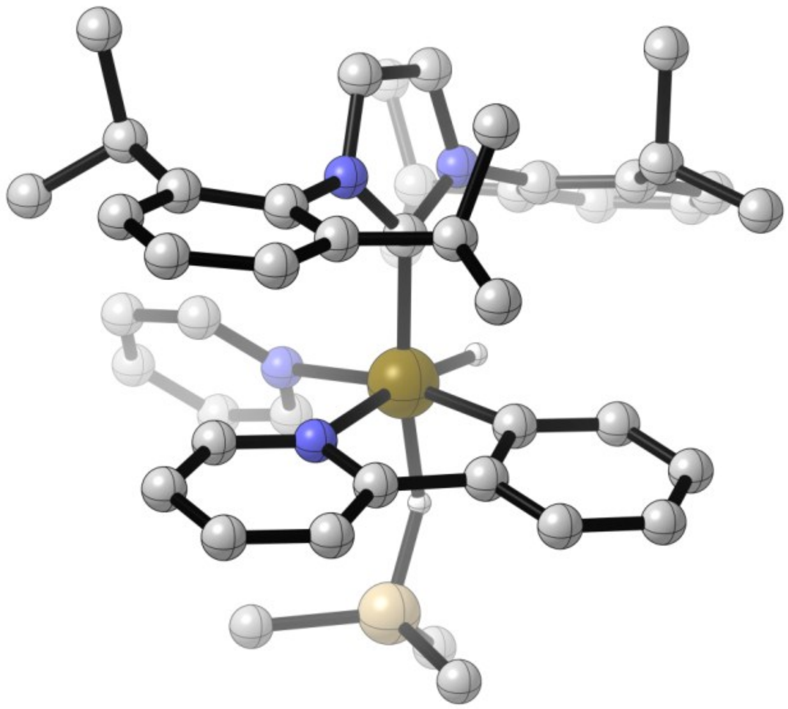

10

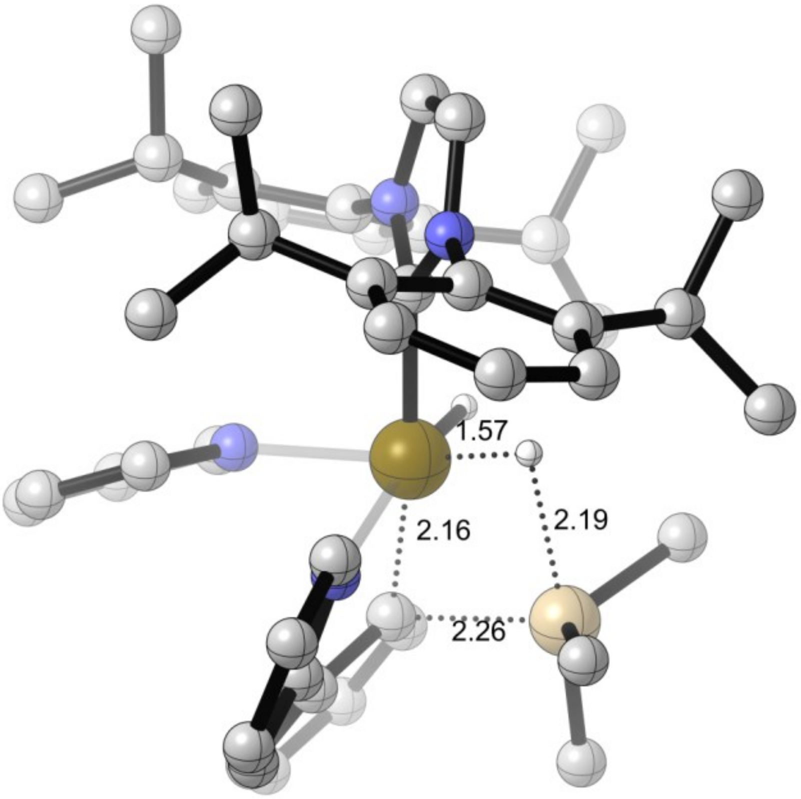

11‡

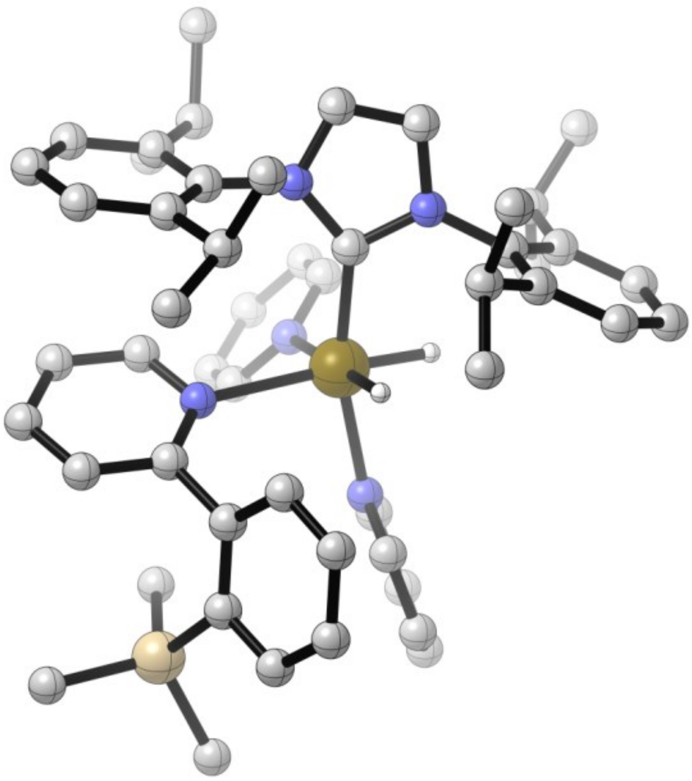

12

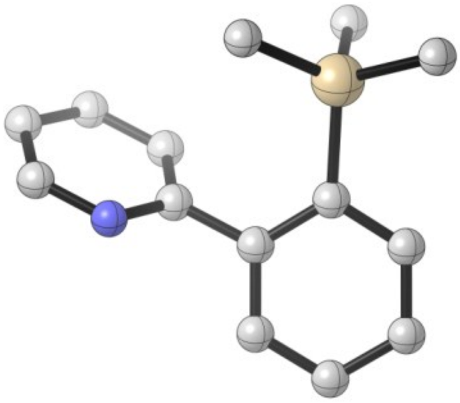

Prod

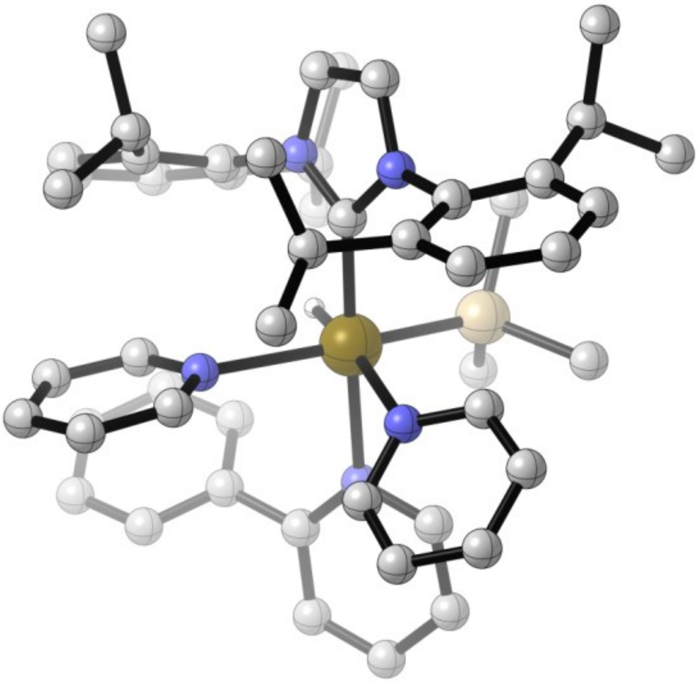

7’

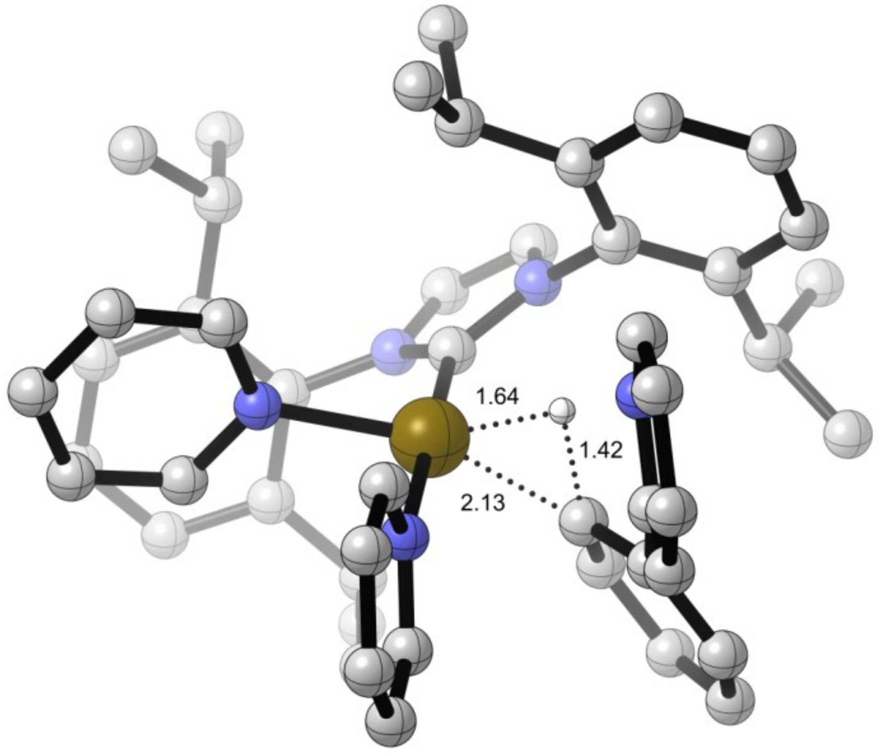

18‡-o

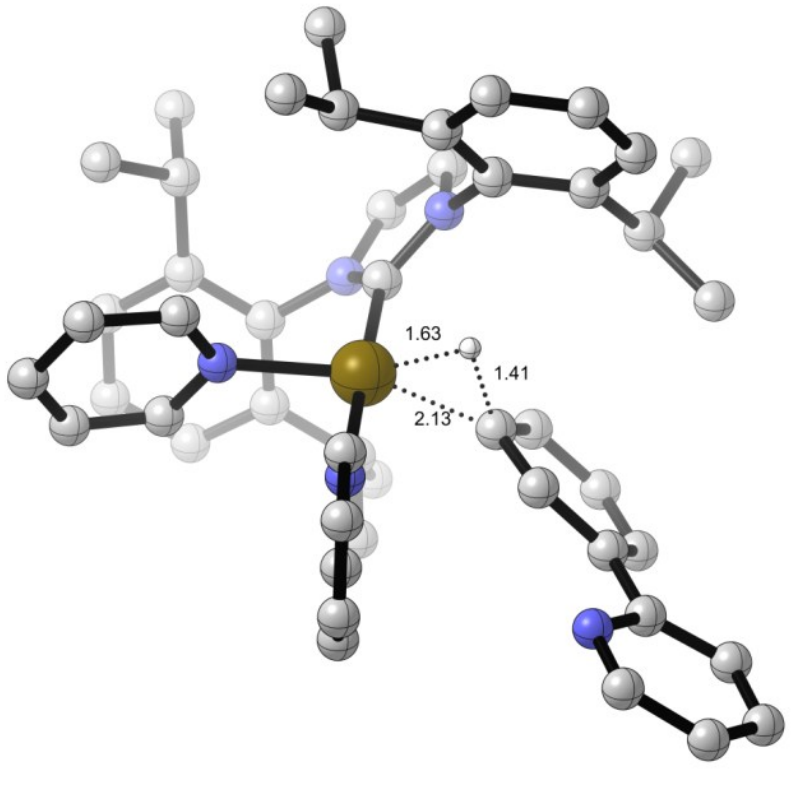

18‡-m

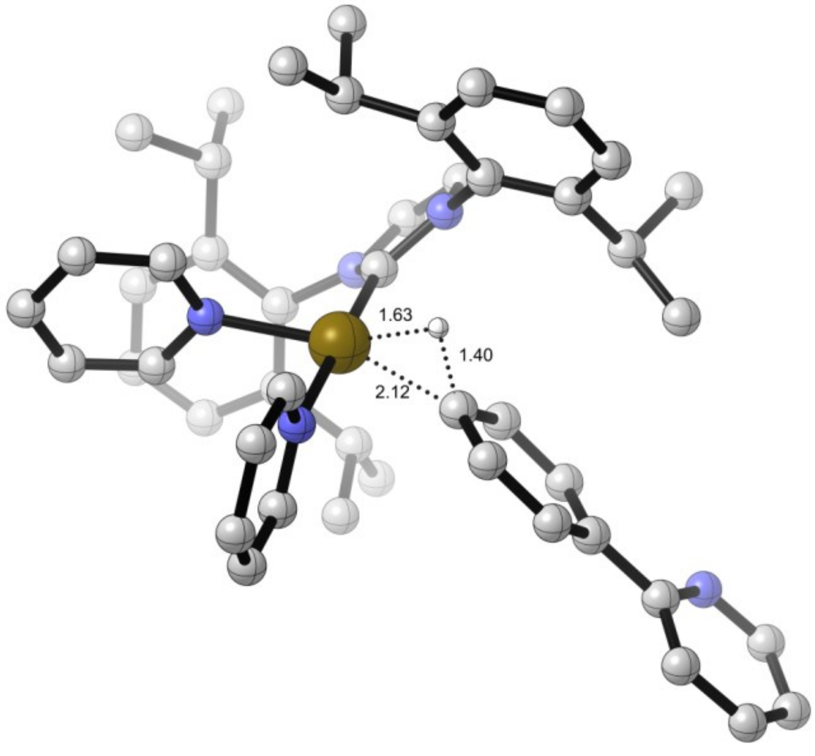

18‡-m

p

Figure S3. Energy variation (in kcalmol^{-1}) with the C-H distance (from 1.1 Å to 2.6 Å in increments of 0.1 Å) for the oxidative addition of phenyl-pyridine C–H bond at intermediate **E** to form structure **F**.

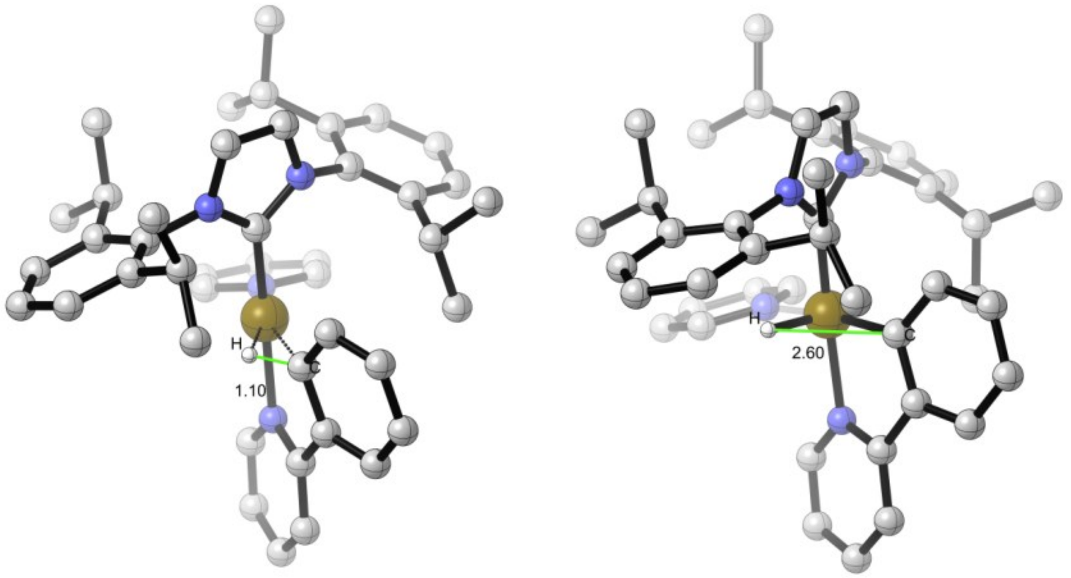

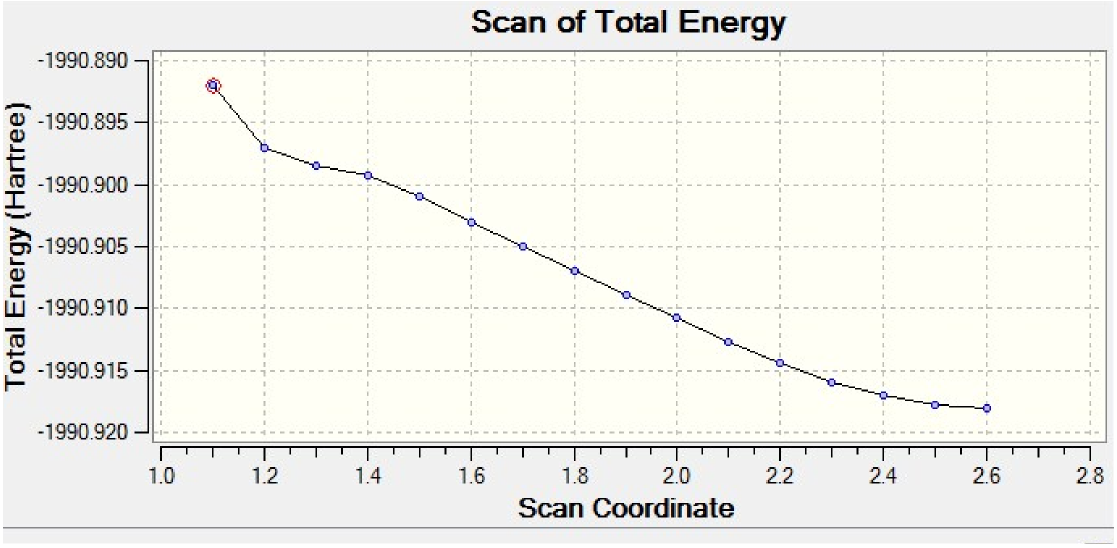

Figure S4. Energy variation (in kcalmol^{-1}) with the H-H distance (from 0.72 Å to 2.22 Å in increments of 0.1 Å) for the oxidative addition of the H-H bond at intermediate **D'** to form complex **A**.

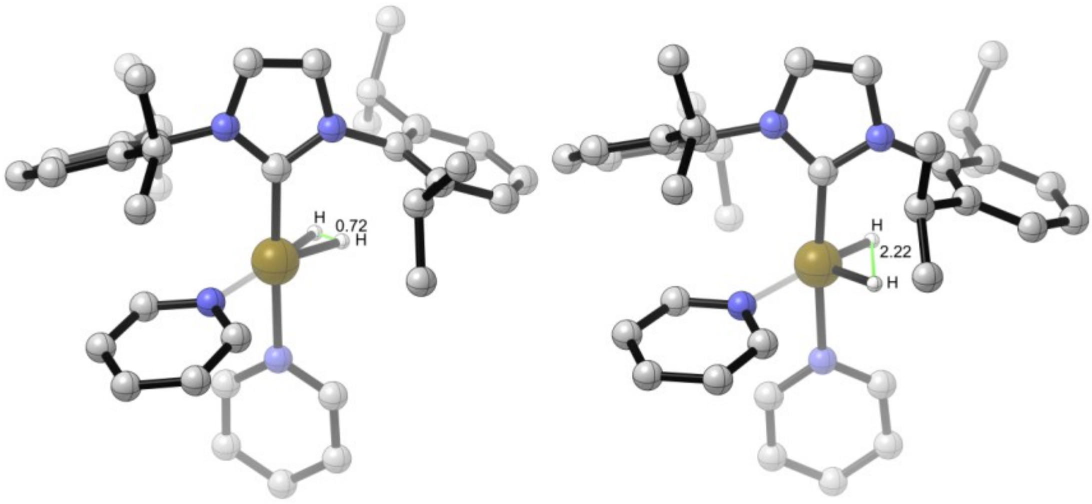

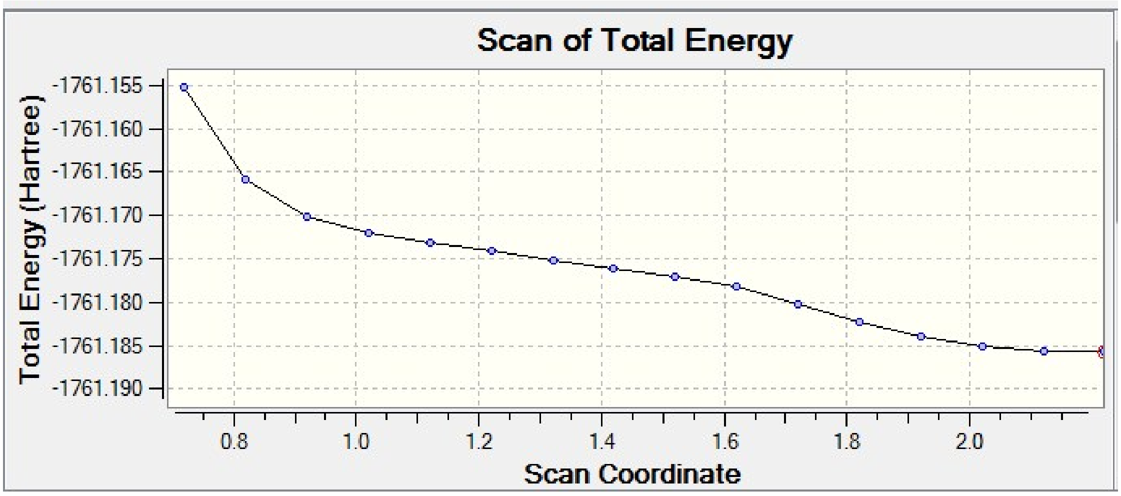

Figure S5. Gibbs free energy profile (relative to **1** and isolated molecules) for the directed orto CH bond activation and non-directed orto, meta, para CH bond activation.

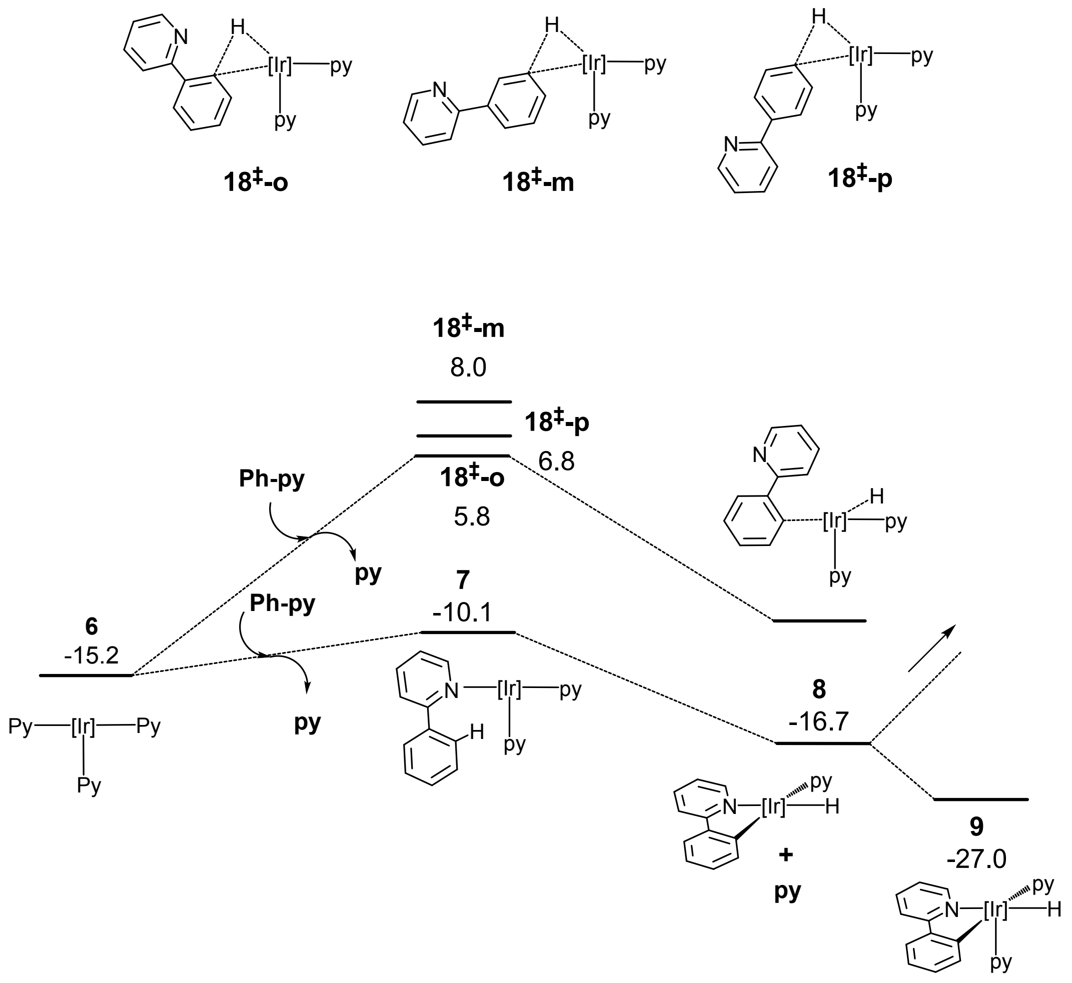

Table S2. Cartesian coordinates (in Å) for all calculated structures:

1

77	-0.026351	0.705547	0.508690
1	0.486042	0.019126	1.844298
1	-1.483875	0.648259	1.084071
6	-0.118037	2.732413	2.788556
6	0.769388	3.697502	0.884603
6	0.033584	3.914559	3.507030
1	-0.539338	1.837086	3.244264
6	0.955214	4.915603	1.533531
1	1.051163	3.575895	-0.160825
1	-0.276547	3.950060	4.552468
1	1.388005	5.756019	0.988456
6	2.549359	1.702495	-1.220270
6	3.108623	0.832917	0.832886
6	3.850194	2.147227	-1.444094
1	1.773411	1.883251	-1.963640
6	4.433868	1.240151	0.692877
1	2.772264	0.313528	1.727135
1	4.092129	2.668387	-2.371930
1	5.147933	1.025427	1.489189
6	-0.505120	1.360174	-2.554608
6	-1.590069	2.846814	-1.163395
6	-0.881529	2.073258	-3.690082
1	0.084563	0.446427	-2.633485
6	-2.006471	3.621209	-2.245128
1	-1.866130	3.107524	-0.140801
1	-0.584808	1.713872	-4.676733
1	-2.617200	4.508349	-2.069933
7	-0.827265	1.748409	-1.307598
7	0.243653	2.618422	1.493859
7	2.173882	1.052055	-0.106963
6	-1.635568	3.237981	-3.534929

1 -1.943586 3.824643 -4.402904
6 0.581741 5.031417 2.872768
1 0.714188 5.972080 3.411508
6 4.818500 1.910565 -0.467415
1 5.849162 2.243335 -0.607962
6 -0.186465 -3.364711 -0.690230
6 -1.508544 -3.111140 -0.548932
1 0.349195 -4.278221 -0.923614
1 -2.375301 -3.757828 -0.629367
6 -0.398800 -1.152226 -0.144808
7 0.478557 -2.165686 -0.451180
7 -1.630096 -1.763837 -0.227933
6 -2.902021 -1.205380 0.151614
6 -3.761300 -0.711257 -0.846332
6 -3.255034 -1.236518 1.515842
6 -5.008345 -0.217287 -0.438629
6 -4.512077 -0.733809 1.870753
6 -5.379705 -0.225988 0.904722
1 -5.702991 0.173708 -1.182953
1 -4.818492 -0.742646 2.918108
1 -6.357623 0.160683 1.200583
6 1.912210 -2.105127 -0.383156
6 2.530318 -2.382932 0.851740
6 2.640853 -1.853200 -1.562183
6 3.930866 -2.367358 0.889692
6 4.037564 -1.827247 -1.466821
6 4.675908 -2.078767 -0.252253
1 4.445688 -2.584606 1.827247
1 4.636449 -1.625034 -2.355799
1 5.766900 -2.062233 -0.199899
6 1.940445 -1.716144 -2.906803
1 0.911345 -1.381624 -2.703672
6 1.722467 -2.767908 2.083737
1 0.681773 -2.468826 1.906006
6 -3.385610 -0.759082 -2.320114
1 -2.286253 -0.752108 -2.376516
6 -2.327845 -1.822502 2.569774
1 -1.323564 -1.879338 2.132582
6 2.175475 -2.051105 3.360780
1 1.526628 -2.338886 4.202356
1 3.207777 -2.311669 3.641799
1 2.107801 -0.958416 3.253679
6 1.743239 -4.294249 2.274344
1 1.371614 -4.817417 1.380791
1 2.765564 -4.654377 2.471754
1 1.109188 -4.586336 3.126274
6 2.593231 -0.700636 -3.851414
1 3.577243 -1.047126 -4.202963
1 1.966468 -0.559673 -4.745817
1 2.736942 0.276232 -3.372174
6 1.845598 -3.087919 -3.599950
1 1.319256 -3.825815 -2.979466
1 1.305628 -3.003700 -4.556257
1 2.852043 -3.482871 -3.809970
6 -2.756726 -3.251808 2.938856
1 -2.056222 -3.691366 3.666974
1 -3.762678 -3.260434 3.387943
1 -2.779721 -3.905871 2.054051
6 -2.211447 -0.934851 3.813890
1 -1.431362 -1.325190 4.486158
1 -1.940159 0.093028 3.532871
1 -3.150530 -0.898232 4.387652
6 -3.880284 -2.062443 -2.972316
1 -3.593784 -2.097882 -4.035514
1 -3.463497 -2.953308 -2.482804
1 -4.978121 -2.130398 -2.913891
6 -3.903102 0.450128 -3.106495
1 -3.467946 0.462483 -4.117174
1 -4.996834 0.416164 -3.228031
1 -3.649194 1.396290 -2.610411

2
77 0.082941 0.627144 0.489417
1 1.442923 0.240632 1.146924
1 -0.558355 0.040806 1.807837
6 1.409699 2.727976 2.263098
6 -0.722524 3.430734 1.733737
6 1.625988 3.845581 3.063430
1 2.179852 1.968200 2.137433
6 -0.586279 4.575293 2.515230
1 -1.646345 3.245263 1.190884
1 2.583708 3.961312 3.573140
1 -1.415598 5.281368 2.580025
6 -2.389096 1.970661 -1.166990
6 -3.002726 1.091538 0.868473
6 -3.636848 2.568127 -1.337721
1 -1.620868 2.068924 -1.926796
6 -4.278470 1.639255 0.772257
1 -2.711200 0.505929 1.736707
1 -3.837087 3.147758 -2.240280
1 -4.998986 1.462930 1.571869
7 -2.060318 1.255943 -0.079225
7 0.253309 2.513138 1.601163
6 -4.605335 2.403718 -0.348292
1 -5.594183 2.855882 -0.450260
6 0.610583 4.793511 3.197028
1 0.749184 5.680841 3.818268
6 0.875366 -3.407364 -0.522446
6 -0.466083 -3.424347 -0.693451
1 1.616461 -4.196570 -0.580987
1 -1.152150 -4.229714 -0.932294
6 0.118039 -1.277410 -0.166924
7 1.225821 -2.097923 -0.214855
7 -0.913681 -2.122405 -0.486572
6 -2.323039 -1.843520 -0.436230
6 -2.974768 -1.365797 -1.592672
6 -3.002935 -2.154745 0.755953
6 -4.360765 -1.187473 -1.515951
6 -4.392914 -1.970175 0.774384
6 -5.062826 -1.490474 -0.347309
1 -4.906709 -0.812931 -2.381393
1 -4.955871 -2.209143 1.678795
1 -6.145920 -1.351694 -0.316389
6 2.580814 -1.758717 0.134012
6 2.927801 -1.727649 1.501376
6 3.514383 -1.528057 -0.894822
6 4.247406 -1.391160 1.824628
6 4.824975 -1.200967 -0.516570
6 5.184746 -1.120183 0.827027
1 4.550893 -1.352517 2.871941

1 5.575294 -1.012268 -1.285519
1 6.210205 -0.861191 1.100223
6 -2.287453 -2.725866 1.974353
6 -2.198833 -1.078164 -2.874110
6 1.933979 -2.113796 2.587821
6 3.154738 -1.693652 -2.365272
1 0.925792 -2.021243 2.165346
1 2.060698 -1.607785 -2.451197
1 -1.260874 -0.586480 -2.568844
1 -1.206423 -2.671399 1.789752
6 -1.831536 -2.373830 -3.622474
1 -1.287318 -2.135519 -4.549831
1 -1.195125 -3.040482 -3.027728
1 -2.742424 -2.927592 -3.899679
6 -2.928396 -0.140028 -3.842432
1 -2.250165 0.155400 -4.657331
1 -3.793603 -0.636862 -4.308744
1 -3.291455 0.772219 -3.352196
6 -2.649279 -4.208186 2.164391
1 -2.424758 -4.796402 1.261935
1 -2.080913 -4.640757 3.002542
1 -3.721603 -4.331763 2.384088
6 -2.555156 -1.924628 3.255535
1 -3.625677 -1.897836 3.511960
1 -2.024981 -2.382435 4.104912
1 -2.192265 -0.890091 3.163956
6 3.550893 -3.096870 -2.859840
1 3.260704 -3.232629 -3.913512
1 4.640594 -3.238881 -2.786297
1 3.072508 -3.893043 -2.272436
6 3.781912 -0.624007 -3.268160
1 4.872804 -0.747977 -3.347467
1 3.374755 -0.703305 -4.287923
1 3.582112 0.389361 -2.897546
6 1.984164 -1.191405 3.810203
1 1.177051 -1.453038 4.511872
1 2.934179 -1.280247 4.359824
1 1.848155 -0.139992 3.518109
6 2.135754 -3.585051 2.988710
1 2.038984 -4.255617 2.121318
1 3.135022 -3.743415 3.424437
1 1.386506 -3.888344 3.737274
6 1.907743 2.832629 -3.462709
6 0.618378 2.493493 -2.640309
6 2.097586 3.071540 -1.042510
6 2.926607 3.239915 -2.358920
1 2.243471 1.980036 -4.069219
1 1.702376 3.666680 -4.149864
1 3.834379 2.619581 -2.356280
1 3.245151 4.286661 -2.471627
6 0.714987 3.549962 -1.525606
1 -0.068810 3.457598 -0.767342
1 0.729180 4.580686 -1.909081
6 0.967993 1.217066 -1.883949
6 1.898890 1.566424 -0.933088
1 -0.290309 2.473253 -3.255969
1 2.528767 3.572818 -0.167561
1 2.677989 0.912776 -0.548077
1 0.868966 0.227474 -2.327748

3‡

6 1.521293 1.724273 -2.117722
6 2.474875 0.819771 -1.499095
1 2.785428 -0.108154 -1.984244
1 0.498950 -0.662641 -1.795000
6 -0.182660 -1.034286 0.604832
6 0.161188 -2.769400 2.060419
6 -1.172583 -2.539318 2.014673
1 0.746621 -3.498703 2.610911
1 -2.004254 -3.027228 2.511539
7 0.751405 -1.856549 1.195985
7 -1.369441 -1.491584 1.115163
6 -2.702109 -1.115574 0.717346
6 -3.220410 -1.639907 -0.489875
6 -3.470089 -0.326038 1.593380
6 -4.551963 -1.339757 -0.798640
6 -4.803500 -0.069610 1.241976
6 -5.339173 -0.571826 0.060269
1 -4.988381 -1.719593 -1.721912
1 -5.429185 0.530257 1.905029
1 -6.380156 -0.364504 -0.197846
1 1.922822 0.211019 -0.245542
77 0.369230 0.487703 -0.725180
6 2.146737 -1.929207 0.846718
6 2.502194 -2.656705 -0.309102
6 3.100574 -1.302328 1.668858
6 3.858686 -2.703871 -0.651873
6 4.446617 -1.385800 1.286128
6 4.822552 -2.069827 0.132434
1 4.168393 -3.255401 -1.540933
1 5.211075 -0.909535 1.902754
1 5.875770 -2.120420 -0.152403
6 -2.561163 1.560804 -0.887679
6 -1.728269 0.792290 -2.898938
6 -3.737892 1.994482 -1.488539
1 -2.409490 1.676866 0.180239
6 -2.873246 1.201926 -3.575420
1 -0.908333 0.301512 -3.421381
6 -3.902171 1.818119 -2.861977
1 -4.511891 2.454586 -0.873984
1 -2.948024 1.032357 -4.650594
1 -4.813129 2.148234 -3.365471
1 1.196038 1.517018 -3.143436
6 0.187186 1.967867 2.094065
6 -0.356779 3.376193 0.350100
6 0.028356 2.987981 3.029091
1 0.472230 0.963168 2.399588
6 -0.552259 4.448154 1.216731
1 -0.482528 3.487929 -0.726715
6 -0.345972 4.256318 2.584786
1 0.195291 2.781513 4.087255
1 -0.848947 5.419343 0.817594
1 -0.478087 5.079525 3.290120
7 0.013950 2.155255 0.776476
7 -1.563409 0.973141 -1.572697
6 -2.906234 0.226996 2.894752
6 -2.382637 -2.524362 -1.406712
6 2.716249 -0.577846 2.947793
6 1.473013 -3.424524 -1.123945
1 1.618570 -0.513323 2.970469

1 0.483647 -3.023866 -0.874697
1 -1.365749 -2.105661 -1.413607
1 -1.813668 0.107620 2.859865
6 1.483374 -4.909004 -0.721051
1 0.713286 -5.467372 -1.275856
1 2.460030 -5.370083 -0.937553
1 1.284445 -5.034831 0.354104
6 1.652474 -3.258080 -2.637047
1 2.581273 -3.725393 -2.999176
1 0.818235 -3.740229 -3.170363
1 1.667397 -2.195293 -2.921671
6 3.142695 -1.386485 4.183666
1 2.824594 -0.881269 5.109279
1 2.701406 -2.394876 4.175467
1 4.237124 -1.503084 4.221820
6 3.274554 0.850982 2.994289
1 2.894139 1.386593 3.878114
1 4.373270 0.852706 3.061057
1 2.993350 1.423164 2.098701
6 -3.426157 -0.568480 4.103758
1 -2.985921 -0.187948 5.038816
1 -4.521243 -0.484757 4.187025
1 -3.180097 -1.637306 4.024406
6 -3.200677 1.723777 3.068005
1 -2.838551 2.317036 2.215127
1 -4.278337 1.916957 3.180672
1 -2.705295 2.107408 3.972357
6 -2.303108 -3.967605 -0.875400
1 -1.689234 -4.585791 -1.548356
1 -1.856270 -4.024849 0.125112
1 -3.308014 -4.415936 -0.827250
6 -2.871577 -2.547007 -2.858831
1 -3.817682 -3.100991 -2.964056
1 -3.023853 -1.536587 -3.260719
1 -2.128361 -3.056844 -3.490547
6 3.340512 3.215169 -2.817336
6 2.111493 3.121300 -1.867340
6 3.588512 1.691158 -0.923582
6 4.339569 2.192483 -2.192351
1 3.076095 2.973144 -3.856827
1 3.758347 4.233493 -2.811520
1 4.595803 1.367978 -2.874190
1 5.282605 2.675682 -1.897918
6 2.833238 2.961271 -0.511876
1 2.174357 2.808613 0.345571
1 3.509863 3.800000 -0.290623
1 1.417175 3.967288 -1.960062
1 4.226931 1.209662 -0.172594

4
77 -0.130303 0.746778 -0.214672
1 -0.271031 0.426018 -1.738107
6 -0.884083 3.103096 -1.998928
6 -0.877883 3.725871 0.221988
6 -1.337704 4.357456 -2.393582
1 -0.682955 2.319792 -2.727007
6 -1.324521 5.007409 -0.088652
1 -0.703280 3.435289 1.255544
1 -1.504133 4.558018 -3.452895
1 -1.485548 5.730105 0.712666
6 -3.094222 1.391326 1.125274
6 -3.176133 0.878575 -1.109789
6 -4.386397 1.913579 1.104255
1 -2.517653 1.406397 2.048864
6 -4.470853 1.378125 -1.226001
1 -2.658356 0.473083 -1.974771
1 -4.819751 2.320157 2.019692
1 -4.973283 1.347738 -2.193926
6 -0.675929 0.050776 2.828775
6 0.507158 2.014902 2.904081
6 -0.786891 0.001563 4.215182
1 -1.114566 -0.730022 2.213067
6 0.437139 2.055707 4.297359
1 1.043262 2.792079 2.362482
1 -1.314392 -0.830601 4.683502
1 0.908891 2.879845 4.834759
7 -0.043276 1.037495 2.166576
7 -0.649192 2.784353 -0.710186
7 -2.491923 0.866796 0.046005
6 -0.221083 1.029271 4.972785
1 -0.288655 1.027061 6.062820
6 -1.559651 5.336171 -1.423968
1 -1.909320 6.332450 -1.702486
6 -5.096986 1.904434 -0.096150
1 -6.111909 2.304134 -0.150712
6 -0.318691 -3.466912 0.461090
6 0.982730 -3.301212 0.773475
1 -0.952397 -4.346351 0.462650
1 1.742310 -4.010805 1.083254
6 0.185335 -1.245834 0.123808
7 -0.799197 -2.221876 0.067316
7 1.292393 -1.961074 0.554172
6 2.700493 -1.645807 0.424262
6 3.421132 -1.119226 1.513501
6 3.319822 -2.015572 -0.788788
6 4.788300 -0.880219 1.326002
6 4.690267 -1.760737 -0.920934
6 5.415385 -1.183122 0.118104
1 5.377150 -0.455960 2.139076
1 5.195753 -2.017660 -1.853691
1 6.481445 -0.981731 -0.006667
6 -2.133700 -2.137342 -0.472392
6 -2.287085 -2.090212 -1.874928
6 -3.236550 -2.267608 0.398303
6 -3.590560 -2.090069 -2.387408
6 -4.518933 -2.236827 -0.165524
6 -4.697802 -2.138035 -1.542812
1 -3.740983 -2.057000 -3.467291
1 -5.392010 -2.319063 0.484048
1 -5.706203 -2.125626 -1.962368
6 2.564446 -2.682409 -1.932372
6 2.760135 -0.884004 2.861937
6 -1.096137 -2.144138 -2.820727
6 -3.089094 -2.562947 1.886622
6 1.878490 1.216264 -0.679322
6 2.543818 2.256954 0.263955
1 2.437599 0.287660 -0.605665
1 3.126287 1.786988 1.069867
1 1.795131 2.914768 0.729906
1 1.723130 -0.597230 2.658641

1 1.524857 -2.826288 -1.618021
1 -0.219732 -1.776482 -2.277188
1 -2.032230 -2.433025 2.158386
6 -1.249818 -1.247824 -4.053783
1 -0.316223 -1.249154 -4.635665
1 -2.052630 -1.589766 -4.725163
1 -1.460448 -0.205294 -3.768786
6 -0.826051 -3.603842 -3.225391
1 -1.688189 -4.029438 -3.762770
1 0.051688 -3.668456 -3.886280
1 -0.633409 -4.235499 -2.345238
6 -3.448183 -4.033430 2.169913
1 -4.510304 -4.225546 1.952570
1 -2.858044 -4.727882 1.553977
1 -3.269394 -4.280015 3.228272
6 -3.921051 -1.635761 2.781752
1 -3.742815 -1.870910 3.842843
1 -3.675417 -0.579578 2.616584
1 -4.999750 -1.758085 2.600441
6 3.135173 -4.078478 -2.225262
1 3.138964 -4.707956 -1.322088
1 2.530417 -4.586349 -2.992689
1 4.169809 -4.023618 -2.598257
6 2.522508 -1.816971 -3.198926
1 2.015771 -0.861308 -3.010397
1 3.531760 -1.598898 -3.579492
1 1.976772 -2.343575 -3.996388
6 2.728303 -2.182745 3.687646
1 3.750243 -2.537877 3.894594
1 2.225550 -2.012186 4.653067
1 2.190302 -2.988342 3.168956
6 3.408696 0.237593 3.679538
1 4.425851 -0.029885 4.004978
1 3.469873 1.176817 3.110848
1 2.819897 0.426659 4.588576
6 3.391666 3.137948 -0.673417
6 2.215439 1.825538 -2.083175
6 2.393028 3.338758 -1.826348
1 2.823317 3.868603 -2.691039
1 1.488291 3.871236 -1.510633
6 4.486245 2.277484 -1.337528
1 5.251238 2.908352 -1.814737
1 4.999838 1.636048 -0.605378
6 3.687350 1.450134 -2.387382
1 3.953906 1.746361 -3.414737
1 3.873378 0.375279 -2.295719
1 3.768000 4.053954 -0.193796
1 1.523579 1.544947 -2.889042

5‡

77 0.097770 0.702098 -0.395608
1 -0.122054 1.706902 -1.605923
6 -2.712442 1.718674 -0.480810
6 -1.746406 1.946448 1.611702
6 -3.810049 2.482818 -0.099035
1 -2.638272 1.289805 -1.478621
6 -2.808318 2.723499 2.063216
1 -0.903650 1.711823 2.257152
1 -4.612490 2.654347 -0.817251
1 -2.800057 3.095019 3.088772
6 3.087383 0.378235 -0.564406
6 2.019667 0.051840 -2.592873
6 4.333941 0.410046 -1.181893
1 2.992353 0.498604 0.512017
6 3.229400 0.068728 -3.279264
1 1.074099 -0.074722 -3.114893
1 5.224775 0.548392 -0.568436
1 3.231275 -0.063820 -4.362078
7 1.939590 0.223632 -1.254703
7 -1.676622 1.472467 0.350644
6 4.414250 0.263934 -2.566350
1 5.377933 0.292608 -3.078842
6 -3.858160 3.013770 1.190299
1 -4.701747 3.626784 1.513647
6 -1.318047 -3.252193 0.615994
6 -0.121504 -3.299924 1.248277
1 -2.104323 -3.991606 0.515820
1 0.357119 -4.086528 1.821169
6 -0.302598 -1.234811 0.289679
7 -1.423196 -1.986195 0.048961
7 0.490914 -2.069764 1.031619
6 1.821364 -1.779602 1.490204
6 1.994590 -0.865662 2.551801
6 2.898940 -2.449463 0.877484
6 3.305424 -0.598447 2.965927
6 4.189005 -2.162200 1.346470
6 4.392961 -1.239837 2.369154
1 3.481854 0.105403 3.779456
1 5.046758 -2.664525 0.896094
1 5.406342 -1.024646 2.715487
6 -2.599453 -1.560154 -0.663320
6 -2.534624 -1.422896 -2.066864
6 -3.784815 -1.347419 0.068334
6 -3.695372 -1.002325 -2.727180
6 -4.921993 -0.941656 -0.645388
6 -4.876809 -0.758572 -2.024168
1 -3.685834 -0.879372 -3.810125
1 -5.856493 -0.766884 -0.110083
1 -5.772402 -0.438845 -2.561734
6 2.702036 -3.484516 -0.222836
6 0.799434 -0.269193 3.280987
6 -1.279464 -1.800929 -2.840205
6 -3.881953 -1.603322 1.567249
6 0.807159 2.774668 -0.916913
1 1.508635 2.671601 -1.756429
6 -0.108080 4.007614 -1.175538
1 -1.154889 3.812770 -0.897438
1 -0.105539 4.309542 -2.234652
1 1.659744 -3.421595 -0.566626
1 -0.008602 -0.162851 2.544250
1 -2.859392 -1.671507 1.967291
1 -0.422285 -1.528350 -2.209494
6 -4.590518 -2.943686 1.835671
1 -5.635064 -2.905166 1.488476
1 -4.106680 -3.784204 1.317595
1 -4.600667 -3.167886 2.913670
6 -4.589418 -0.476809 2.329539
1 -5.648540 -0.388258 2.042537
1 -4.563485 -0.681009 3.411137
1 -4.109204 0.492332 2.154559

6 -1.232541 -3.321502 -3.071864
1 -1.273825 -3.878511 -2.124190
1 -2.080997 -3.650237 -3.692799
1 -0.302136 -3.605444 -3.589015
6 -1.125011 -1.041628 -4.160378
1 -1.167000 0.047722 -4.004630
1 -0.158528 -1.288286 -4.626670
1 -1.902103 -1.311854 -4.891513
6 2.933098 -4.902226 0.328891
1 2.723764 -5.657611 -0.444294
1 3.978049 -5.031077 0.652499
1 2.293096 -5.116999 1.197634
6 3.588711 -3.233487 -1.448578
1 3.379356 -3.987006 -2.223484
1 3.404875 -2.243465 -1.882096
1 4.660068 -3.305015 -1.205480
6 0.304926 -1.243575 4.364776
1 0.046582 -2.224324 3.938551
1 1.080866 -1.402281 5.130252
1 -0.591678 -0.844804 4.865224
6 1.067192 1.119242 3.868647
1 1.426896 1.822241 3.102263
1 0.142875 1.529757 4.304110
1 1.808558 1.090263 4.681375
6 1.920570 5.415971 -0.686695
6 0.475618 5.085370 -0.243039
6 1.634416 3.233822 0.333847
6 2.736029 4.173393 -0.208936
1 1.986184 5.573417 -1.774129
1 2.278739 6.337503 -0.204562
1 3.323803 3.704483 -1.012364
1 3.437100 4.443352 0.596872
6 0.715335 4.253619 1.028791
1 -0.207339 3.825674 1.438767
1 1.230286 4.808879 1.828510
1 -0.169951 5.968055 -0.131444
1 2.016871 2.424926 0.967830

6
77 -0.000227 0.808768 0.000008
6 0.723695 3.647571 -0.903475
6 -0.725088 3.647864 0.902244
6 0.750841 5.039511 -0.937015
1 1.291653 3.047303 -1.615829
6 -0.752616 5.039826 0.935025
1 1.353102 5.550568 -1.689795
1 -1.355008 5.551122 1.687537
6 -2.953574 1.269180 -0.110016
6 -1.937461 1.313598 -2.193627
6 -4.129716 1.800461 -0.631109
1 -2.862476 1.014931 0.944505
6 -3.078401 1.848476 -2.783710
1 -1.039940 1.106402 -2.772831
1 -4.979252 1.961507 0.033746
1 -3.079922 2.055174 -3.854953
6 1.937219 1.314633 2.193179
6 2.952825 1.270487 0.109304
6 3.077998 1.850348 2.782862
1 1.039973 1.107029 2.772671
6 4.128735 1.802630 0.629973
1 3.079664 2.057181 3.854078
1 4.977998 1.964105 -0.035131
7 1.859902 1.045228 0.870422
7 -0.000621 2.959540 -0.000429
7 -1.860363 1.044393 -0.870791
6 4.197719 2.109686 1.989320
1 5.106689 2.530500 2.424011
6 -0.000993 5.752857 -0.001192
1 -0.001139 6.845019 -0.001494
6 -4.198536 2.107184 -1.990520
1 -5.107665 2.527286 -2.425571
6 -0.603912 -3.375703 0.304681
6 0.606686 -3.375636 -0.300038
1 -1.243904 -4.189703 0.626544
1 1.247379 -4.189553 -0.620698
6 0.000450 -1.184426 0.000659
7 -0.967247 -2.043224 0.476953
7 0.968811 -2.043076 -0.474489
6 2.225868 -1.663006 -1.052701
6 3.399453 -1.872571 -0.302679
6 2.243740 -1.136686 -2.362379
6 4.621256 -1.522300 -0.896144
6 3.487799 -0.787022 -2.901497
6 4.666497 -0.978042 -2.176400
1 5.549463 -1.674156 -0.342693
1 3.543284 -0.375855 -3.909815
1 5.627311 -0.708693 -2.621089
6 -2.225071 -1.663399 1.053578
6 -3.397712 -1.873854 0.302335
6 -2.244619 -1.136462 2.362926
6 -4.620343 -1.524103 0.894382
6 -3.489455 -0.787474 2.900710
6 -4.667269 -0.979529 2.174466
1 -5.547863 -1.676611 0.339964
1 -3.546193 -0.376024 3.908854
1 -5.628726 -0.710703 2.618083
1 2.861601 1.015891 -0.945126
1 -1.292859 3.047828 1.614948
6 -3.372913 -2.493917 -1.088617
1 -2.331519 -2.486537 -1.441917
6 -0.967402 -1.023459 3.182050
1 -0.154043 -0.814194 2.473757
6 0.965625 -1.024990 -3.180316
1 0.153290 -0.813378 -2.471542
6 3.376680 -2.492171 1.088524
1 2.335646 -2.485786 1.442872
6 -3.845245 -3.957392 -1.035773
1 -3.769553 -4.425443 -2.029653
1 -3.251896 -4.558186 -0.331156
1 -4.896978 -4.016607 -0.713495
6 -4.199601 -1.703842 -2.110662
1 -4.095205 -2.156101 -3.109143
1 -5.272470 -1.707092 -1.863253
1 -3.870790 -0.659986 -2.175745
6 -0.990972 0.129490 4.189020
1 -1.729132 -0.027498 4.990436
1 -0.009495 0.223316 4.679502
1 -1.222963 1.086385 3.695049
6 -0.658056 -2.360113 3.877874
1 -1.452971 -2.624323 4.593506

1 -0.571952 -3.181816 3.151589
1 0.292405 -2.298377 4.431679
6 0.654518 -2.363188 -3.872426
1 1.448313 -2.629651 -4.588464
1 0.568771 -3.183079 -3.144073
1 -0.296632 -2.302141 -4.425128
6 0.988601 0.125307 -4.190292
1 1.222124 1.083335 -3.699238
1 1.725406 -0.034395 -4.992425
1 0.006451 0.218614 -4.679512
6 3.850568 -3.955137 1.035758
1 4.902107 -4.013335 0.712673
1 3.776177 -4.422941 2.029855
1 3.257334 -4.556831 0.331802
6 4.203531 -1.700796 2.109431
1 3.873353 -0.657388 2.174719
1 4.100978 -2.153002 3.108128
1 5.276101 -1.702608 1.860707

7
77 0.081095 0.703841 0.020517
6 -0.682628 3.365705 -1.393223
6 -0.735722 3.525937 0.911184
6 -1.055427 4.698843 -1.538586
1 -0.514584 2.736139 -2.266677
6 -1.118117 4.862675 0.852162
1 -0.592787 3.024834 1.868787
1 -1.173769 5.118676 -2.538672
1 -1.280429 5.416558 1.778046
6 1.567196 1.085196 -2.523276
6 2.685170 1.967301 -0.693843
6 2.453615 1.690514 -3.408249
1 0.745889 0.463943 -2.875221
6 3.614941 2.593509 -1.517456
1 2.742225 2.043601 0.389600
1 2.322754 1.541321 -4.481075
1 4.420050 3.172842 -1.064017
7 1.663672 1.231577 -1.182577
7 -0.520327 2.780204 -0.191236
6 3.499512 2.465099 -2.902185
1 4.212861 2.949262 -3.572193
6 -1.281525 5.467122 -0.395661
1 -1.580016 6.514536 -0.475554
6 1.814517 -2.945499 1.249697
6 0.574934 -3.386365 0.932241
1 2.667384 -3.455816 1.683592
1 0.126850 -4.369943 1.010316
6 0.654114 -1.159102 0.407937
7 1.864016 -1.596949 0.908098
7 -0.123458 -2.298306 0.415121
6 -1.415454 -2.432165 -0.205895
6 -1.476650 -2.428392 -1.617807
6 -2.554994 -2.630858 0.597532
6 -2.733070 -2.581935 -2.211955
6 -3.790092 -2.771960 -0.051204
6 -3.881787 -2.740302 -1.438669
1 -2.821731 -2.572320 -3.297914
1 -4.694334 -2.912870 0.542105
1 -4.854493 -2.841901 -1.923833
6 3.100950 -0.863047 0.915750
6 3.416637 -0.034307 2.007401
6 3.988973 -1.056353 -0.166521
6 4.643367 0.645048 1.980692
6 5.202828 -0.359181 -0.143710
6 5.526873 0.488713 0.915124
1 4.914291 1.296066 2.814374
1 5.908487 -0.483339 -0.966236
1 6.480666 1.021377 0.914400
6 -2.474076 -2.766284 2.111562
6 -0.214820 -2.328631 -2.466461
6 2.491773 0.124061 3.201624
6 3.679716 -2.027909 -1.297939
6 -1.205294 0.495658 2.585299
6 -2.853017 0.973961 0.992817
6 -1.970101 0.974378 3.637801
1 -0.222479 0.068349 2.761021
6 -3.635958 1.540903 2.021544
6 -3.198020 1.567594 3.338368
1 -4.601846 1.972804 1.769490
1 -3.814945 2.018421 4.118173
7 -1.580391 0.564835 1.284189
6 -3.457299 0.820477 -0.358258
6 -2.718000 0.718864 -1.547570
6 -4.865323 0.754305 -0.460220
6 -3.353938 0.630309 -2.785021
6 -5.499732 0.646614 -1.694898
1 -5.482597 0.752305 0.437923
6 -4.745769 0.604339 -2.870960
1 -2.752619 0.569661 -3.694749
1 -6.589577 0.590683 -1.737209
1 -5.240714 0.534496 -3.842202
1 -1.622769 0.684646 -1.478331
1 -1.532198 -2.300062 2.438040
1 0.477259 -1.661205 -1.934930
1 2.591698 -2.179371 -1.317361
1 1.608382 -0.500871 3.009222
6 -0.445877 -1.714802 -3.850166
1 -0.964146 -0.747010 -3.782920
1 0.519460 -1.556453 -4.355671
1 -1.044304 -2.369144 -4.502390
6 0.464447 -3.702940 -2.600419
1 0.718235 -4.130743 -1.620677
1 -0.197197 -4.414110 -3.119741
1 1.396731 -3.619001 -3.181497
6 -2.443046 -4.249543 2.524913
1 -2.344360 -4.345792 3.617728
1 -3.376238 -4.750795 2.223285
1 -1.612636 -4.799467 2.061447
6 -3.625171 -2.061484 2.840532
1 -4.576340 -2.599498 2.706357
1 -3.425326 -2.025085 3.922182
1 -3.769493 -1.035167 2.484626
6 4.094442 -1.507515 -2.679188
1 3.716777 -2.182968 -3.462647
1 3.698300 -0.503765 -2.873034
1 5.189129 -1.467759 -2.790574
6 4.340619 -3.393949 -1.039612
1 4.072584 -4.106914 -1.835006
1 5.437702 -3.295517 -1.026216
1 4.032788 -3.829570 -0.079813

6 3.145253 -0.398545 4.489641
1 3.458317 -1.448220 4.380758
1 4.036388 0.190079 4.758449
1 2.439460 -0.337809 5.333118
6 2.018640 1.576663 3.350485
1 2.862672 2.256591 3.545027
1 1.507191 1.903326 2.431496
1 1.314199 1.675738 4.191605
1 -1.591391 0.904643 4.658098

8

77 -0.129845 0.063777 -0.705235
1 0.059705 -1.107540 -1.758453
6 -2.784114 1.464578 -1.441402
6 -2.783316 -0.790438 -1.963032
6 -4.054197 1.632642 -1.984427
1 -2.253521 2.302638 -0.990168
6 -4.052544 -0.703224 -2.522693
1 -2.246208 -1.736387 -1.919374
1 -4.526277 2.615308 -1.954794
1 -4.528597 -1.601682 -2.915241
7 -2.150010 0.277777 -1.437305
6 -4.701976 0.531210 -2.541851
1 -5.703012 0.627935 -2.965402
6 3.930645 -1.109103 -0.458695
6 4.083971 0.229243 -0.604809
1 4.653028 -1.918327 -0.438166
1 4.966788 0.846985 -0.733565
6 1.848158 -0.187573 -0.389113
7 2.569986 -1.347101 -0.310458
7 2.808663 0.781366 -0.538832
6 2.610412 2.200182 -0.443097
6 2.827164 2.805942 0.809869
6 2.278782 2.930103 -1.599661
6 2.681071 4.197923 0.884768
6 2.133591 4.317721 -1.471350
6 2.335529 4.945534 -0.240180
1 2.842719 4.703342 1.838824
1 1.875056 4.917695 -2.345275
1 2.230482 6.029995 -0.161315
6 2.069888 -2.657787 0.008719
6 1.805134 -3.557540 -1.037225
6 1.935152 -2.994886 1.371951
6 1.397340 -4.851979 -0.684237
6 1.533092 -4.301621 1.669053
6 1.270252 -5.221337 0.653295
1 1.185071 -5.581729 -1.467560
1 1.410092 -4.604265 2.708891
1 0.958631 -6.236654 0.908949
6 2.036785 2.222196 -2.923061
6 3.240535 2.003155 2.035758
6 1.984237 -3.170981 -2.497199
6 2.213372 -1.980103 2.475175
6 -0.408861 2.898471 0.789203
6 -1.143069 1.015410 1.966103
6 -0.808079 3.762462 1.803269
1 0.057835 3.278580 -0.120046
6 -1.549796 1.833233 3.034137
6 -1.385502 3.212280 2.951299
1 -0.654102 4.836254 1.694370
1 -1.997631 1.386501 3.920842
1 -1.702687 3.853161 3.776726
6 -1.330068 -0.437012 1.901255
6 -2.002896 -1.164112 2.899964
6 -0.873545 -1.099108 0.730118
6 -2.240469 -2.526957 2.746885
1 -2.361279 -0.660494 3.799232
6 -1.122819 -2.470158 0.590971
6 -1.800679 -3.176016 1.587495
1 -2.771177 -3.080519 3.523344
1 -0.774248 -2.999281 -0.294585
1 -1.981791 -4.245086 1.456562
7 -0.568087 1.569833 0.866659
1 2.529100 1.240198 -2.865245
1 3.214019 0.938891 1.768407
1 1.928449 -0.992322 2.080849
1 2.094778 -2.077998 -2.539140
6 4.685358 2.334717 2.441278
1 4.782742 3.386185 2.754833
1 5.002463 1.701293 3.284212
1 5.385651 2.168537 1.608566
6 2.272319 2.184427 3.211399
1 2.596082 1.567424 4.063991
1 2.231859 3.229629 3.555541
1 1.254677 1.876061 2.940293
6 2.623034 2.962683 -4.130369
1 2.119119 3.925297 -4.308479
1 3.696139 3.162460 -3.992449
1 2.502290 2.358159 -5.042212
6 0.537565 1.968252 -3.109842
1 0.087238 1.498469 -2.206784
1 -0.020180 2.909539 -3.237548
1 0.327574 1.309116 -3.963442
6 0.760065 -3.532151 -3.348625
1 -0.155121 -3.081382 -2.935691
1 0.887458 -3.159964 -4.377054
1 0.608511 -4.620908 -3.410848
6 3.266691 -3.795578 -3.070618
1 3.213958 -4.895728 -3.051104
1 3.419203 -3.482630 -4.115542
1 4.154505 -3.495746 -2.492903
6 3.707602 -1.932895 2.841911
1 3.886663 -1.170535 3.616845
1 4.036668 -2.904924 3.241976
1 4.344567 -1.688848 1.981712
6 1.376874 -2.212193 3.737316
1 1.707500 -3.107279 4.287329
1 1.489243 -1.355332 4.419600
1 0.311346 -2.324995 3.504245
6 -4.805933 -0.681036 0.732370
6 -4.812410 0.611628 1.265853
6 -5.947221 1.404249 1.081374
6 -7.025172 0.871012 0.372951
6 -6.915942 -0.434429 -0.119482
7 -5.832294 -1.196350 0.049285
1 -5.991995 2.419057 1.484757
1 -3.928893 -1.322175 0.857878
1 -3.945393 0.982926 1.812750
1 -7.937634 1.448011 0.208165
1 -7.747247 -0.882622 -0.676302

9

77 -0.008336 -0.781980 0.413997
1 1.169664 -1.575397 -0.269515
6 -0.449962 -2.315137 3.104045
6 0.222692 -3.677065 1.361374
6 -0.449696 -3.386380 3.995035
1 -0.712136 -1.314605 3.440878
6 0.247409 -4.795943 2.186777
1 0.489886 -3.744946 0.307632
1 -0.721854 -3.215815 5.037709
1 0.539439 -5.762268 1.772853
7 -0.123860 -2.451729 1.806165
6 -0.095032 -4.653389 3.533278
1 -0.081118 -5.511777 4.208273
6 1.187193 1.421589 -2.982580
6 -0.064773 1.921591 -2.862939
1 1.955167 1.583200 -3.731703
1 -0.626370 2.606143 -3.488720
6 0.295217 0.560447 -1.062099
7 1.412269 0.616521 -1.872230
7 -0.593587 1.415509 -1.676166
6 -1.814619 1.958889 -1.126299
6 -1.712860 2.991222 -0.160983
6 -3.053031 1.541833 -1.649045
6 -2.904621 3.541207 0.322645
6 -4.216107 2.137621 -1.137175
6 -4.145034 3.117204 -0.153518
1 -2.864113 4.326921 1.076331
1 -5.189487 1.835459 -1.526437
1 -5.060528 3.568193 0.236007
6 2.757771 0.223063 -1.512332
6 3.230011 -1.066108 -1.822742
6 3.574023 1.198489 -0.903382
6 4.535049 -1.393559 -1.436518
6 4.877367 0.825488 -0.548531
6 5.349642 -0.460624 -0.794349
1 4.926074 -2.388489 -1.654645
1 5.530485 1.557609 -0.070449
1 6.365700 -0.734769 -0.501713
6 -3.163913 0.548397 -2.794513
6 -0.371184 3.568869 0.273751
6 2.391463 -2.046543 -2.621201
6 3.124281 2.639707 -0.710254
6 -2.817128 0.307767 1.501800
6 -0.909643 0.988048 2.676629
6 -3.673599 0.917325 2.409889
1 -3.205355 -0.209553 0.624427
6 -1.725005 1.618501 3.633926
6 -3.108505 1.583556 3.501590
1 -4.752216 0.874118 2.258591
1 -1.269673 2.126062 4.483252
1 -3.742868 2.073089 4.243712
6 0.556786 0.901833 2.745901
6 1.299349 1.487690 3.789869
6 1.203105 0.107302 1.756289
6 2.667777 1.258592 3.895168
1 0.807950 2.116121 4.535351
6 2.581112 -0.124506 1.901206
6 3.302637 0.433240 2.957398
1 3.237546 1.712155 4.708602
1 3.095671 -0.755729 1.177254
1 4.374078 0.235420 3.043831
7 -1.484711 0.336539 1.630244
6 -1.251784 -2.203851 -2.140142
6 -2.392111 -2.726440 -0.207724
6 -1.962488 -3.152442 -2.870602
1 -0.492637 -1.587157 -2.618388
6 -3.151862 -3.693202 -0.862265
1 -2.533058 -2.534536 0.856248
6 -2.928240 -3.922358 -2.220351
1 -1.755195 -3.278721 -3.934408
1 -3.903991 -4.255439 -0.306563
1 -3.499768 -4.678753 -2.762267
7 -1.442265 -1.998622 -0.825178
1 -2.197048 0.036240 -2.883523
1 0.346161 2.738209 0.337441
1 2.053724 2.691599 -0.933108
1 1.350764 -1.726735 -2.509092
6 0.131668 4.579437 -0.776805
1 -0.588985 5.405586 -0.880702
1 1.094565 5.009184 -0.463599
1 0.274124 4.127962 -1.766112
6 -0.392225 4.258722 1.642028
1 0.638236 4.475568 1.958246
1 -0.931659 5.218166 1.604928
1 -0.858229 3.637013 2.414716
6 -3.428220 1.281479 -4.121381
1 -4.393148 1.811513 -4.090120
1 -2.650022 2.026619 -4.341395
1 -3.460895 0.567716 -4.959684
6 -4.238887 -0.517783 -2.552959
1 -4.090720 -1.035995 -1.595202
1 -5.249573 -0.082154 -2.547812
1 -4.216231 -1.271642 -3.353461
6 2.481110 -3.485778 -2.103837
1 2.309037 -3.525442 -1.018100
1 1.727785 -4.117904 -2.600348
1 3.465337 -3.936038 -2.305865
6 2.745807 -1.964723 -4.115082
1 3.795355 -2.251317 -4.286909
1 2.108593 -2.642065 -4.706556
1 2.611848 -0.943871 -4.504650
6 3.842184 3.550697 -1.720929
1 3.462361 4.581889 -1.649310
1 4.926824 3.576576 -1.531125
1 3.695441 3.204459 -2.755436
6 3.302867 3.156459 0.721643
1 4.351239 3.102773 1.052620
1 2.998234 4.212891 0.778256
1 2.696621 2.584992 1.433905

10

77 -0.174626 0.243776 0.844708
1 -1.748358 0.214542 0.972933
6 -1.692594 -0.257052 -3.058983
6 -0.386035 -0.165580 -3.386921
1 -2.580349 -0.336046 -3.675404
1 0.111801 -0.171164 -4.348953
6 -0.518472 -0.131062 -1.104529
7 -1.766767 -0.265512 -1.672645
7 0.333033 -0.106253 -2.194386

6 1.774648 -0.217857 -2.239012
6 2.364651 -1.461821 -1.904798
6 2.533422 0.850739 -2.764197
6 3.758457 -1.555519 -1.986150
6 3.925172 0.691302 -2.839297
6 4.535492 -0.487788 -2.432270
1 4.248635 -2.489006 -1.714860
1 4.537181 1.502488 -3.235820
1 5.621374 -0.590563 -2.489129
6 -3.033223 -0.527661 -1.024823
6 -3.875086 0.556737 -0.709023
6 -3.418263 -1.873122 -0.832723
6 -5.120603 0.267110 -0.138392
6 -4.673897 -2.103832 -0.253554
6 -5.513703 -1.048759 0.095162
1 -5.796244 1.081098 0.125025
1 -5.004520 -3.127883 -0.080524
1 -6.488263 -1.254553 0.543475
6 1.920513 2.116766 -3.353000
6 1.532243 -2.695799 -1.568849
6 -3.475165 1.992633 -1.012725
6 -2.558869 -3.048087 -1.288202
6 2.961102 0.974075 0.581028
6 2.451429 -1.140028 1.436288
6 4.326717 0.789054 0.758632
1 2.574732 1.904557 0.165075
6 3.818525 -1.382482 1.655564
6 4.759304 -0.417771 1.315822
1 5.027784 1.571123 0.466494
1 4.138422 -2.328321 2.090373
1 5.822623 -0.605090 1.479829
6 1.373269 -2.080797 1.762298
6 1.614846 -3.319535 2.381767
6 0.046099 -1.663546 1.470752
6 0.557193 -4.152761 2.731109
1 2.634056 -3.637721 2.608493
6 -1.003390 -2.519857 1.839177
6 -0.755391 -3.745105 2.462993
1 0.750850 -5.110746 3.217517
1 -2.031451 -2.211422 1.644963
1 -1.590809 -4.389922 2.747748
7 2.052338 0.048143 0.908679
6 0.275825 3.130787 -0.382580
6 -1.082151 3.154373 1.478711
6 0.233361 4.519385 -0.488894
1 0.828687 2.526258 -1.095843
6 -1.165840 4.543199 1.456780
1 -1.617383 2.570498 2.225979
6 -0.495274 5.245580 0.454218
1 0.769424 5.015541 -1.297828
1 -1.763097 5.058489 2.210604
1 -0.544868 6.335327 0.404799
7 -0.361059 2.453311 0.585079
1 0.867732 2.172986 -3.039492
1 0.744564 -2.390725 -0.866935
1 -1.505293 -2.750894 -1.178047
1 -2.378353 2.039154 -0.971881
1 -0.313343 0.608602 2.595396
14 0.426673 0.977008 3.902489
6 -1.023685 1.604846 4.919215
1 -0.687820 1.810489 5.949270
1 -1.449785 2.537112 4.518480
1 -1.824525 0.850535 4.969866
6 1.153364 -0.572406 4.649598
1 0.463660 -1.424319 4.557888
1 2.105440 -0.850374 4.178874
1 1.340414 -0.389196 5.720992
6 1.698660 2.316403 3.591948
1 1.273474 3.169247 3.042450
1 2.066981 2.690543 4.561735
1 2.560970 1.932361 3.029127
6 0.855761 -3.259724 -2.833866
1 0.286173 -4.166619 -2.580035
1 0.157880 -2.552771 -3.297558
1 1.613155 -3.536021 -3.584254
6 2.332925 -3.821561 -0.905939
1 1.642567 -4.579239 -0.508989
1 2.995694 -4.322508 -1.629417
1 2.947748 -3.466050 -0.070921
6 1.935698 2.057990 -4.892320
1 1.446172 2.947351 -5.319117
1 2.969809 2.025998 -5.268570
1 1.420269 1.168445 -5.280826
6 2.633076 3.396769 -2.887131
1 3.644467 3.470895 -3.313058
1 2.079809 4.286253 -3.226187
1 2.736366 3.446457 -1.793334
6 -3.912616 2.401961 -2.430596
1 -3.607839 3.438893 -2.644551
1 -3.469776 1.757776 -3.202517
1 -5.007830 2.342543 -2.531019
6 -4.024564 2.993804 0.009924
1 -3.574011 3.984408 -0.150007
1 -5.114494 3.115682 -0.086134
1 -3.812392 2.679291 1.041794
6 -2.820079 -3.379933 -2.770618
1 -2.217164 -4.247574 -3.079226
1 -3.880868 -3.634472 -2.921678
1 -2.572705 -2.550233 -3.443262
6 -2.774158 -4.324798 -0.464647
1 -1.979913 -5.051786 -0.691989
1 -2.756430 -4.136195 0.614467
1 -3.733265 -4.805811 -0.712382

11‡

77 -0.000936 -0.601932 -0.376501
1 -0.966693 -0.184325 -1.532997
6 -0.349914 3.651918 -0.688039
6 0.989444 3.583812 -0.509518
1 -1.001908 4.486805 -0.917889
1 1.758056 4.348391 -0.526267
6 0.165307 1.465005 -0.276216
7 -0.844275 2.363541 -0.526891
7 1.287678 2.249508 -0.256067
6 2.612408 1.854425 0.132994
6 3.529225 1.442886 -0.855276
6 2.955177 1.988939 1.493814
6 4.819256 1.102456 -0.426508
6 4.270823 1.674244 1.862878
6 5.187099 1.220345 0.915236

1 5.556089 0.762606 -1.154821
1 4.576828 1.773373 2.905790
1 6.204402 0.966023 1.221205
6 -2.261505 2.110271 -0.582425
6 -3.024105 2.338590 0.582520
6 -2.838330 1.734407 -1.811173
6 -4.407350 2.133443 0.497317
6 -4.224575 1.527635 -1.836688
6 -5.000708 1.722191 -0.695052
1 -5.030409 2.294495 1.377310
1 -4.706620 1.231323 -2.769172
1 -6.080949 1.565413 -0.738962
6 1.950535 2.476757 2.530340
6 3.164269 1.470023 -2.333833
6 -2.390502 2.827518 1.879843
6 -2.018093 1.642026 -3.089723
6 -0.379351 -3.369745 0.698156
6 -1.205390 -4.414983 1.138295
6 -0.709744 -2.636491 -0.473544
6 -2.355998 -4.752162 0.426784
1 -0.970756 -4.950550 2.060366
6 -1.881209 -2.996649 -1.168906
6 -2.692811 -4.040909 -0.731436
1 -2.995063 -5.564563 0.778602
1 -2.151037 -2.452638 -2.076476
6 -1.290152 -0.715577 2.492400
6 -2.907772 -0.863657 0.850272
6 -2.180050 -1.139787 3.474645
1 -0.257980 -0.467335 2.737134
6 -3.863870 -1.287049 1.770763
1 -3.155080 -0.733493 -0.202407
6 -3.489493 -1.453794 3.103866
1 -1.845928 -1.219923 4.509970
1 -4.881761 -1.487626 1.434559
1 -4.210326 -1.803007 3.846337
7 -1.635630 -0.617399 1.197347
1 0.949803 2.413295 2.080125
1 2.081172 1.288350 -2.407130
1 -0.968970 1.487782 -2.802706
1 -1.353804 2.455297 1.897343
1 1.207445 -0.410419 -1.360891
14 0.970900 -2.502940 -1.975075
6 3.449485 2.864942 -2.922329
1 2.897903 3.658164 -2.399968
1 4.523389 3.100272 -2.854779
1 3.160266 2.898645 -3.984247
6 3.875925 0.401919 -3.170332
1 3.738509 -0.604685 -2.757701
1 3.477335 0.405209 -4.195845
1 4.956962 0.596849 -3.246145
6 2.206916 3.951205 2.883124
1 3.197182 4.077737 3.348736
1 2.172572 4.591722 1.989777
1 1.448670 4.317698 3.592886
6 1.924189 1.607936 3.795436
1 1.752013 0.547769 3.557856
1 2.866105 1.674389 4.361659
1 1.117955 1.943529 4.466040
6 -2.102255 2.966092 -3.869084
1 -3.139397 3.178196 -4.173563
1 -1.750798 3.815582 -3.264658
1 -1.483245 2.919010 -4.778769
6 -2.425307 0.459371 -3.975295
1 -2.420936 -0.482516 -3.406642
1 -3.429026 0.592663 -4.407261
1 -1.721522 0.354029 -4.814281
6 -2.346746 4.366062 1.937360
1 -1.878634 4.699897 2.876860
1 -1.777594 4.804077 1.108518
1 -3.367225 4.778890 1.902270
6 -3.096443 2.302455 3.135465
1 -2.489165 2.521531 4.027108
1 -4.071672 2.791385 3.284383
1 -3.264344 1.221050 3.094269
6 2.752463 -2.628583 -1.355097
1 3.361568 -3.014549 -2.189640
1 3.189174 -1.686733 -1.003708
1 2.820062 -3.366589 -0.541421
6 0.744456 -1.636872 -3.635793
1 -0.325101 -1.596106 -3.889963
1 1.131294 -0.610222 -3.649666
1 1.250129 -2.226121 -4.418431
6 0.594722 -4.319874 -2.402917
1 1.399127 -4.673733 -3.071325
1 0.573052 -4.970694 -1.516475
1 -0.363053 -4.429047 -2.931830
1 -3.594837 -4.302080 -1.289769
6 0.829069 -2.954784 1.428820
6 1.530609 -3.797185 2.304905
7 1.282653 -1.701393 1.162293
6 2.718156 -3.357947 2.883742
1 1.160689 -4.803581 2.499170
6 2.442926 -1.298020 1.693502
6 3.193782 -2.085142 2.561570
1 3.276339 -4.009856 3.559168
1 2.777988 -0.306784 1.408671
1 4.130834 -1.700100 2.965350

12

77 -0.153379 -0.492758 0.405150
6 -4.016847 -0.075238 -1.226505
6 -3.678067 1.228946 -1.331390
1 -4.931929 -0.603202 -1.473828
1 -4.226525 2.088917 -1.699645
6 -1.858008 0.127620 -0.471064
7 -2.909355 -0.738352 -0.717780
7 -2.359491 1.342749 -0.887529
6 -1.695861 2.611419 -1.045539
6 -0.898374 2.823797 -2.193273
6 -1.992117 3.640972 -0.131252
6 -0.401616 4.115858 -2.401251
6 -1.476460 4.918211 -0.393147
6 -0.694583 5.155432 -1.519355
1 0.220133 4.319355 -3.272181
1 -1.697316 5.738377 0.292135
1 -0.305957 6.157846 -1.712857
6 -2.892465 -2.178241 -0.653827
6 -3.435344 -2.828626 0.467975
6 -2.390665 -2.881884 -1.766223
6 -3.441218 -4.229559 0.471475

6 -2.414862 -4.280480 -1.714295
6 -2.930191 -4.950499 -0.605176
1 -3.854456 -4.763302 1.329554
1 -2.033116 -4.856208 -2.558904
1 -2.942183 -6.042573 -0.585233
6 -2.855075 3.411821 1.101154
6 -0.611173 1.708426 -3.191445
6 -4.013997 -2.062952 1.645284
6 -1.893642 -2.163625 -3.010682
1 -1.057017 -1.674290 0.874740
1 0.089765 -1.331466 -0.904269
6 2.193593 -2.474202 0.562044
6 1.473506 -1.887296 2.672583
6 3.069273 -3.423478 1.082919
1 2.096560 -2.335054 -0.509804
6 2.324953 -2.810342 3.271256
1 0.801053 -1.288278 3.281829
6 3.149945 -3.594810 2.463628
1 3.677122 -4.012202 0.395370
1 2.327141 -2.911949 4.357608
1 3.829764 -4.329027 2.901098
6 1.020578 2.458339 0.340946
6 2.501070 1.197822 -0.899631
6 1.758574 3.624305 0.174777
1 0.105338 2.480473 0.921295
6 3.264612 2.347993 -1.164127
6 2.901344 3.575064 -0.620932
1 1.414947 4.548146 0.638895
1 4.148016 2.250746 -1.794058
1 3.496943 4.469993 -0.813861
7 1.413747 -1.695959 1.338761
7 1.358200 1.264292 -0.180236
6 -1.717448 0.559885 2.942477
6 0.511094 1.129709 3.098724
6 -1.976893 1.038259 4.224590
1 -2.506704 0.123747 2.334146
6 0.338393 1.638491 4.384255
1 1.492396 1.135201 2.624298
6 -0.929735 1.587117 4.966099
1 -2.989039 0.977514 4.627485
1 1.192805 2.059677 4.916556
1 -1.098606 1.970349 5.974687
7 -0.495684 0.591167 2.384213
6 3.022011 -0.092105 -1.434116
6 4.263592 -0.593618 -0.969597
6 2.323785 -0.749405 -2.456104
6 4.766061 -1.751790 -1.594123
6 2.851819 -1.891897 -3.058070
1 1.361754 -0.360124 -2.779841
6 4.083422 -2.391233 -2.630337
1 5.714524 -2.176119 -1.258679
1 2.305616 -2.386361 -3.862627
1 4.510072 -3.280337 -3.100475
14 5.264500 0.100524 0.502899
6 4.136550 0.741482 1.875779
1 3.609813 1.665511 1.599773
1 3.393623 -0.020477 2.150629
1 4.747377 0.956757 2.767938
6 6.459680 1.454328 -0.042894
1 5.950325 2.396615 -0.291448
1 7.175402 1.666430 0.768609
1 7.038959 1.135182 -0.924260
6 6.280111 -1.318850 1.219967
1 7.052078 -1.678797 0.521760
1 6.799715 -0.971943 2.128135
1 5.643741 -2.172013 1.500298
1 -2.971740 2.325945 1.226576
1 -0.476552 0.785612 -2.605764
1 -1.666264 -1.127940 -2.729083
1 -3.813880 -0.996024 1.468310
6 -5.540055 -2.225768 1.719498
1 -6.023445 -1.895856 0.787055
1 -5.820144 -3.277981 1.884750
1 -5.956846 -1.632520 2.549012
6 -3.340687 -2.458279 2.967220
1 -3.703265 -1.826117 3.793488
1 -3.563392 -3.502936 3.233491
1 -2.247376 -2.353474 2.901861
6 -2.993247 -2.124604 -4.084777
1 -2.645880 -1.584760 -4.979633
1 -3.277962 -3.143508 -4.391680
1 -3.899198 -1.620086 -3.716820
6 -0.599793 -2.768955 -3.563585
1 0.159673 -2.856007 -2.774502
1 -0.762660 -3.768232 -3.996526
1 -0.196268 -2.130746 -4.365988
6 -1.792746 1.492700 -4.154734
1 -1.986892 2.407715 -4.736404
1 -1.559676 0.684141 -4.864453
1 -2.717292 1.217512 -3.632222
6 0.668301 1.938956 -4.003384
1 1.534842 2.149719 -3.361734
1 0.897870 1.045709 -4.602735
1 0.554413 2.774972 -4.710665
6 -4.257278 4.015778 0.919940
1 -4.882841 3.816823 1.804355
1 -4.201169 5.107526 0.784918
1 -4.770857 3.597115 0.042515
6 -2.192987 3.956720 2.374518
1 -2.796285 3.701139 3.257952
1 -1.186236 3.539263 2.523984
1 -2.102498 5.053345 2.348310

Prod
6 -3.037967 0.625206 -1.089221
6 -1.733967 0.134852 -1.128169
6 -1.279073 -0.682578 -0.079568
6 -3.323977 -0.574010 0.964976
6 -3.853064 0.276616 -0.010755
1 -3.414214 1.265320 -1.891179
1 -1.069271 0.365502 -1.960595
1 -3.942728 -0.888968 1.813634
1 -4.879876 0.639553 0.068545
6 0.114927 -1.211167 -0.081521
6 0.279553 -2.604546 -0.057941
6 1.241440 -0.353812 -0.113494
6 1.549562 -3.175021 -0.126042
1 -0.609058 -3.235975 0.006764
6 2.511574 -0.960680 -0.180451
6 2.671707 -2.347409 -0.203143

1 1.662897 -4.261917 -0.119458
1 3.407335 -0.335697 -0.197081
1 3.673481 -2.780490 -0.261264
7 -2.077492 -1.047079 0.934423
14 1.171089 1.535241 0.132246
6 2.831095 2.070975 0.862693
1 3.665390 1.925932 0.157742
1 2.794417 3.144484 1.111494
1 3.064767 1.516945 1.785863
6 0.950792 2.474736 -1.494760
1 -0.060130 2.363792 -1.913524
1 1.127849 3.551100 -1.331619
1 1.674074 2.125899 -2.249810
6 -0.184251 2.009740 1.358204
1 -0.176753 1.335074 2.228869
1 -0.011973 3.037258 1.719735
1 -1.189111 1.970965 0.914629

7'

77 -0.074239 -0.614613 -0.327441
6 0.978888 3.488414 -0.522902
6 2.210078 3.009155 -0.258748
1 0.630742 4.496887 -0.712126
1 3.167109 3.508018 -0.174584
6 0.766700 1.220753 -0.216758
7 0.100367 2.412954 -0.468131
7 2.081765 1.636031 -0.060976
6 3.236539 0.902232 0.397870
6 4.343771 0.733657 -0.466322
6 3.291743 0.518604 1.759375
6 5.459878 0.042460 0.031946
6 4.439679 -0.144412 2.206469
6 5.505949 -0.404349 1.347668
1 6.319708 -0.117053 -0.620047
1 4.506495 -0.463595 3.245733
1 6.388371 -0.932296 1.716350
6 -1.313234 2.704077 -0.365797
6 -1.777188 3.213283 0.867196
6 -2.143184 2.621535 -1.503982
6 -3.136153 3.543514 0.965865
6 -3.493359 2.955674 -1.347398
6 -3.992127 3.395054 -0.121079
1 -3.525168 3.931857 1.908529
1 -4.162189 2.897315 -2.205861
1 -5.049129 3.653313 -0.024081
14 0.834320 -0.881846 -2.519585
6 2.331430 -2.063727 -2.725537
1 2.114238 -3.116721 -2.493393
1 2.627654 -2.033077 -3.787746
1 3.209083 -1.770309 -2.134561
6 -0.476763 -1.627175 -3.687270
1 -1.345905 -0.958280 -3.777608
1 -0.045016 -1.741189 -4.696114
1 -0.842383 -2.613924 -3.367896
6 1.386443 0.708200 -3.442853
1 0.668865 0.933734 -4.246388
1 1.471631 1.609706 -2.822544
1 2.359088 0.541560 -3.928421
6 2.196352 0.894014 2.747502
1 1.233611 0.837463 2.221037
6 4.447547 1.384067 -1.840280
1 3.442964 1.692890 -2.148865
6 -1.567384 2.293494 -2.870016
1 -0.761345 1.573199 -2.705761
6 -0.844028 3.522375 2.032348
1 0.116262 3.030680 1.838876
1 -1.166770 0.126603 -1.157355
6 -0.165037 -3.645075 -0.893196
6 -2.352092 -3.026732 -0.484930
6 -0.474848 -4.995588 -0.949135
1 0.857667 -3.329269 -1.054374
6 -2.729372 -4.383664 -0.493278
6 -1.792606 -5.384039 -0.715602
1 0.313067 -5.717387 -1.167808
1 -3.784081 -4.628097 -0.369360
1 -2.089775 -6.434687 -0.735961
6 -1.128602 -0.633638 2.911546
6 -2.646918 0.292790 1.458255
6 -2.020821 -0.558932 3.978771
1 -0.121835 -1.010012 3.069517
6 -3.594172 0.434624 2.474068
1 -2.871341 0.614897 0.445557
6 -3.290742 -0.020737 3.753690
1 -1.712140 -0.900947 4.968092
1 -4.554985 0.890966 2.239847
1 -4.016368 0.059801 4.566075
6 0.791237 -2.897408 1.768451
6 2.643815 -2.205684 0.600591
6 1.487806 -3.948376 2.356306
1 -0.273268 -2.775358 1.958840
6 3.412211 -3.248576 1.118140
1 3.076050 -1.503591 -0.103731
6 2.831984 -4.133529 2.024009
1 0.972710 -4.619142 3.045767
1 4.453445 -3.344942 0.809454
1 3.407976 -4.955508 2.454861
7 1.352664 -2.017980 0.915174
7 -1.437472 -0.250048 1.661019
7 -1.053828 -2.659128 -0.635756
6 -3.458754 -2.044122 -0.401012
6 -4.451237 -2.190885 0.581320
6 -3.626710 -1.078653 -1.404070
6 -5.581227 -1.373499 0.575064
1 -4.326796 -2.939281 1.366882
6 -4.762537 -0.269222 -1.413616
1 -2.872007 -0.986895 -2.182899
6 -5.739923 -0.409830 -0.424266
1 -6.339477 -1.489476 1.352472
1 -4.893449 0.467966 -2.205826
1 -6.629173 0.224223 -0.439734
6 5.334320 2.642592 -1.750772
1 5.336276 3.179035 -2.712276
1 5.003746 3.344207 -0.971388
1 6.374396 2.367589 -1.515125
6 5.003744 0.459037 -2.930800
1 4.966776 0.969968 -3.904937
1 6.056837 0.196227 -2.746155
1 4.430657 -0.470721 -3.016315
6 2.401410 2.345430 3.221483
1 1.604010 2.639062 3.920271

1 3.365920 2.445089 3.743451
1 2.396895 3.057362 2.384882
6 2.117780 -0.038879 3.960774
1 2.992301 0.071718 4.619820
1 1.233175 0.209413 4.565735
1 2.052067 -1.096684 3.665135
6 -0.589003 5.039208 2.107106
1 -1.512956 5.577063 2.372286
1 0.166393 5.264687 2.876125
1 -0.233468 5.448413 1.151129
6 -1.339089 3.011026 3.388271
1 -2.328147 3.415989 3.650619
1 -1.402065 1.919054 3.410682
1 -0.642671 3.323958 4.181276
6 -0.950072 3.552715 -3.504303
1 -0.501778 3.308693 -4.479691
1 -1.718920 4.325218 -3.664696
1 -0.159573 3.986320 -2.876963
6 -2.561096 1.657443 -3.844017
1 -3.038526 0.765461 -3.419245
1 -3.353433 2.359490 -4.147751
1 -2.036720 1.349448 -4.760744

18‡-o

77 -0.088346 -0.565589 -0.100321
1 -1.414411 0.397339 -0.073149
6 -1.330870 -3.133869 -1.234628
6 -1.536694 -3.010522 1.068749
6 -1.979024 -4.365429 -1.267425
1 -0.983671 -2.646485 -2.146169
6 -2.192444 -4.236472 1.120301
1 -1.361619 -2.423167 1.969696
1 -2.134517 -4.867106 -2.223708
1 -2.527817 -4.628544 2.081233
7 -1.112730 -2.466568 -0.085774
6 -2.419669 -4.930205 -0.069258
1 -2.935043 -5.893124 -0.063473
6 -1.124603 0.715954 -2.594323
6 -1.860211 0.747121 -3.778365
6 -1.612513 0.078070 -1.437956
6 -3.139969 0.187608 -3.821931
1 -1.432742 1.216486 -4.667619
6 -2.954397 -0.391888 -1.459281
6 -3.685068 -0.353688 -2.659238
1 -3.726936 0.214479 -4.742144
1 -4.716562 -0.711415 -2.666145
6 2.269501 -2.448518 0.021886
6 1.435178 -1.927051 2.121011
6 3.099137 -3.430830 0.552678
1 2.259711 -2.233446 -1.044651
6 2.230156 -2.897716 2.722711
1 0.767851 -1.296576 2.705616
6 3.079797 -3.668762 1.927426
1 3.751694 -3.994212 -0.115005
1 2.183751 -3.033296 3.804198
1 3.718115 -4.435221 2.371841
7 1.446305 -1.708445 0.790549
6 0.866646 1.190795 -0.144455
6 2.332458 2.880981 -0.606293
6 1.210762 3.448871 -0.102967
1 3.259373 3.317104 -0.962304
1 0.957985 4.486047 0.085993
7 0.326579 2.414222 0.185716
7 2.125297 1.503500 -0.606070
6 -0.911419 2.624954 0.897418
6 -0.974506 2.239115 2.256896
6 -1.988697 3.254361 0.244630
6 -2.165599 2.483536 2.946114
6 -3.161878 3.467947 0.983180
6 -3.253498 3.086884 2.316033
1 -2.245520 2.209053 3.998212
1 -4.015425 3.946310 0.501492
1 -4.176132 3.265290 2.872862
6 3.207026 0.578821 -0.832296
6 4.106602 0.350240 0.235173
6 3.374865 -0.015170 -2.096127
6 5.168800 -0.533138 0.011531
6 4.453270 -0.896747 -2.266183
6 5.339226 -1.157290 -1.224245
1 5.878544 -0.733089 0.815353
1 4.605019 -1.375292 -3.235592
1 6.177153 -1.841171 -1.378171
6 -3.618149 -0.858057 -0.214910
6 -4.570414 -1.892378 -0.214324
6 -3.837907 -0.585186 2.066354
6 -5.161564 -2.266624 0.990960
1 -4.822451 -2.409940 -1.140385
6 -4.791428 -1.602968 2.162880
1 -3.521915 -0.026701 2.953759
1 -5.898760 -3.072585 1.016388
1 -5.232801 -1.860764 3.127456
7 -3.271391 -0.225881 0.916920
1 -0.131494 1.157359 -2.581733
6 0.228271 1.648804 2.980396
1 0.783501 1.047025 2.248514
6 -1.912900 3.745782 -1.192789
1 -1.059683 3.247004 -1.673066
6 3.980246 1.075896 1.568436
1 2.941784 1.420800 1.663912
6 2.437557 0.261698 -3.258229
1 1.750944 1.056378 -2.937418
6 4.893129 2.315080 1.601629
1 4.685354 3.006339 0.774250
1 4.757277 2.866835 2.544915
1 5.951175 2.016938 1.530776
6 4.278668 0.184534 2.779746
1 3.692895 -0.742166 2.759107
1 5.343599 -0.089228 2.835185
1 4.037596 0.722938 3.709372
6 3.190103 0.782769 -4.490787
1 3.870415 0.023048 -4.906294
1 2.477908 1.056157 -5.284881
1 3.789962 1.673140 -4.247679
6 1.594451 -0.978433 -3.588715
1 0.997254 -1.275820 -2.712386
1 0.894596 -0.769302 -4.411566
1 2.232895 -1.823772 -3.891634
6 -1.665235 5.264172 -1.238819
1 -1.574160 5.609668 -2.280654
1 -2.502280 5.808815 -0.774128
1 -0.749065 5.555674 -0.704451

6 -3.170014 3.390977 -1.998419
1 -3.006247 3.596450 -3.066766
1 -3.432709 2.331111 -1.891672
1 -4.035823 3.992331 -1.680360
6 1.163126 2.763477 3.480307
1 1.510100 3.401532 2.654778
1 0.646887 3.407071 4.209956
1 2.051340 2.335769 3.971915
6 -0.153683 0.704742 4.124785
1 -0.860680 -0.068879 3.785646
1 0.746181 0.208981 4.521572
1 -0.617920 1.236917 4.968960

18‡-m

77 -0.051851 -0.416892 -0.364843
1 0.976629 0.850047 -0.434217
6 1.871076 -2.805580 -0.521431
6 1.654801 -1.722833 -2.557276
6 2.835707 -3.667221 -1.033917
1 1.562691 -2.853176 0.523383
6 2.610700 -2.546697 -3.144976
1 1.171442 -0.919632 -3.116171
1 3.287210 -4.418220 -0.384618
1 2.878770 -2.401232 -4.192465
7 1.295808 -1.843740 -1.266208
6 3.217032 -3.536031 -2.369221
1 3.979940 -4.189743 -2.797284
6 1.472701 0.839154 1.988329
6 2.548816 0.904180 2.872765
6 1.642211 0.325298 0.686806
6 3.825971 0.503476 2.475541
1 2.386644 1.266705 3.890686
6 2.950436 0.008814 0.280110
6 4.040718 0.060501 1.160333
1 4.645758 0.532193 3.194613
6 -1.962109 -2.774160 -0.358094
6 -1.826630 -1.730405 -2.424318
6 -2.708038 -3.820437 -0.893874
1 -1.709200 -2.747416 0.700624
6 -2.568114 -2.739261 -3.029863
1 -1.459098 -0.878173 -2.992340
6 -3.017798 -3.809948 -2.253658
1 -3.042233 -4.624532 -0.237538
1 -2.789390 -2.675843 -4.096194
1 -3.602775 -4.616541 -2.700503
7 -1.523259 -1.745935 -1.109865
6 -1.265321 0.929284 0.477663
6 -2.708839 1.993791 1.890049
6 -2.068680 2.950103 1.174235
1 -3.464506 2.063157 2.665382
1 -2.152088 4.031326 1.190853
7 -1.201301 2.294069 0.305458
7 -2.228565 0.763923 1.444941
6 -0.472753 2.968616 -0.743019
6 -0.933868 2.824194 -2.074549
6 0.631533 3.779630 -0.415537
6 -0.220375 3.479718 -3.082713
6 1.305046 4.419521 -1.467799
6 0.894010 4.265325 -2.785990
1 -0.544598 3.390421 -4.119291
1 2.165851 5.050739 -1.243514
1 1.434829 4.769420 -3.590031
6 -2.884542 -0.474723 1.782525
6 -4.078933 -0.784375 1.092900
6 -2.365756 -1.303766 2.794570
6 -4.739449 -1.970910 1.432866
6 -3.059020 -2.488121 3.087456
6 -4.233824 -2.819919 2.416587
1 -5.664885 -2.235248 0.918880
1 -2.676050 -3.152045 3.864741
1 -4.764811 -3.740923 2.668240
1 0.491745 1.164281 2.325715
6 -2.207345 2.055879 -2.405077
1 -2.244457 1.180589 -1.741703
6 1.083940 4.032806 1.014183
1 0.611329 3.277722 1.654996
6 -4.673738 0.145040 0.044470
1 -3.892458 0.853151 -0.258256
6 -1.096902 -0.968385 3.555874
1 -0.778664 0.028990 3.232033
6 -5.835114 0.961099 0.636957
1 -5.522771 1.533713 1.521783
1 -6.224728 1.673085 -0.107321
1 -6.662426 0.300544 0.941329
6 -5.127145 -0.591821 -1.221479
1 -4.318651 -1.197401 -1.649369
1 -5.982910 -1.256728 -1.027841
1 -5.449055 0.133891 -1.984285
6 -1.334900 -0.903435 5.070988
1 -1.614736 -1.885120 5.484360
1 -0.417435 -0.578717 5.586159
1 -2.137756 -0.192946 5.321189
6 0.029666 -1.948036 3.197122
1 0.220596 -1.921855 2.112962
1 0.965324 -1.669046 3.703908
1 -0.227686 -2.978197 3.491159
6 0.611192 5.417062 1.492237
1 0.899711 5.583262 2.542059
1 1.064733 6.217305 0.886358
1 -0.481062 5.530030 1.418037
6 2.603750 3.895264 1.181940
1 2.871233 3.933832 2.248388
1 2.971978 2.943418 0.775792
1 3.141472 4.714866 0.680520
6 -3.448394 2.920710 -2.119281
1 -3.490776 3.244412 -1.070083
1 -3.444062 3.822674 -2.751148
1 -4.370686 2.359162 -2.335291
6 -2.252416 1.529873 -3.843318
1 -1.332479 0.985404 -4.108613
1 -3.108698 0.849218 -3.967554
1 -2.378617 2.341234 -4.576475
1 3.159063 -0.315809 -0.735637
6 5.371423 -0.384447 0.665544
6 6.568527 -0.034538 1.314554
6 7.779917 -0.496308 0.802373
1 6.555587 0.605145 2.197346
6 6.534314 -1.575899 -0.931568
6 7.770920 -1.289380 -0.346228
1 8.720758 -0.233026 1.291205
1 6.483534 -2.189059 -1.839234

1 8.695178 -1.672355 -0.782757
7 5.373580 -1.142956 -0.445017

18‡-p

77 -0.446854 -0.408652 -0.738919
1 0.726369 0.660665 -1.122778
6 0.433163 -3.024814 -2.070373
6 -0.289158 -1.457710 -3.616699
6 0.679859 -3.960567 -3.071663
1 0.631992 -3.245364 -1.020787
6 -0.065194 -2.334622 -4.674722
1 -0.662834 -0.445382 -3.780976
1 1.069234 -4.944583 -2.806294
1 -0.272622 -2.013326 -5.696577
7 -0.043988 -1.795209 -2.336866
6 0.427899 -3.612107 -4.399796
1 0.615348 -4.322623 -5.207773
6 2.168225 0.050875 0.775464
6 3.504778 -0.168715 1.079322
6 1.652157 -0.190631 -0.515074
6 4.415817 -0.591111 0.095494
1 3.883414 0.004123 2.087077
6 2.582213 -0.563488 -1.510584
6 3.927025 -0.770879 -1.209155
1 4.604557 -1.046238 -2.018972
6 -2.683417 -2.370378 -0.131115
6 -3.201247 -0.928797 -1.872051
6 -3.813178 -3.143225 -0.385589
1 -2.003488 -2.617610 0.682733
6 -4.348146 -1.650634 -2.184880
1 -2.921039 -0.039314 -2.432802
6 -4.663040 -2.783953 -1.431400
1 -4.015880 -4.010380 0.243687
1 -4.983375 -1.318452 -3.007199
1 -5.557163 -3.370580 -1.652503
7 -2.376692 -1.282571 -0.864388
6 -0.810284 0.889181 0.741962
6 -1.196716 1.817902 2.790676
6 -0.705970 2.796414 1.991814
1 -1.504437 1.827595 3.830917
1 -0.500146 3.842983 2.187609
7 -0.486219 2.227128 0.741143
7 -1.276729 0.663447 2.015185
6 -0.120902 3.000089 -0.422711
6 -1.114455 3.234897 -1.404927
6 1.177849 3.537161 -0.524818
6 -0.752084 3.985504 -2.527506
6 1.482142 4.289397 -1.670479
6 0.536277 4.503722 -2.665230
1 -1.489438 4.185711 -3.304559
1 2.481210 4.713633 -1.777832
1 0.796020 5.089569 -3.549801
6 -2.005880 -0.498622 2.458495
6 -3.415606 -0.453211 2.364921
6 -1.324268 -1.601535 3.006193
6 -4.135200 -1.564845 2.818897
6 -2.092099 -2.696023 3.432818
6 -3.482030 -2.680095 3.342451
1 -5.224719 -1.558251 2.761421
1 -1.589110 -3.567240 3.856514
1 -4.061642 -3.537597 3.692176
1 1.508821 0.401399 1.565008
6 -2.552908 2.767361 -1.220306
1 -2.517586 1.779266 -0.740810
6 2.234046 3.385566 0.559495
1 1.911383 2.582666 1.233995
6 -4.152282 0.769461 1.836376
1 -3.429145 1.383802 1.285489
6 0.186885 -1.647036 3.136604
1 0.567449 -0.660129 2.849694
6 -4.696610 1.620121 2.996701
1 -3.899940 1.925063 3.689742
1 -5.178634 2.532507 2.612175
1 -5.446096 1.057453 3.575586
6 -5.279430 0.416371 0.858745
1 -4.920162 -0.212490 0.034844
1 -6.105430 -0.115055 1.355908
1 -5.702788 1.335681 0.425372
6 0.634207 -1.905090 4.582164
1 0.334735 -2.905540 4.931976
1 1.731138 -1.845052 4.655487
1 0.203768 -1.164732 5.273918
6 0.788330 -2.671474 2.163604
1 0.505815 -2.420717 1.129229
1 1.886988 -2.660694 2.217323
1 0.440882 -3.691533 2.393897
6 2.349289 4.677797 1.387190
1 3.085266 4.552093 2.196676
1 2.679918 5.518740 0.757347
1 1.389879 4.967748 1.841988
6 3.604292 2.988846 -0.009092
1 4.290097 2.711273 0.804925
1 3.526561 2.129545 -0.688414
1 4.068341 3.821722 -0.560161
6 -3.314583 3.722180 -0.283444
1 -2.835809 3.798261 0.702676
1 -3.359275 4.733930 -0.716080
1 -4.347447 3.372631 -0.129438
6 -3.318556 2.597865 -2.536323
1 -2.756269 1.987098 -3.260362
1 -4.287485 2.110689 -2.346871
1 -3.534794 3.564200 -3.017066
1 2.254569 -0.682122 -2.543706
6 5.845239 -0.774736 0.454086
6 6.755943 -1.432574 -0.392372
6 8.086038 -1.556580 0.005536
1 6.430167 -1.854981 -1.343146
6 7.500062 -0.401453 2.017933
6 8.476349 -1.028237 1.236676
1 8.808147 -2.064979 -0.637663
1 7.763184 0.021057 2.994476
1 9.506986 -1.101330 1.588663
7 6.229804 -0.277652 1.642199

H2

1 0.000000 0.000000 0.380436
1 0.000000 0.000000 -0.380436

NBE

6 -1.186998 -0.778740 -0.519202
6 0.085525 -1.126550 0.323726
6 0.085995 1.126523 0.323722

6 -1.186586 0.779168 -0.519337
1 -1.136659 -1.208734 -1.529886
1 -2.089957 -1.178995 -0.032290
1 -1.135920 1.208969 -1.530087
1 -2.089405 1.179954 -0.032604
6 0.036434 0.000003 1.379978
1 0.910611 -0.000174 2.047501
1 -0.889054 0.000200 1.978889
6 1.278082 -0.671619 -0.504362
6 1.278527 0.671142 -0.504138
1 0.115641 -2.161192 0.691358
1 0.116449 2.161160 0.691342
1 1.926425 1.332178 -1.083314
1 1.925984 -1.332927 -1.083225

NBA
6 1.252562 0.780666 -0.493351
6 0.000107 1.130961 0.339114
6 0.000146 -1.130962 0.339112
6 1.252601 -0.780623 -0.493331
1 1.204086 1.208182 -1.506772
1 2.161931 1.178571 -0.016417
1 1.204185 -1.208172 -1.506741
1 2.161973 -1.178467 -0.016349
6 0.001091 -0.000001 1.387494
1 -0.894169 -0.000016 2.029847
1 0.897496 0.000012 2.028376
6 -1.253221 0.780803 -0.492381
6 -1.253182 -0.780844 -0.492402
1 0.000173 2.158265 0.731679
1 0.000241 -2.158266 0.731675
1 -2.162207 -1.178269 -0.014377
1 -2.162250 1.178166 -0.014309
1 -1.205993 -1.208080 -1.506086
1 -1.206090 1.208073 -1.506052

py
6 -1.143139 -0.724829 -0.000221
6 -1.199930 0.672541 -0.000114
6 0.000093 1.386165 0.000081
6 1.200019 0.672392 0.000215
6 1.143043 -0.724969 0.000142
7 -0.000092 -1.415775 -0.000106
1 0.000138 2.479051 0.000155
1 -2.068393 -1.312990 -0.000377
1 -2.164359 1.185063 -0.000210
1 2.164533 1.184755 0.000369
1 2.068215 -1.313252 0.000190

Ph-py
6 -2.695275 -1.228791 -0.000039
6 -3.523154 -0.102621 0.000013
6 -2.910189 1.151600 0.000061
6 -1.519429 1.225576 0.000056
6 -0.763145 0.037161 -0.000043
7 -1.366959 -1.162969 -0.000071
1 -3.509003 2.065680 0.000110
1 -3.131829 -2.234694 -0.000073
1 -4.609675 -0.209046 0.000032
1 -1.031044 2.199443 0.000140
6 0.726136 0.037076 -0.000020
6 1.413352 -1.190188 0.000033
6 1.481693 1.223130 -0.000061
6 2.806866 -1.229745 0.000070
1 0.822533 -2.105995 0.000047
6 2.876440 1.183386 -0.000048
1 0.987662 2.195514 -0.000129
6 3.546328 -0.043246 0.000026
1 3.320538 -2.194293 0.000121
1 3.443273 2.117438 -0.000078
1 4.638513 -0.073301 0.000040

HSiR3
14 0.000004 0.000008 -0.384102
1 0.000028 0.000020 -1.882120
6 -1.332832 1.190173 0.225343
1 -2.331448 0.887951 -0.130041
1 -1.360163 1.214390 1.327637
1 -1.144945 2.216546 -0.129798
6 1.697150 0.559154 0.225350
1 1.731903 0.570378 1.327640
1 2.492095 -0.116572 -0.130078
1 1.934610 1.575215 -0.129763
6 -0.364327 -1.749337 0.225338
1 -1.346973 -2.099938 -0.130132
1 0.396916 -2.462994 -0.129726
1 -0.372031 -1.785045 1.327629

References

[1] SAINT+, version 6.01; Bruker AXS, Inc.; Madison, WI, 2001.

[2] G. M. Sheldrick, SABADS, University of Göttingen: Göttingen, Germany, 1999.

[3] (*a*) G. M. Sheldrick, SHELXL–97, University of Göttingen: Göttingen, Germany, 1997; (*b*) G. M. Sheldrick, *Acta Crystallogr.*, **2008**, *A64*, 112.

[4] L. J. Farrugia, *J. Appl. Crystallogr.*, 1999, 32, 837.

[5] Gaussian 09, Revision D.01, M. J. Frisch, G. W. Trucks, H. B. Schlegel, G. E. Scuseria, M. A. Robb, J. R. Cheeseman, G. Scalmani, V. Barone, B. Mennucci, G. A. Petersson, H. Nakatsuji, M. Caricato, X. Li, H. P. Hratchian, A. F. Izmaylov, J. Bloino, G. Zheng, J. L. Sonnenberg, M. Hada, M. Ehara, K. Toyota, R. Fukuda, J. Hasegawa, M. Ishida, T. Nakajima, Y. Honda, O. Kitao, H. Nakai, T. Vreven, J. A. Montgomery, Jr. , J. E. Peralta, F. Ogliaro, M. Bearpark, J. J. Heyd, E. Brothers, K. N. Kudin, V. N. Staroverov, R. Kobayashi, J. Normand, K. Raghavachari, A. Rendell, J. C. Burant, S. S. Iyengar, J. Tomasi, M. Cossi, N. Rega, J. M. Millam, M. Klene, J. E. Knox, J. B. Cross, V. Bakken, C. Adamo, J. Jaramillo, R. Gomperts, R. E. Stratmann, O. Yazyev, A. J. Austin, R. Cammi, C. Pomelli, J. W. Ochterski, R. L. Martin, K. Morokuma, V. G. Zakrzewski, G. A. Voth, P. Salvador, J. J. Dannenberg, S. Dapprich, A. D. Daniels, C. Farkas, J. B. Foresman, J. V. Ortiz, J. Cioslowski, D. J. Fox, Gaussian, Inc., Wallingford CT, 2009.

[6] (a) C. Lee, W. Yang, R. G. Parr, Phys. Rev. B 1988, 37, 785–789. (b) A. D. Becke, *J. Chem. Phys.* **1993**, *98*, 1372–1377. (c) A. D. Becke, *J. Chem. Phys.* **1993**, *98*, 5648–5652.

[7] S. Grimme, J. Antony, S. Ehrlich, H. Krieg, *J. Chem. Phys.* **2010**, *132*, 154104.

[8] F. Weigend, R. Ahlrichs, *Phys. Chem. Chem. Phys.* **2005**, *7*, 3297–3305.

[9] J. Tomasi, B. Mennucci, R. Cammi, *Chem. Rev.* **2005**, *105*, 2999–3093.

[10] R. Tanaka, M. Yamashita, L. W. Chung, K. Morokuma, K. Nozaki, *Organometallics* **2011**, *30*, 6742–6750.
